# Distinct or Overlapping Areas of Mitochondrial Thioredoxin 2 May Be Used for Its Covalent and Strong Non-Covalent Interactions with Protein Ligands

**DOI:** 10.3390/antiox13010015

**Published:** 2023-12-20

**Authors:** Charalampos Ntallis, Haralampos Tzoupis, Theodore Tselios, Christos T. Chasapis, Alexios Vlamis-Gardikas

**Affiliations:** 1 Department of Chemistry, University of Patras, 26504 Rion, Greece; c.ntallis@uu.nl (C.N.); htzoupis@upatras.gr (H.T.); ttselios@upatras.gr (T.T.); 2 Institute of Chemical Biology, National Hellenic Research Foundation, Vas. Constantinou 48, 11635 Athens, Greece; cchasapis@eie.gr

**Keywords:** thioredoxin, mitochondria, hot spots, contact area, interface, molecular recognition

## Abstract

In silico approaches were employed to examine the characteristics of interactions between human mitochondrial thioredoxin 2 (HsTrx2) and its 38 previously identified mitochondrial protein ligands. All interactions appeared driven mainly by electrostatic forces. The statistically significant residues of HsTrx2 for interactions were characterized as “contact hot spots”. Since these were identical/adjacent to putative thermodynamic hot spots, an energy network approach identified their neighbors to highlight possible contact interfaces. Three distinct areas for binding emerged: (i) one around the active site for covalent interactions, (ii) another antipodal to the active site for strong non-covalent interactions, and (iii) a third area involved in both kinds of interactions. The contact interfaces of HsTrx2 were projected as respective interfaces for *Escherichia coli* Trx1 (EcoTrx1), 2, and HsTrx1. Comparison of the interfaces and contact hot spots of HsTrx2 to the contact residues of EcoTx1 and HsTrx1 from existing crystal complexes with protein ligands supported the hypothesis, except for a part of the cleft/groove adjacent to Trp^30^ preceding the active site. The outcomes of this study raise the possibility for the rational design of selective inhibitors for the interactions of HsTrx2 with specific protein ligands without affecting the entirety of the functions of the Trx system.

## 1. Introduction

Thioredoxins (Trxs) are small proteins initially discovered in *Escherichia coli* (*E. coli*) as electron donors to ribonucleotide reductase [1]. The whole assortment for the transfer of electrons involves NADPH as the prime source from which they are delivered first to Trx reductase (TrxR) and then to Trx. All three constitute the Trx system, which is ubiquitous in all organisms, including viruses ([2] and references therein). A backup system in which TrxR is replaced by glutathione reductase and glutathione (GSH) while Trx is substituted by glutaredoxin (Grx) is the Grx system [3]. The redox action of Trxs and Grxs is mediated by their active site of the CxxC type. An internal disulfide is formed in the active site upon the reduction of substrates. The active site disulfide will be reduced normally by TrxR for Trxs or GSH for Grxs. Electrons from NADPH keep the two systems reduced [4]. In terms of evolution, the Trx and Grx systems seem to have stemmed from a common ancestor molecule [5] and predate aerobiosis [6,7,8].

Mammalian cells possess two distinct Trx systems concerning the cytosol: HsTrx1 and TrxR1. The systems concerning mitochondria are HsTrx2 and TrxR2. Human HsTrx2 has two active site cysteines, whereas human HsTrx1 has two catalytic and three additional cysteines. The latter renders the molecule sensitive to redox regulation [9,10,11]. HsTrx2 combined with peroxiredoxin 3 constitute most likely the key molecules for the reduction of hydrogen peroxide in the mitochondria in vitro [12] and in an endothelial cell model of sepsis [13]. A common denominator of proteins belonging to the Trx/Grx superfamily is the Trx fold, consisting of four beta sheets surrounded by four alpha helices [14,15]. The Trx/Grx fold is a relatively common element in protein structure geometry and may be found in proteins that are mostly, but not always, involved in electron flow [16].

The involvement of Trxs and Grxs in the reduction of ribonucleotides and in antioxidant functions requires a constant electron flow to specific substrates via transient covalent bonds of the enzymes with the substrates [4,17]. However, Trxs and Grxs may interact with ligands in a non-covalent manner: *E. coli* Trx1 (EcoTrx1) and gene 5 protein (g5p) of phage T7 interact non-covalently to constitute the T7 DNA polymerase, essential for replication of the phage DNA [18]. Reduced HsTrx1 [19,20], human Grx1 [21], and reduced HsTrx2 may also bind non-covalently to mitochondrial ASK1 [22]. Reduced HsTrx1 may interact non-covalently with the T3SS effector of *Salmonella enterica* (SlrP), an E3 ubiquitin ligase for HsTrx1 [23]. Trx-interacting protein (TXNIP), a central molecule in the signal transduction processes, may also bind non-covalently to HsTrx1 [24,25].

The identification of electron acceptors of Trxs most often employs a monothiol Trx, which may become the bait for disulfide substrates [26,27,28,29]. An approach using affinity columns with immobilized monothiol mouse mitochondrial HsTrx2 combined with a yeast two-hybrid system screen presented a 53-membered mitochondrial interactome (52 interactor proteins plus the mitochondrial 16S RNA) for HsTrx2 [30]. The ligands implicated HsTrx2 in mitochondrial integrity, formation of iron-sulfur clusters, detoxification of aldehydes, mitoribosome assembly and protein synthesis, protein folding, ADP ribosylation, amino acid and lipid metabolism, glycolysis, the TCA cycle, and the electron transport chain [30]. Approximately half of the detected protein ligands of HsTrx2 bound non-covalently, while some bound both covalently (disulfides) and non-covalently [30]. Herein, molecular docking and network-based approaches were applied to map the most significant amino acid residues of HsTrx2 for its interaction with protein ligands. These “contact hot spots” were used to define interface areas for covalent and strong non-covalent interactions. The putative interface areas and contact hot spots of HsTrx2 were compared to contacts of EcoTrx1 and HsTrx1 in their known crystal complexes with protein ligands. Apart from a small subset of residues of the cleft/groove next to the active site, all proposed contact residues HsTrx2 matched those of the other Trxs.

## 2. Materials and Methods

### 2.1. Numbering and Sequences of the Residues of the Different Trxs

For residues concerning HsTrx2, the mitochondrial targeting signal sequence (residues 1–59) [31] was omitted, with the first amino acid being T (in sequence T^1^TF…). The numberings for the residue of EcoTrx1 correspond to a molecule of 108 amino acid residues as in the crystal structure of T7 DNA polymerase (without the first Met) [32]. In EcoTrx2, the amino acids 1–34 corresponding to an area participating in Zn binding (as implied from the sequence of Trx2 from *Rhodobacter capsulatus* [33]) were omitted. Number one is now a G from the G^1^EVI… sequence (G^35^E^36^V^37^I^38^… in the whole sequence). Whenever “EcoTrx2” is mentioned in this work, it is meant for the molecule without its first 34 amino acids. The sequence for HsTrx1 was as in [34].

### 2.2. The Model of HsTrx2 Used for Docking Studies

A monothiol form of HsTrx2 (ΔTrx2C93S) without its mitochondrial targeting signal (molecule starting as T^1^TF…) was employed to select potential ligands for HsTrx2 [30]. To simulate the intermolecular interaction of HsTrx2 with its ligands, the crystal structure of the reduced form of the HsTrx2 was used (PDB ID: 1w89, chain A from the crystal structure was selected, all water molecules were removed) [35] after having mutated Cys^34^ (or Cys^93^ if the mitochondrial signal is included) to serine and after having removed the mitochondrial signal with the structure editing tool of the ChimeraX software (version 1.3) [36]. The serine rotamer type was selected from the Dunbrack residue library [37] based on the highest prevalence value.

### 2.3. Selection of the Three-Dimensional Structures of the Protein Ligands of HsTrx2

The three-dimensional structures of the ligands of HsTrx2 were from the Uniprot Knowledgebase (UniprotKB), which contains a collection of functional and structural information on proteins (https://www.uniprot.org/, accessed on 1 September 2023). Fifty-two protein structures of protein ligands, preferably in high resolution, were selected for protein-protein docking with the monothiol HsTrx2 (from [30]). Structures were experimentally resolved (crystallographic, NMR, and cryo-EM structures from the PDB) or predicted (AlphaFold [38]) when no experimental structures were available.

### 2.4. Protein-Protein Docking Simulations

The docking simulations of HsTrx2 involved ligands whose binding was previously identified experimentally [30]. For the prediction of the protein-protein interactions (PPIs), the PRISM web server (http://cosbi.ku.edu.tr/prism/index.php, accessed on 1 September 2023 [39,40]) was used. The PRISM algorithm considers architectural features and evolutionary conservation characteristics on the interface of resolved complexes extracted from the respective PDBs. These resolved interfaces serve as templates for the prediction of not-yet-resolved complexes based on their structure and sequence similarity. The PRISM protocol includes rigid structural similarity, flexible refinement, and energy minimization. The binding energy scores were calculated using the CHARMM52 force field [39,40].

### 2.5. Calculation of Binding Affinities of HsTrx2-Ligand Complexes by PRODIGY

The complexes from the PRISM results were used as input structures for the PRODIGY web server (https://wenmr.science.uu.nl/prodigy/, accessed on 1 October 2023). The PRODIGY’s algorithm correlates the number of interfacial contacts (ICs) and the percentage of non-interacting surface (% NIS) per residue category (charged, polar, and apolar) in a protein-protein interface with the binding affinity (*K_D_*) of the complex [41,42,43]. The predictive model of the PRODIGY’s algorithm employs Equation (1) [42]:

ΔG_predicted_ = 0.09459 ICs_charged/charged_ + 0.10007 ICs_charged/apolar_ − 0.19577 ICs_polar/polar_ + 0.22671 ICs_polar/apolar_ − 0.18681 (% NIS _apolar_) − 0.13810 (% NIS_charged_) + 15.9433 (1)

Based on the predicted binding energy (ΔG), the equilibrium dissociation constant (*K_D_*) was calculated by Equation (2), where R is the ideal gas constant (kcal·K^−1^·mol^−1^), and T is the temperature (in K) (Equation (2)):

ΔG_predicted_ = R·T·ln*K_D_* (2)

All calculations of binding energy were performed at room temperature (298.15 K or 25 °C). The results obtained from PRODIGY and the IC and % NIS values for each complex are presented in detail in Supplementary Data S1.

The van der Waals (VdW), electrostatic, and desolvation energy contributions for the complexes of Trxs with protein ligands were performed via the HADDOCK2.4 webserver (https://wenmr.science.uu.nl/haddock2.4/, accessed on 1 October 2023) [44,45], based on the available PRISM complexes.

### 2.6. Mapping of the Interactions between HsTrx2 and Protein Ligands

The intermolecular interactions of the selected HsTrx2-ligand complexes (as detected by PRISM) were analyzed using the BIOVIA Discovery Studio Visualizer-2021 software (version v21.1.0.20298). For each of the 38 complexes presented in table of Section 3.1, the intermolecular interactions that participated in the molecular recognition of HsTrx2 with its substrates are presented in Supplementary Data S2, while some of them are discussed in the Results section. The interacting residues of HsTrx2 for each complex interface are shown in Supplementary Data S2.

The identity and percentage recurrence of HsTrx2 residues participating in interactions with ligands were analyzed with respect to (i) covalent bonding (disulfide bond formation, confirmed by DTT), (ii) strong non-covalent interaction (confirmed by acid elution), and (iii) total interactions, which included (i), (ii), the weak non-covalent interaction confirmed by yeast two-hybrid screenings, and three interactions from BioGRID (https://thebiogrid.org/, accessed on 1 March 2023). All contact residues of HsTrx2 derived by BIOVIA were considered validated contacts.

### 2.7. Contact Hot Spots

The definition of statistically significant contact hot spot residues (or contact hot spots) for HsTrx2 was based on the ranking of the residues of HsTrx2 participating in contacts with protein substrates (analysis of the selected 38 complexes of HsTrx2 by BIOVIA). The percentage contribution of each HsTrx2 residue participating in complexes with ligands was calculated by measuring first the total amount of contacts for all residues of HsTrx2 with all interacting ligands. In the second step, the percentage contribution of each residue of HsTrx2 was calculated by comparing its total contacts to the total hits of all other residues. The procedure was performed separately for strong non-covalent and covalent interactions, while it also included all contacts for total interactions (Supplementary Data S3). A “contact hot spot” is defined herein as any residue of HsTrx2 that appeared more frequently than others as a contact point with different proteins. This is not a thermodynamic definition.

### 2.8. Combinations of the Contact Residues of HsTrx2 for Interaction with Protein Ligands

This was calculated according to Equation (3):

(3) $$C(n,r)=\frac{n!}{\left(r!(n-r)!\right)}$$

where *n* is the total population/amount and *r* is the subset whose combinations are to be examined on *n*.

(https://www.calculatorsoup.com/calculators/discretemathematics/combinations.php, accessed on 1 August 2023).

### 2.9. Prediction of Possible Thermodynamic Hot Spot Residues of HsTrx2

The web server SpotOn (https://alcazar.science.uu.nl/cgi/services/SPOTON/spoton/, accessed on 1 September 2023) [46] was used to predict possible hot spots in the interface of the complexes of HsTrx2 with its identified protein ligands. The server identifies and classifies interfacial residues as thermodynamic hot spots and null spots [46,47]. A residue is considered a “thermodynamic hot spot” if its replacement with Ala changes the interacting energy for more than 2 kcal/mol. In comparison, the replacement of a “null spot” with Ala changes the binding energy by less than 2 kcal/mol [47]. The participation of residues of HsTrx2 in potential thermodynamic hot spots/null spots when in different complexes was presented as their percentage participation relative to all other residues in the 38 examined complexes.

Putative thermodynamic hot spot residues for complex formation of HsTrx2 with other proteins were also detected by PROT-ON (http://proton.tools.ibg.edu.tr:8001/, accessed on 1 September 2023) [48]. This web tool performs mutation scans on all residues that participate in protein-protein interfaces and proposes those that may affect mostly, or, inthe least, a given interaction [48]. Mutations that may result in lower ΔGs are called “enriching”, while mutations that may weaken a complex (resulting in new positive ΔΔGs) are called “depleting”. In this way, critical thermodynamic residues for the interaction of proteins in interfaces are highlighted. The inputs for the analyses by PROT-ON were the complexes detected earlier (Section 2.4) by PRISM.

### 2.10. Network Analysis

The model structure of the monothiol HsTrx2 was used as input in the NASP web server (https://bioinf.iiit.ac.in/NAPS/index.php, accessed on 1 September 2023) [49,50] for the identification of the centrality degree of the contact hot spots. The algorithm considers amino acid residues as nodes and connections between them as links/edges. The frameworks of links in the three-dimensional network of the nodes (C^α^s of participating residues) are user-defined and may present information on networks and subnetworks of participating residues (Protein Contact Network). In our study, the distance of 7 Å was considered the threshold for link formation: if the C^α^-C^α^ distance between two residues was less than 7 Å, an edge was drawn between the two nodes. The default value of 1 for residue separation of the protein’s backbone was used to capture short-range interactions.

The identification of energy network neighbors was performed by selecting first the “energy network” in the “network type” setting of the server. The force field was a type of CHARMM [51]. Edges were considered weighted. The interaction energy between residues was set between upper (1 kcal/mol) and lower (0.1 kcal/mol) default thresholds. The van der Waal and electrostatic interaction energies were considered for calculating the interaction energy between any 2 given residues [49,50]. The edge weights of the network were equal to the normalized interaction energies. Next, a visual analysis of the energy network followed: at the highlighted selection of “neighbors”, all neighbors to the residues of interest were selected. We coined these residues “energy neighbors”. Comparison and classification of residues detected as participating in covalent, strong non-covalent, and all interactions were performed using the web tool “venny” https://bioinfogp.cnb.csic.es/tools/venny/index2.0.2.html, accessed on 1 August 2023).

### 2.11. Comparative Analysis of the Statistical Hot Spot Residues of HsTrx2 with the Contact Residues of EcoTrx1 and HsTrx1 in Their Crystal Complexes with Ligands

The crystal structures of EcoTrx1 complexed to (i) g5p (PDB ID: 1T7P), (ii) *E. coli* TrxR (PDB ID: 1F6M), (iii) *E. coli* methionine sulfoxide reductase A (MsrA) (PDB ID: 6YEV), and (iv) *E. coli* PAPS reductase (PDB ID: 2O8V) were used to identify the residues of EcoTrx1 that interacted with the different protein ligands. The crystals of HsTrx1 with (i) the *Salmonella* T3SS effector SlrP (PDB ID: 4PUF), (ii) human TrxR (PDB ID: 3QFA), and (iii) Txnip (PDB ID: 4LL4) were used to the same aim. The intermolecular interactions at the interface of all complexes were analyzed using the BIOVIA Discovery Studio Visualizer-2021 software (version v21.1.0.20298). Superimposition of the structures was by the matchmaker tool of the ChimeraX software (version 1.3). The sequence alignment algorithm was Needleman–Wunsch, and the selected matrix was BLOSUM-62.

## 3. Results

### 3.1. Physicochemical Parameters of the Interactions of HsTrx2 with Ligands

From the 53 experimentally detected complexes of monothiol HsTrx2 with its substrates (as in [30]), the server confirmed 38 (>70% agreement with the experimental data). The acquired complexes by PRISM were analyzed by the PRODIGY web server to present the previously unknown interaction characteristics of the interactions (ΔGs and *K_D_*s) of HsTrx2 and its protein ligands. All complexes possessed negative docking values (Prism’s algorithm internal protocol, Table 1).

In general, docking scores were rather lower for strong non-covalent interactions, while ΔGs appeared similar for covalent and strong non-covalent interactions (Table 1). The ΔG and *K_D_* and for the simulated in silico interaction of *E. coli* Trx1 with g5p were −10.2 kcal/mol and 32 nM, respectively, while the simulated ΔG and *K_D_* values for the interaction of EcTrxR and EcoTrx1 were −12.3 kcal/mol and 0.96 nM. This is quite different from the experimentally determined ΔG and *K_D_* values for the interaction of EcoTrx1 with g5p (ΔG about 12 kcal/mol, *K_D_* of 2.2 nM [52], or even 5 nM [53]). The absolute values of the in silico ΔGs and *K_D_*s of Table 1 must be considered as a ranking of ligand preferences. All in silico ΔGs range from −5.8 to −12.1 kcal/mol, with reflecting *K_D_*s from 580 to 0.01 nM. The highest *K_D_*s were observed for isocitrate dehydrogenase [NAD] subunit alpha (58,000 nM), acetyl-coenzyme A synthetase 2-like protein (28,000 nM) and Elongation factor Tu (25,000 nM). The lowest was observed for peroxiredoxin-2 (0.23 nM), NADH dehydrogenase [ubiquinone] iron-sulfur protein 3 (1.3 nM), pyruvate dehydrogenase E1 component subunit alpha, somatic form, (4.5 nM), and O-acetyl-ADP-ribose deacetylase MACROD1 (chain A) (2.4 nM). In comparison, the *K_D_*s and ΔGs for complexes between a protease-inhibitor and antigen-antibody range from μM to 10 fM, corresponding to −8 and −19 kcal/mol, respectively [54]. The complexes of HsTrx2 and its ligands belong, therefore, to the middle and low range of the binding strength of known interactions, which is consistent with the dynamic exchanges occurring in the cytosol of a living cell.

**Figure 1:**
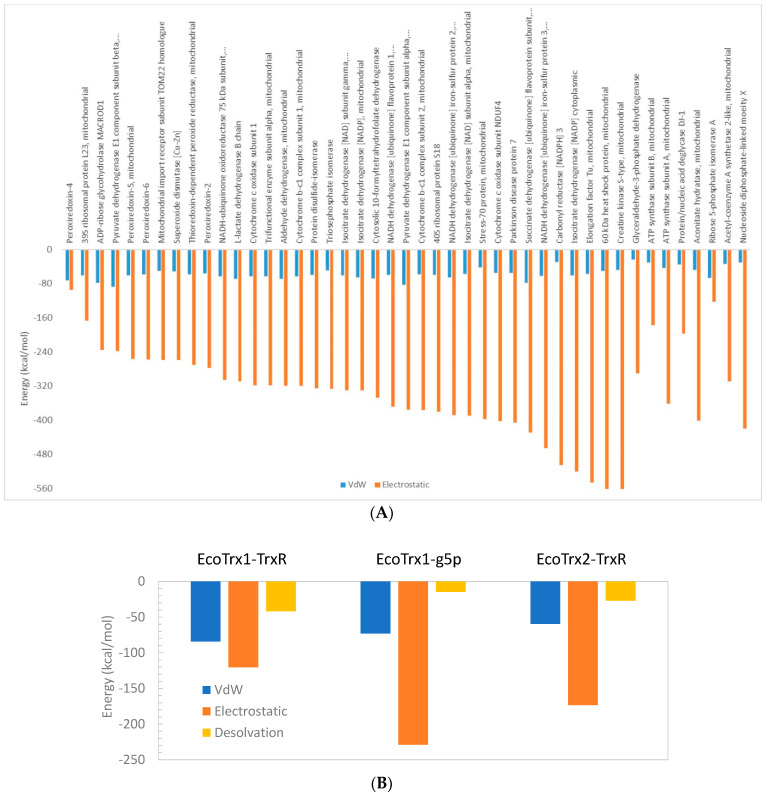
Van der Waals (VdW) and electrostatic (and desolvation in **B**) energy contributions to the binding energy for (**A**) each HsTrx2-protein complex and (**B**) for the complexes of EcoTrx1 and EcoTrx2 with TrxR and the gene 5 protein (g5p) as calculated by the HADDOCK web server.

Apart from its interaction with peroxiredoxin-4, the overall impression is that interactions of HsTrx2 with ligands are mainly based on electrostatic forces (Figure 1A) with van der Waals and desolvation energies (not shown in Figure 1A), contributing a fraction to the interaction. This was also the case for the interactions of EcoTrx1 with g5p and TrxR, and EcoTrx2 with TrxR (Figure 1B), where the contribution of van der Waals and desolvation energies to the interactions was rather low. The interactions of EcoTrx1 with other proteins have also been explained in terms of geometric and electrostatic complementarily [55].

### 3.2. The Contact Hot Spots of HsTrx2 for Molecular Recognition

**Figure 2:**
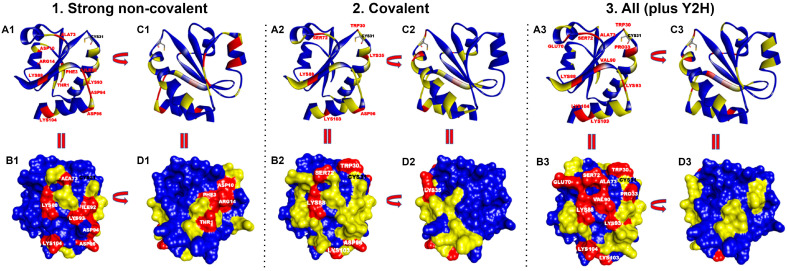
Thermal representation of the contact hot spots of HsTrx2 and their statistical distribution. Prioritized residues in the contact surfaces of HsTrx2 are shown for **1**. Strong non-covalent interactions, **2**. Covalent interactions, and **3**. All interactions together with their percentage (%) in contacts with substrates. Contact hot spot residues (>3%) are colored in red, median hot spot residues (1–2%) in yellow, and non-hot spot residues (~≤1%) in blue. (**A1**–**A3**) Ribbon representation (hot spots labeled in red; Cys^31^ residue’s side chain is depicted with sticks’ representation and labeled in black). (**B1**–**B3**) Surface representation (hot spots labeled in white; Cys^31^ is labeled in black. (**C1**–**C3**) Ribbon representation of (**A1**–**A3**) rotated 180° with direction from right to left. (**D1**–**D3**) Surface representation of (**B1**–**B3**) rotated 180° with direction from right to left.

**Figure 3:**
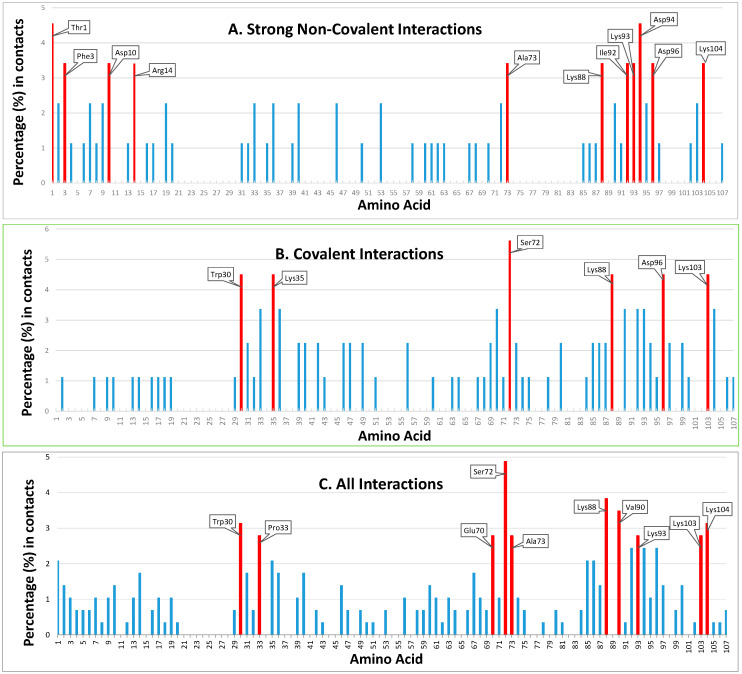
Positioning and ranking of the contact residues of HsTrx2 for its different kinds of interactions with substrates. The *Y* axis represents the statistical preference (as percentage) of each residue in the 38 examined complex interfaces to participate in the complexes. The *X* axis corresponds to amino acid numbering. Part (**A**) corresponds to strong non-covalent interactions, (**B**) to covalent interactions, and (**C**) to all detected interactions, including data from the yeast two-hybrid screens. All bars correspond to the statistical participation in contacts of each protein residue. Red bars indicate the percentages of the residues considered as contact hot spots.

The interfaces of the simulated docked complexes (PRISM) between HsTrx2 and its 38 identified ligands (Table 1) were analyzed (BIOVIA) to determine the residues of HsTrx2 involved in intermolecular interactions (results are shown in detail in Supplementary Data S2). In total, 77 out of 107 residues of HsTrx2 were involved in contacts in its complexes with other proteins. Almost all identified residues were accessible to solvent (Supplementary Data S2, Table S2). The threshold value for statistical hot spots for all interactions was slightly more than 1%, corresponding roughly to one contact hit. In such a manner, all statistical hot spot residues had at least two-fold more intermolecular interactions than the other interacting residues (Figure 2 and Figure 3). This threshold has left out many residues involved in detected interactions as insignificant contacts.

#### 3.2.1. Definition of “Contact Hot Spots” for HsTrx2

The interacting residues of HsTrx2 with protein ligands (Supplementary Data S3) were ranked (Figure 2 and Figure 3) according to their percentage of participation in the different kinds of interactions: covalent, strong non-covalent, and total (including yeast two-hybrid data). The threshold for all residues was one hit. “Contact hot spots” were defined as the residues that participated in most contacts for the different kinds of interactions. In practical terms, this was translated as 10–20% of all residues involved in the respective type of contacts.

#### 3.2.2. Contact Hot Spots for Strong Non-Covalent Interactions

Fifty-one residues were seemingly involved in strong non-covalent interactions (acidic elutions). Thr^1^ and Asp^94^ were highest in preferences followed by Phe^3^, Asp^10^, Arg^14^, Ala^73^, Lys^88^, Ile^92^, Lys^93^, Asp^96^, and Lys^104^ (Figure 2(A1–D1) and Figure 3A). In total, 11/61 of involved residues (22%) appeared most prominent in strong non-covalent interactions. The distribution of non-covalent hot spots can be divided into two groups. One with somewhat diffused dispersion proximal to the active site (Lys^93^, Asp^94^, and Asp^96^) and another distal to the active site where residues are locally concentrated (Thr^1^, Phe^3^, Asp^10^, and Arg^14^). There is an overlap of the proximal non-covalent hot spots with those of the active site (“covalent” Lys^103^ with “non-covalent” Lys^104^). However, non-covalent hot spots of the distal site seem completely isolated from the covalent hot spots. Hot spots Lys^88^ and Asp^96^ were common for covalent and strong non-covalent interactions.

#### 3.2.3. Contact Hot Spots for Covalent Interactions

Of the 107 residues of HsTrx2, 58 appeared involved in covalent interactions (elutions by DTT). Six (10%) (Trp^30^, Lys^35^, Ser^72^, Lys^88^, Asp^96^, and Lys^103^) appeared as the most prominent (Figure 2(A2–D2) and Figure 3B). Trp^30^ (3.9 Å from Cys^31^) and Lys^35^ (6.1 Å from Cys^31^) are in proximity to the active site and may offer charges and surfaces for electrostatic and van der Waal interactions, respectively, with ligands. The other four hot spot residues (Ser^72^ 13.7 Å, Lys^88^ 12.7 Å, Asp^96^ 24.8 Å, and Lys^103^ 25.5 Å) are rather distant from Cys^31^, which provides the thiolate for catalytic reactions. Covalent hot spots are concentrated at a small area around the attacking thiol and at an exposed part between the two greater, rather extended, surfaces of the molecule.

#### 3.2.4. Contact Hot Spots for All (Covalent, Strong Non-Covalent and Weak Non-Covalent) Interactions

**Figure 4:**
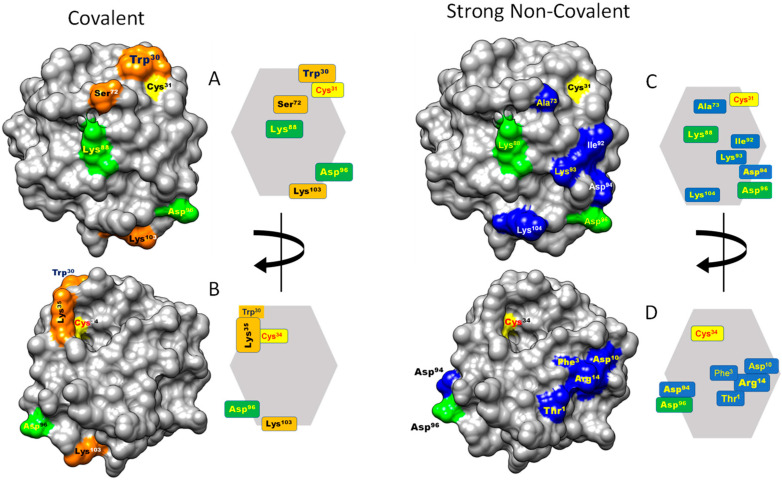
Contact hot spots on the surface of HsTrx2. The molecule is presented on the one extended side with the active site up and to the right (“front”, (**A**,**C**)) and after 180 degrees turn (“back”, (**B**,**D**)). Hot spots for covalent interactions are shown in orange, strong non-covalent in blue, and in green are the hot spots participating in covalent and strong non-covalent interactions. All hot spot residues are also shown placed on a simplified polygonal representation of HsTrx2. The structure of HsTrx2 is from PDB ID: 1w89, chain A with all water molecules removed [35].

Overall, 77 out of the 107 residues of HsTrx2 were likely involved in contacts with protein ligands. Ser^72^, Lys^88^, and Val^90^ appeared as the most prominent. Other statistically significant residues were Trp^30^, Pro^33^, Glu^70^, Ala^73^, Lys^93^, Lys^103,^ and Lys^104^ (Figure 2(A3–D3) and Figure 3C), amounting to 10 residues or 13% of all residues involved in contacts. Ser^72^ had the highest frequency of intermolecular interactions with ligands. Trp^30^ and Pro^33^ are next to the active site cysteines Cys^31^ and Cys^34^. The distribution of the contact hot spots of different types allowed for the representation of HsTrx2 as a flat, convex structure resembling a rounded polygonal brick (Figure 4). The one flat side with the catalytic Cys^31^ on the top right corner contains the highest concentration of contact hot spots. The back site with the resolving Cys^34^ on the top back left corner contains fewer contact hot spots that are mostly related to non-covalent interactions (the distal site). All contact hot spot residues can be imagined as protruding from the greater flat surface, while the catalytic cysteines are rather inwards the protein structure.

#### 3.2.5. Type and Organization of Contact Hot Spots

The outstanding statistical hot spots for HsTrx2 were 15 in total, composed of nine amino acid species (Thr, Phe, 3Asp, Arg, Trp, 5Lys, Ser, Ala, and Ile). Nowhere was attacking Cys^31^ highlighted as a statistical hot spot, an observation most likely reflecting its interaction with substrate disulfides as a secondary event following the docking of the surrounding hot spots. Asp and Lys residues are present as contacts three and five times, respectively. Due to their sequential proximity, Asp^94^ and Asp^96^ could be regarded as a negatively charged point for binding, while Lys^103^ and Lys^104^ a positive. Considering this, the resulting number of different hot spots becomes 13. If adjacent identical amino acids identified on substrates were considered one hot spot, the number of hot spots per ligand ranged from 3 to 13 with a median of 7 (“6.789”). With an average of 7 hot spots per ligand out of a selection of 13 possible hot spot positions, the total number of combinations is 1716. If the average number of hot spots per ligand is 3, the total combinations are 286. These numbers result from rather modest considerations of what a hot spot might be (Section 3.2). Therefore, even from a numerical point of view, HsTrx2 can accommodate many more ligands than the hitherto described [30]. Most of the contact hot spots of HsTrx2 were in packed clustering (adjacent residues 1, 3, 10, 14, then 30, 31, 35, then 72, 73, 88, then 92, 93, 94, 96 and 103, 104) a phenomenon observed in thermodynamic hot spots that may serve to exclude bulk water to strengthen protein-protein interactions [56]. Other contact hot spots (e.g., 94, 96, 103, and 104) were located on exposed or “edge” areas, which has also been observed before for thermodynamic hot spot residues [57].

### 3.3. Prediction of Possible Thermodynamic Hot Spots and Null Spots by SpotOn

**Figure 5:**
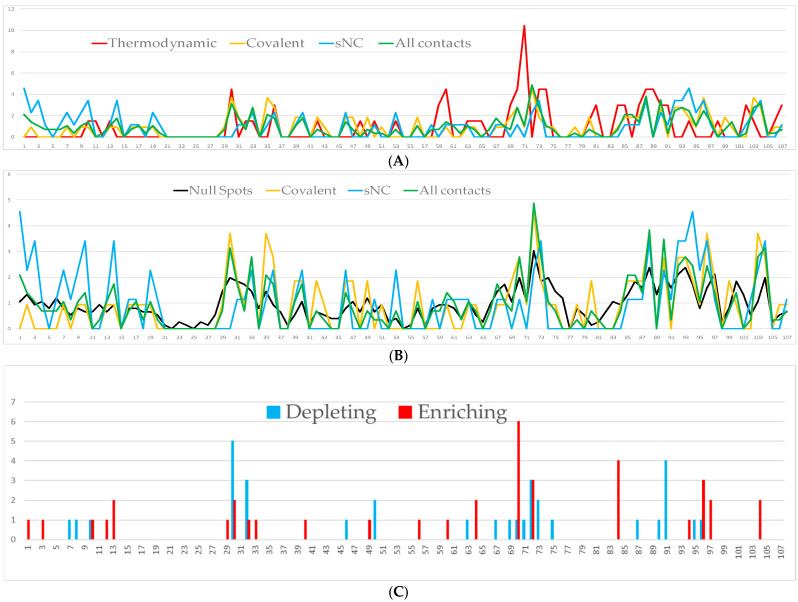
Predicted thermodynamic hot spots, null spots, and critical residues for the interaction of HsTrx2 with protein ligands. The *X* axis represents the numbering of all amino acids of HsTrx2 (1–107). (**A**) Comparison of the covalent, sNC, and all statistical contacts with thermodynamic hot spots by SpotOn. Covalent contacts are in orange line, sNC in blue, statistical in green, and thermodynamic hot spots in red. (**B**) Comparison of the covalent, sNC, and all statistical contacts with null spots by SpotOn. Covalent contacts are in orange line, sNC in blue, statistical in green, and null contacts in black. The *Y* axis in (**A**,**B**) represents the percentage frequency of each residue in the 38 examined complex interfaces. The absolute Y values thus represent the statistical preference of any amino acid for a contact residue or potential thermodynamic hot spot or null spot. (**C**) Predicted residues of HsTrx2 by PROT-ON whose replacements may result in significant changes in binding to protein ligands. Results are shown as total hits per amino acid (*Y* axis, a size of one corresponds to one interaction) for all 38 complexes analyzed. Red bars correspond to residues whose mutations may increase binding, and blue bars residues whose changes may decrease binding.

For the 38 complexes of HsTrx2 and its protein ligands, 36 putative thermodynamic hot spot residues were deduced by SpotOn [46] (Figure 5A,B). All these were previously (Section 3.2) identified as contact residues besides Phe^11^ Gln^48^, Leu^65^, Phe^89^, and Ala^98^, which were therefore excluded as false positives. Of the 31 remaining putative thermodynamic hot spots, 24 were also covalent contacts (in a total of 58, ratio 0.41) including the 30, 88, and 103 covalent contact hot spots (but not 35, 72, and 96), while 20 corresponded to sNC contacts (in a total of 51, ratio 0.39) including the 10, 73, 88, and 94 sNC contact hot spots (but not 1, 3, 14, 92, 93, 96, and 104). Despite the almost identical ratio, 8 SpotOn hot spots were unique for covalent interactions; 16 were common, while 4 appeared exclusively involved in sNC interactions (Supplementary Data S4). Although thermodynamic and contact residues overlapped throughout their range, their statistical distributions differed (Figure 5). A marked example is Val^71^, which appeared as the most popular putative thermodynamic hot spot of HsTrx2, at the same time having a quite low-ranking score as a contact hot spot (Figure 5A). The residue is next to Ser^72^, the most favorite contact hot spot. Overall, putative thermodynamic hot spots by SpotOn were identical or placed close to the contact hot spots.

Only 5 residues (out of 107) were not considered null spots from the SpotOn online tool. All 57 covalent and 50 sNC contacts were considered putative null spots, with 37 residues being common for both categories. The 16 proposed thermodynamic hot spots that were common in covalent and sNC interactions were all included in the 37 common null spot residues. The percentage distribution of the null spots for HsTrx2 (Figure 5B) generally follows the distribution preference of the total contact hits, especially if a 0.25% threshold is subtracted from the percentage distribution of the null spots. Such a restriction is logical, given the proposition of the program for a high number of residues, some of them not accessible to solvent (e.g., 21–28). In summary, a comparison of the topological distribution of putative null spots and thermodynamic hot spots by SpotOn overlapped partially or completely with contact residues and contact hot spots (Figure 5A).

#### 3.3.1. Critical Residues for the Interaction of HsTrx2 and Its Protein Substrates by PROT-ON

Extended in silico mutagenesis [48] of the interfaces of HsTrx2 and its 38 protein ligands (Supplementary Data S4) proposed residues whose replacements might result in increasing (enriching) or diminishing (depleting) binding to substrates (Figure 5C). Twenty depleting and twenty-one enriching residues were identified, four of which (48, 83, 89, 101) were excluded for not being recognized previously as contact residues by BIOVIA. The remaining, especially the “depleting”, can be considered critical for the interactions of HsTrx2 as they constitute conserved sites with lower ΔGs for ligand binding. They were allocated at it first 13 amino acids and parts 29–33, 40–50 (scattered), 60–75, and 83–104 (Figure 5C). For most interactions, mutations at 70 (Glu) resulted in stronger binding (enriching) while at 30 (Trp) at lower (depleting). There was significant overlap of these residues with the “all contacts” hot spots (Figure 5A,C). Compared to the contact hot spots for covalent interactions (30, 35, 72, 88, 96, and 103), the PROT-ON approach did not identify the three Lys (35, 88, and 103), while comparison with the contact hot spots for strong non-covalent interactions (1, 3, 10 14, 73, 88, 92, 93, 94, 96, and 104), 14, 88, 92, and 93 were not recognized. At the same time, neighboring residues of the former (e.g., 13, 87, 89, 93, and 94) were considered critical by PROT-ON. The outcome of PROT-ON for contact hot spots is reminiscent of that of the putative thermodynamic hot spots by SpotOn: critical residues by PROT-ON were identical or close to contact hot spots. Therefore, to consider all significant residues of HsTrx2 that may be involved in interactions with substrates, the concept of contact hot spots is not accurate enough. For a better inclusion of contact points, it would be helpful if neighboring residues to the contact hot spots were also considered. These, together with the contact hot spots, would present areas with a higher possibility of contacts with ligands. A network approach was used that centered on contact hot spots and their surrounding energy neighbors to define these areas.

### 3.4. Network Insights for HsTrx2

Network analysis perceives the structure of a protein as a continuous network with amino acids graphically shown as dots centering on their C^α^s. The interactions can be user-defined and are represented as links (or “edges”).

#### 3.4.1. Subnetwork Analysis

**Figure 6:**
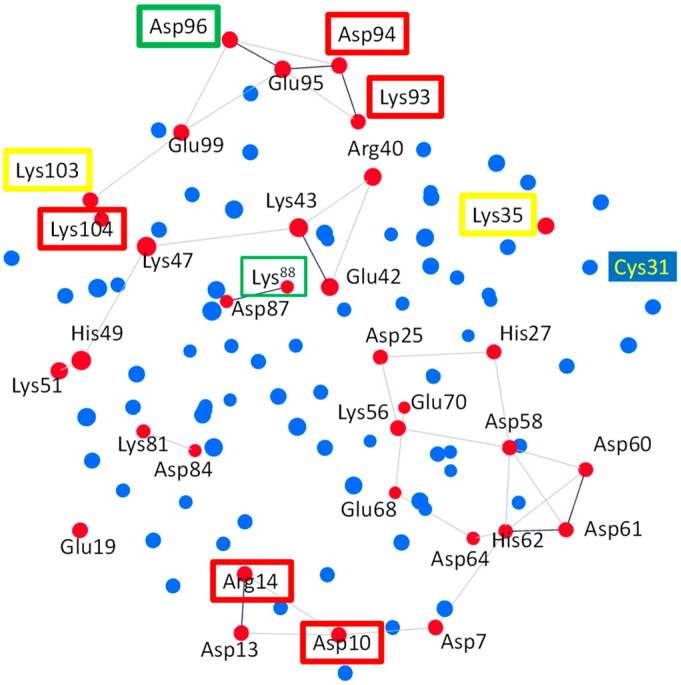
Three-dimensional C^α^ subnetworks of charged residues (all red circles) of HsTrx2. In red boxes are charged contact hot spot residues participating in strong non-covalent interactions. In yellow boxes are charged contact hot spots for covalent interactions, while in green are charged residue contact statistical hot spots participating in both covalent and strong non-covalent interactions. Subnetworks are presented as red circles connected with lines. All other residues are shown in blue. Subnetworks are presented as red circles connected with lines. Cys^31^ that is not participating in the subnetwork is also highlighted.

The residues of HsTrx2 may interact directly and indirectly with each other through their participation in subnetworks. To examine whether any of the identified contact hot spots participated in subnetworks, the charged, hydrophilic, and hydrophobic-related communication networks for the participating C^α^s were constructed for all residues of HsTrx2 (Figure 6 and Supplementary Data S5, Figures S1 and S2). The charged amino acids (Glu, Lys, Asp, Arg, and His) formed five subnetworks, which included residues of HsTrx2 involved in strong non-covalent interactions (Figure 6, Supplementary Data S5, Figure S1). The contact hot spot residue Lys^88^ in direct communication with Asp^87^ formed a two-membered subnetwork. Lys^81^ and Asp^84^ also formed a two-membered network. A six-membered subnetwork without any hot spot was formed by Lys^51^, His^49^, Lys^47^, Lys^43^, Arg^40^, and Glu^42^. The contact hot spot residues Asp^10^ and Arg^14^ that participate in strong non-covalent interactions communicated directly between themselves and also participated in a subnetwork with residues Asp^7^ and Asp^13^, themselves connected via His^62^, to Asp^64^, Asp^61^, Asp^60^, and Asp^58^ and through them to Glu^68^, Lys^56^, Glu^70^, Asp^25^, and His^27^ to form a 14-membered subnetwork. The fifth subnetwork was formed by hot spot residues Lys^93^, Asp^94^, Asp^96^, and Lys^104^ (responsible for strong non-covalent interaction), which may also communicate (directly and/or indirectly) with the residues Glu^95^, Glu^99^, and Lys^103^, resulting in a seven-membered subnetwork. The residues participating in networks may sense their interactions with ligands and perhaps modify the overall interaction pattern (i.e., salt bridges, electrostatic, and hydrogen bond interactions; Supplementary Data S2).

All hydrophobic residues (Ala, Ile, Leu, Val, Met, Phe, Pro, and Trp) formed extensive subnetworks (Supplementary Data S5, Figure S2A) where they were all interconnected, including contact hot spots Ala^73^ and Ile^92^. Gly residues did not participate in any subnetwork. Hydrophilic residues (Ser, Thr, Tyr, Cys, Gln, and Asn) did not form extensive subnetworks (Supplementary Data S5, Figure S2B): Thr^1^ is linked to Thr^2^, Asn^4^ to Gln^6^, Cys^34^ to Cys^31^ and itself to Gln^29^. There is a five-member subnetwork consisting of Thr^20^, Ser^18^, Asn^17^, Gln^12^, and Asn^82^. The top contact hot spot, Ser^72^, was not related to any subnetwork.

The interaction of binding surfaces and contacts within a charge-related network may be affected by the pH of the surrounding milieu [58]. The links involving His residues (His^27^-Asp^25^, His^49^-Lys^51^, and Asp^64^/Asp^7^-His^62^-Asp^60^/Asp^61^), for example, that were present in the crystal structure, could be lost due to protonation of the His resulting in subsequent loss of their positive charges (Supplementary Data S5, Figure S1). His residues can thus become switches in charge-related networks. In the 14-membered subnetwork of charged residues, for example (Figure 6), where hot spots Asp^10^ and Arg^14^ participate, deprotonation of His^62^ will “isolate” the hot spots from the protruding area (HsTrx2 residues 58–72) essential for the binding of NF-κB and Ref-1 on HsTrx1 [59]. However, Asp^10^ and Arg^14^ are alanines in HsTrx1, providing an example of differentiation of the contact hot spots resulting in altered contact areas among Trxs.

#### 3.4.2. Energy Network Neighbors

The approach of putative thermodynamic and null spots by SpotOn and critical residues for binding (PROT-ON) showed that the interactive residues of HsTrx2 with proposed thermodynamic significance were identical or adjacent to contact hot spots. To further examine the amino acids in the vicinity of the contact hot spots that might be involved directly or indirectly with ligand recognition and network participation, the neighboring C^α^s of all contact hot spots were identified by the energy network neighbor web tool https://academic.oup.com/nar/article/47/W1/W462/5491746?login=false, accessed on 1 May 2023 (Table 2). Additional satellite residues were identified as being possibly involved (at least indirectly) in the interaction of HsTrx2 with its ligands. In this way, all residues in the vicinity of contact hot spots were interrelated. Related putative contact areas emerged.

**Figure 7:**
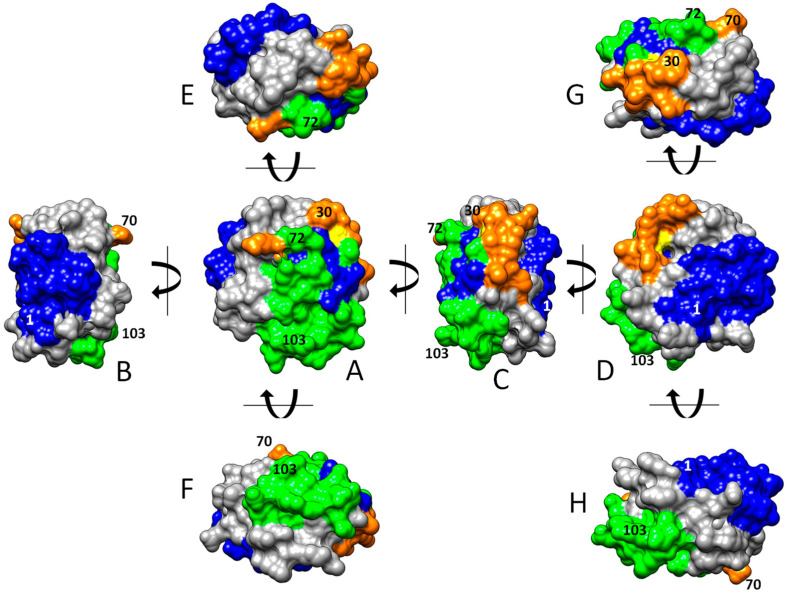
Surface areas of HsTrx2 involved in protein ligand recognition. In green are surfaces participating in covalent and strong non-covalent interactions, in orange are areas involved in covalent bonding, and in blue are regions involved in strong non-covalent interactions. The topology of interactions was calculated by combining (i) molecular docking that pinpointed contact hot spots and (ii) a network approach for the identification of the energy network neighbors of the contact hot spots. The structure of HsTrx2 is from PDB ID: 1w89, chain A, with all water molecules removed [35]. The orientations of shown molecules stem from (**A**) after vertical or horizontal turns of 90 degrees (**B**–**H**) in the direction of the arrows.

The three-dimensional graphical representation of HsTrx2, including the identified satellite residues of the contact hot spots, proposed three major interacting areas with protein ligands (Figure 7): (i) an exposed corner area around the active site with preference for covalent interactions (thiol-disulfide interchange) (Figure 7A,C,E), (ii) a greater area in the one flat one side of the molecule adjacent to the active site for both covalent and non-covalent interactions (Figure 7B,D), and (iii) a third area on the opposite flat side, more amenable to non-covalent interactions (Figure 7A,C,F). These outcomes should be considered tentative preferences. All three areas are not exclusive to covalent or non-covalent interactions as testified by the presence of “exclusive covalent” or “exclusive strong non-covalent” binding residues in the immediate proximity of the described “strong non-covalent” and “covalent” binding areas, respectively. Moreover, if the selection threshold for contact residues is lowered, some covalent contact points will be found in the non-covalent binding area (Figure 4) and more non-covalent contacts in the only covalent one.

#### 3.4.3. The Energy Network Neighbors of Contact Hot Spots Define Contact Areas That Include Most of the Putative Thermodynamic Hot Spots

**Figure 8:**
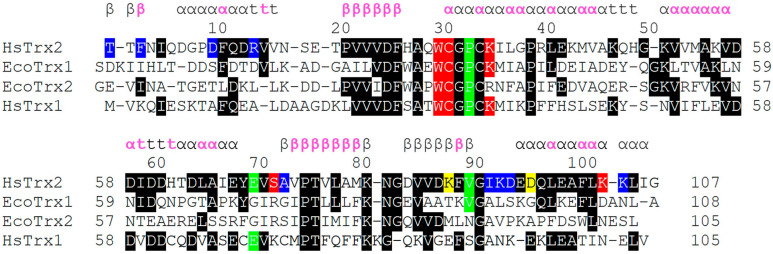
Alignment of HsTrx1 and EcoTrx1 and 2, relatively, to the three-dimensional structure of HsTrx2. At first, Trxs were aligned according to their primary sequence by Clustal Omega (https://www.ebi.ac.uk/Tools/msa/clustalo/, accessed on 1 May 2023). All residues identical to those of HsTrx2 were boxed on a black background. Then, each structure of the three Trxs was separately aligned to the three-dimensional structure of HsTrx2 (by the matchmaker function of UCSF Chimera). The resulting separate alignments of Trxs with HsTrx2 were put together. Residues in red, blue, and yellow backgrounds represent hot spot residues involved in covalent, strong non-covalent, and both covalent and strong non-covalent interactions, respectively. Residues in green background are additional hot spots identified by docking comparisons of all interacting partners (Figure 2(A3–D3) and Figure 3C). The secondary structure of HsTrx2 is shown above the alignment as derived from UniProt entry with ID Q99757. Pink letters correspond to residues non-accessible to solvent (as analyzed by BIOVIA Discovery Studio Visualizer-2021).

Comparison of the selected residues (contact hot spots plus energy neighbors) of the contact areas of HsTrx2 with those of putative thermodynamic importance as revealed by SpotOn (Section 3.3) and PROT-ON (Section 3.3.1) showed (Table 3) that most of the thermodynamic hot spots (35 out of 48) were included in the proposed interface areas (as discussed in Section 3.4.2). Some of the putative hot spots by SpotOn and PROT-ON that were not selected by the network approach (40, 42, 49, 69, 80, 81, 84, and 85) were adjacent to the network contact areas (Supplementary Data S4, Figure S1). Distinct putative thermodynamic hot spots, not in the proximity of any network-based surface areas, were residues 59, 60, 63, 64, and 67 (Supplementary Data S4, Figure S2). Their location is within the cleft/groove next to Trp^30^ preceding the active site. The residues for the groove are 59–76 for EcoTrx1 [15], hence 58–75 for HsTrx2 (Figure 8). The exclusion of the 13 thermodynamic hot spots from the network-derived contact areas reflects their limited participation as contact residues in the 38 examined complexes. If the number of protein ligands was greater (e.g., using less stringent washing protocols of the monothiol HsTrx2 affinity column than the high salt and acid treatment of the current protocol, Section 2.6 [30]) or if the threshold for the statistically significant contact residues (the herein “contact hot spots”) was lowered, more putative thermodynamic hot spots of the cleft region would have been included in the network contact areas.

### 3.5. Differentiation of Contact Surfaces among Trxs

The specificity of Trxs, Grxs, and other enzymes is dictated by their interacting surfaces, itself formed by the need for interaction with respective ligands. A comparison of the primary sequence of HsTrx2 with HsTrx1, and EcoTrx1, 2 revealed (Figure 8) that apart from the area 20–36 that includes their active sites, the overall homology is limited. This becomes even more profound when the proteins are aligned according to their three-dimensional structures (Figure 8).

#### 3.5.1. Protein Ligand Recognition by HsTrx2, Implications for Other Trxs

The proposed contact hot spots of HsTrx2 were compared to the respective residues of the three other Trxs (EcoTrx1, EcoTrx2, and HsTrx1). Residues identical to the contact hot spot of HsTrx2 were only those close to its active site, all involved in covalent interactions (Figure 8). This most likely reflects the conservation of the dithiol-disulfide exchange catalytic mechanism of Trxs. A significant lack or very limited homology was observed for the first 20 residues (area for mostly non-covalent interactions for HsTrx2). HsTrx2 was more homologous to HsTrx1 but not for the contact hot spots. Despite the lack of significant homology, a comparison of the three-dimensional structure of the four Trxs (Figure 8) showed that the Trx fold was almost identical in all four (Table 4).

#### 3.5.2. The Putative Interactive Surface Areas (Interfaces) of EcoTrx1, 2, and HsTrx1 in View of the Findings for HsTrx2

**Figure 9:**
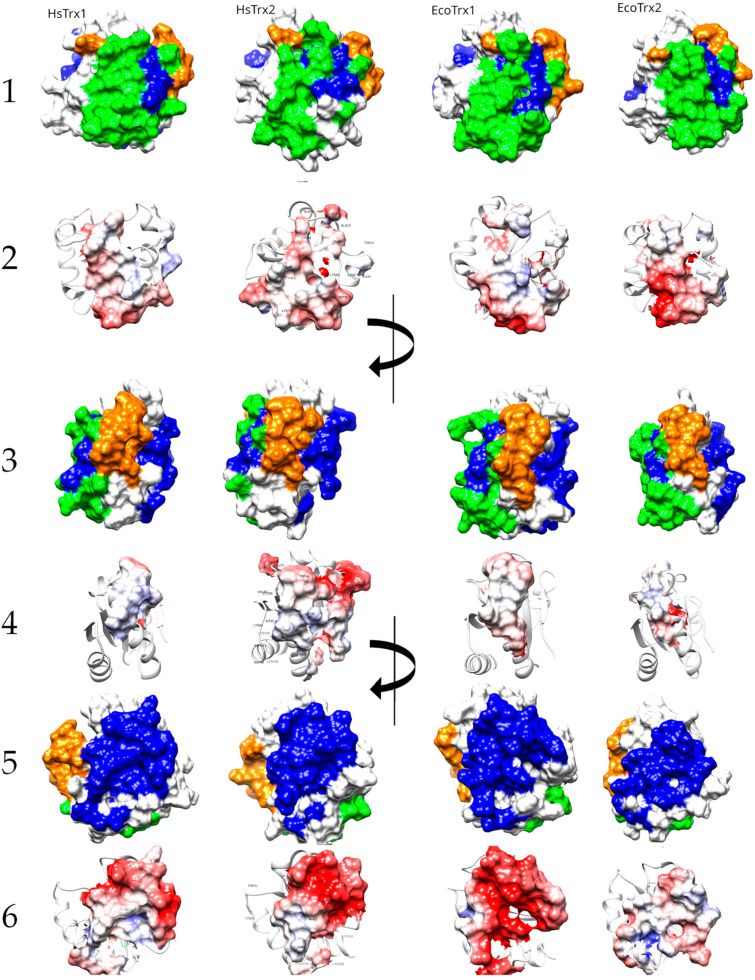
Proposed surfaces and relevant charge maps used by HsTrx1, HsTrx2, EcoTrx1, and EcoTrx2 for interactions with protein ligands. Lines **1**, **3**, and **5** correspond to molecular recognition by covalent bonds (orange), strong non-covalent interactions (blue), and both covalent and strong non-covalent interactions (green). Lines **2**, **4**, and **6** present the charge distribution in the aforementioned areas. Positively charged residues are in red, while negatively charged in blue.

The structural alignment of the four Trxs (Figure 8) was used to project the contact areas of HsTrx2 with protein ligands (statistical contact hot spots of HsTrx2 and accompanying energy network neighbors) on HsTrx1, EcoTrx1, and EcoTrx2 (Table 5, Figure 9).

As the major factor concerning the interactions of HsTrx2 and its ligands was electrostatic interactions (Figure 1), the charge distributions of all Trxs are also presented (Figure 9). The active sites of the four Trxs have increased identity in the participating residues (WCGPCK/R), resulting in no marked changes in the charges of that area (Figure 9). Apart from this short stretch, the four Trxs are quite distinct in their shapes and charges, around the proposed hot spots/contact areas (Figure 9(2,4)). There is no charge consensus in the binding areas concerning the area for covalent and strong non-covalent interactions among the four Trxs. The same applies to the area secluded for strong non-covalent interactions except for HsTrx2 and EcoTrx1, which appear sharing some charge pattern features (Figure 9(6)). Surface charges are expected to differentiate the binding behavior of Trxs so that the contact types and respective areas of HsTrx2 may not be reflected to the other Trxs (hence the quotation marks in Table 5).

### 3.6. Comparison of the Proposed Hot Spots and Surface Areas of the Four Trxs by Network Measures

In protein networks, the protein fold is perceived as a user-defined continuum of nodes and links in space. There are different measures describing the properties of the C^α^s of protein networks. Centrality measures (e.g., closeness centrality, betweenness centrality, average nearest neighbor degree, eccentricity, etc.) are used to detect interactions between amino acids in the protein network [49,60].

#### 3.6.1. Closeness Centrality

**Figure 10:**
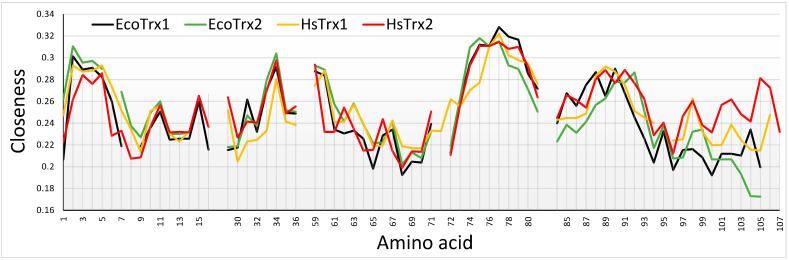
Comparison of closeness centralities among the four different Trxs. The contact hot spots for the interactions HsTrx2 were 1, 3, 10, 14, 30, 31, 33, 35, 70, 72, 73, 88, 90, 92, 93, 94, 96, 103, and 104. Sequence alignment is as shown in Figure 8, with the amino acid numbers of the *X* axis corresponding to those of HsTrx2.

Closeness centrality provides information on how central (more direct contacts) a particular node is by considering the total amount of direct links that it may have with other nodes, in this case, the C^α^s of different amino acid residues. The higher the closeness, the more direct contacts for a particular C^α^. Contact hot spots residues for covalent interactions Trp^30^, Cys^31^, Lys^35^, Ser^72^, Asp^96^, and Lys^103^ had all low to medium closeness values (Figure 10). The closeness of Lys^88^ was somewhat elevated. Strong non-covalent interaction contact hot spots Trp^30^, Glu^70^, Ser^72^, and Ala^73^ had lower closenesses, Pro^33^, Lys^93^, Lys^103^, and Lys^104^ average and Val^90^ relatively elevated (Figure 10). Overall, closeness centralities were lower for the contact hot spots of HsTrx2 and the corresponding contact areas of the other Trxs. The pattern of changes and absolute values of measured closeness centralities were similar for the four Trxs with buried residues giving higher values while exposed surface residues gave lower. The relatively lower overall closenesses for the HsTrx2 contact hot spots most likely reflect their external positioning. A noticeable difference between HsTrx2 and the other Trxs was the comparatively higher closenesses of C-terminal residues 100–107. This is probably due to the closer proximity of the alpha helix to the core of HsTrx2 relative to the other Trxs. Differences in closenesses were observed at the C-termini (after 94 for HsTrx2) for all Trxs. It is not known whether this is related anyhow to differentiation in substrate recognition. The 99–107 part of HsTrx2 is proposed to be involved in contacts with protein substrates (Table 5A). A marked observation concerning all Trxs is the very low closeness values of the Trp^30^ and Cys^31^ (attacking thiolate), a reflection of their exposed location for binding to substrates and participating in thiol-disulfide interchange, respectively. The positioning of a Trp or a Cys (Cys^31^) at the interface of ligand-substrate complexes is unusual for interactions of high energy [57]. Residues 74–80 with relatively higher closeness measures were not accessible to solvent (Supplementary Data S2) as they belong to the hydrophobic core of the molecule, providing thus many contacts hence their higher closenesses. The lowest closeness measures were observed for all Trxs in the cleft/groove area adjacent to the active site (residues 64–72 for HsTrx2) reflecting its extreme exposure to solvent.

#### 3.6.2. Betweenness Measures

Betweenness is a centrality measure that calculates how many times a node is on the shortest path between all network nodes. It does this by identifying all the shortest paths and then by counting how many times each node participates in one path. Therefore, betweenness shows which nodes are the most frequent ‘bridges’ in a network. All contact hot spots for covalent and strong non-covalent interactions of HsTrx2 had quite low betweenness values (Supplementary Data S5, Figure S3), which can again be explained in view of their exposed positioning. Betweenness values were not identical for the residues of different Trxs, but the variation was not related to any hot spot residues, contact, or thermodynamics (as seen later by energy fluctuations of the residues). As with the centrality (and following ANN degree measurements), betweenness values were low for the attacking thiolate of (Cys^31^ in HsTrx2) in all Trxs. This reflects the more exposed location of this residue, in contrast to the rather buried resolving Cys^34^, which had much higher values (Supplementary Data S5, Figure S4).

#### 3.6.3. Average Nearest Νeighbor Degree (ANN Degree) Plot

Low ANN degrees (average degree of the immediate neighbors to a node) were also observed for the attacking thiolates of all four Trxs (Supplementary Data S5, Figure S4), while the measures of hot spot residues did not differ significantly from those of other surface residues.

### 3.7. Validation of the Proposed Contact Hot Spots of HsTrx2 by the Contact Residues of EcoTrx1 and HsTrx1 Complexed with Protein Ligands

These were compared to contact residues of the other three Trxs complexed with protein ligands (the corresponding residues are shown in Supplementary Data S5, Table S1) to verify and validate the proposed statistically significant hot spot residues of HsTrx2. To this aim, the available crystal complexes of EcoTrx1 and HsTrx1 with known ligands were first analyzed by BIOVIA (Supplementary Data S6). The validated contact residues were compared to those from the herein-predicted contact areas for HsTrx2. As no available crystal complex of EcoTrx2 with any protein ligand is known, the contacts of HsTrx2 with ligands were perceived as the contacts of the molecule to itself in its homogeneous crystals.

#### 3.7.1. Comparison of the Contact Hot Spots of HsTrx2 and the Interacting Contact Residues of EcoTrx1 in Complexes with Protein Ligands

**Figure 11:**
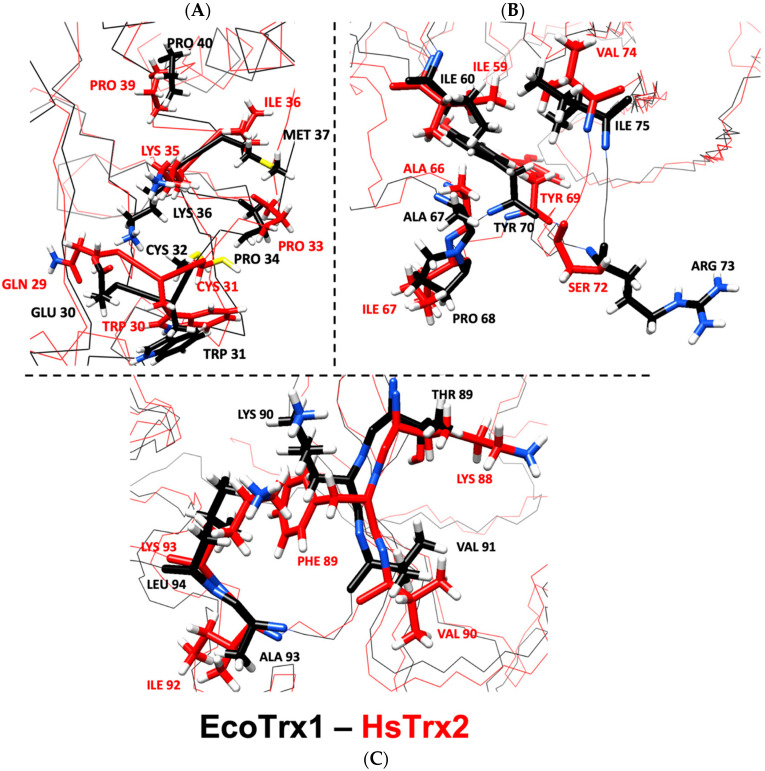
Overlay of minimized energy structures of EcoTrx1 (black) and HsTrx2 (red). (**A**) corresponds to the active site, (**B**) to the cleft/groove area, and (**C**) to the C-terminal contact area. All highlighted residues are contact points of EcoTrx1 to its ligands in crystal complexes with the respective residues of HsTrx2 shown in comparison. With black is the backbone for EcoTrx1, with red for HsTrx2.

According to the examined crystals of EcoTrx1 in complexes with four different ligands (Supplementary Data S6), 18 residues of EcoTrx1 were involved in molecular recognition (Table 6). Four of these contact residues were identical to the herein identified statistically significant contact hot spots of HsTrx2 (HsTrx2 labeled red, Table 6). Two more residues of EcoTrx1 (Cys^32^, Pro^40^) were identical to residues of HsTrx2 (labeled green in HsTrx2, Table 6) proposed to be involved in molecular recognition (Table 5A). A seventh residue of EcoTrx1, Ile^75^, was like Val^74^ of HsTrx2. Distinct differences were noted for Glu^30^ Met^37^, Pro^68^, Lys^90^, and Leu^94^ of EcoTrx1 (Gln^29^ Ile^36^, Ile^67^, Phe^89^, and Lys^93^ in HsTrx2). Four residues of EcoTrx1 did not belong to the proposed contact area as proposed by the findings for HsTrx2 (Table 6, gray shading). However, three out of the four were identical for both proteins, leaving “odd” Pro^68^ of EcoTrx1 in place of Ile^67^ of HsTrx2. Therefore, 14 out of 18 amino acids of EcoTrx1 involved in molecular recognition in crystal complexes belonged to the herein proposed (Table 5C) contact area. Out of the four residues not identified as belonging to contact areas, three were identical to residues of HsTrx2, and only Pro^68^ of EcoTrx1 differed (Ile^67^ in HsTrx2). Glu^30^, Met^37^, Pro^68^, Lys^90^, and Leu^94^ of EcoTrx1 (Ile^75^ is like Val^74^ of HsTrx2) were markedly distinct as contact points in the respective contact areas of HsTrx1 and 2 (Table 5A,C) and could possibly serve as points of differentiation in substrate recognition for the two Trxs. After superimposition of the minimized energy structures of the two proteins, the RMSD was 3.3 Å (Table 4). All verified contacts of EcoTrx1 were closely matched to those of HsTrx2 in the three-dimensional structures of the two proteins (Figure 11). In conclusion, (1) the side chains of contact hot spots of HsTrx2 appeared identical or like verified EcoTrx1 contacts; (2) the projected contact areas of HsTrx2 by the herein network approach are reflected on EcoTrx1 apart from parts 60–70, which contain nevertheless identical contact residues; and (3) the structural placement of verified contact residues of EcoTrx1 is almost identical to that of respective HsTrx2 residues. HsTrx2 and EcoTrx1 may thus employ respective areas for substrate recognition that may differentiate in distinct residues.

#### 3.7.2. Comparison of the Contact Hot Spots of HsTrx2 and the Corresponding Residues of HsTrx1 in Complexes with Protein Ligands

**Figure 12:**
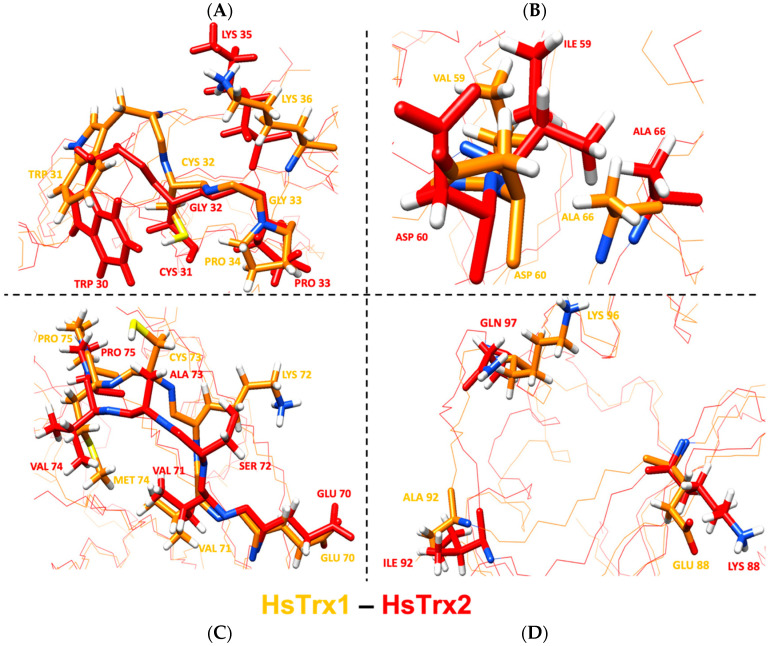
Overlay of the minimized energy structures of HsTrx1 (orange) and HsTrx2 (red). (**A**) corresponds to the active site, (**B**) to the cleft/groove area, and (**C**) to residues around all contact hot spot Ser^72^ of HsTrx2, and (**D**) to the Lys^88^, Ile ^92^, and Glu^97^ of HsTrx2. All highlighted residues are contact points of HsTrx1 and its ligands in crystal complexes with the respective residues of HsTrx2 shown in comparison. With orange is the backbone for EcoTrx1, with red for HsTrx2. Figures were made with ChimeraX, version 1.3.

Out of the 17 residues identified as contacts of HsTrx1 in complexes with ligands (Table 7), 10 residues were identical for both proteins, with 14 belonging to the proposed contact area for HsTrx1 (Table 5B). Two out of the three residues identified as not belonging to the proposed molecular recognition area of HsTrx1 (shaded gray in Table 7) were identical to those of HsTrx2, with the remaining Val^59^ of HsTrx1 being very similar to Ile^59^ of HsTrx2. The remaining six differing residues were Lys^72^, Cys^73^, Met^74^, Glu^88^, Ala^92^, Lys^96^, being Ser^72^, Ala^73^, Val^74^, Lys^88^, Ile^92^, Gln^97^ respectively in HsTrx2. With Lys^96^ of HsTrx1 being like Gln^97^ of HsTrx2, marked differences in the proposed contact areas concerned five residues (Val^59^ of the shaded gray area is excluded as being like Ile^59^ of HsTrx2). As was the case for EcoTrx1, the statistically significant contact hot spots of HsTrx2 matched closely in the three-dimensional structure of the corresponding residues of HsTrx1 (Figure 12). The overall conformations of the two proteins were quite similar as after superimposition of their minimized energy structures; the RMSD was 1.9 Å (Table 4). Could the observed differences provide the fine differentiation needed for substrate recognition? For example, Met^74^ of HsTrx1 (markedly different from the respective Val^74^ of HsTrx2) is suggested as participating in contacts with ASK1 [63]).

Conclusions: (1) as was the case for EcoTrx1, the contact hot spots of HsTrx2 appeared identical or like verified contacts of HsTrx1. (2) The projected contact areas of HsTrx2 by the herein network approach were reflected on HsTrx1 apart from residues 59–66, which contained similar/identical contact residues for both. (3) Verified contact residues of HsTrx1 are in the same position on the Trx fold as the respective residues in HsTrx2. Therefore, as was the case for EcoTrx1, HsTrx2, and HsTrx1, which apparently share respective areas for substrate recognition that may differentiate in distinct residues.

#### 3.7.3. Contact Residues of HsTrx2 in View of Its Crystal Structure

As there are no available crystal structures of HsTrx2 in complexes with ligands, the crystal contacts of HsTrx2 in its own crystals served as a basis for the proposition of possible contact hot spots. In each unit cell of a crystal there are three dimers in contact with each other and in contact with dimers from other cells [35]. The principal hydrophobic contacts involved Ile^36^, Val^86^, Ile^92^, and Leu^105^. Thr^1^, Arg^40^, His^49^, Asp^64^, Glu^68^, and Lys^104^ participated in hydrogen bonds. In a simulated contact with human peroxiredoxin 5, Trp^30^, Asp^58^, Asp^60^, Asp^61^, and Thr^63^ were the residues of HsTrx2 that participated in contacts [35]. If all these residues are considered as contact points of HsTrx2 with substrates, four of them (Thr^1^, Trp^30^, Ile^92^, and Lys^104^) are also characterized as contact hot spots in the current study, two (Ile^36^ and Leu^105^) are placed in the greater hot spot contact areas (Table 2) while the remaining nine (Arg^40^, His^49^, Asp^58^, Asp^60^, Asp^61^, Thr^63^, Asp^64^, Glu^68^, and Val^86^) are left out. It should be noted, however, that all last nine residues appeared to interact with ligands (Supplementary Data S2). They were not named contact hot spots simply because they were not considered statistically significant. Residues Asp^58^, Asp^60^, Asp^61^, Thr^63^, Asp^64^, and Glu^68^ belong to the characteristic cleft close to the active site [15].

#### 3.7.4. Participation of Cleft/Groove (Residues 58–75) of HsTrx2 in Substrate Recognition

A dent on the surface of Trxs next to the Trp preceding the active site sequence has been implicated in substrate recognition. This area is referred to as cleft [15] or groove [66]. It consists of residues 59–76 for EcoTrx1 [15] and respectively (Figure 8) 58–75 for HsTrx2 and HsTrx1, and 57–74 for the shortened (by 34 residues herein version, Section 2.1) EcoTrx2. Residues from that area interacted with substrates for both EcoTrx1 and HsTrx1 (Table 6 and Table 7), while the solution structures of HsTrx1 with trideca peptides from Ref-1 [59] and NF-κB [67], also showed interactions within the cleft area (HsTrx2 residues 58, 59, 61, 63, 66, 67, 71, 73, and 74 for NF-κB [67] and 59, 66, 71, 72, and 73 for Ref-1 [59]). In HsTrx2, the one “wall” of the cleft consists of 30, 60, 63, (64), and 67, and the other from 70 and 72 (73). In the bottom of the cleft are 59, 66, and 71, with a gap in the wall above 74 (PROT-ON, Supplementary Data S4, Figure S2). While residues 70–74 were recognized as contacts using the energy neighbor approach (Table 2), the other residues 60, 63, 64, 67, 66, and 69 (or 58–69) were not. According to BIOVIA (Section 2.6), the program used for the analysis of the HsTrx2 contacts in its complexes with ligands as determined by PRISM (Section 2.4), HsTrx2 can interact with its cleft area (58–69 sequence) with protein disulfide isomerase (Ile^59^, Asp^60^, Ala^66^, and Ile^67^), the alpha subunit of component E1 of pyruvate dehydrogenase (Asp^60^, Asp^61^, His^62^, Thr^63^, and Ile^67^), the alpha subunit of mitochondrial ATP synthase (Thr^63^, Ile^67^, and Glu^68^), and peroxiredoxins 2 (Ile^59^ and Ala^66^) and 5 (Asp^61^, Asp^64^, and Ile^67^) (Supplementary Data S2). These residues were not highlighted because none of them was considered statistically significant to constitute a contact hot spot (Figure 3).

The prediction of putative thermodynamic hot spots for residues 58–69 of HsTrx2 was somewhat uncertain. Residues 59, 60, 63, 64, and 69 were considered potential thermodynamic hot spots by SpotOn (Supplementary Data S4). We focused on Ile^59^ and Asp^60^ of HsTrx2 as their equivalents are involved in the binding of EcoTrx1 (Ile^60^ instead of Ile^59^ in HsTrx2) and HsTrx1 (Val^59^, Asp^60^ in HsTrx2) to protein ligands (Table 6 and Table 7), while Asp^60^ is also a contact in the self-crystals of HsTrx2 (Section 3.7.3). When residues 59 and 60 of HsTrx2 were mutated in silico to alanines, the resulting new binding energies were below the 2 kcal/mol threshold that defines thermodynamic hot spots (Table 8) [57]. Meanwhile, the SpotOn server recognized as thermodynamic hot spots Ile^59^ and Asp^60^ but only in the complex of HsTrx2 to protein disulfide isomerase and peroxiredoxin-3 (Supplementary Data S4, UniProt IDs P07237 and P30048, the complex with peroxiredoxin-3, however, was not recognized by BIOVIA and was therefore ignored). In the case of protein disulfide isomerase, the ΔΔGs of the Ala mutations of Ile^59^ and Asp^60^ with SpotOn and PROT-ON, were below the 2 kcal/mol threshold spots (Table 8) to constitute thermodynamic hot spots for strong binding. Therefore, it could be that residues 59 and 60 of HsTrx2 are contacts with substrates but not thermodynamic hot spots, at least for the examined complexes.

The other web tool seeking for putative hot spots, PROT-ON, considered 63, 67, and 69 in the area 58–69 of HsTrx2 as significant thermodynamic (“depleting”) hot spots (Figure 4C). The ligands of the HsTrx2 complexes for which the predictions were performed concerned PDBs P30044 (peroxiredoxin-5) and O75489 (NADH dehydrogenase [ubiquinone] iron-sulfur protein 3, mitochondrial) for residue 63, O75828 (Carbonyl reductase [NADPH] 3) for 67 and A8MXV4 (Nucleoside diphosphate-linked moiety X motif 19) for residue 69 (Supplementary Data S4). The analyses by PRISM (Section 2.4) and BIOVIA (Section 2.6) however, on whom all this work is based, did not detect any interactions for 63. Sixty-three is also absent as a contact residue in the verified contacts (Table 6 and Table 7). PRISM and PROT-ON coincided on 67 (Ile^67^ of HsTrx2 with Lys^180^ of carbonyl reductase [NADPH] 3) and Tyr^69^ in its interaction with nucleoside diphosphate-linked moiety X motif 19, while the residue could also interact (in PRISM and not PROT-ON) with Ala^237^ from O-acetyl-ADP-ribose deacetylase MACROD1 (UniProt KB: Q9BQ69). Thus, one hot spot (63) was questionable, while the program proposed residues 67 and 69, which were also contacts in the analysis by BIOVIA (67 and 69 were also implied contacts for HsTrx2 in view of the results for EcoTrx1, Table 6). It could be that the two residues may contribute significantly to binding to ligands but not as much as to lead to the formation of stable complexes that will not dissociate by the stringent washing protocols of the affinity columns [30] before analysis by mass spectrometry. Better answers for the critical residues of HsTrx2 for binding to substrates and their energy contributions may come from wet lab experiments regarding the effects of point mutants of HsTrx2 on the thermodynamic stability of specific complexes.

## 4. Discussion

Protein-protein interactions are inextricably linked to life with established trends concerning their nature and dynamics. Interfaces in molecular contacts usually appear extended ([57] and references therein). Interactions between proteins may vary in strength and duration, both reflected in the ΔGs and *K_D_*s of the specific pairs. A large-scale analysis regarding the interface structures in crystal complexes available in the Protein Data Bank revealed that interfaces are usually flat [68]. However, even extended, and flat interfaces utilize discontinuous interacting regions and small pockets as binding sites [68]. The free energy of the binding may not be evenly distributed among the participating amino acids [69]. Localized hot spots in the dimer interface, contribute most of the binding energy for the interaction [57], with the growth hormone and its receptor providing a characteristic example [70,71]. While more stable complexes employ extensive hydrophobic areas [72], enzymes [73], or even antibodies (e.g., the complex between antibody D1.3 and the anti-D1.3 antibody E5.2 [74]), contact areas may be dependent on many weaker transient contacts, without necessarily the participation of significant thermodynamic hot spot residues. This work used an in silico approach to identify the residues and resulting contact areas of HsTrx2 involved in its interactions with protein ligands. The outcome is a putative insight into the placing and competition of different ligands for a limited number of binding areas placed on the same protein.

### 4.1. Properties of the Contact Hot Spots and Contact Areas of HsTrx2

To analyze the interaction of HsTrx2 with its protein ligands, we used in silico approaches for its 53-membered experimentally verified interactome [30]. Thirty-eight interactions with HsTrx2 were finally selected by the docking algorithm of PRISM that gave the respective three-dimensional complexes. The energy and types of interactions suggested that the specificity of HsTrx2-ligand interactions was determined by complementary surface geometry and opposing charges, a general phenomenon [57], with EcoTrx1 being no exception [55]. Next, the HsTrx2-ligand interfaces were analyzed to identify the amino acid contacts involved in different types of interactions examined (e.g., covalent, strong non-covalent, and total). Residues participating with higher frequencies in contacts with ligands were herein defined as “statistically significant contact residues” or simply “contact hot spots” (Figure 2 and Figure 3). According to the elution protocol of the affinity ligands, the contact hot spots were further divided as covalent or strong non-covalent. The selection of contact hot spots was dependent on the number of examined ligands, the interacting residues, and the chosen threshold for statistical significance. The proposed contact hot spots for HsTrx2 were 15, belonging to nine amino acid species (Thr, Phe, 3Asp, Arg, Trp, 5Lys, Ser, Ala, and Ile). Although the first-ever interaction of Trxs with substrates was via thiol-disulfide interchange mediated by the active site cysteines [1], the attacking Cys^31^ of HsTrx2 was not identified herein as a contact hot spot. Apparently, its participation in the reaction is a secondary event following the docking via the covalent contact hot spots. Nine out of the fifteen different contact hot spots of HsTrx2 were charged. The participation of ionic residues in the interaction area is consistent with the general tendency for the contribution of electrostatic interactions in the interfaces of transient protein complexes [75,76], such as the ones that an enzyme like HsTrx2 (and the other Trxs) is likely to participate.

The proposed contact hot spots were not selected by any thermodynamic criteria. Comparison of the distribution of contact hot spots to those of potential thermodynamic hot spots and null spots, however, showed that they often identified or were neighbors. This implied that contact hot spots have thermodynamic relevance, especially if neighboring residues are considered. A network approach [50] was applied to identify the energy neighbors of the contact hot spots to include the latter as possible contact sites. Contact hot spots, together with their close energy neighbors, defined three major interacting (or contact) areas for protein ligands: a corner-like exposed area including the active site involved exclusively in covalent interactions, a neighboring extended area for covalent and strong non-covalent interactions, and, finally, a third region relatively isolated from the other two, apparently involved exclusively in strong non-covalent interactions. HsTrx2 may thus share motifs of molecular recognition for covalent and strong non-covalent interactions but may also differentiate between the two types of interactions, which is convenient for a small molecule with limited total surface area. The different positioning of the three proposed binding areas on the same molecule is a logical correlation of allocating a specific interaction to a defined place. When a protein contains an area that is repeatedly reused for binding by many proteins, it can be characterized as a multipartner protein that may link several cellular processes [77]. This is the case for HsTrx2 [30], with the herein-described distribution of its binding areas confirming the multiplicity of its targets/functions. EcoTrx1 is also a protein with broad substrate specificity (as demonstrated by its multiple functions and substrates identified by the monothiol trapping approach [28,29]), while the same applies to many plant Trxs ([27] and references therein).

Trxs of different species maintain general domain architecture [5], and when in complexes with protein ligands, the structures of the ligands are more likely to change [15]. That could entail the possibility of common recognition motifs between Trxs and their protein partners. As the 15 detected contact hot spots of HsTrx2 retained a degree of variability among the four Trxs examined (Figure 8), it would be tempting to consider their placement in space, combined with some other elements of Trx structure (such as the active site with the preceding Trp), as a starting grid for molecular recognition. This approach has not been possible for the covalent interactions of Trxs [15]. In any case, it would be too risky, though, to use a hypothetical grid to predict ligands by mere computational methods, as applications based entirely on in silico docking are prone to errors [15,78]. Wet lab experiments remain the best way to identify and verify protein substrates/ligands [15].

### 4.2. The Topology of the Contact Hot Spots and Respective Contact Areas of HsTrx2 Is Reflected in the Other TRXS

HsTrx2 was used as a basis for alignment with the other Trxs (EcoTrx1, EcoTrx2, and HsTrx1), and its contact hot spots and relevant contact areas were projected to their respective residues/areas. The contact hot spots of HsTrx2 and the derived contact areas were next validated by comparing them to defined contact residues in selected complexes of EcoTrx1 and HsTrx1 with other proteins. These complexes have been characterized previously by crystallography (e.g., EcoTrx1 and HsTrx1, Table 6 and Table 7) or NMR (Ref-1 and NF-κB binding peptides to HsTrx1 [59,67]). For HsTrx2, its own crystal and a proposed interaction with peroxiredoxin 5 were used to define its contacts with protein ligands [35]. Apart from regions 58–69, all validated contact points of EcoTrx1 and HsTrx1 were within the predicted contact areas as extrapolated from HsTrx2. Moreover, there was significant identity in the contact hot spots of HsTrx2 with corresponding residues-contacts of the other Trxs. These observations validate the herein-described contact areas of HsTrx2 but are also in favor of the extrapolated areas for at least HsTrx1 and EcoTrx1. Changes in the side chains of the proposed contact areas of the four Trxs allowed for the differentiation of displayed surface charges on a conserved fold (Figure 9). This is a general rule for functionally diverse enzyme superfamilies representing the one-third of the known ones [79].

A part (58–69) of the cleft/groove region (58–75 for HsTrx2 and HsTrx1, 59–76 for EcoTrx1 [15]) that was not detected by the current approach as an interface contained validated contact residues for EcoTrx1, HsTrx1, and HsTrx2. This region may also participate in interactions of HsTrx2 with proteins (in 5 out of the 38 examined complexes). The prioritization and determination of contact areas exclusively on statistically contact hot spots however, left out residues with statistically fewer contacts. Given the high stringency (extensive salt washes followed by acid and DTT elutions) used to detect the interactions of HsTrx2 with its protein ligands, it could be that many of the ligands interacting with the 58–69 area (or other areas appearing as no contact areas) were washed away before the proteomic analysis occurred. Ligands of weaker binding characteristics could be discovered in future experiments by washing the affinity columns containing immobilized monothiol HsTrx2 with increasing ionic strengths. The weak interactions of HsTrx2 (labile interactome) could be numerous, concern vital cellular functions, and be subject to subtle changes in the cellular milieu.

### 4.3. Network Measures Show Similarities and Fine Differences among the Four Trxs

To examine the possibility of common general characteristics and differences among the contact hot spot residues of HsTrx2 and the corresponding residues on the other three Trxs, selected node centrality measures (closeness, betweenness centralities, and ANN degree) were estimated and compared. The respective surface residues among the four molecules were almost identical in terms of their network properties. This showed the similarity of the organization of different proteins sharing a common fold while being constituted by different amino acid sequences. The relatively low centrality degrees of the contact hot spots are explainable by their exposed positioning, something distinct for the active sites and preceding Trp (Trp^30^ for HsTrx2) of all four Trxs. This contrasts with the active and allosteric sites of other enzymes located more within the protein structure, where closenesses are generally higher [80]. Another peculiarity of the active site of Trxs is the somewhat exposed Cys contributing to the attacking thiolates, which are normally not preferred for contact interfaces [57]. The 58–72 cleft for HsTrx2 (involved in HsTrx1, EcoTrx1, 2 in binding to substrates 3.6.4 [66]) had the lowest closeness values due to its extremely exposed nature.

Subnetwork analysis revealed subtle adjustments in the surfaces of the Trxs for their interactions with substrates. An example is the differentiation of the subnetworks of the polar residues of HsTrx1 and HsTrx2. In HsTrx2, the polar residues can be connected via His^62^ to Asp^10^ and Arg^14^, which are contact hot spots for covalent interactions (Figure 6). In HsTrx1, Asp^10^ and Arg^14^ are replaced by Ala, the replacements changing the subnetwork on HsTrx1 and providing an example of fine-tuning for the differentiation of ligand contacts between two similar proteins.

### 4.4. Individual Hot Spots as Possible Pharmacological Targets

Inhibitors of the Trx system usually target the much larger TrxRs, which have a more complicated inner electron flow starting from coenzyme NADPH to bound FAD and then to their active sites that will finally reduce Trxs [81]. Moreover, sizes and active sites may differ among TrxRs of different organisms, making them amenable targets for selective inhibition without affecting the host [82]. Synthetic alkylating inhibitors (alkyl-2-imidazolyl disulfides compounds) for HsTrx1 that can target its active site thiols have been used in treatments against different types of cancer ([83] and references therein). These compounds most likely interact also with the highly active selenates of the selenocysteines of mammalian TrxRs. Would it be possible to selectively target individual Trxs of the Trx system without affecting the activity of other thiol/selenothiol-involving pathways or TrxRs? The identification of hot spots and their neighboring residues for the interaction of HsTrx2 with its ligands could, in principle, render Trxs selective targets for small inhibitors [76,84]. Lys^88^ and especially Ser^72^ (with energy neighbors Glu^70^, Val^71^, and Ala^73^, the latter also a contact hot spot for HsTrx2) stand out as the most prevalent contact hot spots (perhaps counterparts of the low ΔG “super-hot spots” [76]). In the four Trxs of this work, the respective positions were occupied by different residues (Figure 8). Such details may provide fine points for pharmacological interventions by peptide-based inhibitors with an irreversible mode of action [85] that will be able to single out and neutralize specifically HsTrx2 and, therefore, the mitochondrial Trx system without disturbing internal TrxR1, 2, HsTrx1, and the bacterial TrxRs and Trxs of the microbiome of the gut. As far as we know, this approach has not been attempted yet. In general, Trxs have not been studied extensively with respect to their topology of hot spots related to their interactions with ligands. In comparison, great efforts have been placed to clarify and explain fine differences in their similar catalytic mechanism differentiated by the pKas of their redox-active cysteines and the redox potentials of their active sites (e.g., [86]). Future studies would show whether inhibitory compounds to the contact hot spots of Trxs could attain any kind of selectivity and therapeutic potential.

## 5. Conclusions

We provide suggestive evidence for the identity of the statistically preferred contact residues (contact hot spots) and relevant interfaces of HsTrx2 with its protein ligands. HsTrx2 has apparently three general areas for interactions: one specialized in covalent interactions close to the active site, one distant to the active site for strong non-covalent interactions, and a third area bordering the active site where both kinds of interactions may take place. Comparison of the contact residues of EcoTrx1 and HsTrx1 complexed with protein ligands to the contact hot spots of HsTrx2 revealed identities and ranging similarities in the side chains of the amino acids involved in contacts. Moreover, all residues were placed on an almost identical backbone structure. Substrate recognition in Trxs can thus be largely viewed as being dictated by small changes in the location of side chains of hot spot residues placed in dedicated contact areas of the conserved active site and structure of the Trx fold.

## Figures and Tables

**Table 1:** Apparent equilibrium dissociation constants (*K_D_*s) and Gibbs free energy changes (ΔGs) for the interaction of monothiol HsTrx2 and 38 selected ligands. ΔGs and *K_D_*s were calculated by the PRODIGY web server. The types of interactions between HsTrx2 and ligands were covalent (C), strong non-covalent (NC), both (NC, C), co-fractionation, or not mentioned (NM). AF corresponds to Alpha Fold. The numbering (#) of the protein ligands is according to the obtained docking score.

#	UniProt ID	Protein Species	PDB ID or AF	PRISM		PRODIGY		Type of Interaction ^c^
				Docking Score ^a^ (kcal)	Interface ID	ΔG (kcal/mol)	*K_D_* (nM) ^b^	
1	P22695	Cytochrome b-c1 complex subunit 2, mitochondrial	5xte (chain J)	−50.22	1h9rAB	−10.3	2800	NC
2	P30048	Peroxiredoxin-3	5jcg (chain A)	−49.26	2vocAB	−7.9	1500	C
3	P49821	NADH dehydrogenase [ubiquinone] flavoprotein 1, mitochondrial	5xtb (chain A)	−48.13	2i7dAB	−7.7	2200	NC
4	P07237	Protein disulfide isomerase	6i7s (chain A)	−47.77	1uvzEF	−9.1	190	NC
5	P07195	L-lactate dehydrogenase B chain	7dbj (chain A)	−37.33	2ldxCD	−9.3	160	NC
6	O00483	Cytochrome c oxidase subunit NDUFA4	5z62 (chain N)	−37.25	3k6cBC	−8.5	590	NC
7	Q13162	Peroxiredoxin-4	3tjj (chain A)	−34.22	1u2eAC	−10.2	32	NC, C
8	O75489	NADH dehydrogenase [ubiquinone] iron-sulfur protein 3, mitochondrial	5xtb (chain O)	−32.7	3s55BC	−12.1	1.3	NC
9	P00441	Superoxide dismutase 1, soluble	2c9v (chain A)	−26.54	1f9mAB	−8.2	1000	Co-fractionation/ BioGRID
10	P40939	Trifunctional enzyme subunit alpha, mitochondrial	5zqz (chain A)	−26.11	1oypAB	−9.2	160	NC
11	P62269	40S ribosomal protein S18	6zxg (chain U)	−23.24	2a2oCD	−8	1300	NC
12	P32119	Peroxiredoxin-2	7kiz (chain A)	−22.05	1u2eAC	−13.1	0.23	NC, C
13	Q9NUB1	Acetyl-coenzyme A synthetase 2-like, mitochondrial	AF	−21.81	1c2yGH	−6.3	24,000	C
14	P06576	ATP synthase subunit beta, mitochondrial	AF	−17.99	1nswCD	−6.7	11,000	NC, C
15	P00395	Cytochrome c oxidasesubunit 1	5z62 (chain A)	−17.92	1utrAB	−9.9	58	NC
16	P50213	Isocitrate dehydrogenase [NAD] subunit alpha, mitochondrial	7ce3 (chain A)	−17.46	1f9mAB	−5.8	58,000	C
17	P49247	Ribose 5-phosphateisomerase A	AF	−15.87	3md9AB	−9.3	150	NM BioGRID
18	P08559	Pyruvate dehydrogenase E1 component subunit alpha, somatic form, mitochondrial	2ozl (chain A)	−13.94	3m8eAB	−11.4	4.5	NC
19	O75306	NADH dehydrogenase [ubiquinone] iron-sulfur protein 2, mitochondrial	5xtd (chain P)	−13.5	2r78CD	−9.8	61	NC
20	P30041	Peroxiredoxin-6	5b6m (chain A)	−12.42	1u2eAC	−9.1	210	C
21	P11177	Pyruvate dehydrogenase E1 component subunit beta, mitochondrial	2ozl (chain B)	−12.3	1mn8AB	−8.4	720	C
22	P30044	Peroxiredoxin-5	3mng (chain A)	−11.72	2bkkCD	−6.9	9100	NC, C
23	P38646	Stress-70 protein, mitochondrial	AF	−11.13	3p01AC	−7.4	3500	C
24	P25705	ATP synthase subunit alpha, mitochondrial	AF	−8.98	1f9mAB	−10.7	1500	NC, C
25	Q99798	Aconitate hydratase, mitochondrial	AF	−8.58	1yllAB	−10	46	C
26	Q9BQ69	O-acetyl-ADP-ribose deacetylase MACROD1	2x47 (chain A)	−8.33	3qqmAB	−11.7	2.4	C
27	A8MXV4	Nucleoside diphosphate-linked moiety X motif 19	AF	−7.98	3dxbCG	−10.6	18	C
28	P31930	Cytochrome b-c1 complex subunit 1, mitochondrial	5xte (chain K)	−6.69	2j0fAC	−7.9	1500	C
29	O75891	Cytosolic 10-formyltetra-hydrofolate dehydrogenase	AF	−5.45	3zymAB	−9.8	60	C
30	O75828	Carbonyl reductase [NADPH] 3	2HRB (chain A)	−5.45	1jmuFG	−8.4	640	C
31	P49411	Elongation factor Tu, mitochondrial, EF-Tu	AF	−3.92	2b6mAB	−6.3	25,000	C
32	Q99497	Protein/nucleic acid deglycase DJ-1	1p5f (chain A)	−3.65	1barAB	−9.8	62	C
33	P17540	Creatine kinase S-type, mitochondrial	4z9m (chain A)	−2.68	1tilEF	−7	7900	C
34	P04406	Glyceraldehyde-3-phosphate dehydrogenase	6ynd (chain A)	−2.39	3kbqAB	−10.2	32	NC, C
35	P48735	Isocitrate dehydrogenase [NADP], mitochondrial	5i96 (chain A)	−1.84	2ywbCD	−9.4	130	NC
36	Q16540	Mitochondrial ribosomal protein L23	7of0 (chain EA)	−1.41	2ux8BC	−9.9	50	Co-fractionation/ BioGRID
37	P60174	Triosephosphate isomerase	6upf (chain A)	−1.04	1zvnAB	−9.7	75	NC, C
38	Q86WU2	Probable D-lactate dehydrogenase, mitochondrial	AF	−2.67	2j0fAC	−8.4	710	C

^a^ Fiberdock scoring, ^b^ at 25.0 ℃, ^c^ Abbreviations used: C, covalent interaction confirmed by DTT; NC, non-covalent interaction (confirmed either by acid or the yeast two-hybrid system); NM, interactions mined from the co-fractionation/BioGRID repository.

**Table 2:** Energy network Neighbors for all contact hot spot residues of HsTrx2. All residues are presented as numbers. “C” stands for covalent and “sNC” for strong non-covalent interactions. The presented *Cys^31^ is not considered a “contact hot spot” (Section 3.2.5).

			Contact Hot Spots Plus Neighbors				
Contact Hot Spot	Interaction Type	Energy Neighbors	All sNC	All C	Common C and sNC	Only C	Onlys NC
Thr^1^	sNC	2, 3, 46, 50, 53, 54	1–18 33 37 46 50 53–56 71–79 87–107	28–39 70–73 77–79 87–90 94–107	33 37 71–73 77–79 87–90 94–107	28–32 34–36 38 39 70	1–18 46 50 53–56 74–76 91–93
Phe^3^	sNC	1, 2, 4, 5, 54–56					
Asp^10^	sNC	5–9, 11–14					
Arg^14^	sNC	10–13, 15–18					
Trp^30^	C	28, 29, 31, 32					
*Cys^31^	Active site	28–30, 32–34, 74					
Lys^35^	C	32–34, 36–39					
Ser^72^	C	70, 71, 73					
Ala^73^	sNC	71, 72, 74–76					
Lys^88^	C/sNC	77–79, 87, 89, 90					
Ile^92^	sNC	33, 37, 91, 93, 94					
Lys^93^	sNC	37, 91, 92, 94, 95, 97, 98					
Asp^94^	sNC	92, 93, 95–98					
Asp^96^	C/sNC	94, 95, 97–100					
Lys^103^	C	99, 100–102, 104–107					
Lys^104^	sNC	100–103, 105–107					

**Table 3:** Proposed thermodynamic hot spot residues by SpotOn and PROT-ON. All presented residues were proposed as thermodynamic hot spots. Residues not included as participating in the contact areas of HsTrx2 by the approach of Section 3.4.2 (contact hot spots and their energy satellites) are shown in red (in total, 13 residues: 40, 42, 49, 59, 60, 63, 64, 67, 69, 80, 81, 84, and 85). Residues in yellow boxes were highlighted by both PROT-ON and SpotOn approaches.

Predicted Higher Energy Thermodynamic Hot Spots			Contact Hot Spots Plus Energy Neighbors		
SpotOn	PROT ON Enriching	PROT-On Depleting	Common C and sNC	Only C	Only sNC
10, 13, 30, 32, 33, 36, 42, 50, 53, 59, 60, 63, 64, 69, 70, 71, 73, 74, 80, 81, 84, 85, 87, 88, 90, 91, 94, 102, 103, 106, 107	1, 3, 10, 12, 13, 29, 30, 32, 33, 40, 49, 56, 60, 64, 70, 72, 84, 94, 96, 97, 104	7, 8, 10, 30, 32, 46, 50, 63, 67, 69, 70–73, 75, 87, 90, 91, 95, 96	33, 37 71–73 77–79 87–90, 94–107	28–32 34–36 38, 39 70	1–18 46, 50 53–56 74–76 91–93

**Table 4:** RMSDs (in Å) between the superimposed minimized energy structures of HsTrx1, HsTrx2, EcoTrx1, and EcoTrx2. Numbers in parentheses correspond to the number of pair atoms used for the acquired RMSDs.

	EcoTrx1	EcoTrx2	HsTrx1	HsTrx2
HsTrx2	3.3 (101)	1.9 (104)	1.9 (104)	
EcoTrx1		1.3 (105)	2.4 (103)	3.3 (101)
EcoTrx2	1.3 (105)		4.2 (101)	1.9 (104)

**Table 5:** Contact areas of HsTrx2 (**A**) and the respective residues of HsTrx1 (**B**), EcoTrx1 (**C**), and EcoTrx2 (**D**). In red are the residues for covalent interactions (Only C), in blue are residues for strong non-covalent interactions (Only sNC), and in green are the residues involved in both covalent and non-covalent interactions (Common). Quotation marks reflect the uncertainty of the type of binding for the indicated areas of HsTrx1 and EcoTrx1, 2.

**(A) HsTrx2**		
**Only C**	**Only sNC**	**Common**
**28–32, 34–36, 38, 39, 70**	**1–18, 46, 50, 53–56 74–76, 91–93**	**33, 37, 71–73, 77–79 87–90, 94–107**
**(B) HsTrx1**		
**“Only C”**	**“Only sNC”**	**“Common”**
**29–33, 35–37, 39, 40, 70**	**1–18, 47, 50, 53–56 74–76, 91–93**	**34, 38, 71–73, 77–79 87–90, 94–107**
**(C) EcoTrx1**		
**“Only C”**	**“Only sNC”**	**“Common”**
**29–33, 35–37, 39, 40, 71**	**2–19, 47, 50, 54–57 75–77, 92–94**	**34, 38, 72–74, 78–80 88–91, 95–108**
**(D) EcoTrx2**		
**“Only C”**	**“Only sNC”**	**“Common”**
**27–31, 33–35, 37, 38, 69**	**2–17, 45, 48, 52–55 73–75, 90–92**	**32, 36, 70–72, 76–78 86–89, 93–105**

**Table 6:** Contact residues of EcoTrx1 with g5p, TrxR, MsrA, PAPS reductase (PAPS red), and corresponding residues in HsTrx2. With red are the contact hot spots of HsTrx2 for molecular recognition and with green are the identical corresponding contact residues of EcoTrx1 (all interactions are presented in Supplementary Data S6, part 1). Numbers in the PDB ID columns signify the numbers of interactions of specific residues of EcoTrx1 with residues of ligands (all interactions presented in Supplementary Data S6). The “% preference” shows how statistically important was the respective residue for contacts with the substrates for EcoTrx1. The gray shading in the column “Residue in EcoTrx1” indicates the amino acids that did not correspond to the contact hot spots/interacting area of HsTrx2. The numbering 1 in the column “Contact area” corresponds to residues assigned herein exclusively for covalent interactions, 2 for residues involved only in strong non-covalent interactions and 3 corresponds to residues involved in both covalent and strong non-covalent interactions. “Cleft” corresponds to the groove region next to the Trp adjacent to the active site.

Residue in EcoTrx1	g5p 1T7P [32]	TrxR 1F6M [61]	Msr A6YEV	PAPS Red 2O8V [62]	Total Hits/Residue	Preference in EcoTrx1 (%)	Residue in HsTrx2	Contact Area
Glu^30^				1	1	2.6	Gln^29^	1
Trp^31^	1	1	1	1	4	10.3	**Trp^30^**	
Cys^32^	1	1	1	1	4	10.3	Cys^31^	
Pro^34^	1	1	1	1	4	10.3	**Pro^33^**	3
Lys^36^				1	1	2.6	**Lys^35^**	1
Met^37^	1	1			2	5.1	Ile^36^	
Pro^40^		1			1	2.6	Pro^39^	
Ile^60^	1			1	2	5.1	Ile^59^	Cleft
Ala^67^	1				1	2.6	Ala^66^	
Pro^68^	1				1	2.6	Ile^67^	
Tyr^70^		1			1	2.6	Tyr^69^	
Arg^73^	1	1	1	1	4	10.3	**Ser^72^**	3
Ile^75^	1	1	1	1	4	10.3	Val^74^	2
Thr^89^	1				1	2.6	**Lys^88^**	3
Lys^90^			1		1	2.6	Phe^89^	
Val^91^	1		1		2	5.1	**Val^90^**	
Ala^93^	1	1	1	1	4	10.3	**Ile^92^**	2
Leu^94^	1				1	2.6	Lys^93^	

**Table 7:** Contact residues of HsTrx1 with SlrP, TrxR, and Txnip and corresponding residues in HsTrx2. With red are the contact hot spots of HsTrx2 for molecular recognition and with green are the identical residues for HsTrx1. The numbers in the PDB ID columns indicate interactions for the specific residues of HsTrx1 (all interactions presented in Supplementary Data S6, part 2). The “% preference” shows how statistically significant was the respective residue for contacts with the substrates for HsTrx1. The gray shading in the column “Residue in HsTrx1” indicates the amino acids do not correspond to the hot spots/interacting area of HsTrx2. The number 1 in the column “Contact area” corresponds to residues assigned herein for covalent interactions, 2 to the exclusive region for strong non-covalent interactions, and 3 to residues involved in both covalent and strong non-covalent interactions. “Cleft” corresponds to the groove region next to the Trp just before the active site.

Residue in HsTrx1	SlrP 4PUF [23]	Trx R3QFA [64]	TXNIP 4LL4 [65]	Total Hits/Residue	Preference in HsTrx1 (%)	Residue in HsTrx2	Contact Area
Trp^31^		1	1	2	7.7	**Trp^30^**	1
Cys^32^		1	1	2	7.7	Cys^31^	
Gly^33^			1	1	3.8	Gly^32^	
Pro^34^			1	1	3.8	**Pro^33^**	3
Lys^36^		1		1	3.8	Lys^35^	1
Val^59^	1	1		2	7.7	Ile^59^	Cleft
Asp^60^		1	1	2	7.7	Asp^60^	
Ala^66^		1		1	3.8	Ala^66^	
Glu^70^		1		1	3.8	**Glu^70^**	1
Val^71^		1		1	3.8	Val^71^	3
Lys^72^	1	1		2	7.7	**Ser^72^**	
Cys^73^	1		1	2	7.7	**Ala^73^**	
Met^74^	1	1	1	3	11.5	Val^74^	2
Pro^75^	1			1	3.8	Pro^75^	
Glu^88^	1			1	3.8	**Lys^88^**	3
Ala^92^	1		1	2	7.78	**Ile^92^**	2
Lys^96^	1			1	3.8	Gln^97^	3

**Table 8:** The effect of point mutations of Ile^59^ and Asp^60^ of HsTrx2 to Alas in its interaction with ligands. PRODIGY was used to calculate thermodynamic parameters before and after point mutations. “wt” stands for wild type.

UniProt ID	Protein (Ligand of HsTrx2)	HsTrx2	ΔG (kcal/mol)	ΔΔG (kcal/mol)	*K_D_* (nM)
P32119	Peroxiredoxin-2	wt	−13.1	0	0.23
		I59A	−12.9	−0.2	0.33
P00441	Superoxide dismutase 1	wt	−8.2	0	1000
		I59A	−8.2	0	1000
P07237	Protein disulfide isomerase	wt	−9.1	0	190
		I59A	−9.1	0	190
		D60A	−9.3	0.2	150
		I59A D60A	−9.3	0.2	150
P08559	Pyruvate dehydrogenase E1 component subunit α	wt	−11.4	0	4.5
		D60A	−11.4	0	4.4
P11177	Pyruvate dehydrogenase E1 component subunit β	wt	−8.4	0	720
		D60A	−8.4	0	740

## Data Availability

Data are contained within the article and Supplementary Materials.

## Supplementary Materials

The following supporting information can be downloaded at: https://www.mdpi.com/article/10.3390/antiox13010015/s1, Supplementary Data S1: Calculation of Binding affinities of HsTrx2-ligand complexes by PRODIGY; Supplementary Data S2: Mapping of HsTrx2-ligand interactions; Supplementary Data S3: Contact hot spots of HsTrx2; Supplementary Data S4: Prediction of possible thermodynamic hot spots by SpotOn and PROT-ON; Supplementary Data S5: Network parameters; Supplementary Data S6: Contact residues of EcoTrx1 and HsTrx1 in their crystal complexes with protein ligands.
