# Supplementary material for: Distinct or Overlapping Areas of Mitochondrial Thioredoxin 2 May Be Used for Its Covalent and Strong Non-Covalent Interactions with Protein Ligands
Source: Antioxidants (Basel). 2023 Dec 20;13(1):15. doi: 10.3390/antiox13010015 (PMC10812433; doi:10.3390/antiox13010015)
Supplement: Supplementary file 1 [file antioxidants-13-00015-s001.zip › Supplementary data S1 (PRODIGY).pdf]

Supplementary Data S1: *Calculation of binding affinities of HsTrx2-ligand complexes by PRODIGY*

Distinct or shared areas of mitochondrial thioredoxin 2 may be used for its covalent and strong non-covalent interactions with protein ligands

Charalampos Ntallis [1], Haralambos Tzoupis [1], Theodore Tselios [1], Christos T. Chasapis [2] and Alexios Vlamis-Gardikas [1,*]

[1] Department of Chemistry, University of Patras, Rion 26504, Greece; xntallis@gmail.com, c.ntallis@uu.nl (C.N.); haralambostz@gmail.com (H.T.); ttselios@upatras.gr (T.T.)
[2] Institute of Chemical Biology, National Hellenic Research Foundation, Vas. Constantinou 48 av, Athens, 11635, Greece; cchasapis@eie.gr. (C.T.C).

*Correspondence: avlamis@upatras.gr; Tel.: +30-2610-997634

1. **Nucleoside diphosphate-linked moiety X motif 19 (UniProt KB: A8MXV4):**

BINDING AFFINITY AND K_D PREDICTION

The binding affinity (ΔG) and dissociation constant (K_d)predicted values are:

**Protein-protein complex**	**ΔG (kcal mol^{-1})**	**K_D (M) at 25.0 ℃**
HsTrx2-A8MXV4	-10.6	1.8E-08

PREDICTION DETAILS

Number of Interfacial Contacts (ICs) per property:

ICs charged-charged:	7
ICs charged-polar:	8
ICs charged-apolar:	44
ICs polar-polar:	3
ICs polar-apolar:	11
ICs apolar-apolar:	16

Non-Interacting Surface (NIS) per property:

NIS charged:	28.41 %
NIS apolar:	45.13 %

Table of the ICs at the interface:

HsTrx2 residue(#1)	Number(#1)	Chain(#1)	A8MXV4 residue(#2)	Number(#2)	Chain(#2)
LYS	88	A	TRP	255	B
TYR	69	A	GLU	253	B
GLN	12	A	HIS	346	B
GLY	83	A	ARG	347	B
ALA	79	A	ARG	347	B
GLY	83	A	MET	302	B
PHE	11	A	ARG	347	B
LYS	88	A	GLY	58	B
GLY	83	A	TRP	255	B
LYS	88	A	PRO	51	B
ASP	84	A	MET	302	B
GLN	12	A	THR	298	B
GLN	12	A	THR	344	B
TYR	69	A	SER	251	B
LYS	88	A	ILE	254	B
VAL	16	A	HIS	346	B
GLU	70	A	LYS	252	B
PHE	26	A	HIS	348	B
MET	80	A	LYS	252	B
ASN	17	A	LEU	297	B
VAL	24	A	LYS	252	B

GLU	68	A	LEU	349	B
VAL	85	A	ALA	59	B
LEU	78	A	LYS	252	B
ASP	84	A	LEU	205	B
LYS	88	A	GLN	48	B
GLY	83	A	TYR	350	B
TYR	69	A	HIS	346	B
GLU	70	A	LEU	250	B
LEU	65	A	HIS	346	B
PHE	26	A	ARG	347	B
ASN	17	A	ALA	299	B
ASP	84	A	TYR	350	B
ASN	82	A	ARG	206	B
GLN	12	A	ALA	299	B
GLU	68	A	SER	251	B
ASP	84	A	ARG	347	B
GLY	83	A	HIS	346	B
VAL	86	A	MET	56	B
ASP	84	A	TRP	255	B
PHE	11	A	HIS	346	B
TYR	69	A	LYS	252	B
VAL	86	A	PRO	57	B
TYR	69	A	PHE	249	B
GLU	68	A	HIS	346	B
GLN	12	A	GLY	301	B
LEU	78	A	HIS	348	B
TYR	69	A	LEU	250	B
VAL	86	A	SER	50	B
VAL	86	A	TRP	255	B
LYS	88	A	LYS	252	B
THR	76	A	LYS	252	B
VAL	71	A	SER	251	B
VAL	86	A	GLY	58	B
VAL	86	A	HIS	52	B
VAL	24	A	ARG	347	B
ASN	17	A	ASP	300	B
VAL	86	A	PRO	51	B
LEU	78	A	ARG	347	B
PHE	11	A	LYS	252	B
GLN	12	A	MET	302	B
ASN	82	A	MET	302	B
GLU	68	A	HIS	348	B
GLN	12	A	ASP	300	B
LYS	88	A	HIS	60	B
VAL	85	A	PRO	57	B
VAL	86	A	ARG	49	B

ASN	17	A	THR	298	B
VAL	16	A	ARG	347	B
TYR	69	A	HIS	348	B
LEU	65	A	HIS	348	B
ASP	87	A	PRO	57	B
GLU	68	A	LEU	250	B
LEU	65	A	LYS	252	B
VAL	16	A	TYR	345	B
VAL	85	A	ARG	347	B
LYS	88	A	GLU	253	B
VAL	85	A	TRP	255	B
GLU	70	A	SER	251	B
ASN	82	A	GLY	207	B
VAL	85	A	GLY	58	B
PHE	26	A	LYS	252	B
LYS	104	A	PRO	51	B
ASP	87	A	GLY	58	B
MET	80	A	ARG	347	B
VAL	71	A	LYS	252	B
ASP	87	A	PRO	51	B
VAL	86	A	ALA	59	B
GLN	12	A	TYR	345	B

2. Cytochrome c oxidase subunit NDUFA4 (UniProt KB: O00483):

BINDING AFFINITY AND K_D PREDICTION

The binding affinity (ΔG) and dissociation constant (K_d)predicted values are:

**Protein-protein complex**	**ΔG (kcal mol^{-1})**	**K_D (M) at 25.0 ℃**
HsTrx2-O00483	-8.5	5.9E-07

PREDICTION DETAILS

Number of Interfacial Contacts (ICs) per property:

ICs charged-charged:	2
ICs charged-polar:	1
ICs charged-apolar:	9
ICs polar-polar:	1
ICs polar-apolar:	18
ICs apolar-apolar:	11

Non-Interacting Surface (NIS) per property:

NIS charged:	27.98 %
NIS apolar:	45.83 %

Table of the ICs at the interface:

HsTrx2 residue	Number(#1)	Chain(#1)	O00483 residue	Number(#2)	Chain(#2)
ARG	14	A	LEU	18	B
VAL	52	A	LEU	15	B
ILE	5	A	PHE	21	B
PHE	3	A	PHE	21	B
GLY	50	A	SER	14	B
THR	2	A	ILE	22	B
ASN	4	A	VAL	20	B
HIS	49	A	LEU	15	B
SER	18	A	LEU	18	B
VAL	53	A	LEU	15	B
ASP	13	A	PHE	21	B
ASP	10	A	PHE	21	B
GLY	50	A	LEU	15	B
VAL	53	A	LEU	18	B
GLN	6	A	PHE	21	B
HIS	49	A	GLN	8	B
HIS	49	A	HIS	12	B
ARG	14	A	PRO	17	B
ILE	5	A	ILE	22	B
GLN	6	A	GLY	25	B
PHE	3	A	LEU	18	B
GLY	50	A	HIS	12	B
ASN	4	A	PHE	21	B
PHE	3	A	PHE	19	B
ARG	14	A	PHE	21	B
LYS	56	A	ILE	22	B
ASN	4	A	GLY	25	B
ASN	4	A	LEU	18	B
THR	1	A	LEU	15	B
PHE	3	A	ILE	22	B
PHE	3	A	VAL	20	B
GLN	6	A	VAL	20	B
THR	1	A	LEU	18	B
LYS	51	A	HIS	12	B
THR	1	A	PHE	19	B
ASN	4	A	ILE	22	B
ASP	61	A	GLY	25	B
THR	2	A	LEU	18	B
ASN	4	A	ALA	26	B
GLN	6	A	THR	24	B
ASN	4	A	GLY	23	B
THR	2	A	PHE	19	B

3. NADH dehydrogenase [ubiquinone] iron-sulfur protein 2, mitochondrial

(UniProt KB: O75306):

Binding affinity and K_D prediction

The binding affinity (ΔG) and dissociation constant (K_d) predicted values are:

Protein-protein complex	ΔG (kcal mol^{-1})	K_D (M) at 25.0 °C
HsTrx2-O75306	-9.8	6.1E-08

Prediction details

Number of Interfacial Contacts (ICs) per property:

ICs charged-charged:	9
ICs charged-polar:	16
ICs charged-apolar:	27
ICs polar-polar:	6
ICs polar-apolar:	16
ICs apolar-apolar:	15

Non-Interacting Surface (NIS) per property:

NIS charged:	29.96 %
NIS apolar:	42.70 %

Table of the ICs at the interface:

HsTrx2 residue	Number(#1)	Chain(#1)	O75306 residue	Number(#2)	Chain(#2)
LEU	102	A	ALA	241	B
THR	1	A	THR	46	B
GLU	99	A	ALA	241	B
LYS	47	A	PHE	242	B
LYS	51	A	THR	43	B
LYS	103	A	GLU	240	B
PRO	39	A	ARG	142	B
ASN	4	A	ARG	48	B
ARG	14	A	THR	46	B
ASP	13	A	PRO	45	B
VAL	52	A	THR	46	B
ARG	14	A	PRO	45	B
LYS	103	A	PHE	242	B
VAL	53	A	THR	46	B
ILE	106	A	PHE	242	B
ILE	5	A	ARG	48	B
LYS	103	A	PRO	238	B
GLU	19	A	THR	46	B
GLU	19	A	THR	43	B
GLU	42	A	GLU	83	B
THR	1	A	PHE	81	B

THR	2	A	PHE	81	B
SER	18	A	PRO	45	B
LYS	104	A	LEU	235	B
MET	44	A	PHE	242	B
LYS	43	A	PHE	81	B
ILE	106	A	ALA	241	B
ARG	40	A	VAL	117	B
LYS	35	A	ASN	131	B
GLN	97	A	LYS	200	B
ASN	17	A	THR	46	B
THR	20	A	VAL	47	B
GLN	48	A	PHE	242	B
ALA	100	A	SER	237	B
VAL	52	A	PHE	242	B
PRO	21	A	THR	46	B
ASN	17	A	PRO	45	B
LYS	35	A	THR	115	B
ALA	55	A	ARG	48	B
ASP	94	A	LYS	200	B
ASP	96	A	ASN	236	B
THR	1	A	PRO	49	B
GLU	99	A	GLU	240	B
VAL	22	A	THR	46	B
ALA	100	A	LEU	235	B
GLY	50	A	THR	43	B
ILE	36	A	THR	115	B
ARG	14	A	PRO	49	B
GLN	97	A	ASN	236	B
GLU	99	A	SER	237	B
GLU	95	A	TYR	245	B
GLY	107	A	ALA	241	B
ARG	14	A	ARG	48	B
GLU	19	A	PRO	45	B
GLU	42	A	PHE	81	B
THR	20	A	ARG	44	B
THR	1	A	VAL	47	B
HIS	49	A	CYS	80	B
LYS	103	A	ALA	241	B
ARG	14	A	VAL	47	B
PHE	11	A	ARG	48	B
ILE	36	A	VAL	117	B
LYS	103	A	TRP	239	B
VAL	53	A	VAL	47	B
ASP	10	A	ARG	48	B
VAL	45	A	PHE	81	B
THR	20	A	THR	46	B

ALA	100	A	ASN	236	B
ILE	36	A	ALA	116	B
LYS	103	A	SER	237	B
THR	20	A	THR	43	B
VAL	45	A	PHE	242	B
ALA	46	A	PHE	81	B
THR	1	A	ARG	48	B
LYS	35	A	ASP	113	B
LYS	43	A	GLN	77	B
THR	2	A	ARG	48	B
LEU	102	A	PHE	242	B
ARG	14	A	ARG	44	B
GLU	99	A	PHE	242	B
SER	18	A	THR	46	B
LYS	47	A	VAL	244	B
PHE	3	A	ARG	48	B
VAL	53	A	ARG	48	B
GLU	19	A	ARG	44	B
LYS	47	A	TYR	245	B
GLY	50	A	VAL	47	B
ASP	13	A	THR	46	B
HIS	49	A	VAL	244	B

4. NADH dehydrogenase [ubiquinone] iron-sulfur protein 3, mitochondrial (UniProt KB: O75489):

Binding affinity and K$_D$ prediction

The binding affinity (ΔG) and dissociation constant (K_d)predicted values are:

Protein-protein complex	ΔG (kcal mol^{-1})	K_D (M) at 25.0 ℃
HsTrx2-O75489	-12.1	1.3E-09

Prediction details

Number of Interfacial Contacts (ICs) per property:

ICs charged-charged:	6
ICs charged-polar:	4
ICs charged-apolar:	25
ICs polar-polar:	1
ICs polar-apolar:	24
ICs apolar-apolar:	15

Non-Interacting Surface (NIS) per property:

NIS charged:	29.96 %
NIS apolar:	42.80 %

Table of the ICs at the interface:

HsTrx2 residue	Number(#1)	Chain(#1)	O75489 residue	Number(#2)	Chain(#2)
ASP	7	A	TYR	119	B
HIS	49	A	GLY	73	B
THR	1	A	PRO	71	B
THR	2	A	PRO	71	B
GLU	42	A	TYR	70	B
LYS	56	A	TYR	70	B
ASP	61	A	TYR	192	B
THR	1	A	LYS	75	B
ILE	5	A	LEU	80	B
GLN	6	A	LEU	80	B
ASP	60	A	LEU	177	B
PHE	3	A	TYR	70	B
THR	2	A	ASN	69	B
ASP	13	A	GLU	114	B
ASP	13	A	ARG	111	B
GLN	6	A	TYR	119	B
GLN	29	A	TYR	192	B
HIS	49	A	PRO	71	B
PRO	9	A	TYR	119	B
ARG	14	A	ARG	111	B
ALA	46	A	GLU	72	B
GLY	8	A	PHE	118	B
THR	1	A	ALA	78	B
THR	2	A	ALA	78	B
ALA	46	A	TYR	70	B
ASP	10	A	LEU	80	B
PHE	3	A	ALA	78	B
GLN	6	A	VAL	115	B
ASP	7	A	THR	120	B
ASN	4	A	PRO	81	B
ARG	14	A	ALA	76	B
GLY	50	A	PRO	71	B
ASP	60	A	CYS	176	B
THR	1	A	HIS	74	B
GLN	6	A	TYR	122	B
ASP	7	A	PHE	118	B
THR	2	A	HIS	74	B
ARG	14	A	LEU	80	B
ARG	14	A	HIS	74	B
THR	63	A	GLY	178	B
VAL	45	A	PRO	71	B
HIS	62	A	TYR	119	B
HIS	49	A	HIS	74	B

VAL	53	A	HIS	74	B
ASP	10	A	VAL	115	B
ASN	4	A	ALA	77	B
PHE	3	A	PRO	81	B
ALA	46	A	PRO	71	B
ARG	14	A	VAL	79	B
GLN	6	A	PHE	118	B
PRO	9	A	PHE	118	B
ASN	4	A	LEU	80	B
ASP	61	A	TYR	119	B
ASP	13	A	VAL	115	B
THR	1	A	ALA	76	B
ASP	60	A	GLY	178	B
THR	1	A	TYR	70	B
ALA	46	A	ASN	69	B
THR	2	A	TYR	70	B
ARG	14	A	VAL	115	B
THR	1	A	ALA	77	B
LYS	47	A	PRO	71	B
HIS	49	A	GLU	72	B
THR	2	A	ALA	77	B
PHE	11	A	PHE	118	B
PHE	3	A	ALA	76	B
PRO	9	A	GLU	114	B
PHE	3	A	ALA	77	B
GLY	50	A	HIS	74	B
PHE	3	A	LEU	80	B
GLN	6	A	PRO	81	B
ASP	10	A	PHE	118	B
ASN	4	A	TYR	70	B
THR	63	A	CYS	176	B
THR	63	A	LEU	177	B

- **Carbonyl reductase [NADPH] 3 (UniProt KB: O75828):**

Binding affinity and K_D prediction

The binding affinity (ΔG) and dissociation constant (K_d)predicted values are:

Protein-protein complex	ΔG (kcal mol^{-1})	K_D (M) at 25.0 ℃
HsTrx2-O75828	-8.4	6.4E-07

Prediction details

Number of Interfacial Contacts (ICs) per property:

ICs charged-charged:	11
ICs charged-polar:	9
ICs charged-apolar:	24
ICs polar-polar:	2

ICs polar-apolar:	7
ICs apolar-apolar:	10

Non-Interacting Surface (NIS) per property:

NIS charged:	37.36 %
NIS apolar:	37.36 %

Table of the ICs at the interface:

HsTrx2 residue	Number(#1)	Chain(#1)	O75828 residue	Number(#2)	Chain(#2)
SER	72	A	MET	109	B
ALA	73	A	GLU	108	B
SER	72	A	LYS	106	B
GLU	70	A	LYS	173	B
SER	72	A	ILE	105	B
GLU	70	A	GLU	108	B
ILE	67	A	LYS	180	B
VAL	86	A	SER	69	B
LYS	88	A	ASP	66	B
ASP	64	A	GLU	177	B
VAL	86	A	GLN	68	B
PHE	89	A	LYS	112	B
VAL	90	A	LYS	112	B
SER	72	A	ASP	104	B
ALA	66	A	LYS	180	B
ILE	67	A	VAL	176	B
ILE	67	A	GLU	177	B
THR	76	A	LYS	112	B
VAL	86	A	ARG	71	B
GLU	70	A	PHE	116	B
VAL	71	A	LYS	180	B
TRP	30	A	ILE	105	B
LYS	104	A	GLN	68	B
VAL	90	A	MET	109	B
ASP	84	A	LEU	67	B
SER	72	A	THR	110	B
ASP	87	A	GLN	68	B
SER	72	A	LYS	180	B
ILE	67	A	ASN	181	B
ILE	67	A	LYS	173	B
GLU	70	A	MET	172	B
VAL	86	A	LEU	67	B
GLU	68	A	LYS	173	B
TRP	30	A	PRO	102	B
ILE	67	A	GLU	108	B

GLU	70	A	VAL	169	B
THR	76	A	MET	109	B
VAL	71	A	VAL	176	B
VAL	85	A	ARG	71	B
PHE	101	A	ASP	66	B
ILE	59	A	GLU	108	B
ASP	87	A	ASP	65	B
VAL	86	A	ASP	66	B
LEU	105	A	GLN	68	B
SER	72	A	LYS	112	B
ALA	73	A	MET	109	B
THR	63	A	LYS	180	B
LYS	88	A	PHE	116	B
ILE	59	A	ILE	105	B
ALA	73	A	ILE	105	B
LYS	88	A	ASP	65	B
VAL	85	A	LEU	67	B
THR	63	A	ASN	181	B
VAL	71	A	GLU	108	B
ASP	87	A	LEU	67	B
ASP	84	A	ARG	71	B
ILE	67	A	ASP	178	B
GLU	70	A	LYS	112	B
SER	72	A	GLU	108	B
ASP	87	A	ASP	66	B
LYS	88	A	LYS	112	B
ALA	66	A	GLU	108	B
LYS	88	A	LEU	67	B

5. Cytosolic 10-formyltetrahydrofolate dehydrogenase (UniProt KB: O75891):

Binding affinity and K_D prediction

The binding affinity (ΔG) and dissociation constant (K_d)predicted values are:

Protein-protein complex	ΔG (kcal mol^{-1})	K_D (M) at 25.0 ℃
HsTrx2-O75891	-9.8	6.0E-08

Prediction details

Number of Interfacial Contacts (ICs) per property:

ICs charged-charged:	18
ICs charged-polar:	6
ICs charged-apolar:	34
ICs polar-polar:	0
ICs polar-apolar:	5
ICs apolar-apolar:	11

Non-Interacting Surface (NIS) per property:

NIS charged:	33.04 %
NIS apolar:	41.59 %

Table of the ICs at the interface:

HsTrx2 residue	Number(#1)	Chain(#1)	O75891 residue	Number(#2)	Chain(#2)
GLY	107	A	ARG	491	B
HIS	49	A	ALA	326	B
LYS	103	A	LEU	371	B
LYS	51	A	THR	317	B
GLY	107	A	GLY	370	B
LYS	51	A	VAL	322	B
ILE	106	A	ASP	369	B
ASP	87	A	GLU	497	B
HIS	49	A	VAL	322	B
VAL	52	A	ASP	369	B
HIS	49	A	ARG	333	B
GLN	48	A	LEU	367	B
LYS	104	A	ARG	491	B
GLN	97	A	ARG	531	B
LYS	103	A	TYR	490	B
LYS	47	A	GLU	366	B
VAL	86	A	GLN	498	B
MET	44	A	GLU	366	B
ILE	106	A	CYS	368	B
GLU	99	A	GLY	370	B
HIS	49	A	GLU	366	B
GLN	48	A	ALA	326	B
GLY	107	A	ASP	369	B
LYS	104	A	GLN	498	B
LYS	103	A	ARG	491	B
LYS	103	A	GLY	370	B
LYS	51	A	GLU	320	B
MET	44	A	ASP	369	B
PHE	101	A	ARG	531	B
ALA	46	A	GLU	366	B
ASP	96	A	ARG	483	B
LYS	51	A	LEU	367	B
LYS	47	A	CYS	368	B
LYS	51	A	THR	323	B
GLN	97	A	TYR	490	B
GLY	50	A	LEU	367	B
GLU	99	A	ASP	369	B

GLN	48	A	GLU	366	B
LEU	102	A	GLY	370	B
LYS	93	A	ARG	531	B
LYS	103	A	ASP	369	B
ALA	100	A	ARG	487	B
ILE	106	A	LEU	367	B
GLN	48	A	ASP	369	B
PHE	101	A	ASP	494	B
GLY	50	A	VAL	322	B
LYS	103	A	LEU	488	B
GLY	107	A	GLY	397	B
VAL	45	A	ASP	369	B
LEU	102	A	ASP	369	B
ALA	100	A	ASP	494	B
GLY	107	A	LEU	371	B
GLY	107	A	LEU	395	B
GLN	48	A	CYS	368	B
ALA	100	A	TYR	490	B
LYS	47	A	LEU	367	B
LYS	51	A	ALA	319	B
LYS	51	A	LEU	395	B
GLU	99	A	ARG	487	B
LYS	47	A	GLU	363	B
LYS	88	A	GLU	497	B
VAL	86	A	ASP	494	B
LYS	103	A	ARG	487	B
HIS	49	A	LEU	367	B
ALA	100	A	ARG	491	B
GLY	107	A	LYS	394	B
GLY	107	A	ARG	396	B
LYS	104	A	ASP	494	B
ASP	96	A	ARG	487	B
GLU	19	A	GLU	318	B
ILE	106	A	LEU	395	B
GLU	99	A	LEU	371	B
VAL	86	A	GLU	497	B
HIS	49	A	VAL	330	B

6. Cytochrome c oxidase subunit 1 (UniProt KB: P00395):

Binding affinity and K_D prediction

The binding affinity (ΔG) and dissociation constant (K_d)predicted values are:

Protein-protein complex	ΔG (kcal mol^{-1})	K_D (M) at 25.0 ℃
HsTrx2-P00395	-9.9	5.8E-08

Prediction details

Number of Interfacial Contacts (ICs) per property:

ICs charged-charged:	1
ICs charged-polar:	5
ICs charged-apolar:	37
ICs polar-polar:	1
ICs polar-apolar:	12
ICs apolar-apolar:	20

Non-Interacting Surface (NIS) per property:

NIS charged:	14.10 %
NIS apolar:	55.95 %

Table of the ICs at the interface:

HsTrx2 residue	Number(#1)	Chain(#1)	P00395 residue	Number(#2)	Chain(#2)
ILE	106	A	LEU	113	B
LYS	81	A	MET	117	B
GLN	97	A	MET	466	B
LYS	103	A	LEU	29	B
ASP	84	A	MET	117	B
ASP	87	A	LEU	48	B
LYS	81	A	VAL	118	B
VAL	85	A	ALA	120	B
PHE	101	A	LEU	36	B
LYS	81	A	ALA	116	B
PHE	89	A	LEU	47	B
VAL	90	A	LEU	47	B
GLU	70	A	LEU	48	B
ASP	84	A	VAL	118	B
LYS	88	A	GLY	49	B
SER	72	A	ASN	46	B
ASP	96	A	VAL	465	B
ASP	84	A	ALA	116	B
VAL	86	A	MET	117	B
LYS	88	A	LEU	48	B
LYS	104	A	ALA	32	B
ASP	87	A	ILE	53	B
VAL	86	A	ILE	53	B
GLY	83	A	GLU	119	B
ASN	82	A	MET	117	B
PRO	21	A	MET	117	B
LYS	88	A	LEU	47	B
VAL	86	A	ALA	116	B
THR	76	A	LEU	47	B

GLU	70	A	ASN	46	B
SER	72	A	LEU	48	B
LYS	104	A	LEU	113	B
ASN	82	A	VAL	118	B
PHE	89	A	LEU	48	B
ALA	100	A	ALA	32	B
VAL	86	A	VAL	56	B
LYS	104	A	LEU	36	B
LYS	104	A	LEU	29	B
GLN	97	A	LEU	35	B
VAL	86	A	ILE	57	B
ASP	87	A	LEU	36	B
GLN	97	A	PHE	459	B
VAL	85	A	ILE	53	B
LYS	104	A	LEU	33	B
ASN	82	A	ALA	114	B
GLU	70	A	ASN	50	B
ASP	84	A	GLY	121	B
SER	72	A	GLY	49	B
LYS	104	A	SER	34	B
ASP	84	A	GLU	119	B
LEU	105	A	LEU	113	B
GLY	83	A	MET	117	B
LYS	88	A	ILE	53	B
ASP	84	A	ALA	120	B
LYS	104	A	ILE	57	B
SER	72	A	LEU	47	B
PHE	89	A	LEU	36	B
GLU	70	A	GLY	49	B
ALA	100	A	VAL	28	B
ASP	87	A	ILE	57	B
ASP	96	A	THR	463	B
GLN	97	A	ALA	32	B
ASP	94	A	MET	466	B
LYS	93	A	LEU	36	B
ASP	96	A	LEU	462	B
ASN	82	A	GLU	119	B
LYS	88	A	ILE	57	B
GLU	70	A	LEU	47	B
LYS	104	A	VAL	28	B
LYS	81	A	LEU	113	B
LYS	88	A	LEU	36	B
PHE	101	A	ALA	32	B
GLN	97	A	LEU	462	B
LYS	93	A	LEU	35	B
GLY	107	A	LEU	29	B

ASP	96	A	MET	466	B

7. **Superoxide dismutase 1, soluble (UniProt KB: P00441):**

BINDING AFFINITY AND K$_D$ PREDICTION

The binding affinity (ΔG) and dissociation constant (K_d)predicted values are:

**Protein-protein complex**	**ΔG (kcal mol^{-1})**	***K$_D$* (M) at 25.0 ℃**
HsTrx2-P00441	-8.2	1.0E-06

PREDICTION DETAILS

Number of Interfacial Contacts (ICs) per property:

ICs charged-charged:	1
ICs charged-polar:	5
ICs charged-apolar:	14
ICs polar-polar:	0
ICs polar-apolar:	14
ICs apolar-apolar:	18

Non-Interacting Surface (NIS) per property:

NIS charged:	32.14 %
NIS apolar:	42.86 %

Table of the ICs at the interface:

HsTrx2 residue	Number(#1)	Chain(#1)	P00441 residue	Number(#2)	Chain(#2)
GLN	29	A	GLY	108	B
ILE	59	A	CYS	111	B
LYS	35	A	ALA	152	B
TRP	30	A	LYS	3	B
TRP	30	A	ILE	112	B
ASP	60	A	GLY	114	B
GLN	29	A	ILE	151	B
ASP	60	A	CYS	111	B
TRP	30	A	GLY	150	B
GLN	29	A	HIS	110	B
THR	63	A	ARG	115	B
ASP	60	A	ILE	112	B
GLN	29	A	ILE	149	B
THR	63	A	GLY	108	B
TRP	30	A	GLY	108	B
TRP	30	A	LEU	106	B
TRP	30	A	SER	105	B
GLN	29	A	ILE	113	B
ASP	58	A	ILE	113	B

GLN	29	A	GLY	114	B
TRP	30	A	ALA	4	B
GLN	29	A	CYS	111	B
GLY	32	A	ILE	151	B
TRP	30	A	GLU	21	B
ASP	58	A	ILE	151	B
CYS	31	A	ILE	151	B
TRP	30	A	ILE	149	B
TRP	30	A	THR	2	B
ALA	28	A	ILE	151	B
GLN	29	A	GLY	150	B
ASP	60	A	ILE	149	B
TRP	30	A	ILE	113	B
ASP	60	A	ARG	115	B
ASP	61	A	GLY	114	B
TRP	30	A	CYS	111	B
TRP	30	A	ILE	151	B
TRP	30	A	VAL	29	B
CYS	31	A	ILE	113	B
ASP	60	A	ILE	113	B
TRP	30	A	HIS	110	B
TRP	30	A	GLN	22	B
SER	34	A	ILE	151	B
ALA	28	A	ILE	113	B
GLN	29	A	ILE	112	B
ASP	60	A	ILE	151	B
TRP	30	A	PHE	20	B
LYS	35	A	ILE	151	B
TRP	30	A	SER	107	B
GLN	29	A	ARG	115	B
GLY	32	A	ALA	152	B
THR	63	A	ASP	109	B
GLN	6	A	ASP	52	B

8. Glyceraldehyde-3-phosphate dehydrogenase (UniProt KB: P04406):

Binding affinity and K_D prediction

The binding affinity (ΔG) and dissociation constant (K_d)predicted values are:

**Protein-protein complex**	**ΔG (kcal mol^{-1})**	**K_D (M) at 25.0 ℃**
HsTrx2-P04406	-10.2	3.2E-08

Prediction details

Number of Interfacial Contacts (ICs) per property:

ICs charged-charged:	8
ICs charged-polar:	3

ICs charged-apolar:	19
ICs polar-polar:	1
ICs polar-apolar:	16
ICs apolar-apolar:	8

Non-Interacting Surface (NIS) per property:

NIS charged:	29.43 %
NIS apolar:	41.46 %

Table of the ICs at the interface:

HsTrx2 residue	Number(#1)	Chain(#1)	P04406 residue	Number(#2)	Chain(#2)
SER	72	A	ALA	62	B
VAL	85	A	ARG	80	B
GLU	70	A	LEU	40	B
GLU	70	A	ASP	39	B
ALA	73	A	ALA	62	B
PRO	33	A	ASN	64	B
LYS	88	A	ILE	76	B
VAL	74	A	ASN	64	B
THR	76	A	GLY	65	B
LEU	78	A	ASP	39	B
LYS	104	A	LYS	86	B
VAL	85	A	GLU	79	B
LYS	88	A	PHE	77	B
ASP	87	A	LYS	86	B
ASP	87	A	GLU	79	B
GLY	91	A	ASN	64	B
VAL	86	A	GLN	78	B
VAL	90	A	LYS	66	B
TYR	69	A	ASN	41	B
VAL	90	A	LEU	40	B
ALA	73	A	ASN	64	B
ALA	73	A	GLY	65	B
TYR	69	A	ASP	39	B
PHE	101	A	ASP	89	B
VAL	71	A	ASN	41	B
LYS	88	A	LEU	40	B
THR	76	A	LEU	40	B
LYS	88	A	ASP	39	B
GLU	70	A	TYR	42	B
VAL	85	A	GLN	78	B
PRO	75	A	ASN	64	B
ASP	87	A	LYS	84	B

ASP	87	A	GLN	78	B
VAL	86	A	LYS	84	B
GLY	83	A	ARG	80	B
GLU	68	A	ASP	39	B
VAL	90	A	ASN	64	B
ASP	84	A	ARG	80	B
GLU	70	A	ASN	41	B
TYR	69	A	LEU	40	B
LEU	78	A	GLN	78	B
LYS	88	A	GLN	78	B
ASP	87	A	PHE	77	B
GLU	70	A	TYR	45	B
ALA	73	A	GLU	63	B
VAL	85	A	PRO	36	B
VAL	86	A	ARG	80	B
ALA	79	A	GLN	78	B
VAL	71	A	LEU	40	B
SER	72	A	ASN	41	B
VAL	71	A	ASP	39	B
PRO	75	A	GLY	65	B
SER	72	A	LEU	40	B
VAL	86	A	GLU	79	B
VAL	90	A	GLY	65	B

9. ATP synthase subunit beta, mitochondrial (UniProt KB: P06576):

BINDING AFFINITY AND K_D PREDICTION

The binding affinity (ΔG) and dissociation constant (K_d)predicted values are:

**Protein-protein complex**	**ΔG (kcal mol^{-1})**	***K_D* (M) at 25.0 °C**
HsTrx2-P06576	-6.7	1.1E-05

PREDICTION DETAILS

Number of Interfacial Contacts (ICs) per property:

ICs charged-charged:	1
ICs charged-polar:	1
ICs charged-apolar:	15
ICs polar-polar:	0
ICs polar-apolar:	9
ICs apolar-apolar:	25

Non-Interacting Surface (NIS) per property:

NIS charged:	29.13 %
NIS apolar:	47.17 %

Table of the ICs at the interface:

HsTrx2 residue	Number(#1)	Chain(#1)	P06576 residue	Number(#2)	Chain(#2)
ILE	92	A	GLU	77	B
GLY	32	A	GLU	105	B
VAL	90	A	GLY	78	B
ALA	73	A	LEU	79	B
CYS	31	A	SER	106	B
ILE	92	A	GLY	57	B
PRO	75	A	GLY	78	B
ALA	73	A	HIS	102	B
PRO	33	A	ASP	76	B
THR	76	A	PRO	81	B
TRP	30	A	GLY	104	B
CYS	31	A	GLY	104	B
CYS	31	A	LEU	79	B
PRO	33	A	GLU	77	B
ALA	73	A	ILE	82	B
VAL	74	A	GLY	78	B
PRO	33	A	SER	106	B
GLY	32	A	SER	106	B
SER	72	A	PRO	80	B
LYS	88	A	PRO	81	B
GLY	91	A	GLY	78	B
ALA	73	A	PRO	81	B
VAL	90	A	LEU	79	B
PRO	33	A	LEU	79	B
ILE	36	A	GLU	77	B
TRP	30	A	GLU	105	B
VAL	90	A	PRO	80	B
ILE	92	A	ALA	59	B
SER	72	A	HIS	102	B
VAL	71	A	ILE	82	B
PRO	75	A	GLU	77	B
SER	72	A	ILE	82	B
VAL	74	A	PRO	80	B
PRO	33	A	GLY	78	B
TRP	30	A	SER	106	B
SER	72	A	PRO	81	B
PRO	75	A	LEU	79	B
GLY	91	A	GLU	77	B
CYS	31	A	GLU	105	B
PRO	33	A	PHE	75	B
GLY	32	A	ASP	76	B
ALA	73	A	PRO	80	B

VAL	74	A	LEU	79	B
GLY	32	A	GLU	77	B
ILE	92	A	GLY	78	B
GLU	70	A	GLU	169	B
VAL	74	A	HIS	102	B
VAL	90	A	PRO	81	B
LYS	93	A	GLY	57	B
GLN	97	A	GLY	57	B
GLY	91	A	LEU	79	B

10. L-lactate dehydrogenase B chain, LDH-B (UniProt KB: P07195):

Binding affinity and K_D prediction

The binding affinity (ΔG) and dissociation constant (K_d)predicted values are:

Protein-protein complex	ΔG (kcal mol^{-1})	K_D (M) at 25.0 ℃
HsTrx2-P07195	-9.3	1.6E-07

Prediction details

Number of Interfacial Contacts (ICs) per property:

ICs charged-charged:	7
ICs charged-polar:	17
ICs charged-apolar:	20
ICs polar-polar:	16
ICs polar-apolar:	25
ICs apolar-apolar:	10

Non-Interacting Surface (NIS) per property:

NIS charged:	31.49 %
NIS apolar:	40.26 %

Table of the ICs at the interface:

HsTrx2 residue	Number(#1)	Chain(#1)	P07195 residue	Number(#2)	Chain(#2)
VAL	16	A	ALA	208	B
ASN	17	A	CYS	186	B
ARG	14	A	CYS	294	B
ARG	14	A	PRO	273	B
THR	20	A	GLN	307	B
LYS	51	A	ASN	306	B
ASN	4	A	ARG	270	B
THR	1	A	ASN	306	B
PHE	3	A	ILE	295	B
PHE	11	A	HIS	187	B
THR	1	A	SER	303	B

HIS	62	A	LEU	268	B
ASN	17	A	GLY	204	B
PHE	3	A	LEU	268	B
THR	2	A	SER	303	B
ARG	14	A	PRO	293	B
GLY	50	A	ILE	305	B
PHE	3	A	ILE	305	B
THR	1	A	VAL	304	B
GLY	50	A	ASN	306	B
ASP	10	A	ILE	271	B
ASP	13	A	TRP	189	B
ASN	4	A	ILE	295	B
ASN	17	A	ALA	208	B
ASP	13	A	ARG	172	B
ILE	5	A	ARG	270	B
GLY	83	A	GLY	209	B
ASN	4	A	LEU	268	B
ASN	4	A	SER	269	B
VAL	53	A	ASN	306	B
GLN	6	A	LEU	296	B
ARG	14	A	TRP	189	B
GLN	12	A	SER	184	B
GLU	19	A	GLY	204	B
ASP	13	A	HIS	187	B
PHE	3	A	ARG	270	B
GLN	6	A	SER	263	B
ASN	17	A	ASN	206	B
ARG	14	A	HIS	187	B
ARG	14	A	GLN	307	B
PHE	3	A	ILE	271	B
ILE	5	A	LEU	268	B
GLN	6	A	MET	264	B
ASP	7	A	ARG	270	B
GLN	12	A	SER	185	B
PHE	3	A	SER	269	B
ASN	17	A	VAL	205	B
GLN	6	A	HIS	272	B
ASN	4	A	ILE	271	B
GLN	6	A	ASP	259	B
THR	20	A	ASN	306	B
PRO	9	A	SER	184	B
GLN	6	A	ARG	270	B
GLY	50	A	LYS	308	B
ASP	13	A	GLY	188	B
VAL	16	A	GLY	209	B
ASN	17	A	HIS	187	B

ASN	17	A	SER	211	B
ASN	4	A	ASN	267	B
GLU	19	A	LYS	308	B
GLN	12	A	HIS	187	B
ILE	5	A	SER	269	B
GLN	6	A	LEU	268	B
GLU	19	A	SER	203	B
GLN	6	A	SER	269	B
ASP	13	A	CYS	186	B
GLY	50	A	GLN	307	B
GLN	6	A	GLU	262	B
THR	1	A	ILE	305	B
THR	2	A	ILE	305	B
GLN	6	A	LYS	266	B
ARG	14	A	ILE	305	B
ASN	17	A	GLY	209	B
ASP	10	A	ARG	270	B
GLU	19	A	VAL	201	B
GLU	19	A	GLN	307	B
GLN	48	A	ASN	306	B
ARG	14	A	HIS	272	B
ASN	82	A	GLY	209	B
ASN	17	A	VAL	210	B
SER	18	A	ASN	206	B
ASP	13	A	SER	184	B
ASN	17	A	VAL	207	B
VAL	53	A	ILE	305	B
HIS	49	A	ASN	306	B
PRO	9	A	ARG	172	B
THR	20	A	LYS	308	B
ASP	10	A	SER	269	B
PRO	9	A	HIS	187	B
ARG	14	A	ILE	271	B
GLU	19	A	LEU	309	B
THR	1	A	ILE	295	B
ASN	82	A	GLU	214	B
ASN	17	A	SER	185	B
THR	2	A	ILE	295	B

11. Protein disulfide-isomerase (UniProt KB: P07237):

BINDING AFFINITY AND K_D PREDICTION

The binding affinity (ΔG) and dissociation constant (K_d)predicted values are:

**Protein-protein complex**	**ΔG (kcal mol^{-1})**	***K_D* (M) at 25.0 ℃**
HsTrx2-P07237	-9.1	1.9E-07

PREDICTION DETAILS

Number of Interfacial Contacts (ICs) per property:

ICs charged-charged:	1
ICs charged-polar:	6
ICs charged-apolar:	17
ICs polar-polar:	2
ICs polar-apolar:	17
ICs apolar-apolar:	18

Non-Interacting Surface (NIS) per property:

NIS charged:	36.59 %
NIS apolar:	37.47 %

Table of the ICs at the interface:

HsTrx2 residue	Number(#1)	Chain(#1)	P07237 residue	Number(#2)	Chain(#2)
CYS	31	A	HIS	438	B
SER	72	A	PRO	441	B
VAL	74	A	PHE	440	B
ILE	67	A	PRO	395	B
SER	72	A	CYS	397	B
ASP	61	A	THR	428	B
ASP	60	A	MET	356	B
VAL	71	A	ALA	394	B
VAL	71	A	TRP	396	B
CYS	31	A	SER	439	B
GLY	32	A	VAL	437	B
GLN	29	A	GLU	431	B
CYS	31	A	VAL	437	B
ALA	73	A	CYS	397	B
SER	72	A	ALA	394	B
SER	72	A	TRP	396	B
ILE	59	A	PHE	440	B
ALA	73	A	PHE	440	B
ALA	66	A	TRP	396	B
GLU	70	A	TRP	396	B
THR	63	A	THR	428	B
THR	63	A	ALA	429	B
TRP	30	A	VAL	437	B
GLN	6	A	GLU	303	B
TRP	30	A	THR	428	B
THR	63	A	PRO	395	B
HIS	62	A	TRP	396	B

SER	72	A	GLY	398	B
ALA	73	A	SER	439	B
PRO	33	A	HIS	438	B
GLY	32	A	LYS	436	B
SER	72	A	PHE	440	B
VAL	74	A	HIS	438	B
TRP	30	A	VAL	435	B
THR	63	A	TRP	396	B
ASP	60	A	THR	428	B
VAL	74	A	SER	439	B
VAL	74	A	VAL	437	B
ASP	64	A	TRP	396	B
TRP	30	A	ASN	430	B
GLY	32	A	HIS	438	B
ASP	61	A	MET	356	B
SER	72	A	SER	439	B
TRP	30	A	GLU	431	B
TRP	30	A	LYS	436	B
TRP	30	A	ALA	429	B
ILE	59	A	THR	428	B
CYS	31	A	LYS	436	B
ILE	67	A	TRP	396	B
TRP	30	A	PHE	440	B
ASP	60	A	GLU	431	B
PRO	75	A	HIS	438	B
TRP	30	A	SER	427	B
ASP	60	A	ALA	429	B
GLU	68	A	TRP	396	B
ILE	59	A	TRP	396	B
TRP	30	A	HIS	438	B
ASP	60	A	SER	427	B
SER	72	A	HIS	399	B
VAL	71	A	CYS	397	B
SER	72	A	CYS	400	B

12. Pyruvate dehydrogenase E1 component subunit alpha, somatic form, mitochondrial (UniProt KB: P08559):

BINDING AFFINITY AND K_D PREDICTION

The binding affinity (ΔG) and dissociation constant (K_d)predicted values are:

**Protein-protein complex**	**ΔG (kcal mol^{-1})**	**K_D (M) at 25.0 ℃**
HsTrx2-P08559	-11.4	4.5E-09

PREDICTION DETAILS

Number of Interfacial Contacts (ICs) per property:

ICs charged-charged:	17
ICs charged-polar:	17
ICs charged-apolar:	29
ICs polar-polar:	1
ICs polar-apolar:	14
ICs apolar-apolar:	10

Non-Interacting Surface (NIS) per property:

NIS charged:	33.23 %
NIS apolar:	39.94 %

Table of the ICs at the interface:

HsTrx2 residue	Number(#1)	Chain(#1)	P08559 residue	Number(#2)	Chain(#2)
ASP	61	A	ARG	44	B
ILE	5	A	GLN	51	B
PRO	9	A	HIS	261	B
PRO	9	A	GLN	51	B
ASP	61	A	LYS	48	B
ASP	64	A	ALA	49	B
ASP	7	A	LEU	47	B
THR	63	A	ALA	321	B
ASP	64	A	LYS	48	B
GLN	29	A	ARG	314	B
GLY	8	A	LYS	48	B
ASP	64	A	LEU	106	B
PRO	9	A	MET	265	B
ILE	67	A	THR	325	B
ASP	7	A	HIS	261	B
ASP	7	A	GLN	51	B
GLU	68	A	LEU	52	B
ASP	64	A	LEU	47	B
GLU	68	A	THR	325	B
ASP	64	A	ALA	321	B
GLN	6	A	LEU	47	B
PRO	9	A	LEU	47	B
THR	63	A	ILE	317	B
ASP	60	A	GLU	318	B
LEU	65	A	LEU	52	B
VAL	16	A	LYS	54	B
VAL	57	A	GLN	51	B
LEU	65	A	GLN	51	B
THR	63	A	ARG	314	B
THR	63	A	ASP	319	B

GLU	68	A	ILE	57	B
ASP	61	A	ARG	314	B
ASP	64	A	ILE	317	B
GLY	8	A	LYS	54	B
ILE	67	A	ALA	321	B
ILE	67	A	GLU	318	B
ASP	60	A	ARG	44	B
LEU	65	A	LYS	48	B
PRO	9	A	LYS	54	B
ASP	58	A	ARG	314	B
GLU	68	A	ALA	321	B
ASP	64	A	GLN	55	B
ASP	60	A	LYS	48	B
PHE	11	A	GLN	51	B
GLN	12	A	LYS	54	B
GLN	29	A	LYS	307	B
ILE	59	A	GLU	318	B
GLY	8	A	ASP	50	B
ALA	66	A	GLU	318	B
ASP	64	A	ALA	320	B
ILE	67	A	ILE	317	B
PRO	9	A	ASP	50	B
ASP	7	A	LYS	54	B
HIS	62	A	LEU	47	B
ASP	64	A	MET	45	B
GLU	68	A	GLN	55	B
GLY	8	A	ALA	49	B
LEU	65	A	LYS	54	B
ILE	59	A	LYS	48	B
PRO	9	A	LEU	64	B
ILE	67	A	ALA	320	B
ASP	7	A	ASP	50	B
HIS	62	A	GLN	51	B
ASP	64	A	GLN	322	B
ASP	61	A	LEU	47	B
THR	63	A	GLU	318	B
GLU	68	A	THR	110	B
TYR	69	A	GLN	55	B
THR	63	A	LYS	315	B
LEU	65	A	GLN	55	B
ASP	60	A	ARG	314	B
THR	63	A	GLN	51	B
GLY	8	A	LEU	47	B
ASP	7	A	ASP	66	B
ASP	61	A	GLN	51	B
HIS	62	A	ARG	44	B

ASP	64	A	GLU	318	B
HIS	62	A	LYS	48	B
PHE	11	A	LYS	54	B
ASP	64	A	LEU	52	B
GLY	8	A	LEU	52	B
ASP	64	A	GLN	51	B
ASP	60	A	LYS	315	B
GLY	8	A	GLN	51	B
ILE	67	A	GLN	322	B
ILE	67	A	ASP	319	B
THR	63	A	LYS	48	B
PHE	11	A	GLN	55	B

13. Pyruvate dehydrogenase E1 component subunit beta, mitochondrial (UniProt KB: P11177):

BINDING AFFINITY AND K_D PREDICTION

The binding affinity (ΔG) and dissociation constant (K_d)predicted values are:

Protein-protein complex	ΔG (kcal mol^{-1})	K_D (M) at 25.0 ℃
HsTrx2-P11177	-8.4	7.2E-07

PREDICTION DETAILS

Number of Interfacial Contacts (ICs) per property:

ICs charged-charged:	5
ICs charged-polar:	4
ICs charged-apolar:	11
ICs polar-polar:	2
ICs polar-apolar:	17
ICs apolar-apolar:	7

Non-Interacting Surface (NIS) per property:

NIS charged:	29.13 %
NIS apolar:	45.95 %

Table of the ICs at the interface:

HsTrx2 residue	Number(#1)	Chain(#1)	P11177 residue	Number(#2)	Chain(#2)
VAL	71	A	HIS	206	B
ILE	67	A	ASP	289	B
ILE	67	A	LYS	255	B
GLU	68	A	ASP	289	B
SER	72	A	HIS	206	B
ILE	67	A	PHE	287	B
ILE	67	A	THR	256	B

VAL	74	A	THR	205	B
THR	76	A	THR	205	B
VAL	71	A	THR	205	B
ILE	59	A	LYS	255	B
GLU	70	A	LEU	327	B
ALA	73	A	HIS	206	B
ILE	67	A	MET	254	B
SER	72	A	VAL	231	B
ASP	60	A	LYS	255	B
GLU	70	A	HIS	258	B
GLU	70	A	HIS	206	B
ILE	59	A	THR	205	B
ALA	73	A	THR	205	B
SER	72	A	CYS	233	B
VAL	74	A	GLU	232	B
VAL	71	A	THR	256	B
PHE	26	A	THR	205	B
SER	72	A	GLU	232	B
ALA	66	A	LYS	255	B
ALA	73	A	GLU	232	B
GLU	70	A	ASP	289	B
SER	72	A	THR	205	B
ILE	59	A	MET	254	B
ALA	73	A	GLY	230	B
SER	72	A	ILE	207	B
THR	63	A	ALA	251	B
ILE	67	A	ASN	257	B
THR	63	A	LYS	255	B
GLU	68	A	ASN	257	B
CYS	31	A	GLN	203	B
THR	63	A	PHE	287	B
VAL	74	A	GLY	204	B
THR	63	A	MET	254	B
SER	72	A	GLY	230	B
ILE	67	A	VAL	253	B
SER	72	A	GLY	204	B
ASP	64	A	PHE	287	B
ALA	73	A	VAL	231	B
ASP	64	A	MET	254	B

14. Creatine kinase S-type, mitochondrial (UniProt KB: P17540):

BINDING AFFINITY AND K_D PREDICTION

The binding affinity (ΔG) and dissociation constant (K_d)predicted values are:

Protein-protein complex	ΔG (kcal mol^{-1})	K_D (M) at 25.0 ℃

Protein-protein complex	ΔG (kcal mol^{-1})	K_D (M) at 25.0 ℃
HsTrx2-P17540	-7.0	7.9E-06

PREDICTION DETAILS

Number of Interfacial Contacts (ICs) per property:

ICs charged-charged:	3
ICs charged-polar:	4
ICs charged-apolar:	10
ICs polar-polar:	1
ICs polar-apolar:	9
ICs apolar-apolar:	12

Non-Interacting Surface (NIS) per property:

NIS charged:	34.11 %
NIS apolar:	39.65 %

Table of the ICs at the interface:

HsTrx2 residue	Number(#1)	Chain(#1)	P17540 residue	Number(#2)	Chain(#2)
GLY	38	A	ARG	46	B
TRP	30	A	GLY	240	B
TRP	30	A	THR	237	B
LYS	35	A	ASN	78	B
TRP	30	A	CYS	238	B
GLY	32	A	GLN	89	B
GLU	42	A	ARG	46	B
GLY	38	A	LEU	47	B
LYS	43	A	LEU	47	B
PRO	33	A	GLY	84	B
GLN	29	A	VAL	95	B
THR	2	A	LEU	47	B
GLY	32	A	GLY	84	B
LYS	35	A	TYR	73	B
LYS	35	A	THR	86	B
TRP	30	A	PRO	234	B
LYS	35	A	LEU	87	B
GLU	42	A	PHE	48	B
LYS	56	A	PHE	48	B
PRO	39	A	LEU	47	B
TRP	30	A	ASP	96	B
GLY	32	A	THR	86	B
ILE	36	A	THR	86	B
PRO	39	A	ARG	46	B
ILE	36	A	GLY	84	B

ILE	36	A	TYR	85	B
GLN	29	A	ASP	96	B
ALA	46	A	LEU	47	B
ILE	36	A	ASN	78	B
TRP	30	A	GLN	92	B
LYS	35	A	LEU	76	B
PRO	33	A	GLN	89	B
LYS	35	A	ARG	77	B
ILE	36	A	VAL	80	B
LYS	35	A	ASP	88	B
GLN	29	A	GLN	92	B
GLU	42	A	LEU	47	B
LYS	35	A	GLN	89	B
TRP	30	A	ALA	239	B

15. Cytochrome b-c1 complex subunit 2, mitochondrial (UniProt KB: P22695):

BINDING AFFINITY AND K_D PREDICTION

The binding affinity (ΔG) and dissociation constant (K_d)predicted values are:

Protein-protein complex	ΔG (kcal mol^{-1})	K_D (M) at 25.0 ℃
HsTrx2-P22695	-10.3	2.8E-08

PREDICTION DETAILS

Number of Interfacial Contacts (ICs) per property:

ICs charged-charged:	2
ICs charged-polar:	1
ICs charged-apolar:	39
ICs polar-polar:	0
ICs polar-apolar:	10
ICs apolar-apolar:	34

Non-Interacting Surface (NIS) per property:

NIS charged:	13.35 %
NIS apolar:	54.37 %

Table of the ICs at the interface:

HsTrx2 residue	Number(#1)	Chain(#1)	P22695 residue	Number(#2)	Chain(#2)
ASP	94	A	VAL	291	B
ILE	36	A	ILE	156	B
TRP	30	A	ARG	177	B
ASP	96	A	LEU	292	B
GLU	70	A	ILE	115	B
ILE	92	A	LEU	150	B

GLU	95	A	ILE	153	B
GLY	32	A	TRP	163	B
ILE	36	A	LEU	150	B
LYS	35	A	LEU	160	B
GLN	97	A	LEU	295	B
ASP	94	A	LEU	288	B
SER	72	A	ILE	188	B
SER	72	A	LEU	192	B
SER	72	A	LEU	185	B
ILE	36	A	LEU	160	B
CYS	31	A	ARG	177	B
LYS	88	A	LEU	121	B
SER	72	A	ILE	118	B
PHE	89	A	LEU	295	B
LEU	37	A	LEU	160	B
ASP	94	A	LYS	287	B
GLU	70	A	LEU	195	B
ARG	40	A	ILE	164	B
GLU	70	A	LEU	192	B
LYS	93	A	LEU	295	B
ASP	96	A	LEU	288	B
ASP	96	A	GLY	289	B
LYS	104	A	MET	303	B
VAL	90	A	LEU	121	B
GLU	70	A	ILE	118	B
ILE	36	A	GLY	157	B
ILE	36	A	VAL	161	B
ALA	100	A	LEU	292	B
GLY	32	A	ILE	164	B
CYS	31	A	TRP	163	B
VAL	74	A	PHE	181	B
ASP	94	A	GLY	289	B
ARG	40	A	LEU	160	B
ILE	36	A	ASP	159	B
GLU	95	A	LEU	288	B
LYS	35	A	TRP	163	B
GLU	70	A	GLU	111	B
LYS	35	A	ILE	164	B
PHE	89	A	LEU	299	B
ASP	87	A	ALA	302	B
ILE	36	A	ILE	153	B
ILE	36	A	TRP	163	B
VAL	90	A	ILE	118	B
ILE	92	A	ILE	164	B
ILE	36	A	ILE	164	B
GLU	95	A	PRO	154	B

LYS	93	A	LEU	299	B
PRO	39	A	ILE	156	B
ALA	73	A	PHE	181	B
ASP	94	A	ALA	152	B
GLY	32	A	LEU	160	B
GLU	70	A	PHE	199	B
GLU	95	A	ALA	152	B
ARG	40	A	ILE	156	B
PRO	39	A	LEU	160	B
LYS	88	A	LEU	299	B
PRO	33	A	LEU	150	B
ILE	36	A	GLN	162	B
ILE	92	A	LEU	295	B
ARG	40	A	LEU	150	B
LYS	88	A	ILE	118	B
SER	34	A	ILE	164	B
GLY	32	A	ARG	177	B
GLN	97	A	LEU	292	B
GLN	29	A	ARG	177	B
ILE	36	A	THR	147	B
ALA	73	A	ILE	188	B
SER	72	A	PHE	181	B
ARG	40	A	ILE	153	B
ASP	94	A	LEU	292	B
ALA	73	A	LEU	185	B
LEU	98	A	LEU	292	B
PRO	33	A	ILE	164	B
LYS	93	A	LEU	150	B
ALA	100	A	LEU	296	B
GLY	32	A	TRP	165	B
LYS	35	A	ILE	156	B
LEU	37	A	ILE	164	B
ASP	94	A	GLY	290	B
PHE	89	A	LEU	121	B

16. ATP synthase subunit alpha, mitochondrial (UniProt KB: P25705):

BINDING AFFINITY AND K_D PREDICTION

The binding affinity (ΔG) and dissociation constant (K_d)predicted values are:

**Protein-protein complex**	**ΔG (kcal mol^{-1})**	***K_D* (M) at 25.0 ℃**
HsTrx2-P25705	-10.7	1.5E-08

PREDICTION DETAILS

Number of Interfacial Contacts (ICs) per property:

ICs charged-charged:	9

ICs charged-polar:	9
ICs charged-apolar:	21
ICs polar-polar:	4
ICs polar-apolar:	22
ICs apolar-apolar:	20

Non-Interacting Surface (NIS) per property:

NIS charged:	30.95 %
NIS apolar:	43.58 %

Table of the ICs at the interface:

HsTrx2 residue	Number(#1)	Chain(#1)	P25705 residue	Number(#2)	Chain(#2)
ILE	67	A	ILE	81	B
ILE	67	A	ILE	125	B
ASP	64	A	ILE	77	B
ALA	66	A	ASP	122	B
SER	72	A	GLY	120	B
ALA	73	A	ALA	283	B
ILE	67	A	GLY	78	B
THR	63	A	ASP	79	B
ILE	67	A	LYS	123	B
GLU	70	A	ASP	122	B
ALA	73	A	ASP	281	B
GLU	68	A	LYS	123	B
GLN	29	A	LEU	326	B
SER	72	A	ALA	279	B
ASP	64	A	ASP	79	B
ILE	67	A	ASN	121	B
PRO	33	A	VAL	319	B
ILE	67	A	ILE	77	B
VAL	74	A	VAL	319	B
ILE	67	A	GLY	80	B
ALA	73	A	SER	280	B
ILE	92	A	ARG	214	B
ASP	60	A	ARG	329	B
TRP	30	A	GLN	323	B
TRP	30	A	LEU	327	B
GLN	29	A	ALA	336	B
ALA	73	A	THR	278	B
ALA	73	A	ALA	282	B
VAL	90	A	LYS	252	B
SER	72	A	ASP	281	B
SER	72	A	GLN	286	B

GLY	32	A	ARG	322	B
GLN	29	A	ARG	322	B
ASP	94	A	ARG	214	B
VAL	71	A	SER	280	B
THR	63	A	GLY	78	B
LYS	93	A	LYS	252	B
ILE	67	A	LEU	124	B
ASP	60	A	GLN	323	B
ASP	60	A	LEU	327	B
TRP	30	A	ASP	79	B
ALA	73	A	GLN	286	B
VAL	71	A	ASN	121	B
SER	72	A	SER	280	B
TRP	30	A	MET	324	B
VAL	71	A	ALA	282	B
ASP	64	A	GLY	78	B
ASP	60	A	ASP	79	B
ILE	92	A	LYS	252	B
VAL	74	A	GLN	323	B
SER	72	A	ASN	121	B
ILE	67	A	ASP	122	B
VAL	90	A	ALA	279	B
SER	72	A	ALA	282	B
GLU	70	A	LYS	123	B
TRP	30	A	ALA	336	B
GLU	68	A	ASP	122	B
GLN	97	A	ARG	214	B
SER	72	A	GLN	323	B
GLU	70	A	SER	280	B
TRP	30	A	ARG	322	B
TRP	30	A	VAL	319	B
ILE	59	A	GLN	323	B
ILE	59	A	LEU	327	B
ILE	67	A	ASP	79	B
GLU	70	A	ASN	121	B
LYS	88	A	SER	280	B
ALA	73	A	GLN	323	B
CYS	31	A	ARG	322	B
THR	76	A	ALA	279	B
CYS	31	A	VAL	319	B
TRP	30	A	LEU	326	B
ILE	59	A	ASP	79	B
ALA	66	A	ASP	79	B
ASP	60	A	LEU	326	B
VAL	90	A	SER	280	B
THR	63	A	ILE	77	B

TYR	69	A	LYS	123	B
THR	63	A	GLY	80	B
GLN	97	A	LYS	252	B
TRP	30	A	GLU	335	B
ALA	73	A	ALA	279	B
SER	72	A	ALA	283	B
GLU	70	A	LEU	124	B
THR	63	A	LEU	327	B

17. Peroxiredoxin-6 (UniProt KB: P30041):

BINDING AFFINITY AND K$_D$ PREDICTION

The binding affinity (ΔG) and dissociation constant (K$_d$)predicted values are:

Protein-protein complex	ΔG (kcal mol^{-1})	K_D (M) at 25.0 °C
HsTrx2-P30041	-9.1	2.1E-07

PREDICTION DETAILS

Number of Interfacial Contacts (ICs) per property:

ICs charged-charged:	7
ICs charged-polar:	7
ICs charged-apolar:	29
ICs polar-polar:	0
ICs polar-apolar:	11
ICs apolar-apolar:	26

Non-Interacting Surface (NIS) per property:

NIS charged:	35.42 %
NIS apolar:	42.92 %

Table of the ICs at the interface:

HsTrx2 residue	Number(#1)	Chain(#1)	P30041 residue	Number(#2)	Chain(#2)
GLU	99	A	VAL	179	B
LYS	104	A	VAL	163	B
GLY	107	A	ASP	180	B
VAL	90	A	LEU	5	B
GLU	99	A	ALA	176	B
ILE	92	A	LEU	7	B
GLU	99	A	THR	177	B
VAL	90	A	LEU	6	B
PHE	89	A	SER	146	B
VAL	90	A	SER	146	B
LYS	103	A	ASP	180	B
ILE	106	A	ASP	180	B

ALA	73	A	GLY	4	B
LYS	93	A	SER	146	B
VAL	85	A	LEU	148	B
PHE	89	A	LEU	148	B
GLY	107	A	VAL	179	B
LYS	43	A	LYS	215	B
ASP	87	A	LEU	148	B
SER	72	A	GLY	115	B
GLN	97	A	LYS	144	B
VAL	90	A	LEU	7	B
ASP	87	A	TYR	149	B
VAL	86	A	TYR	149	B
GLN	97	A	LEU	145	B
LYS	104	A	GLU	159	B
LYS	88	A	SER	146	B
ILE	92	A	LEU	6	B
ILE	106	A	VAL	179	B
ASP	87	A	GLU	159	B
VAL	85	A	THR	153	B
LEU	78	A	LEU	148	B
LYS	88	A	LEU	148	B
LYS	103	A	THR	177	B
HIS	49	A	LEU	211	B
GLY	107	A	TRP	181	B
GLN	97	A	LEU	167	B
GLN	97	A	LEU	7	B
VAL	90	A	LEU	143	B
ILE	92	A	GLY	8	B
VAL	90	A	GLY	4	B
GLN	48	A	VAL	179	B
VAL	90	A	LEU	148	B
LYS	103	A	TRP	181	B
LYS	104	A	ARG	162	B
LYS	104	A	SER	166	B
GLY	91	A	LEU	7	B
LYS	104	A	ASP	180	B
VAL	85	A	TYR	149	B
ASP	94	A	LEU	7	B
ASP	96	A	ALA	176	B
LYS	103	A	VAL	179	B
VAL	74	A	LEU	6	B
PHE	89	A	ILE	147	B
GLU	70	A	PRO	150	B
GLY	91	A	LEU	5	B
ASP	96	A	VAL	175	B
GLU	99	A	PRO	178	B

ASP	87	A	ILE	147	B
ALA	100	A	SER	166	B
THR	76	A	LEU	148	B
LYS	88	A	TYR	149	B
GLY	91	A	LEU	6	B
ASP	96	A	LEU	190	B
LYS	51	A	VAL	179	B
LYS	93	A	LYS	144	B
ASP	87	A	LEU	145	B
LYS	47	A	TYR	217	B
LYS	93	A	LEU	145	B
LYS	88	A	ILE	147	B
ASP	87	A	SER	146	B
GLU	70	A	PRO	119	B
HIS	49	A	TYR	217	B
VAL	85	A	PRO	150	B
ALA	100	A	THR	177	B
LYS	103	A	PRO	178	B
LYS	93	A	LEU	7	B
LEU	102	A	VAL	179	B
LYS	104	A	VAL	179	B
PRO	75	A	LEU	6	B

18. Peroxiredoxin-5 (UniProt KB: P30044):

BINDING AFFINITY AND K_D PREDICTION

The binding affinity (ΔG) and dissociation constant (K_d)predicted values are:

Protein-protein complex	ΔG (kcal mol^{-1})	K_D (M) at 25.0 °C
HsTrx2-P30044	-6.9	9.1E-06

PREDICTION DETAILS

Number of Interfacial Contacts (ICs) per property:

ICs charged-charged:	11
ICs charged-polar:	11
ICs charged-apolar:	21
ICs polar-polar:	3
ICs polar-apolar:	6
ICs apolar-apolar:	10

Non-Interacting Surface (NIS) per property:

NIS charged:	31.89 %
NIS apolar:	45.95 %

Table of the ICs at the interface:

HsTrx2 residue	Number(#1)	Chain(#1)	P30044 residue	Number(#2)	Chain(#2)
THR	63	A	GLY	31	B
GLU	68	A	LYS	93	B
ASP	61	A	GLY	31	B
ILE	67	A	GLU	91	B
ASP	64	A	LYS	63	B
SER	72	A	ASN	21	B
GLU	70	A	ARG	95	B
GLU	68	A	GLU	91	B
ILE	67	A	GLY	92	B
THR	63	A	LYS	32	B
ILE	67	A	ALA	90	B
GLU	68	A	GLY	92	B
THR	63	A	VAL	70	B
TRP	30	A	LEU	28	B
HIS	62	A	GLN	68	B
THR	63	A	VAL	69	B
THR	63	A	GLN	68	B
LYS	88	A	ASN	21	B
ILE	67	A	GLU	16	B
ASP	61	A	GLN	68	B
TRP	30	A	GLY	31	B
GLU	70	A	GLY	17	B
VAL	74	A	GLU	27	B
ASP	64	A	VAL	69	B
TRP	30	A	LYS	30	B
ASP	64	A	GLN	68	B
SER	72	A	LYS	22	B
ALA	66	A	GLU	16	B
THR	63	A	LYS	33	B
ASP	60	A	GLY	31	B
ASP	60	A	LYS	30	B
ALA	73	A	LYS	22	B
ILE	67	A	VAL	70	B
ILE	67	A	ARG	95	B
GLU	70	A	GLU	91	B
ASP	64	A	GLY	92	B
ASP	60	A	LYS	32	B
GLU	68	A	ARG	95	B
VAL	71	A	GLU	16	B
ILE	59	A	ARG	95	B
ALA	66	A	ARG	95	B
SER	72	A	GLU	16	B
ALA	73	A	VAL	23	B
ILE	67	A	VAL	94	B

ASP	60	A	GLN	68	B
THR	63	A	LEU	28	B
TRP	30	A	GLU	27	B
VAL	71	A	ARG	95	B
GLU	70	A	GLU	16	B
TYR	69	A	GLU	91	B
VAL	71	A	VAL	23	B
ILE	59	A	LYS	32	B
GLU	70	A	ARG	86	B
THR	63	A	ARG	95	B
ILE	67	A	VAL	69	B
SER	72	A	ARG	95	B
ILE	67	A	LYS	93	B
SER	72	A	VAL	23	B
ILE	67	A	GLN	68	B
ASP	60	A	GLU	27	B
TYR	69	A	GLY	92	B
SER	72	A	ASN	24	B

19. Thioredoxin-dependent peroxide reductase, mitochondrial (Peroxiredoxin-3) (UniProt KB: P30048):

BINDING AFFINITY AND K_D PREDICTION

The binding affinity (ΔG) and dissociation constant (K_d)predicted values are:

**Protein-protein complex**	**ΔG (kcal mol^{-1})**	***K_D* (M) at 25.0 °C**
HsTrx2-P30048	-7.9	1.5E-06

PREDICTION DETAILS

Number of Interfacial Contacts (ICs) per property:

ICs charged-charged:	0
ICs charged-polar:	7
ICs charged-apolar:	8
ICs polar-polar:	3
ICs polar-apolar:	17
ICs apolar-apolar:	27

Non-Interacting Surface (NIS) per property:

NIS charged:	29.09 %
NIS apolar:	43.18 %

Table of the ICs at the interface:

HsTrx2 residue	Number(#1)	Chain(#1)	P30048 residue	Number(#2)	Chain(#2)
VAL	90	A	ASN	89	B

ALA	73	A	THR	44	B
ILE	67	A	SER	78	B
TRP	30	A	CYS	47	B
VAL	74	A	PHE	43	B
VAL	71	A	PHE	43	B
GLY	32	A	PRO	143	B
VAL	74	A	PHE	45	B
CYS	31	A	PRO	143	B
ASP	61	A	SER	118	B
TRP	30	A	LEU	41	B
SER	72	A	PHE	43	B
TRP	30	A	LEU	142	B
TRP	30	A	PRO	40	B
ILE	67	A	ASP	74	B
SER	72	A	TRP	82	B
VAL	71	A	ASP	42	B
ASP	60	A	LEU	41	B
SER	72	A	ASN	89	B
ALA	73	A	PHE	45	B
CYS	31	A	LEU	142	B
SER	72	A	PRO	48	B
TRP	30	A	THR	44	B
SER	72	A	ASP	42	B
ASP	60	A	PRO	40	B
GLU	70	A	PHE	43	B
ALA	73	A	ASN	89	B
TRP	30	A	PRO	143	B
TRP	30	A	LEU	120	B
SER	72	A	GLY	91	B
ASP	60	A	THR	44	B
PRO	33	A	PHE	45	B
GLU	70	A	ASN	89	B
ASP	60	A	LEU	120	B
TRP	30	A	VAL	46	B
SER	72	A	GLY	90	B
ILE	59	A	LEU	41	B
SER	72	A	PHE	45	B
CYS	31	A	VAL	46	B
ASP	60	A	SER	118	B
ALA	73	A	GLY	90	B
TRP	30	A	VAL	144	B
ASP	60	A	LEU	142	B
CYS	31	A	PHE	45	B
ILE	59	A	THR	44	B
ALA	73	A	PRO	48	B
SER	72	A	THR	85	B

GLN	29	A	PRO	143	B
THR	63	A	LEU	41	B
ILE	67	A	PHE	43	B
TRP	30	A	ARG	123	B
PRO	75	A	PHE	45	B
LYS	88	A	ASN	89	B
VAL	74	A	THR	44	B
VAL	71	A	THR	44	B
TRP	30	A	PHE	45	B
ILE	59	A	PHE	43	B
ALA	66	A	PHE	43	B
THR	63	A	ASP	74	B
ALA	73	A	PHE	43	B
SER	72	A	THR	44	B
GLN	29	A	LEU	142	B

20. Cytochrome b-c1 complex subunit 1, mitochondrial (UniProt KB: P31930):

BINDING AFFINITY AND K_D PREDICTION

The binding affinity (ΔG) and dissociation constant (K_d)predicted values are:

Protein-protein complex	ΔG (kcal mol^{-1})	K_D (M) at 25.0 ℃
HsTrx2-P31930	-7.9	1.5E-06

PREDICTION DETAILS

Number of Interfacial Contacts (ICs) per property:

ICs charged-charged:	7
ICs charged-polar:	6
ICs charged-apolar:	21
ICs polar-polar:	0
ICs polar-apolar:	2
ICs apolar-apolar:	15

Non-Interacting Surface (NIS) per property:

NIS charged:	25.06 %
NIS apolar:	41.52 %

Table of the ICs at the interface:

HsTrx2 residue	Number(#1)	Chain(#1)	P31930 residue	Number(#2)	Chain(#2)
ILE	36	A	THR	345	B
ARG	40	A	HIS	446	B
ILE	92	A	ASP	349	B
ILE	36	A	ASP	349	B
GLY	32	A	LYS	352	B

PRO	33	A	PRO	448	B
CYS	31	A	LYS	352	B
TRP	30	A	LEU	453	B
GLY	32	A	GLU	452	B
CYS	31	A	GLU	452	B
GLY	32	A	HIS	446	B
CYS	31	A	LEU	453	B
ILE	36	A	THR	447	B
LYS	35	A	GLU	452	B
LYS	93	A	ALA	316	B
ILE	36	A	ALA	346	B
GLY	32	A	GLY	348	B
TRP	30	A	LYS	352	B
ILE	36	A	HIS	446	B
ILE	36	A	ALA	344	B
ASP	94	A	THR	345	B
ILE	92	A	ALA	316	B
ASP	94	A	GLN	318	B
ASP	94	A	ALA	346	B
LEU	37	A	ASP	349	B
TRP	30	A	GLU	452	B
ASP	94	A	ASP	349	B
ILE	36	A	GLY	348	B
ASP	94	A	ALA	316	B
VAL	74	A	LYS	352	B
ARG	40	A	THR	345	B
ASP	96	A	GLN	318	B
ARG	40	A	ALA	346	B
GLY	32	A	PRO	448	B
LYS	35	A	HIS	446	B
PRO	33	A	LEU	453	B
CYS	31	A	PRO	448	B
PRO	33	A	ASP	349	B
ARG	40	A	ASP	349	B
LYS	35	A	PRO	448	B
LYS	35	A	PHE	449	B
PRO	33	A	GLY	348	B
ILE	36	A	PRO	448	B
GLN	29	A	GLU	452	B
GLY	32	A	LEU	453	B
TRP	30	A	ASP	451	B
LYS	93	A	LYS	315	B
PRO	33	A	LYS	352	B
ASP	94	A	THR	317	B
LYS	93	A	ASP	349	B
ILE	36	A	ALA	347	B

21. Peroxiredoxin-2 (UniProt KB: P32119):

BINDING AFFINITY AND K_D PREDICTION

The binding affinity (ΔG) and dissociation constant (K_d)predicted values are:

Protein-protein complex	ΔG (kcal mol^{-1})	K_D (M) at 25.0 ℃
HsTrx2-P32119	-13.1	2.3E-10

PREDICTION DETAILS

Number of Interfacial Contacts (ICs) per property:

ICs charged-charged:	7
ICs charged-polar:	9
ICs charged-apolar:	39
ICs polar-polar:	3
ICs polar-apolar:	25
ICs apolar-apolar:	34

Non Interacting Surface (NIS) per property:

NIS charged:	34.39 %
NIS apolar:	41.18 %

Table of the ICs at the interface:

HsTrx2 residue	Number(#1)	Chain(#1)	P32119 residue	Number(#2)	Chain(#2)
VAL	71	A	GLY	4	B
GLU	68	A	ASP	145	B
ALA	100	A	GLY	175	B
SER	72	A	VAL	143	B
GLN	97	A	PHE	162	B
LYS	104	A	TRP	176	B
SER	72	A	GLY	4	B
ASP	87	A	GLN	140	B
SER	72	A	VAL	117	B
VAL	71	A	ALA	2	B
LYS	88	A	ILE	141	B
ALA	100	A	PRO	173	B
VAL	90	A	ALA	6	B
VAL	85	A	VAL	143	B
VAL	71	A	GLY	116	B
LEU	102	A	ALA	174	B
GLU	99	A	ALA	174	B
ALA	73	A	GLY	4	B
SER	72	A	ALA	2	B
VAL	85	A	VAL	148	B

ASP	96	A	VAL	171	B
LYS	104	A	GLY	175	B
PRO	75	A	ILE	8	B
VAL	77	A	ILE	141	B
GLU	70	A	VAL	143	B
LYS	88	A	GLN	140	B
SER	72	A	TYR	115	B
GLU	70	A	THR	120	B
ILE	67	A	ASP	121	B
ASP	84	A	PRO	147	B
GLN	97	A	VAL	171	B
VAL	90	A	ARG	7	B
ASP	96	A	CYS	172	B
GLU	70	A	VAL	117	B
PHE	89	A	ILE	141	B
VAL	90	A	ILE	141	B
SER	72	A	TYR	126	B
LYS	104	A	PRO	173	B
GLU	99	A	PRO	173	B
VAL	85	A	THR	142	B
LEU	78	A	VAL	143	B
LYS	88	A	VAL	143	B
GLN	97	A	CYS	172	B
VAL	90	A	ILE	8	B
ILE	67	A	LYS	119	B
GLU	70	A	LEU	118	B
VAL	85	A	ASN	144	B
LYS	93	A	ILE	8	B
GLU	70	A	GLY	116	B
PHE	89	A	GLN	140	B
VAL	90	A	GLN	140	B
VAL	86	A	ASN	144	B
LYS	103	A	ALA	174	B
LYS	104	A	LEU	158	B
LYS	93	A	ARG	139	B
VAL	74	A	ARG	7	B
GLU	70	A	TYR	126	B
THR	76	A	ILE	141	B
ASP	87	A	LEU	158	B
LYS	88	A	THR	142	B
ALA	73	A	ALA	6	B
ILE	59	A	SER	3	B
ILE	92	A	ILE	8	B
GLN	97	A	THR	165	B
ALA	73	A	SER	3	B
LYS	88	A	TYR	126	B

TYR	69	A	VAL	143	B
VAL	85	A	GLY	149	B
PHE	101	A	ALA	174	B
SER	72	A	GLY	116	B
ASP	96	A	PHE	194	B
LYS	93	A	PHE	162	B
LYS	88	A	LEU	158	B
ALA	100	A	CYS	172	B
ALA	73	A	ARG	7	B
VAL	86	A	GLU	154	B
VAL	74	A	ALA	6	B
ASP	96	A	ALA	174	B
SER	72	A	ASN	5	B
ALA	73	A	ILE	8	B
VAL	71	A	SER	3	B
ASP	96	A	PRO	185	B
VAL	90	A	ARG	139	B
ILE	67	A	THR	120	B
SER	72	A	ALA	6	B
VAL	85	A	ASP	145	B
GLY	83	A	VAL	148	B
ASP	96	A	SER	190	B
ALA	73	A	ASN	5	B
SER	72	A	SER	3	B
VAL	74	A	ALA	2	B
THR	76	A	ALA	2	B
ASP	96	A	PRO	173	B
LYS	103	A	GLY	175	B
ASP	84	A	VAL	148	B
ILE	67	A	LEU	118	B
SER	72	A	PHE	130	B
ASP	96	A	ILE	183	B
LYS	104	A	ALA	174	B
GLU	70	A	LYS	119	B
LYS	104	A	ARG	157	B
LYS	88	A	ASN	144	B
SER	72	A	ILE	141	B
ASP	96	A	LYS	184	B
LYS	104	A	GLU	154	B
ILE	59	A	ALA	2	B
VAL	86	A	VAL	148	B
ALA	100	A	TRP	176	B
GLN	97	A	ARG	139	B
ALA	66	A	ALA	2	B
GLY	91	A	ILE	8	B
ASP	87	A	GLU	154	B

ALA	73	A	ALA	2	B
ASP	84	A	ASN	144	B
ALA	100	A	ALA	174	B
ASP	87	A	THR	142	B
ASP	94	A	VAL	171	B

22. Stress-70 protein, mitochondrial (UniProt KB: P38646):

BINDING AFFINITY AND K_D PREDICTION

The binding affinity (ΔG) and dissociation constant (K_d)predicted values are:

**Protein-protein complex**	**ΔG (kcal mol^{-1})**	***K_D* (M) at 25.0 ℃**
HsTrx2-P38646	-7.4	3.5E-06

PREDICTION DETAILS

Number of Interfacial Contacts (ICs) per property:

ICs charged-charged:	7
ICs charged-polar:	2
ICs charged-apolar:	15
ICs polar-polar:	0
ICs polar-apolar:	5
ICs apolar-apolar:	5

Non-Interacting Surface (NIS) per property:

NIS charged:	34.44 %
NIS apolar:	37.75 %

Table of the ICs at the interface:

HsTrx2 residue	Number(#1)	Chain(#1)	P38646 residue	Number(#2)	Chain(#2)
LYS	51	A	LEU	450	B
GLN	48	A	GLY	451	B
LYS	103	A	ARG	513	B
GLN	48	A	MET	493	B
HIS	49	A	GLY	451	B
HIS	49	A	MET	493	B
PHE	101	A	ARG	513	B
LYS	51	A	GLY	451	B
LYS	47	A	VAL	453	B
ASP	96	A	ARG	513	B
GLY	107	A	ALA	476	B
LYS	51	A	GLU	448	B
LYS	51	A	THR	449	B
GLN	97	A	ARG	513	B
GLY	50	A	GLY	451	B

LYS	103	A	ALA	476	B
GLY	50	A	MET	493	B
LEU	98	A	ARG	513	B
GLU	95	A	ARG	513	B
HIS	49	A	GLU	492	B
GLN	48	A	GLY	452	B
GLN	48	A	VAL	453	B
LYS	47	A	LEU	450	B
GLN	48	A	LEU	450	B
ALA	100	A	ARG	513	B
GLY	107	A	LEU	450	B
HIS	49	A	GLY	452	B
HIS	49	A	VAL	453	B
LYS	47	A	GLY	451	B
LYS	103	A	ASP	477	B
ILE	106	A	LEU	450	B
GLU	99	A	ARG	513	B
LYS	51	A	GLY	452	B
LYS	103	A	ALA	475	B

23. Trifunctional enzyme subunit alpha, mitochondrial (UniProt KB: P40939):

BINDING AFFINITY AND K_D PREDICTION

The binding affinity (ΔG) and dissociation constant (K_d)predicted values are:

Protein-protein complex	ΔG (kcal mol^{-1})	K_D (M) at 25.0 ℃
HsTrx2-P40939	-9.2	1.6E-07

PREDICTION DETAILS

Number of Interfacial Contacts (ICs) per property:

ICs charged-charged:	6
ICs charged-polar:	15
ICs charged-apolar:	13
ICs polar-polar:	1
ICs polar-apolar:	19
ICs apolar-apolar:	16

Non-Interacting Surface (NIS) per property:

NIS charged:	28.97 %
NIS apolar:	46.43 %

Table of the ICs at the interface:

HsTrx2 residue	Number(#1)	Chain(#1)	P40939 residue	Number(#2)	Chain(#2)
VAL	86	A	LEU	433	B

ASP	87	A	GLN	432	B
ASP	96	A	LYS	440	B
LYS	103	A	GLN	436	B
SER	72	A	ASP	423	B
GLY	91	A	ASN	428	B
ILE	92	A	SER	427	B
GLN	97	A	LYS	440	B
ASP	87	A	GLY	431	B
PHE	101	A	GLN	436	B
LYS	88	A	GLN	432	B
SER	72	A	ARG	422	B
PRO	33	A	PHE	420	B
ASP	94	A	LYS	440	B
SER	72	A	PHE	426	B
LYS	104	A	TYR	435	B
VAL	85	A	LEU	394	B
ALA	73	A	PHE	426	B
ASP	87	A	LEU	394	B
GLN	97	A	GLN	436	B
ASP	87	A	LEU	433	B
PHE	89	A	THR	430	B
GLY	32	A	PHE	420	B
LYS	93	A	ILE	388	B
SER	72	A	PHE	405	B
CYS	31	A	PHE	420	B
LYS	93	A	LYS	386	B
TRP	30	A	ASP	423	B
VAL	74	A	SER	427	B
LYS	93	A	THR	430	B
PHE	89	A	GLY	431	B
LYS	104	A	GLY	431	B
LYS	104	A	GLN	436	B
VAL	90	A	ASP	423	B
VAL	90	A	ASN	428	B
PHE	89	A	LEU	429	B
PHE	101	A	GLY	437	B
LYS	93	A	ASN	428	B
GLY	91	A	SER	427	B
ILE	92	A	LYS	386	B
LYS	88	A	THR	430	B
ALA	73	A	SER	427	B
VAL	90	A	PHE	426	B
LYS	88	A	GLY	431	B
ALA	100	A	GLN	436	B
ILE	92	A	ASN	428	B
VAL	74	A	ASP	423	B

LYS	104	A	LEU	433	B
TRP	30	A	PHE	420	B
GLN	97	A	GLY	437	B
VAL	90	A	THR	430	B
GLN	97	A	HIS	363	B
LEU	105	A	GLN	436	B
VAL	90	A	GLY	431	B
GLU	99	A	GLN	436	B
PRO	75	A	SER	427	B
LYS	93	A	THR	387	B
ALA	73	A	ASP	423	B
VAL	90	A	LEU	429	B
ALA	100	A	PHE	438	B
ASP	94	A	HIS	363	B
GLN	97	A	ILE	388	B
LYS	104	A	ASP	434	B
GLN	97	A	LYS	386	B
VAL	90	A	SER	427	B
ALA	100	A	GLY	437	B
GLY	91	A	THR	430	B
VAL	86	A	LEU	394	B
ASP	94	A	LYS	386	B
LYS	104	A	GLN	432	B

24. Isocitrate dehydrogenase [NADP], mitochondrial (UniProt KB: P48735):

BINDING AFFINITY AND K_D PREDICTION

The binding affinity (ΔG) and dissociation constant (K_d)predicted values are:

Protein-protein complex	ΔG (kcal mol^{-1})	K_D (M) at 25.0 °C
HsTrx2-P48735	-9.4	1.3E-07

PREDICTION DETAILS

Number of Interfacial Contacts (ICs) per property:

ICs charged-charged:	18
ICs charged-polar:	12
ICs charged-apolar:	26
ICs polar-polar:	2
ICs polar-apolar:	5
ICs apolar-apolar:	8

Non-Interacting Surface (NIS) per property:

NIS charged:	31.58 %
NIS apolar:	38.68 %

Table of the ICs at the interface:

HsTrx2 residue	Number(#1)	Chain(#1)	P48735 residue	Number(#2)	Chain(#2)
PRO	33	A	LYS	299	B
LYS	93	A	GLN	267	B
ASP	94	A	ASN	252	B
PRO	33	A	ASP	292	B
ARG	40	A	ASP	292	B
VAL	90	A	TYR	247	B
ILE	92	A	HIS	287	B
ASP	94	A	ARG	288	B
GLN	97	A	ASP	264	B
GLU	95	A	ASN	252	B
VAL	90	A	GLU	286	B
ASP	96	A	GLY	260	B
ASP	94	A	HIS	287	B
GLY	32	A	LYS	299	B
LYS	93	A	LYS	263	B
GLY	32	A	ASP	292	B
PRO	33	A	ARG	288	B
ILE	92	A	GLN	296	B
ASP	94	A	THR	253	B
ASP	96	A	LEU	255	B
PHE	89	A	TYR	285	B
VAL	90	A	TYR	285	B
GLU	95	A	THR	253	B
GLY	91	A	TYR	247	B
ASP	94	A	LYS	251	B
GLY	91	A	ARG	288	B
ASP	96	A	LYS	256	B
ILE	92	A	ASP	292	B
ILE	36	A	ASP	292	B
GLY	91	A	GLU	286	B
GLN	97	A	HIS	287	B
ASP	94	A	LEU	289	B
ASP	94	A	GLU	286	B
VAL	90	A	TRP	284	B
LYS	104	A	ASP	271	B
ARG	40	A	THR	253	B
TRP	30	A	LYS	299	B
GLY	91	A	HIS	287	B
GLN	97	A	GLN	267	B
CYS	31	A	LYS	299	B
PRO	33	A	GLN	296	B
ASP	94	A	ILE	254	B

GLU	95	A	HIS	287	B
GLU	95	A	LYS	256	B
ARG	40	A	LEU	289	B
ASP	96	A	LYS	263	B
LYS	104	A	GLN	267	B
PRO	75	A	ARG	288	B
GLN	97	A	LYS	263	B
GLY	91	A	GLN	296	B
ASP	96	A	ARG	261	B
ASP	94	A	LYS	263	B
ILE	92	A	MET	293	B
ASP	94	A	THR	250	B
PHE	89	A	GLU	286	B
LEU	37	A	ASP	292	B
ALA	100	A	GLN	267	B
LYS	93	A	ARG	288	B
LYS	93	A	LEU	289	B
ASP	96	A	ASP	259	B
GLU	95	A	LYS	263	B
LYS	93	A	GLU	286	B
GLN	97	A	TYR	285	B
ASP	96	A	ASN	252	B
LYS	93	A	HIS	287	B
ASP	96	A	ALA	257	B
GLN	97	A	ASN	252	B
ILE	92	A	ARG	288	B
ILE	92	A	LEU	289	B
ILE	36	A	LEU	289	B
ILE	92	A	GLU	286	B

Ribose 5-phosphate isomerase A (UniProt KB: P49247):

Binding affinity and K_D prediction

The binding affinity (ΔG) and dissociation constant (K_d)predicted values are:

Protein-protein complex	ΔG (kcal mol^{-1})	K_D (M) at 25.0 ℃
HsTrx2-P49247	-9.3	1.5E-07

Prediction details

Number of Interfacial Contacts (ICs) per property:

ICs charged-charged:	6
ICs charged-polar:	8
ICs charged-apolar:	23
ICs polar-polar:	6
ICs polar-apolar:	16
ICs apolar-apolar:	19

Non-Interacting Surface (NIS) per property:

NIS charged:	26.61 %
NIS apolar:	44.34 %

Table of the ICs at the interface:

HsTrx2 residue	Number(#1)	Chain(#1)	P49247 residue	Number(#2)	Chain(#2)
ALA	28	A	PHE	7	B
GLU	42	A	GLN	2	B
THR	2	A	GLN	2	B
LYS	35	A	PHE	7	B
GLY	32	A	PRO	153	B
THR	2	A	MET	1	B
ASN	4	A	PRO	4	B
VAL	90	A	LYS	276	B
VAL	90	A	GLY	187	B
CYS	31	A	TYR	188	B
ASP	94	A	ARG	191	B
VAL	71	A	MET	277	B
GLU	70	A	VAL	281	B
GLU	42	A	PRO	4	B
VAL	86	A	THR	273	B
LYS	56	A	PRO	4	B
LYS	88	A	LYS	276	B
ILE	92	A	TYR	188	B
GLN	97	A	ASN	289	B
GLN	97	A	GLY	187	B
ASP	94	A	ALA	189	B
GLU	70	A	PRO	279	B
PRO	75	A	TYR	188	B
VAL	90	A	ASP	283	B
ILE	36	A	GLU	154	B
LYS	93	A	MET	290	B
SER	72	A	VAL	281	B
GLY	91	A	GLY	187	B
GLU	70	A	MET	277	B
SER	72	A	GLY	280	B
SER	34	A	TYR	188	B
ILE	92	A	PRO	153	B
VAL	85	A	THR	273	B
LEU	78	A	MET	277	B
GLN	29	A	THR	9	B
ASP	87	A	THR	273	B
THR	2	A	ARG	3	B

GLU	42	A	ARG	3	B
GLN	29	A	PHE	7	B
GLN	97	A	ALA	189	B
HIS	27	A	GLY	5	B
SER	72	A	ILE	278	B
SER	72	A	VAL	282	B
HIS	27	A	PRO	6	B
PHE	3	A	ARG	3	B
VAL	74	A	TYR	188	B
GLU	70	A	GLY	280	B
ASN	4	A	GLY	5	B
VAL	85	A	MET	277	B
ARG	40	A	GLU	154	B
LYS	88	A	THR	273	B
ASP	61	A	PRO	6	B
SER	72	A	LYS	276	B
ASN	4	A	PRO	6	B
TYR	69	A	MET	277	B
GLY	91	A	TYR	188	B
GLN	97	A	SER	190	B
GLN	29	A	PRO	6	B
GLU	70	A	ILE	278	B
LYS	104	A	ASN	289	B
ASP	58	A	PRO	6	B
LYS	93	A	ILE	288	B
GLU	42	A	GLY	5	B
PRO	33	A	PRO	153	B
LYS	93	A	ASN	289	B
LYS	56	A	GLY	5	B
TRP	30	A	PHE	7	B
LYS	93	A	GLY	187	B
LYS	88	A	MET	277	B
ASP	94	A	SER	190	B
GLN	97	A	MET	290	B
GLU	70	A	LYS	276	B
LYS	56	A	ARG	3	B
CYS	31	A	PHE	7	B
PRO	33	A	TYR	188	B
GLN	29	A	SER	8	B
ILE	92	A	GLY	187	B
THR	1	A	GLN	2	B

25. Elongation factor Tu, mitochondrial, EF-Tu (UniProt KB: P49411):

Binding affinity and K_D prediction

The binding affinity (ΔG) and dissociation constant (K_d)predicted values are:

Protein-protein complex	ΔG (kcal mol^{-1})	K_D (M) at 25.0 ℃
HsTrx2-P49411	-6.3	2.5E-05

PREDICTION DETAILS

Number of Interfacial Contacts (ICs) per property:

ICs charged-charged:	2
ICs charged-polar:	3
ICs charged-apolar:	7
ICs polar-polar:	2
ICs polar-apolar:	12
ICs apolar-apolar:	16

Non-Interacting Surface (NIS) per property:

NIS charged:	33.50 %
NIS apolar:	44.17 %

Table of the ICs at the interface:

HsTrx2 residue	Number(#1)	Chain(#1)	P49411 residue	Number(#2)	Chain(#2)
ASP	60	A	ASN	438	B
VAL	74	A	LEU	440	B
GLY	32	A	THR	443	B
ASP	94	A	GLU	444	B
PRO	33	A	GLU	446	B
CYS	31	A	THR	439	B
GLY	32	A	ALA	441	B
VAL	90	A	LYS	347	B
CYS	31	A	ALA	441	B
ALA	73	A	LEU	440	B
VAL	90	A	HIS	349	B
ILE	92	A	ALA	441	B
ILE	59	A	ASN	438	B
PRO	75	A	ALA	441	B
PRO	33	A	LEU	440	B
SER	72	A	LYS	347	B
TRP	30	A	GLU	353	B
ALA	73	A	THR	439	B
VAL	74	A	HIS	349	B
SER	72	A	LEU	416	B
LYS	93	A	GLU	444	B
SER	34	A	ALA	441	B
ILE	92	A	THR	443	B
GLY	32	A	LEU	440	B

VAL	74	A	THR	439	B
CYS	31	A	LEU	440	B
ILE	92	A	GLU	444	B
TRP	30	A	ASN	407	B
VAL	74	A	ALA	441	B
ALA	73	A	HIS	349	B
SER	72	A	THR	439	B
ILE	92	A	MET	442	B
GLY	91	A	ALA	441	B
PRO	75	A	LEU	440	B
SER	72	A	SER	345	B
SER	72	A	ILE	346	B
TRP	30	A	LEU	440	B
PRO	33	A	THR	443	B
SER	72	A	HIS	349	B
TRP	30	A	ASN	438	B
PRO	33	A	ALA	441	B
PRO	33	A	MET	442	B

26. NADH dehydrogenase [ubiquinone] flavoprotein 1, mitochondrial (UniProt KB: P49821):

BINDING AFFINITY AND K_D PREDICTION

The binding affinity (ΔG) and dissociation constant (K_d)predicted values are:

Protein-protein complex	ΔG (kcal mol^{-1})	K_D (M) at 25.0 ℃
HsTrx2-P49821	-7.7	2.2E-06

PREDICTION DETAILS

Number of Interfacial Contacts (ICs) per property:

ICs charged-charged:	4
ICs charged-polar:	4
ICs charged-apolar:	18
ICs polar-polar:	1
ICs polar-apolar:	7
ICs apolar-apolar:	40

Non-Interacting Surface (NIS) per property:

NIS charged:	33.51 %
NIS apolar:	38.42 %

Table of the ICs at the interface:

HsTrx2 residue	Number(#1)	Chain(#1)	P49821 residue	Number(#2)	Chain(#2)
CYS	31	A	ARG	152	B

ALA	73	A	VAL	197	B
ALA	73	A	GLN	170	B
LYS	88	A	LYS	219	B
CYS	31	A	PHE	196	B
TRP	30	A	TYR	177	B
GLN	97	A	GLY	237	B
GLY	32	A	ASP	194	B
LYS	35	A	ARG	152	B
CYS	31	A	ASP	194	B
ILE	92	A	PHE	236	B
GLU	70	A	TYR	163	B
PHE	89	A	GLY	218	B
VAL	90	A	GLY	218	B
ILE	92	A	ARG	152	B
ILE	36	A	ARG	152	B
PRO	75	A	TYR	155	B
PHE	89	A	LYS	219	B
PRO	75	A	PHE	196	B
ASP	94	A	CYS	238	B
PHE	89	A	ILE	216	B
VAL	90	A	ILE	216	B
GLY	91	A	PHE	236	B
ASP	94	A	PHE	236	B
LEU	98	A	PHE	236	B
TRP	30	A	PHE	196	B
PHE	89	A	GLU	217	B
LYS	88	A	GLY	218	B
ILE	92	A	TYR	112	B
VAL	90	A	VAL	197	B
SER	34	A	ARG	152	B
TRP	30	A	ASP	194	B
VAL	90	A	SER	215	B
TRP	30	A	VAL	195	B
VAL	71	A	ARG	199	B
LYS	88	A	GLU	217	B
ILE	92	A	TYR	155	B
PRO	33	A	ARG	152	B
SER	72	A	VAL	198	B
GLN	97	A	PHE	236	B
VAL	74	A	PHE	196	B
PHE	101	A	GLY	218	B
LYS	88	A	SER	215	B
ALA	73	A	VAL	198	B
VAL	90	A	TYR	155	B
VAL	90	A	GLU	217	B
PHE	89	A	PHE	236	B

PRO	33	A	TYR	112	B
ALA	73	A	PHE	196	B
GLU	70	A	ARG	199	B
LYS	93	A	PHE	236	B
ILE	92	A	PRO	110	B
ILE	92	A	CYS	238	B
PRO	33	A	TYR	155	B
VAL	74	A	TYR	155	B
ILE	92	A	VAL	235	B
PRO	33	A	PHE	196	B
ALA	73	A	VAL	195	B
GLN	97	A	GLU	217	B
GLY	91	A	ILE	216	B
SER	72	A	ARG	199	B
PRO	33	A	ALA	153	B
VAL	74	A	VAL	197	B
VAL	71	A	VAL	197	B
VAL	90	A	VAL	198	B
GLY	91	A	TYR	155	B
SER	72	A	PHE	196	B
SER	72	A	VAL	197	B
SER	72	A	GLN	170	B
VAL	90	A	PHE	236	B
ALA	73	A	TYR	155	B
PHE	101	A	GLU	217	B
GLY	32	A	ARG	152	B
GLY	32	A	PHE	196	B

27. Isocitrate dehydrogenase [NAD] subunit alpha, mitochondrial (UniProt KB: P50213):

Binding affinity and K_D prediction

The binding affinity (ΔG) and dissociation constant (K_d)predicted values are:

Protein-protein complex	ΔG (kcal mol^{-1})	K_D (M) at 25.0 ℃
HsTrx2-P50213	-5.8	5.8E-05

Prediction details

Number of Interfacial Contacts (ICs) per property:

ICs charged-charged:	2
ICs charged-polar:	5
ICs charged-apolar:	13
ICs polar-polar:	1
ICs polar-apolar:	4
ICs apolar-apolar:	14

Non-Interacting Surface (NIS) per property:

NIS charged:	30.91 %
NIS apolar:	43.33 %

Table of the ICs at the interface:

HsTrx2 residue	Number(#1)	Chain(#1)	P50213 residue	Number(#2)	Chain(#2)
ALA	28	A	ASP	28	B
ASP	60	A	ILE	34	B
LYS	35	A	GLU	38	B
GLN	29	A	MET	24	B
GLY	32	A	ALA	21	B
TRP	30	A	GLN	35	B
TRP	30	A	ASP	28	B
CYS	31	A	ALA	21	B
TRP	30	A	LYS	25	B
SER	72	A	LYS	336	B
ASP	60	A	GLN	35	B
TRP	30	A	ILE	26	B
ASP	60	A	ASP	28	B
CYS	31	A	LYS	25	B
TRP	30	A	VAL	23	B
GLN	29	A	ILE	34	B
TRP	30	A	LYS	31	B
GLY	32	A	MET	24	B
VAL	74	A	ASP	28	B
CYS	31	A	MET	24	B
GLN	29	A	TRP	36	B
TRP	30	A	ALA	21	B
LYS	35	A	MET	24	B
TRP	30	A	ALA	32	B
ASP	60	A	VAL	4	B
ILE	59	A	ASP	28	B
GLN	29	A	GLN	35	B
ASP	60	A	ALA	32	B
TRP	30	A	MET	24	B
TRP	30	A	TRP	36	B
TRP	30	A	ALA	29	B
GLN	29	A	GLU	37	B
TRP	30	A	PRO	33	B
TRP	30	A	PHE	27	B
ASP	61	A	GLN	35	B
ASP	58	A	GLN	35	B
TRP	30	A	ILE	34	B
GLY	32	A	LYS	25	B

CYS	31	A	ASP	28	B

28. Triosephosphate isomerase (UniProt KB: P60174):

Binding affinity and K_D prediction

The binding affinity (ΔG) and dissociation constant (K_d)predicted values are:

Protein-protein complex	ΔG (kcal mol^{-1})	K_D (M) at 25.0 ℃
HsTrx2-P60174	-9.7	7.5E-08

Prediction details

Number of Interfacial Contacts (ICs) per property:

ICs charged-charged:	5
ICs charged-polar:	9
ICs charged-apolar:	20
ICs polar-polar:	0
ICs polar-apolar:	14
ICs apolar-apolar:	21

Non-Interacting Surface (NIS) per property:

NIS charged:	33.33 %
NIS apolar:	38.96 %

Table of the ICs at the interface:

HsTrx2 residue	Number(#1)	Chain(#1)	P60174 residue	Number(#2)	Chain(#2)
ILE	106	A	THR	75	B
VAL	90	A	ASP	49	B
LYS	104	A	GLU	77	B
LEU	105	A	PHE	74	B
PHE	101	A	MET	82	B
ALA	100	A	GLY	72	B
LYS	93	A	ASP	49	B
GLN	97	A	GLY	87	B
ALA	100	A	THR	70	B
LYS	104	A	ALA	73	B
VAL	90	A	TYR	47	B
ASP	96	A	MET	82	B
ILE	92	A	ASP	49	B
LYS	104	A	SER	79	B
GLY	91	A	ALA	46	B
LYS	104	A	GLY	72	B
GLN	97	A	MET	82	B
LYS	93	A	CYS	86	B
LYS	103	A	PHE	74	B

VAL	74	A	PHE	50	B
GLN	97	A	GLY	81	B
LYS	104	A	MET	82	B
ALA	100	A	SER	79	B
LYS	104	A	THR	75	B
ASP	87	A	THR	75	B
GLN	97	A	LYS	84	B
GLY	107	A	GLY	72	B
VAL	86	A	THR	75	B
GLY	91	A	PHE	50	B
LEU	105	A	ALA	73	B
ALA	100	A	ASN	71	B
LYS	93	A	ILE	48	B
LYS	103	A	GLY	72	B
ALA	100	A	MET	82	B
ASP	94	A	ASP	85	B
LEU	105	A	GLY	72	B
LYS	104	A	ILE	78	B
GLN	97	A	ASP	49	B
PHE	89	A	ALA	46	B
VAL	90	A	ALA	46	B
ALA	79	A	THR	75	B
GLY	91	A	ASP	49	B
LYS	93	A	ALA	46	B
PHE	89	A	THR	45	B
GLY	107	A	ALA	73	B
LEU	105	A	THR	75	B
VAL	86	A	GLY	76	B
ASP	96	A	ASP	85	B
LYS	93	A	THR	45	B
PRO	75	A	PHE	50	B
ASP	87	A	GLU	77	B
GLN	97	A	CYS	86	B
LYS	104	A	PHE	74	B
LYS	103	A	ALA	73	B
LYS	88	A	ALA	46	B
ASP	87	A	PHE	74	B
SER	72	A	TYR	47	B
GLN	97	A	ASP	85	B
ILE	106	A	ALA	73	B
ASP	94	A	CYS	86	B
LYS	88	A	THR	45	B
VAL	90	A	PHE	50	B
ALA	73	A	TYR	47	B
LYS	103	A	ASN	71	B
LYS	104	A	GLY	76	B

GLN	97	A	ILE	83	B
ASP	87	A	GLY	76	B
LEU	98	A	MET	82	B
ILE	92	A	PHE	50	B

29. 40S ribosomal protein S18 (UniProt KB: P62269):

BINDING AFFINITY AND K_D PREDICTION

The binding affinity (ΔG) and dissociation constant (K_d)predicted values are:

Protein-protein complex	ΔG (kcal mol^{-1})	K_D (M) at 25.0 ℃
HsTrx2-P62269	-8.0	1.3E-06

PREDICTION DETAILS

Number of Interfacial Contacts (ICs) per property:

ICs charged-charged:	6
ICs charged-polar:	5
ICs charged-apolar:	33
ICs polar-polar:	2
ICs polar-apolar:	4
ICs apolar-apolar:	26

Non-Interacting Surface (NIS) per property:

NIS charged:	30.71 %
NIS apolar:	43.15 %

Table of the ICs at the interface:

HsTrx2 residue	Number(#1)	Chain(#1)	P62269 residue	Number(#2)	Chain(#2)
LYS	88	A	ALA	91	B
ASP	87	A	PRO	98	B
VAL	86	A	PRO	98	B
VAL	85	A	LEU	88	B
LEU	105	A	ALA	114	B
ILE	106	A	ALA	114	B
TYR	69	A	LYS	89	B
SER	72	A	THR	188	B
GLU	70	A	VAL	197	B
SER	72	A	THR	94	B
LEU	78	A	PRO	98	B
TYR	69	A	LEU	201	B
VAL	86	A	ARG	117	B
GLU	70	A	TRP	195	B
GLU	68	A	ALA	92	B
LYS	81	A	ALA	100	B

LYS	104	A	GLU	118	B
VAL	71	A	ALA	93	B
GLU	70	A	THR	94	B
GLY	107	A	ALA	114	B
ASP	84	A	ALA	100	B
LYS	104	A	ALA	115	B
VAL	85	A	PRO	98	B
SER	72	A	ALA	93	B
LYS	103	A	ALA	114	B
ASP	84	A	GLY	101	B
ILE	67	A	LEU	201	B
LYS	88	A	ALA	72	B
ASP	87	A	ALA	96	B
VAL	86	A	ALA	100	B
LYS	88	A	THR	97	B
TYR	69	A	LEU	88	B
GLU	68	A	LEU	201	B
VAL	71	A	ALA	92	B
ASP	84	A	GLN	84	B
GLU	70	A	TYR	202	B
GLU	70	A	LYS	89	B
LYS	88	A	PRO	98	B
GLU	70	A	ALA	93	B
LYS	104	A	ARG	117	B
GLU	68	A	ARG	85	B
LYS	88	A	GLY	95	B
ASP	87	A	ARG	117	B
SER	72	A	ALA	92	B
ILE	67	A	GLU	196	B
VAL	86	A	ILE	99	B
ILE	67	A	VAL	197	B
VAL	85	A	ALA	92	B
ALA	100	A	ALA	114	B
LEU	105	A	ALA	115	B
LYS	104	A	PHE	116	B
GLY	83	A	LEU	88	B
ASP	84	A	LEU	88	B
LYS	88	A	THR	94	B
VAL	85	A	ALA	100	B
GLU	70	A	ALA	92	B
GLU	70	A	PHE	90	B
LYS	104	A	ALA	114	B
GLU	70	A	ALA	179	B
LYS	88	A	ALA	92	B
TYR	69	A	ALA	93	B
VAL	86	A	LEU	88	B

GLY	107	A	ALA	115	B
VAL	85	A	ILE	99	B
GLU	70	A	TRP	176	B
VAL	85	A	ALA	91	B
LYS	88	A	ALA	96	B
LYS	103	A	ALA	115	B
LYS	88	A	ALA	93	B
GLU	70	A	LEU	201	B
GLY	107	A	GLN	113	B
ASP	87	A	ALA	72	B
ASP	87	A	THR	97	B
TYR	69	A	ALA	92	B
VAL	86	A	THR	97	B
GLU	68	A	LYS	89	B

30. O-acetyl-ADP-ribose deacetylase MACROD1 (UniProt KB: Q9BQ69):

BINDING AFFINITY AND K_D PREDICTION

The binding affinity (ΔG) and dissociation constant (K_d)predicted values are:

**Protein-protein complex**	**ΔG (kcal mol^{-1})**	***K_D* (M) at 25.0 ℃**
HsTrx2-Q9BQ69	-11.7	2.4E-09

PREDICTION DETAILS

Number of Interfacial Contacts (ICs) per property:

ICs charged-charged:	16
ICs charged-polar:	14
ICs charged-apolar:	35
ICs polar-polar:	3
ICs polar-apolar:	15
ICs apolar-apolar:	25

Non-Interacting Surface (NIS) per property:

NIS charged:	37.02 %
NIS apolar:	37.02 %

Table of the ICs at the interface:

HsTrx2 residue	Number(#1)	Chain(#1)	Q9BQ69 residue	Number(#2)	Chain(#2)
SER	72	A	ASP	252	B
LYS	104	A	ALA	285	B
ASP	84	A	ALA	240	B
ASP	87	A	ALA	285	B
ALA	73	A	ASP	252	B
ILE	67	A	GLU	242	B

LYS	104	A	ARG	143	B
VAL	90	A	GLU	289	B
VAL	16	A	ALA	237	B
LYS	88	A	LEU	248	B
GLU	68	A	GLU	242	B
VAL	86	A	ALA	240	B
GLU	70	A	GLY	208	B
GLY	83	A	SER	236	B
LEU	78	A	ALA	241	B
GLN	97	A	GLN	293	B
VAL	85	A	ALA	237	B
LYS	88	A	ALA	241	B
LYS	88	A	ALA	285	B
LYS	104	A	HIS	320	B
VAL	86	A	GLU	281	B
TYR	69	A	SER	245	B
ASP	96	A	LYS	145	B
ASP	84	A	PRO	235	B
ASP	84	A	SER	236	B
GLY	91	A	GLN	293	B
GLU	70	A	LYS	206	B
ASP	94	A	GLN	293	B
LYS	93	A	GLU	292	B
ALA	100	A	ARG	143	B
GLU	68	A	ALA	241	B
MET	80	A	ALA	237	B
PHE	89	A	LEU	248	B
VAL	90	A	LEU	248	B
LEU	78	A	ALA	237	B
PHE	89	A	TRP	290	B
LYS	104	A	ARG	317	B
VAL	71	A	SER	245	B
LYS	104	A	TYR	144	B
TYR	69	A	ALA	241	B
ASP	87	A	TYR	144	B
VAL	85	A	ALA	240	B
VAL	85	A	ARG	244	B
SER	72	A	SER	245	B
PHE	89	A	ARG	244	B
GLU	70	A	CYS	246	B
VAL	86	A	ILE	282	B
GLU	99	A	ARG	143	B
TYR	69	A	GLN	239	B
THR	76	A	LEU	248	B
GLY	91	A	HIS	294	B
PHE	101	A	GLU	289	B

ASP	87	A	GLU	281	B
ASP	87	A	THR	286	B
LYS	88	A	TRP	290	B
ALA	100	A	TYR	144	B
ALA	100	A	LYS	145	B
TYR	69	A	ALA	237	B
VAL	85	A	SER	238	B
GLU	70	A	SER	245	B
LEU	78	A	ALA	240	B
LEU	78	A	ARG	244	B
VAL	85	A	SER	236	B
LYS	88	A	ARG	244	B
GLU	70	A	GLU	242	B
PHE	89	A	GLN	293	B
VAL	90	A	GLN	293	B
GLY	83	A	ALA	240	B
VAL	90	A	TRP	290	B
LYS	93	A	GLN	293	B
VAL	85	A	ILE	282	B
SER	72	A	SER	249	B
GLU	68	A	GLN	239	B
LYS	103	A	ARG	143	B
GLU	70	A	LYS	209	B
GLY	83	A	ALA	237	B
VAL	86	A	ALA	285	B
VAL	77	A	GLU	289	B
ASP	87	A	ILE	282	B
PHE	11	A	ALA	237	B
VAL	90	A	ARG	244	B
GLU	70	A	ALA	241	B
GLN	97	A	GLU	292	B
ASP	84	A	ALA	237	B
GLY	83	A	SER	238	B
GLU	70	A	THR	207	B
PHE	89	A	GLU	289	B
ILE	92	A	GLN	293	B
PHE	89	A	THR	286	B
GLY	83	A	PRO	235	B
PHE	101	A	ARG	143	B
ASP	87	A	GLU	289	B
LYS	104	A	TYR	321	B
LYS	93	A	GLU	289	B
GLU	70	A	SER	249	B
GLU	68	A	SER	238	B
VAL	90	A	HIS	294	B
VAL	85	A	TYR	274	B

LYS	88	A	ILE	282	B
VAL	86	A	TYR	321	B
SER	72	A	LEU	248	B
TYR	69	A	GLU	242	B
TYR	69	A	SER	238	B
LYS	88	A	GLU	289	B
LYS	88	A	THR	286	B
VAL	85	A	ALA	241	B
LYS	88	A	SER	245	B
GLN	97	A	ARG	143	B

31. Acetyl-coenzyme A synthetase 2-like, mitochondrial (UniProt KB: Q9NUB1):

BINDING AFFINITY AND K_D PREDICTION

The binding affinity (ΔG) and dissociation constant (K_d)predicted values are:

Protein-protein complex	ΔG (kcal mol^{-1})	K_D (M) at 25.0 ℃
HsTrx2-Q9NUB1	-6.3	2.4E-05

PREDICTION DETAILS

Number of Interfacial Contacts (ICs) per property:

ICs charged-charged:	8
ICs charged-polar:	10
ICs charged-apolar:	12
ICs polar-polar:	2
ICs polar-apolar:	4
ICs apolar-apolar:	1

Non-Interacting Surface (NIS) per property:

NIS charged:	30.51 %
NIS apolar:	42.28 %

Table of the ICs at the interface:

HsTrx2 residue	Number(#1)	Chain(#1)	Q9NUB1 residue	Number(#2)	Chain(#2)
ASP	10	A	VAL	126	B
LYS	56	A	GLU	124	B
ASP	13	A	ILE	129	B
PHE	3	A	GLU	124	B
THR	1	A	ARG	144	B
PHE	3	A	THR	142	B
PRO	9	A	ARG	140	B
ASN	17	A	THR	137	B
ARG	14	A	GLU	145	B
PRO	9	A	LEU	379	B

ASN	17	A	GLU	138	B
GLN	12	A	GLU	138	B
ASP	7	A	ILE	330	B
ASP	7	A	ARG	140	B
GLN	6	A	GLU	124	B
THR	2	A	PRO	123	B
ASN	17	A	GLY	136	B
ARG	14	A	ILE	141	B
ASP	13	A	LEU	379	B
ARG	14	A	ARG	140	B
PRO	9	A	GLU	138	B
PHE	11	A	ARG	140	B
THR	2	A	GLU	124	B
ASP	10	A	ARG	140	B
HIS	49	A	GLU	148	B
ARG	14	A	THR	142	B
GLN	6	A	VAL	126	B
ASP	13	A	VAL	139	B
ASP	13	A	ARG	140	B
ASP	13	A	THR	137	B
ASN	17	A	ARG	132	B
ARG	14	A	VAL	139	B
PRO	9	A	ARG	378	B
ASN	4	A	GLU	124	B
ASP	13	A	GLU	138	B
GLN	6	A	SER	125	B
THR	1	A	GLU	148	B

32. Probable D-lactate dehydrogenase, mitochondrial (UniProt KB: Q86WU2):

BINDING AFFINITY AND K_D PREDICTION

The binding affinity (ΔG) and dissociation constant (K_d)predicted values are:

**Protein-protein complex**	**ΔG (kcal mol^{-1})**	**K_D (M) at 25.0 ℃**
HsTrx2-Q86WU2	-8.4	7.1E-07

PREDICTION DETAILS

Number of Interfacial Contacts (ICs) per property:

ICs charged-charged:	7
ICs charged-polar:	4
ICs charged-apolar:	26
ICs polar-polar:	2
ICs polar-apolar:	7
ICs apolar-apolar:	10

Non-Interacting Surface (NIS) per property:

NIS charged:	26.54 %
NIS apolar:	44.74 %

Table of the ICs at the interface:

HsTrx2 residue	Number(#1)	Chain(#1)	Q86WU2 residue	Number(#2)	Chain(#2)
VAL	90	A	ASN	74	B
ILE	92	A	ARG	198	B
LYS	88	A	LEU	199	B
ILE	92	A	LEU	199	B
LYS	88	A	GLU	76	B
LEU	37	A	ARG	198	B
ILE	92	A	ASP	196	B
PRO	75	A	GLU	76	B
ILE	92	A	ARG	80	B
VAL	90	A	LEU	199	B
VAL	90	A	GLU	76	B
GLY	91	A	VAL	75	B
VAL	90	A	GLY	197	B
VAL	90	A	GLN	77	B
PHE	101	A	ARG	205	B
VAL	86	A	ARG	205	B
ILE	92	A	SER	79	B
LYS	93	A	GLY	197	B
GLN	97	A	ARG	198	B
THR	76	A	GLU	76	B
VAL	71	A	GLU	76	B
GLY	91	A	ARG	198	B
ILE	92	A	GLY	197	B
VAL	90	A	SER	79	B
ASP	94	A	ARG	198	B
LEU	98	A	ARG	198	B
VAL	74	A	ARG	80	B
GLY	91	A	GLU	76	B
ASN	82	A	PRO	226	B
ILE	92	A	ALA	83	B
SER	72	A	GLN	77	B
VAL	85	A	ARG	205	B
ALA	73	A	GLU	76	B
SER	72	A	ASN	74	B
GLY	91	A	ARG	80	B
ASP	87	A	ARG	205	B
VAL	90	A	VAL	75	B
ALA	73	A	GLN	77	B

ALA	73	A	ARG	80	B
LYS	88	A	GLU	191	B
GLY	91	A	SER	79	B
LYS	88	A	ARG	205	B
LYS	88	A	VAL	75	B
ALA	79	A	ARG	205	B
VAL	90	A	VAL	193	B
PHE	89	A	ARG	198	B
VAL	90	A	ARG	198	B
SER	72	A	GLU	76	B
PHE	89	A	LEU	199	B
VAL	90	A	GLU	191	B
LYS	93	A	ARG	198	B
GLY	91	A	GLY	197	B
PHE	89	A	GLU	76	B
SER	72	A	ARG	80	B
LYS	81	A	PRO	226	B
LYS	88	A	HIS	201	B

33. Peroxiredoxin-4 (UniProt KB: Q13162):

BINDING AFFINITY AND K_D PREDICTION

The binding affinity (ΔG) and dissociation constant (K_d)predicted values are:

Protein-protein complex	ΔG (kcal mol^{-1})	K_D (M) at 25.0 ℃
HsTrx2-Q13162	-10.2	3.2E-08

PREDICTION DETAILS

Number of Interfacial Contacts (ICs) per property:

ICs charged-charged:	6
ICs charged-polar:	11
ICs charged-apolar:	28
ICs polar-polar:	3
ICs polar-apolar:	16
ICs apolar-apolar:	21

Non-Interacting Surface (NIS) per property:

NIS charged:	33.51 %
NIS apolar:	40.21 %

Table of the ICs at the interface:

HsTrx2 residue	Number(#1)	Chain(#1)	Q13162 residue	Number(#2)	Chain(#2)
GLU	70	A	VAL	190	B
VAL	16	A	VAL	221	B

LEU	78	A	LEU	216	B
LYS	88	A	LEU	216	B
ASP	87	A	THR	215	B
VAL	86	A	THR	215	B
SER	72	A	TYR	188	B
GLU	70	A	HIS	197	B
LYS	88	A	ARG	212	B
SER	72	A	SER	185	B
ASP	84	A	SER	224	B
PHE	89	A	ILE	214	B
SER	72	A	VAL	190	B
VAL	86	A	GLN	213	B
LEU	78	A	THR	215	B
ILE	92	A	LYS	80	B
ASP	84	A	GLY	222	B
GLN	97	A	SER	82	B
VAL	86	A	GLU	227	B
GLU	70	A	LEU	192	B
VAL	71	A	GLY	189	B
VAL	85	A	ASN	217	B
TYR	69	A	LEU	216	B
TYR	69	A	LEU	199	B
LYS	104	A	PHE	235	B
ILE	67	A	ASP	194	B
LYS	88	A	ILE	214	B
GLY	83	A	VAL	221	B
ASP	87	A	LEU	231	B
ASP	87	A	PHE	235	B
VAL	86	A	LEU	231	B
VAL	90	A	SER	77	B
VAL	85	A	THR	215	B
VAL	90	A	LYS	80	B
THR	76	A	LEU	216	B
GLU	68	A	ASP	218	B
ILE	67	A	LEU	199	B
ASP	87	A	GLN	213	B
ASP	87	A	ILE	81	B
VAL	85	A	GLU	227	B
TYR	69	A	ASP	218	B
GLU	68	A	LEU	216	B
SER	72	A	TYR	191	B
VAL	90	A	ALA	79	B
GLU	68	A	LEU	199	B
LYS	88	A	THR	215	B
GLU	70	A	ARG	200	B
GLU	68	A	ASN	217	B

LYS	104	A	THR	238	B
VAL	85	A	LEU	231	B
SER	72	A	SER	77	B
LYS	88	A	GLN	213	B
GLU	70	A	THR	198	B
ASP	87	A	ARG	212	B
ALA	73	A	LEU	76	B
GLY	91	A	LYS	80	B
TYR	69	A	ASN	217	B
SER	72	A	GLY	189	B
GLU	70	A	LEU	199	B
GLU	70	A	TYR	191	B
ASP	84	A	VAL	221	B
ALA	73	A	SER	77	B
LYS	104	A	ALA	234	B
ILE	67	A	LEU	192	B
ASP	84	A	ARG	223	B
VAL	71	A	LEU	216	B
GLU	68	A	LEU	192	B
VAL	85	A	ARG	200	B
VAL	85	A	ILE	214	B
PHE	89	A	ILE	81	B
VAL	90	A	ILE	81	B
GLU	70	A	GLY	189	B
GLY	83	A	ASN	217	B
ASP	87	A	ILE	214	B
SER	72	A	LYS	186	B
VAL	86	A	ILE	214	B
ASP	84	A	ASN	217	B
VAL	85	A	LEU	216	B
SER	72	A	LEU	76	B
GLN	12	A	VAL	221	B
LEU	78	A	ILE	214	B
ASP	84	A	THR	215	B
LYS	88	A	ILE	81	B
GLU	70	A	LEU	216	B
MET	80	A	LEU	216	B

34. Mitochondrial ribosomal protein L23 (UniProt KB: Q16540):

BINDING AFFINITY AND K_D PREDICTION

The binding affinity (ΔG) and dissociation constant (K_d)predicted values are:

Protein-protein complex	ΔG (kcal mol^{-1})	K_D (M) at 25.0 ℃
HsTrx2-Q16540	-9.9	5.0E-08

PREDICTION DETAILS

Number of Interfacial Contacts (ICs) per property:

ICs charged-charged:	19
ICs charged-polar:	23
ICs charged-apolar:	23
ICs polar-polar:	6
ICs polar-apolar:	12
ICs apolar-apolar:	6

Non-Interacting Surface (NIS) per property:

NIS charged:	28.96 %
NIS apolar:	40.90 %

Table of the ICs at the interface:

HsTrx2 residue	Number(#1)	Chain(#1)	Q16540 residue	Number(#2)	Chain(#2)
ASN	4	A	GLU	330	B
GLN	6	A	HIS	280	B
THR	1	A	ASP	324	B
ASP	13	A	ALA	71	B
THR	2	A	ASP	324	B
THR	20	A	CYS	78	B
ASN	17	A	LYS	70	B
ASP	13	A	SER	75	B
ALA	46	A	GLY	323	B
GLN	12	A	LYS	70	B
ASN	17	A	ASP	69	B
GLN	6	A	ILE	282	B
GLU	19	A	GLN	72	B
LYS	51	A	ASP	322	B
HIS	49	A	ASP	324	B
ASN	17	A	GLU	68	B
THR	1	A	ASP	322	B
THR	2	A	ASP	322	B
THR	2	A	TYR	319	B
LYS	51	A	LYS	81	B
GLU	19	A	ALA	71	B
GLN	48	A	ASP	322	B
ASP	61	A	LYS	279	B
ASN	4	A	LYS	279	B
LYS	51	A	CYS	78	B
VAL	15	A	ALA	74	B
GLU	19	A	SER	75	B
THR	1	A	CYS	78	B
HIS	49	A	ASP	322	B

ASP	58	A	LYS	279	B
GLN	6	A	VAL	124	B
ASN	4	A	THR	278	B
SER	18	A	ALA	74	B
HIS	49	A	LYS	81	B
ILE	5	A	LYS	279	B
ASN	17	A	ALA	71	B
PRO	9	A	LYS	70	B
ASP	13	A	LEU	77	B
HIS	49	A	CYS	78	B
VAL	53	A	CYS	78	B
ALA	46	A	GLU	327	B
ASP	13	A	ALA	74	B
ASN	17	A	SER	75	B
ARG	14	A	LEU	77	B
ARG	14	A	ALA	74	B
ARG	14	A	TYR	194	B
ASP	61	A	HIS	280	B
ASN	4	A	HIS	280	B
VAL	45	A	ASP	324	B
GLY	50	A	ASP	322	B
GLN	6	A	ASN	281	B
VAL	53	A	LEU	77	B
ASN	17	A	ASP	67	B
THR	1	A	PRO	321	B
HIS	49	A	LEU	80	B
THR	2	A	PRO	321	B
ASP	10	A	LYS	279	B
GLY	50	A	LYS	81	B
ALA	46	A	ASP	324	B
ASP	13	A	GLN	72	B
HIS	49	A	GLY	323	B
ASP	10	A	LYS	70	B
ILE	5	A	HIS	280	B
GLY	50	A	CYS	78	B
ASN	17	A	SER	66	B
GLN	6	A	LYS	279	B
ASP	13	A	LYS	70	B
ASP	13	A	ASP	126	B
LYS	47	A	ASP	324	B
THR	1	A	LEU	77	B
ARG	14	A	LYS	70	B
ALA	46	A	ASP	322	B
ASP	13	A	ASP	69	B
ASN	17	A	ALA	74	B
ASP	7	A	GLN	123	B

THR	1	A	GLY	323	B
ASP	7	A	HIS	280	B
PHE	3	A	LEU	77	B
GLU	42	A	GLU	327	B
GLU	19	A	LYS	76	B
ASP	13	A	LEU	73	B
GLU	42	A	LEU	328	B
LYS	43	A	GLU	327	B
ARG	14	A	ASN	281	B
GLN	48	A	LYS	81	B
GLU	19	A	ALA	74	B
GLN	12	A	ASP	67	B
ASN	17	A	GLN	72	B
GLN	6	A	GLN	123	B

35. Protein/nucleic acid deglycase DJ-1 (UniProt KB: Q99497):

Binding affinity and K_D prediction

The binding affinity (ΔG) and dissociation constant (K_d)predicted values are:

Protein-protein complex	ΔG (kcal mol^{-1})	K_D (M) at 25.0 °C
HsTrx2-Q99497	-9.8	6.2E-08

Prediction details

Number of Interfacial Contacts (ICs) per property:

ICs charged-charged:	9
ICs charged-polar:	6
ICs charged-apolar:	17
ICs polar-polar:	0
ICs polar-apolar:	19
ICs apolar-apolar:	8

Non-Interacting Surface (NIS) per property:

NIS charged:	36.27 %
NIS apolar:	42.65 %

Table of the ICs at the interface:

HsTrx2 residue	Number(#1)	Chain(#1)	Q99497 residue	Number(#2)	Chain(#2)
PRO	9	A	ARG	27	B
ASP	13	A	VAL	20	B
ASP	84	A	ASP	55	B
PRO	9	A	ARG	28	B
LYS	81	A	CYS	53	B
ASN	17	A	PRO	54	B

THR	20	A	VAL	51	B
ASN	17	A	VAL	23	B
PRO	9	A	MET	26	B
PRO	9	A	VAL	23	B
GLN	12	A	VAL	23	B
GLU	68	A	ARG	27	B
ASN	17	A	ASP	55	B
SER	18	A	ILE	52	B
GLU	19	A	PRO	43	B
SER	18	A	VAL	51	B
ASP	10	A	ARG	27	B
GLN	12	A	ILE	31	B
ASN	82	A	ASP	55	B
VAL	16	A	PRO	54	B
ASN	17	A	CYS	53	B
ASP	7	A	ASP	24	B
VAL	16	A	ASP	55	B
ASN	82	A	CYS	53	B
PRO	9	A	VAL	25	B
GLN	12	A	LYS	32	B
GLU	19	A	VAL	50	B
ASN	82	A	PRO	43	B
ASP	13	A	VAL	23	B
GLU	19	A	CYS	53	B
VAL	16	A	CYS	53	B
PRO	9	A	ASP	24	B
ASN	82	A	PRO	54	B
ASN	82	A	ALA	56	B
ASN	17	A	LEU	10	B
ASN	82	A	ASP	42	B
GLY	8	A	ARG	27	B
GLY	83	A	ASP	55	B
ASN	17	A	ILE	52	B
ASN	17	A	VAL	35	B
ASN	17	A	VAL	51	B
GLN	12	A	VAL	33	B
GLN	12	A	ARG	27	B
ASP	10	A	ASP	24	B
ASN	82	A	LYS	41	B
THR	20	A	CYS	53	B
GLY	83	A	CYS	53	B
ASP	13	A	ASP	24	B
GLU	19	A	ILE	52	B
GLU	19	A	VAL	51	B
SER	18	A	CYS	53	B
ASP	7	A	ARG	28	B

PRO	9	A	ALA	29	B
GLY	83	A	PRO	54	B
GLU	19	A	ASP	49	B
ASP	64	A	ARG	27	B
ASP	84	A	PRO	54	B
LEU	65	A	ARG	27	B
GLY	8	A	ARG	28	B

36. Aconitate hydratase, mitochondrial (UniProt KB: Q99798):

BINDING AFFINITY AND K_D PREDICTION

The binding affinity (ΔG) and dissociation constant (K_d)predicted values are:

Protein-protein complex	ΔG (kcal mol^{-1})	K_D (M) at 25.0 ℃
HsTrx2-Q99798	-10.0	4.6E-08

PREDICTION DETAILS

Number of Interfacial Contacts (ICs) per property:

ICs charged-charged:	18
ICs charged-polar:	9
ICs charged-apolar:	29
ICs polar-polar:	4
ICs polar-apolar:	9
ICs apolar-apolar:	8

Non-Interacting Surface (NIS) per property:

NIS charged:	33.46 %
NIS apolar:	38.48 %

Table of the ICs at the interface:

HsTrx2 residue	Number(#1)	Chain(#1)	Q99798 residue	Number(#2)	Chain(#2)
HIS	49	A	LYS	258	B
LYS	47	A	THR	256	B
ALA	28	A	GLU	344	B
LYS	56	A	ASN	341	B
ASP	58	A	ASN	341	B
ILE	36	A	GLU	363	B
LYS	35	A	GLU	344	B
ARG	14	A	LEU	337	B
HIS	49	A	THR	256	B
GLU	95	A	ALA	358	B
LYS	43	A	PRO	360	B
GLN	29	A	GLU	344	B
THR	1	A	ILE	254	B

THR	2	A	ILE	254	B
LYS	35	A	ALA	362	B
LYS	47	A	GLY	253	B
LYS	43	A	VAL	367	B
THR	1	A	ILE	338	B
THR	2	A	ILE	338	B
GLU	42	A	LYS	250	B
PRO	39	A	LYS	346	B
ALA	46	A	THR	256	B
LYS	47	A	ALA	358	B
ARG	40	A	GLU	363	B
LYS	43	A	GLU	363	B
LYS	56	A	GLU	344	B
THR	2	A	ILE	340	B
ASN	4	A	THR	235	B
LYS	43	A	HIS	359	B
HIS	49	A	LEU	255	B
ASP	96	A	LYS	370	B
GLU	42	A	GLY	253	B
PRO	39	A	PRO	360	B
LYS	35	A	GLU	510	B
LYS	56	A	LEU	345	B
ALA	46	A	GLY	253	B
ILE	36	A	ALA	362	B
ALA	46	A	LEU	255	B
ASN	4	A	ASN	341	B
PHE	3	A	GLU	339	B
LYS	47	A	ILE	254	B
PRO	39	A	HIS	348	B
ASN	4	A	GLU	339	B
ARG	40	A	PRO	360	B
ASP	61	A	ASN	341	B
LYS	35	A	LYS	346	B
LYS	47	A	LEU	357	B
LYS	43	A	GLY	253	B
HIS	49	A	ILE	254	B
GLU	95	A	GLU	371	B
LYS	43	A	ALA	358	B
GLU	95	A	VAL	367	B
GLU	42	A	ILE	254	B
THR	1	A	LEU	337	B
THR	2	A	LEU	337	B
HIS	27	A	ASN	341	B
LYS	35	A	PRO	360	B
GLU	95	A	GLU	363	B
HIS	49	A	GLY	259	B

ALA	46	A	ILE	254	B
PHE	3	A	ILE	338	B
ASP	58	A	GLU	344	B
PRO	39	A	GLU	344	B
GLU	95	A	HIS	359	B
LYS	47	A	ASP	356	B
PHE	3	A	ILE	340	B
ILE	92	A	GLU	363	B
ASN	4	A	ILE	340	B
LEU	37	A	GLU	363	B
GLU	95	A	LYS	370	B
GLN	29	A	SER	343	B
GLN	6	A	THR	235	B
LYS	43	A	ILE	254	B
HIS	27	A	GLU	344	B
ARG	40	A	LYS	366	B
ILE	36	A	PRO	360	B
THR	2	A	GLU	339	B

39. Crystal structures of the HsTrx2 protein ligands and their resolutions. The numbering (#) of the protein ligands is according to the obtained docking score.

#	Uniprot ID	PDB ID or AF	PRISM	
			Docking score[a] (kcal)	Resolution (Å)
1	P22695	5xte (chain J)	-50.22	3.40
2	P30048	5jcg (chain A)	-49.26	2.80
3	P49821	5xtb (chain A)	-48.13	3.40
4	P07237	6i7s (chain A)	-47.77	2.50
5	P07195	7dbj (chain A)	-37.33	1.55
6	O00483	5z62 (chain N)	-37.25	3.60
7	Q13162	3tjj (chain A)	-34.22	1.91
8	O75489	5xtb (chain O)	-32.7	3.40
9	P00441	2c9v (chain A)	-26.54	1.07
10	P40939	5zqz (chain A)	-26.11	4.20
11	P62269	6zxg (chain U)	-23.24	2.60

12	P32119	7kiz (chain A)	-22.05	1.70
13	Q9NUB1	AF	-21.81	
14	P06576	AF	-17.99	
15	P00395	5z62 (chain A)	-17.92	3.60
16	P50213	7ce3 (chain A)	-17.46	3.47
17	P49247	AF	-15.87	
18	P08559	2ozl (chain A)	-13.94	1.90
19	O75306	5xtd (chain P)	-13.5	3.70
20	P30041	5b6m (chain A)	-12.42	2.50
21	P11177	2ozl (chain B)	-12.3	1.90
22	P30044	3mng (chain A)	-11.72	1.45
23	P38646	AF	-11.13	
24	P25705	AF	-8.98	
25	Q99798	AF	-8.58	
26	Q9BQ69	2x47 (chain A)	-8.33	1.70
27	A8 MXV4	AF	-7.98	
28	P31930	5xte (chain K)	-6.69	3.40
29	O75891	AF	-5.45	
30	O75828	2HRB (chain A)	-5.45	1.90
31	P49411	AF	-3.92	
32	Q99497	1p5f (chain A)	-3.65	1.10
33	P17540	4z9m (chain A)	-2.68	2.10
34	P04406	6ynd (chain A)	-2.39	1.52
35	P48735	5i96 (chain A)	-1.84	1.55
36	Q16540	7of0 (chain EA)	-1.41	2.20
37	P60174	6upf (chain A)	-1.04	1.65
38	Q86 WU2	AF	-2,67	
