# Supplementary material for: Distinct or Overlapping Areas of Mitochondrial Thioredoxin 2 May Be Used for Its Covalent and Strong Non-Covalent Interactions with Protein Ligands
Source: Antioxidants (Basel). 2023 Dec 20;13(1):15. doi: 10.3390/antiox13010015 (PMC10812433; doi:10.3390/antiox13010015)
Supplement: Supplementary file 1 [file antioxidants-13-00015-s001.zip › Supplementary data S2 (intermolecular interactions of HsTrx2).pdf]

Supplementary Data S2: *Mapping of HsTrx2-ligand interactions*

Distinct or shared areas of mitochondrial thioredoxin 2 may be used for its covalent and strong non-covalent interactions with protein ligands

Charalampos Ntallis [1], Haralambos Tzoupis [1], Theodore Tselios [1], Christos T. Chasapis [2] and Alexios Vlamis-Gardikas [1,*]

[1] Department of Chemistry, University of Patras, Rion 26504, Greece; xntallis@gmail.com, c.ntallis@uu.nl (C.N.); haralambostz@gmail.com (H.T.); ttselios@upatras.gr (T.T.)
[2] Institute of Chemical Biology, National Hellenic Research Foundation, Vas. Constantinou 48 av, Athens, 11635, Greece; cchasapis@eie.gr. (C.T.C).

*Correspondence: avlamis@upatras.gr; Tel.: +30-2610-997634

Table 1. Presentation of the contacts of HsTrx2 with its protein ligands by BIOVIA.

1.1. Nucleoside diphosphate-linked moiety X motif 19 (UniProt KB: A8MXV4):

Interacting residues (A8MXV4-HsTrx2)	Category (type)	From (chemistry)	To (chemistry)	Distance (Å)
A8MXV4:LYS252:NZ - HsTrx2:TYR69	Electrostatic (Pi-cation)	A8MXV4:LYS252:NZ (positive)	HsTrx2:TYR69 (Pi-orbitals)	4,81231
A8MXV4:SER251:CB - HsTrx2:GLU70:O	Hydrogen Bond (carbon)	A8MXV4:SER251:CB (H-donor)	HsTrx2:GLU70:O (H-acceptor)	2,93145
HsTrx2:VAL86:CA - A8MXV4:PRO57:O	Hydrogen Bond (carbon)	HsTrx2:VAL86:CA (H-donor)	A8MXV4:PRO57:O (H-acceptor)	2,49931
HsTrx2:LYS88:CE - A8MXV4:LYS252:O	Hydrogen Bond (carbon)	HsTrx2:LYS88:CE (H-donor)	A8MXV4:LYS252:O (H-acceptor)	3,51382
HsTrx2:VAL85:HN - A8MXV4:TRP255	Hydrogen Bond (Pi-donor)	HsTrx2:VAL85:HN (H-donor)	A8MXV4:TRP255 (Pi-orbitals)	3,05148
A8MXV4:PRO57 - HsTrx2:VAL86	Hydrophobic (alkyl)	A8MXV4:PRO57 (alkyl)	HsTrx2:VAL86 (alkyl)	3,86598
A8MXV4:ARG347 - HsTrx2:MET80	Hydrophobic (alkyl)	A8MXV4:ARG347 (alkyl)	HsTrx2:MET80 (alkyl)	4,20477
A8MXV4:ARG347 - HsTrx2:VAL85	Hydrophobic (alkyl)	A8MXV4:ARG347 (alkyl)	HsTrx2:VAL85 (alkyl)	4,7182
A8MXV4:TRP255 - HsTrx2:VAL85	Hydrophobic (Pi-alkyl)	A8MXV4:TRP255 (Pi-orbitals)	HsTrx2:VAL85 (alkyl)	4,76925
A8MXV4:TRP255 - HsTrx2:VAL85	Hydrophobic (Pi-alkyl)	A8MXV4:TRP255 (Pi-orbitals)	HsTrx2:VAL85 (alkyl)	5,16366
HsTrx2:TYR69 - A8MXV4:LYS252	Hydrophobic (Pi-alkyl)	HsTrx2:TYR69 (Pi-orbitals)	A8MXV4:LYS252 (alkyl)	4,93893
HsTrx2:MET80:SD - A8MXV4:ARG347:NH1	Other (sulfur-X, X: O,N,S)	HsTrx2:MET80:SD (sulfur)	A8MXV4:ARG347:NH1 (N)	2,55191

HsTrx2 interacting residues	Electrostatic interaction	Hydrogen bonding	Salt bridge	Hydrophobic interaction	Other
**TYR69**	+	-	-	+	-
**GLU70**	-	+	-	-	-
**MET80**	-	-	-	+	+
**VAL85**	-	+	-	+	-
**VAL86**	-	+	-	+	-
**LYS88**	-	+	-	-	-

1.2. Cytochrome c oxidase subunit NDUFA4 (UniProt KB: O00483):

Interacting residues (O00483-HsTrx2)	Category (type)	From (chemistry)	To (chemistry)	Distance (Å)
HsTrx2:THR1:N - O00483:PHE19	Electrostatic (Pi-cation)	HsTrx2:THR1:N (positive)	O00483:PHE19 (Pi-orbitals)	3,90088
HsTrx2:ASP10:OD1 - O00483:PHE21	Electrostatic (Pi-anion)	HsTrx2:ASP10:OD1 (negative)	O00483:PHE21 (Pi-orbitals)	2,96022

O00483:LEU18:CD2 - HsTrx2:PHE3	Hydrophobic (Pi-sigma)	O00483:LEU18:CD2 (C-H sigma)	HsTrx2:PHE3 (Pi-orbitals)	3,61828
HsTrx2:VAL53 - O00483:LEU18	Hydrophobic (alkyl)	HsTrx2:VAL53 (alkyl)	O00483:LEU18 (alkyl)	4,60741
O00483:PHE21 - HsTrx2:ARG14	Hydrophobic (Pi-alkyl)	O00483:PHE21 (Pi-orbitals)	HsTrx2:ARG14 (alkyl)	4,38368

HsTrx2 interacting residues	Electrostatic interaction	Hydrogen bonding	Salt bridge	Hydrophobic interaction	Other
**THR1**	+	-	-	-	-
**PHE3**	-	-	-	+	-
**ASP10**	+	-	-	-	-
**ARG14**	-	-	-	+	-
**VAL53**	-	-	-	+	-

1.3. NADH dehydrogenase [ubiquinone] iron-sulfur protein 2, mitochondrial (UniProt KB: O75306):

Interacting residues (075306-HsTrx2)	Category (type)	From (chemistry)	To (chemistry)	Distance (Å)
O75306:ARG48:HH22 - HsTrx2:ASP10:OD1	Hydrogen Bond; Electrostatic (salt bridge)	O75306:ARG48:HH22 (H-donor; positive)	HsTrx2:ASP10:OD1 (H-acceptor; negative)	2,05732
O75306:THR43:N - HsTrx2:GLU19:OE1	Electrostatic (attractive charge)	O75306:THR43:N (positive)	HsTrx2:GLU19:OE1 (negative)	5,36588
O75306:ARG48:NH1 - HsTrx2:ASP10:OD2	Electrostatic (attractive charge)	O75306:ARG48:NH1 (positive)	HsTrx2:ASP10:OD2 (negative)	3,4365
O75306:LYS200:NZ - HsTrx2:ASP94:OD2	Electrostatic (attractive charge)	O75306:LYS200:NZ (positive)	HsTrx2:ASP94:OD2 (negative)	5,04301
HsTrx2:THR1:HT1 - O75306:ARG48:O	Hydrogen Bond (conventional)	HsTrx2:THR1:HT1 (H-donor)	O75306:ARG48:O (H-acceptor)	2,3099
HsTrx2:THR1:HT2 - O75306:ARG48:O	Hydrogen Bond (conventional)	HsTrx2:THR1:HT2 (H-donor)	O75306:ARG48:O (H-acceptor)	2,19898
HsTrx2:ARG14:HH12 - O75306:ARG44:O	Hydrogen Bond (conventional)	HsTrx2:ARG14:HH12 (H-donor)	O75306:ARG44:O (H-acceptor)	2,09477
HsTrx2:ARG14:HH12 - O75306:VAL47:O	Hydrogen Bond (conventional)	HsTrx2:ARG14:HH12 (H-donor)	O75306:VAL47:O (H-acceptor)	2,29505
HsTrx2:GLU19:HN - O75306:THR46:OG1	Hydrogen Bond (conventional)	HsTrx2:GLU19:HN (H-donor)	O75306:THR46:OG1 (H-acceptor)	1,79135
O75306:THR46:HG1 - HsTrx2:THR20:O	Hydrogen Bond (conventional)	O75306:THR46:HG1 (H-donor)	HsTrx2:THR20:O (H-acceptor)	2,3355
HsTrx2:ARG14:CA - O75306:PRO45:O	Hydrogen Bond (carbon)	HsTrx2:ARG14:CA (H-donor)	O75306:PRO45:O (H-acceptor)	3,752
HsTrx2:ARG14:CD - O75306:PRO45:O	Hydrogen Bond (carbon)	HsTrx2:ARG14:CD (H-donor)	O75306:PRO45:O (H-acceptor)	3,26591
HsTrx2:LYS103:CE - O75306:TRP239:O	Hydrogen Bond (carbon)	HsTrx2:LYS103:CE (H-donor)	O75306:TRP239:O (H-acceptor)	2,5079
HsTrx2:LYS104:CE - O75306:LEU235:O	Hydrogen Bond (carbon)	HsTrx2:LYS104:CE (H-donor)	O75306:LEU235:O (H-acceptor)	3,61004
O75306:VAL117 - HsTrx2:ILE36	Hydrophobic (alkyl)	O75306:VAL117 (alkyl)	HsTrx2:ILE36 (alkyl)	5,1491

O75306:ALA241 - HsTrx2:LYS103	Hydrophobic (alkyl)	O75306:ALA241 (alkyl)	HsTrx2:LYS103 (alkyl)	3,18304
HsTrx2:PHE3 - O75306:ARG48	Hydrophobic (Pi-alkyl)	HsTrx2:PHE3 (Pi-orbitals)	O75306:ARG48 (alkyl)	5,35775
O75306:PHE81 - HsTrx2:ALA46	Hydrophobic (Pi-alkyl)	O75306:PHE81 (Pi-orbitals)	HsTrx2:ALA46 (alkyl)	3,92115
O75306:PHE242 - HsTrx2:LEU102	Hydrophobic (Pi-alkyl)	O75306:PHE242 (Pi-orbitals)	HsTrx2:LEU102 (alkyl)	4,80094

HsTrx2 interacting residues	Electrostatic interaction	Hydrogen bonding	Salt bridge	Hydrophobic interaction	Other
**THR1**	-	+	-	-	-
**PHE3**	-	-	-	+	-
**ASP10**	+	+	+	-	-
**ARG14**	-	+	-	-	-
**GLU19**	+	+	-	-	-
**THR20**	-	+	-	-	-
**ILE36**	-	-	-	+	-
**ALA46**	-	-	-	+	-
**ASP94**	+	-	-	-	-
**LEU102**	-	-	-	+	-
**LYS103**	-	+	-	+	-
**LYS104**	-	+	-	-	-

1.4. NADH dehydrogenase [ubiquinone] iron-sulfur protein 3, mitochondrial (UniProt KB: O75489):

Interacting residues (075489-HsTrx2)	Category (type)	From (chemistry)	To (chemistry)	Distance (Å)
O75489:TYR119:HH - HsTrx2:GLN6:OE1	Hydrogen Bond (conventional)	O75489:TYR119:HH (H-donor)	HsTrx2:GLN6:OE1 (H-acceptor)	3,04936
O75489:THR120:HN - HsTrx2:ASP7:OD2	Hydrogen Bond (conventional)	O75489:THR120:HN (H-donor)	HsTrx2:ASP7:OD2 (H-acceptor)	2,73109
HsTrx2:THR1:HT1 - O75489:HIS74:O	Hydrogen Bond (conventional)	HsTrx2:THR1:HT1 (H-donor)	O75489:HIS74:O (H-acceptor)	2,68133
O75489:TYR119:CA - HsTrx2:ASP7:OD2	Hydrogen Bond (carbon)	O75489:TYR119:CA (H-donor)	HsTrx2:ASP7:OD2 (H-acceptor)	3,76026
HsTrx2:ALA46 - O75489:PRO71	Hydrophobic (alkyl)	HsTrx2:ALA46 (alkyl)	O75489:PRO71 (alkyl)	3,31571
O75489:PHE118 - HsTrx2:PRO9	Hydrophobic (Pi-alkyl)	O75489:PHE118 (Pi-orbitals)	HsTrx2:PRO9 (alkyl)	4,63458
HsTrx2:THR2:O - O75489:TYR70	Other (Pi-lone pair)	HsTrx2:THR2:O (lone pair)	O75489:TYR70 (Pi-orbitals)	2,91441
HsTrx2:GLN6:O - O75489:TYR119	Other (Pi-lone pair)	HsTrx2:GLN6:O (lone pair)	O75489:TYR119 (Pi-orbitals)	2,17213

HsTrx2 interacting residues	Electrostatic interaction	Hydrogen bonding	Salt bridge	Hydrophobic interaction	Other

**THR1**	-	+	-	-	-
**THR2**	-	-	-	-	+
**GLN6**	-	+	-	-	+
**ASP7**	-	+	-	-	-
**PRO9**	-	-	-	+	-
**ALA46**	-	-	-	+	-

1.5. Carbonyl reductase [NADPH] 3 (UniProt KB: O75828):

Interacting residues (O75828-HsTrx2)	Category (type)	From (chemistry)	To (chemistry)	Distance (Å)
O75828:ARG71:NH1 - HsTrx2:ASP84:OD2	Electrostatic (attractive charge)	O75828:ARG71:NH1 (positive)	HsTrx2:ASP84:OD2 (negative)	5,56527
O75828:LYS173:NZ - HsTrx2:GLU70:OE2	Electrostatic (attractive charge)	O75828:LYS173:NZ (positive)	HsTrx2:GLU70:OE2 (negative)	3,90191
HsTrx2:GLU70:OE2 - O75828:PHE116	Electrostatic (Pi-anion)	HsTrx2:GLU70:OE2 (negative)	O75828:PHE116 (Pi-orbitals)	4,57189
HsTrx2:SER72:HG - O75828:GLU108:O	Hydrogen Bond (conventional)	HsTrx2:SER72:HG (H-donor)	O75828:GLU108:O (H-acceptor)	3,03268
O75828:LEU67:HN - HsTrx2:VAL86:O	Hydrogen Bond (conventional)	O75828:LEU67:HN (H-donor)	HsTrx2:VAL86:O (H-acceptor)	2,49247
O75828:LYS180:HZ1 - HsTrx2:VAL71:O	Hydrogen Bond (conventional)	O75828:LYS180:HZ1 (H-donor)	HsTrx2:VAL71:O (H-acceptor)	2,59458
O75828:LYS173:CE - HsTrx2:GLU70:OE2	Hydrogen Bond (carbon)	O75828:LYS173:CE (H-donor)	HsTrx2:GLU70:OE2 (H-acceptor)	2,75169
HsTrx2:ALA73 - O75828:MET109	Hydrophobic (alkyl)	HsTrx2:ALA73 (alkyl)	O75828:MET109 (alkyl)	3,18204
HsTrx2:VAL86 - O75828:LEU67	Hydrophobic (alkyl)	HsTrx2:VAL86 (alkyl)	O75828:LEU67 (alkyl)	4,99733
HsTrx2:VAL90 - O75828:MET109	Hydrophobic (alkyl)	HsTrx2:VAL90 (alkyl)	O75828:MET109 (alkyl)	5,34511
O75828:LYS180 - HsTrx2:ILE67	Hydrophobic (alkyl)	O75828:LYS180 (alkyl)	HsTrx2:ILE67 (alkyl)	4,48474

HsTrx2 interacting residues	Electrostatic interaction	Hydrogen bonding	Salt bridges	Hydrophobic interaction	Other
**ILE67**	-	-	-	+	-
**GLU70**	+	+	-	-	-
**VAL71**	-	+	-	-	-
**SER72**	-	+	-	-	-
**ALA73**	-	-	-	+	-
**ASP84**	+	-	-	-	-
**VAL86**	-	+	-	+	-
**VAL90**	-	-	-	+	-

1.6. Cytosolic 10-formyltetrahydrofolate dehydrogenase (UniProt KB: O75891):

Interacting residues (O75891-HsTrx2)	Category (type)	From (chemistry)	To (chemistry)	Distance (Å)
O75891:ARG483:NH1 - HsTrx2:ASP96:OD2	Electrostatic (attractive charge)	O75891:ARG483:NH1 (positive)	HsTrx2:ASP96:OD 2 (negative)	5,57103
O75891:ARG487:NH1 - HsTrx2:ASP96:OD2	Electrostatic (attractive charge)	O75891:ARG487:NH1 (positive)	HsTrx2:ASP96:OD 2 (negative)	5,47565
O75891:ARG487:NH1 - HsTrx2:GLU99:OE1	Electrostatic (attractive charge)	O75891:ARG487:NH1 (positive)	HsTrx2:GLU99:OE 1 (negative)	3,43867
O75891:ARG487:NH2 - HsTrx2:ASP96:OD1	Electrostatic (attractive charge)	O75891:ARG487:NH2 (positive)	HsTrx2:ASP96:OD 1 (negative)	2,51338
O75891:ARG487:NH2 - HsTrx2:GLU99:OE2	Electrostatic (attractive charge)	O75891:ARG487:NH2 (positive)	HsTrx2:GLU99:OE 2 (negative)	4,90214
O75891:ARG491:NH1 - HsTrx2:GLY107:O	Electrostatic (attractive charge)	O75891:ARG491:NH1 (positive)	HsTrx2:GLY107:O (negative)	5,46212
O75891:ARG491:NH2 - HsTrx2:GLY107:OXT	Electrostatic (attractive charge)	O75891:ARG491:NH2 (positive)	HsTrx2:GLY107:O XT (negative)	3,78225
HsTrx2:LYS47:NZ - O75891:GLU366:OE2	Electrostatic (attractive charge)	HsTrx2:LYS47:NZ (positive)	O75891:GLU366: OE2 (negative)	4,34313
HsTrx2:LYS104:NZ - O75891:ASP494:OD1	Electrostatic (attractive charge)	HsTrx2:LYS104:NZ (positive)	O75891:ASP494:O D1 (negative)	5,11744
HsTrx2:LYS103:HZ1 - O75891:ARG487:O	Hydrogen Bond (conventional)	HsTrx2:LYS103:HZ1 (H-donor)	O75891:ARG487:O (H-acceptor)	1,95865
O75891:ARG483:CD - HsTrx2:ASP96:OD2	Hydrogen Bond (carbon)	O75891:ARG483:CD (H-donor)	HsTrx2:ASP96:OD 2 (H-acceptor)	3,40204
HsTrx2:HIS49 - O75891:LEU367	Hydrophobic (Pi-alkyl)	HsTrx2:HIS49 (Pi-orbitals)	O75891:LEU367 (alkyl)	4,93497

HsTrx2 interacting residues	Electrostatic interaction	Hydrogen bonding	Salt bridge	Hydrophobic interaction	Other
**LYS47**	+	-	-	-	-
**HIS49**	-	-	-	+	-
**ASP96**	+	+	-	-	-
**GLU99**	+	-	-	-	-
**LYS103**	-	+	-	-	-
**LYS104**	+	-	-	-	-
**GLY107**	+	-	-	-	-

1.7. Cytochrome c oxidase subunit 1 (UniProt KB: P00395):

Interacting residues (P00395-HsTrx2)	Category (type)	From (chemistry)	To (chemistry)	Distance (Å)
HsTrx2:SER72:HG - P00395:ASN46:O	Hydrogen Bond (conventional)	HsTrx2:SER72:HG (H-donor)	P00395:ASN46:O (H-acceptor)	2,02579
HsTrx2:LYS81 - P00395:MET117	Hydrophobic (alkyl)	HsTrx2:LYS81 (alkyl)	P00395:MET117 (alkyl)	3,69876
HsTrx2:VAL85 - P00395:ILE53	Hydrophobic (alkyl)	HsTrx2:VAL85 (alkyl)	P00395:ILE53 (alkyl)	5,37526
HsTrx2:LYS88 - P00395:LEU48	Hydrophobic (alkyl)	HsTrx2:LYS88 (alkyl)	P00395:LEU48 (alkyl)	4,64438
HsTrx2:ALA100 -	Hydrophobic	HsTrx2:ALA100	P00395:ALA32	3,68605

P00395:ALA32	(alkyl)	(alkyl)	(alkyl)	
HsTrx2:LYS104 - P00395:LEU29	Hydrophobic (alkyl)	HsTrx2:LYS104 (alkyl)	P00395:LEU29 (alkyl)	4,53359
P00395:ALA32 - HsTrx2:LYS104	Hydrophobic (alkyl)	P00395:ALA32 (alkyl)	HsTrx2:LYS104 (alkyl)	3,6587
P00395:ALA116 - HsTrx2:VAL86	Hydrophobic (alkyl)	P00395:ALA116 (alkyl)	HsTrx2:VAL86 (alkyl)	4,48017

HsTrx2 interacting residues	Electrostatic interaction	Hydrogen bonding	Salt bridge	Hydrophobic interaction	Other
**SER72**	-	+	-	-	-
**LYS81**	-	-	-	+	-
**VAL85**	-	-	-	+	-
**VAL86**	-	-	-	+	-
**LYS88**	-	-	-	+	-
**ALA100**	-	-	-	+	-
**LYS104**	-	-	-	+	-

1.8. Superoxide dismutase 1, soluble (UniProt KB: P00441):

Interacting residues (P00441-HsTrx2)	Category (type)	From (chemistry)	To (chemistry)	Distance (Å)
P00441:CYS111:SG - HsTrx2:ASP60:OD1	Hydrogen Bond (conventional)	P00441:CYS111:SG (H-donor)	HsTrx2:ASP60:OD1 (H-acceptor)	2,69937
P00441:ILE113:HN - HsTrx2:GLN29:OE1	Hydrogen Bond (conventional)	P00441:ILE113:HN (H-donor)	HsTrx2:GLN29:OE1 (H-acceptor)	2,63344
P00441:ARG115:HE - HsTrx2:GLN29:OE1	Hydrogen Bond (conventional)	P00441:ARG115:HE (H-donor)	HsTrx2:GLN29:OE1 (H-acceptor)	3,00465
HsTrx2:GLN29:HE21 - P00441:CYS111:O	Hydrogen Bond (conventional)	HsTrx2:GLN29:HE21 (H-donor)	P00441:CYS111:O (H-acceptor)	2,95252
HsTrx2:TRP30:HN - P00441:CYS111:SG	Hydrogen Bond (conventional)	HsTrx2:TRP30:HN (H-donor)	P00441:CYS111:SG (H-acceptor)	2,95435
HsTrx2:TRP30:HE1 - P00441:SER105:O	Hydrogen Bond (conventional)	HsTrx2:TRP30:HE1 (H-donor)	P00441:SER105:O (H-acceptor)	2,2168
P00441:LEU106:CD2 - HsTrx2:TRP30	Hydrophobic (Pi-sigma)	P00441:LEU106:CD2 (C-H sigma)	HsTrx2:TRP30 (Pi-orbitals)	2,57113
P00441:ILE113:CD1 - HsTrx2:TRP30	Hydrophobic (Pi-sigma)	P00441:ILE113:CD1 (C-H sigma)	HsTrx2:TRP30 (Pi-orbitals)	3,21708
P00441:ILE113:CD1 - HsTrx2:TRP30	Hydrophobic (Pi-sigma)	P00441:ILE113:CD1 (C-H sigma)	HsTrx2:TRP30 (Pi-orbitals)	3,09646
HsTrx2:TRP30 - P00441:LEU106	Hydrophobic (Pi-alkyl)	HsTrx2:TRP30 (Pi-orbital)	P00441:LEU106 (alkyl)	4,17291
HsTrx2:TRP30 - P00441:CYS111	Hydrophobic (Pi-alkyl)	HsTrx2:TRP30 (Pi-orbital)	P00441:CYS111 (alkyl)	3,87441
HsTrx2:TRP30 - P00441:ILE112	Hydrophobic (Pi-alkyl)	HsTrx2:TRP30 (Pi-orbital)	P00441:ILE112 (alkyl)	5,45451
HsTrx2:TRP30 - P00441:ALA4	Hydrophobic (Pi-alkyl)	HsTrx2:TRP30 (Pi-orbital)	P00441:ALA4 (alkyl)	3,65657

HsTrx2:TRP30 - P00441:ILE112	Hydrophobic (Pi-alkyl)	HsTrx2:TRP30 (Pi-orbital)	P00441:ILE112 (alkyl)	5,06277

HsTrx2 interacting residues	Electrostatic interaction	Hydrogen bonding	Salt bridge	Hydrophobic interaction	Other
**GLN29**	-	+	-	-	-
**TRP30**	-	+	-	+	-
**ASP60**	-	+	-	-	-

1.9. Glyceraldehyde-3-phosphate dehydrogenase (UniProt KB: P04406):

Interacting residues (P04406-HsTrx2)	Category (type)	From (chemistry)	To (chemistry)	Distance (Å)
P04406:ARG80:NH1 - HsTrx2:ASP84:OD2	Electrostatic (attractive charge)	P04406:ARG80:NH1 (positive)	HsTrx2:ASP84:OD2 (negative)	4,7563
P04406:ARG80:NH2 - HsTrx2:ASP84:OD1	Electrostatic (attractive charge)	P04406:ARG80:NH2 (positive)	HsTrx2:ASP84:OD1 (negative)	3,05355
P04406:ASN41:HN - HsTrx2:GLU70:O	Hydrogen Bond (conventional)	P04406:ASN41:HN (H-donor)	HsTrx2:GLU70:O (H-acceptor)	2,04316
P04406:ASN41:HD22 - HsTrx2:GLU70:OE1	Hydrogen Bond (conventional)	P04406:ASN41:HD22 (H-donor)	HsTrx2:GLU70:OE1 (H-acceptor)	2,15234
P04406:ARG80:HH11 - HsTrx2:VAL85:O	Hydrogen Bond (conventional)	P04406:ARG80:HH11 (H-donor)	HsTrx2:VAL85:O (H-acceptor)	2,09734
HsTrx2:SER72:HG - P04406:ASN41:OD1	Hydrogen Bond (conventional)	HsTrx2:SER72:HG (H-donor)	P04406:ASN41:OD1 (H-acceptor)	2,57994

HsTrx2 interacting residues	Electrostatic interaction	Hydrogen bonding	Salt bridge	Hydrophobic interaction	Other
**GLU70**	-	+	-	-	-
**SER72**	-	+	-	-	-
**ASP84**	+	-	-	-	-
**VAL85**	-	+	-	-	-

1.10. ATP synthase subunit beta, mitochondrial (UniProt KB: P06576):

Interacting residues (P06576-HsTrx2)	Category (type)	From (chemistry)	To (chemistry)	Distance (Å)
P06576:LEU79:HN – HsTrx2:VAL74:O	Hydrogen Bond (conventional)	P06576:LEU79:HN (H-donor)	HsTrx2:VAL74:O (H-acceptor)	2,03783
P06576:GLU105:HN – HsTrx2:TRP30:O	Hydrogen Bond (conventional)	P06576:GLU105:HN (H-donor)	HsTrx2:TRP30:O (H-acceptor)	2,39798
P06576:SER106:HN – HsTrx2:TRP30:O	Hydrogen Bond (conventional)	P06576:SER106:HN (H-donor)	HsTrx2:TRP30:O (H-acceptor)	2,33073
HsTrx2:PRO33:CD – P06576:ASP76:O	Hydrogen Bond (carbon)	HsTrx2:PRO33:CD (H-donor)	P06576:ASP76:O (alkyl)	3,18284
HsTrx2:ALA73 – P06576:LEU79	Hydrophobic (alkyl)	HsTrx2:ALA73 (alkyl)	P06576:LEU79 (alkyl)	4,45742
HsTrx2:ALA73 – P06576:PRO81	Hydrophobic (alkyl)	HsTrx2:ALA73 (alkyl)	P06576:PRO81 (alkyl)	3,93359

HsTrx2:VAL90 – P06576:PRO81	Hydrophobic (alkyl)	HsTrx2:VAL90 (alkyl)	P06576:PRO81 (alkyl)	3,56294
P06576:HIS102 – HsTrx2:ALA73	Hydrophobic (Pi-alkyl)	P06576:HIS102 (Pi-orbitals)	HsTrx2:ALA73 (alkyl)	3,94008

HsTrx2 interacting residues	Electrostatic interaction	Hydrogen bonding	Salt bridge	Hydrophobic interaction	Other
**TRP30**	-	+	-	-	-
**PRO33**	-	+	-	-	-
**ALA73**	-	-	-	+	-
**VAL74**	-	+	-	-	-
**VAL90**	-	-	-	+	-

1.11. L-lactate dehydrogenase B chain, LDH-B (UniProt KB: P07195):

Interacting residues (P07195-HsTrx2)	Category (type)	From (chemistry)	To (chemistry)	Distance (Å)
P07195:ARG172:HH22 - HsTrx2:ASP13:OD1	Electrostatic (attractive charge)	P07195:ARG172:HH22 (positive)	HsTrx2:ASP13:OD1 (negative)	2,52807
P07195:ARG172:NH1 - HsTrx2:ASP13:OD2	Electrostatic (attractive charge)	P07195:ARG172:NH1 (positive)	HsTrx2:ASP13:OD2 (negative)	3,38987
P07195:ARG270:NH2 - HsTrx2:ASP7:OD2	Electrostatic (attractive charge)	P07195:ARG270:NH2 (positive)	HsTrx2:ASP7:OD2 (negative)	4,24882
HsTrx2:ARG14:NH1 - P07195:TRP189	Electrostatic (Pi-cation)	HsTrx2:ARG14:NH1 (positive)	P07195:TRP189 (Pi-orbitals)	4,60065
HsTrx2:ARG14:NH2 - P07195:TRP189	Electrostatic (Pi-cation)	HsTrx2:ARG14:NH2 (positive)	P07195:TRP189 (Pi-orbitals)	3,17384
HsTrx2:ASP13:OD1 - P07195:HIS187	Electrostatic (Pi-anion)	HsTrx2:ASP13:OD1 (negative)	P07195:HIS187 (Pi-orbitals)	2,57417
P07195:ASN206:HD22 - HsTrx2:ASN17:O	Hydrogen Bond (conventional)	P07195:ASN206:HD22 (H-donor)	HsTrx2:ASN17:O (H-acceptor)	2,742
P07195:GLY209:HN - HsTrx2:ASN17:OD1	Hydrogen Bond (conventional)	P07195:GLY209:HN (H-donor)	HsTrx2:ASN17:OD1 (H-acceptor)	2,16377
P07195:ILE271:HN - HsTrx2:ASP10:OD1	Hydrogen Bond (conventional)	P07195:ILE271:HN (H-donor)	HsTrx2:ASP10:OD1 (H-acceptor)	2,67913
P07195:GLN307:HE22 - HsTrx2:GLU19:OE2	Hydrogen Bond (conventional)	P07195:GLN307:HE22 (H-donor)	HsTrx2:GLU19:OE2 (H-acceptor)	1,8805
HsTrx2:THR1:HT1 - P07195:VAL304:O	Hydrogen Bond (conventional)	HsTrx2:THR1:HT1 (H-donor)	P07195:VAL304:O (H-acceptor)	2,42872
HsTrx2:ASN4:HN - P07195:SER269:O	Hydrogen Bond (conventional)	HsTrx2:ASN4:HN (H-donor)	P07195:SER269:O (H-acceptor)	2,40012
HsTrx2:ASN4:HD22 - P07195:LEU268:O	Hydrogen Bond (conventional)	HsTrx2:ASN4:HD22 (H-donor)	P07195:LEU268:O (H-acceptor)	2,71133
P07195:GLY209:CA - HsTrx2:VAL16:O	Hydrogen Bond (carbon)	P07195:GLY209:CA (H-donor)	HsTrx2:VAL16:O (H-acceptor)	3,51736
HsTrx2:GLY50:CA - P07195:ASN306:O	Hydrogen Bond (carbon)	HsTrx2:GLY50:CA (H-donor)	P07195:ASN306:O (H-acceptor)	2,89928

HsTrx2:GLY50:CA - P07195:ASN306:OD1	Hydrogen Bond (carbon)	HsTrx2:GLY50:CA (H-donor)	P07195:ASN306:OD1 (H-acceptor)	3,34119
P07195:ARG172:HH22 - HsTrx2:ASP13:OD1	Hydrogen Bond (conventional)	P07195:ARG172:HH22 (H-donor)	HsTrx2:ASP13:OD1 (H-acceptor)	2,52807
P07195:ILE271:CD1 - HsTrx2:PHE3	Hydrophobic (Pi-sigma)	P07195:ILE271:CD1 (C-H sigma)	HsTrx2:PHE3 (Pi-orbitals)	2,76183
HsTrx2:ARG14 - P07195:ILE271	Hydrophobic (alkyl)	HsTrx2:ARG14 (alkyl)	P07195:ILE271 (alkyl)	4,89557
HsTrx2:VAL53 - P07195:ILE305	Hydrophobic (alkyl)	HsTrx2:VAL53 (alkyl)	P07195:ILE305 (alkyl)	5,46662

HsTrx2 interacting residues	Electrostatic interaction	Hydrogen bonding	Salt bridge	Hydrophobic interaction	Other
**THR1**	-	+	-	-	-
**PHE3**	-	-	-	+	-
**ASN4**	-	+	-	-	-
**ASP7**	+	-	-	-	-
**ASP10**	-	+	-	-	-
**ASP13**	+	+	+	-	-
**ARG14**	+	-	-	+	-
**VAL16**	-	+	-	-	-
**ASN17**	+	+	-	-	-
**GLU19**	-	+	-	-	-
**GLY50**	-	+	-	-	-
**VAL53**	-	-	-	+	-

1.12. Protein disulfide-isomerase (UniProt KB: P07237):

Interacting residues (P07237-HsTrx2)	Category (type)	From (chemistry)	To (chemistry)	Distance (Å)
P07237:TRP396:HE1 - HsTrx2:VAL71:O	Hydrogen Bond (conventional)	P07237:TRP396:HE1 (H-donor)	HsTrx2:VAL71:O (H-acceptor)	1,65644
P07237:CYS397:SG - HsTrx2:SER72:O	Hydrogen Bond (conventional)	P07237:CYS397:SG (H-donor)	HsTrx2:SER72:O (H-acceptor)	3,08697
P07237:VAL437:HN - HsTrx2:TRP30:O	Hydrogen Bond (conventional)	P07237:VAL437:HN (H-donor)	HsTrx2:TRP30:O (H-acceptor)	2,40005
P07237:HIS438:ND1 - HsTrx2:CYS31:SG	Hydrogen Bond (conventional)	P07237:HIS438:ND1 (H-donor)	HsTrx2:CYS31:SG (H-acceptor)	3,73837
HsTrx2:TRP30:CD1 - P07237:VAL437:O	Hydrogen Bond (carbon)	HsTrx2:TRP30:CD1 (H-donor)	P07237:VAL437:O (H-acceptor)	3,03326
P07237:THR428:CA - HsTrx2:ASP60:OD1	Hydrogen Bond (carbon)	P07237:THR428:CA (H-donor)	HsTrx2:ASP60:OD1 (H-acceptor)	2,86913
HsTrx2:ALA66:CB - P07237:TRP396	Hydrophobic (Pi-sigma)	HsTrx2:ALA66:CB (C-H sigma)	P07237:TRP396 (Pi-orbitals)	3,19519
HsTrx2:ALA66:CB - P07237:TRP396	Hydrophobic (Pi-sigma)	HsTrx2:ALA66:CB (C-H sigma)	P07237:TRP396 (Pi-orbitals)	2,76581
HsTrx2:TRP30 - P07237:VAL437	Hydrophobic (Pi-alkyl)	HsTrx2:TRP30 (Pi-orbitals)	P07237:VAL437 (alkyl)	4,60809

P07237:TRP396 - HsTrx2:ILE67	Hydrophobic (Pi-alkyl)	P07237:TRP396 (Pi-orbitals)	HsTrx2:ILE67 (alkyl)	4,89661
P07237:TRP396 - HsTrx2:VAL71	Hydrophobic (Pi-alkyl)	P07237:TRP396 (Pi-orbitals)	HsTrx2:VAL71 (alkyl)	5,25721
P07237:TRP396 - HsTrx2:ILE59	Hydrophobic (Pi-alkyl)	P07237:TRP396 (Pi-orbitals)	HsTrx2:ILE59 (alkyl)	4,70762
P07237:TRP396 - HsTrx2:ILE67	Hydrophobic (Pi-alkyl)	P07237:TRP396 (Pi-orbitals)	HsTrx2:ILE67 (alkyl)	5,23623
P07237:HIS438 - HsTrx2:CYS31	Hydrophobic (Pi-alkyl)	P07237:HIS438 (Pi-orbitals)	HsTrx2:CYS31 (alkyl)	4,83362
P07237:HIS438 - HsTrx2:PRO33	Hydrophobic (Pi-alkyl)	P07237:HIS438 (Pi-orbitals)	HsTrx2:PRO33 (alkyl)	4,51481
P07237:PHE440 - HsTrx2:VAL74	Hydrophobic (Pi-alkyl)	P07237:PHE440 (Pi-orbitals)	HsTrx2:VAL74 (alkyl)	5,42583

HsTrx2 interacting residues	Electrostatic interaction	Hydrogen bonding	Salt bridge	Hydrophobic interaction	Other
**TRP30**	-	+	-	+	-
**CYS31**	-	+	-	+	-
**PRO33**	-	-	-	+	-
**ILE59**	-	-	-	+	-
**ASP60**	-	+	-	-	-
**ALA66**	-	-	-	+	-
**ILE67**	-	-	-	+	-
**VAL71**	-	+	-	+	-
**SER72**	-	+	-	-	-
**VAL74**	-	-	-	+	-

1.13. Pyruvate dehydrogenase E1 component subunit alpha, somatic form, mitochondrial (UniProt KB: P08559):

Interacting residues (P08559-HsTrx2)	Category (type)	From (chemistry)	To (chemistry)	Distance (Å)
P08559:ARG44:HH12 - HsTrx2:ASP61:OD1	Hydrogen Bond; Electrostatic (salt bridge)	P08559:ARG44:HH12 (H-donor;positive)	HsTrx2:ASP61:OD1 (H-acceptor; negative)	2,37628
P08559:ARG44:NH2 - HsTrx2:ASP61:OD2	Electrostatic (attractive charge)	P08559:ARG44:NH2 (positive)	HsTrx2:ASP61:OD2 (negative)	3,98789
P08559:ARG314:NH1 - HsTrx2:ASP61:OD1	Electrostatic (attractive charge)	P08559:ARG314:NH1 (positive)	HsTrx2:ASP61:OD1 (negative)	2,44866
P08559:ARG314:NH2 - HsTrx2:ASP58:OD1	Electrostatic (attractive charge)	P08559:ARG314:NH2 (positive)	HsTrx2:ASP58:OD1 (negative)	4,33613
P08559:ARG314:NH2 - HsTrx2:ASP60:OD2	Electrostatic (attractive charge)	P08559:ARG314:NH2 (positive)	HsTrx2:ASP60:OD2 (negative)	4,11318
P08559:ARG314:NH2 - HsTrx2:ASP61:OD2	Electrostatic (attractive charge)	P08559:ARG314:NH2 (positive)	HsTrx2:ASP61:OD2 (negative)	4,82027
HsTrx2:GLY8:HN - P08559:LEU47:O	Hydrogen Bond (conventional)	HsTrx2:GLY8:HN (H-donor)	P08559:LEU47:O (H-acceptor)	2,555
HsTrx2:THR63:HG1	Hydrogen Bond	HsTrx2:THR63:HG1	P08559:ARG314:O	2,73796

- P08559:ARG314:O	(conventional)	(H-donor)	(H-acceptor)	
P08559:ARG44:HH21 - HsTrx2:ASP61:O	Hydrogen Bond (conventional)	P08559:ARG44:HH21 (H-donor)	HsTrx2:ASP61:O (H-acceptor)	2,69178
P08559:GLN51:HE21 - HsTrx2:HIS62:O	Hydrogen Bond (conventional)	P08559:GLN51:HE21 (H-donor)	HsTrx2:HIS62:O (H-acceptor)	2,93454
P08559:LYS54:HZ2 - HsTrx2:GLY8:O	Hydrogen Bond (conventional)	P08559:LYS54:HZ2 (H-donor)	HsTrx2:GLY8:O (H-acceptor)	2,9115
P08559:ARG44:CD - HsTrx2:ASP61:O	Hydrogen Bond (carbon)	P08559:ARG44:CD (H-donor)	HsTrx2:ASP61:O (H-acceptor)	3,6459
P08559:LYS48:CE - HsTrx2:THR63:OG1	Hydrogen Bond (carbon)	P08559:LYS48:CE (H-donor)	HsTrx2:THR63:OG1 (H-acceptor)	2,35152
HsTrx2:PRO9 - P08559:MET265	Hydrophobic (alkyl)	HsTrx2:PRO9 (alkyl)	P08559:MET265 (alkyl)	5,33093
P08559:ALA321 - HsTrx2:ILE67	Hydrophobic (alkyl)	P08559:ALA321 (alkyl)	HsTrx2:ILE67 (alkyl)	3,83145
HsTrx2:HIS62 - P08559:LEU47	Hydrophobic (Pi-alkyl)	HsTrx2:HIS62 (Pi-orbitals)	P08559:LEU47 (alkyl)	5,40894
P08559:HIS261 - HsTrx2:PRO9	Hydrophobic (Pi-alkyl)	P08559:HIS261 (Pi-orbitals)	HsTrx2:PRO9 (alkyl)	4,92286

HsTrx2 interacting residues	Electrostatic interaction	Hydrogen bonding	Salt bridge	Hydrophobic interaction	Other
**GLY8**	-	+	-	-	-
**PRO9**	-	-	-	+	-
**ASP58**	+	-	-	-	-
**ASP60**	+	-	-	-	-
**ASP61**	+	+	+	-	-
**HIS62**	-	+	-	+	-
**THR63**	-	+	-	-	-
**ILE67**	-	-	-	+	-

1.14. Pyruvate dehydrogenase E1 component subunit beta, mitochondrial (UniProt KB: P11177):

Interacting residues (P11177-HsTrx2)	Category (type)	From (chemistry)	To (chemistry)	Distance (Å)
P11177:LYS255:HZ2 - HsTrx2:ASP60:OD1	Hydrogen Bond; Electrostatic (salt bridge)	P11177:LYS255:HZ2 (H-donor; positive)	HsTrx2:ASP60:OD1 (H- acceptor; negative)	2,89457
P11177:LYS255:HZ3 - HsTrx2:ASP60:OD1	Hydrogen Bond; Electrostatic (salt bridge)	P11177:LYS255:HZ3 (H-donor; positive)	HsTrx2:ASP60:OD1 (H-acceptor; negative)	2,82152
HsTrx2:SER72:HG - P11177:GLY230:O	Hydrogen Bond (conventional)	HsTrx2:SER72:HG (H-donor)	P11177:GLY230:O (H-acceptor)	2,36981
P11177:HIS206:HN - HsTrx2:SER72:O	Hydrogen Bond (conventional)	P11177:HIS206:HN (H-donor)	HsTrx2:SER72:O (H-acceptor)	2,8329
P11177:GLU232:HN - HsTrx2:SER72:O	Hydrogen Bond (conventional)	P11177:GLU232:HN (H-donor)	HsTrx2:SER72:O (H-acceptor)	1,65014
HsTrx2:THR63:CB - P11177:MET254:O	Hydrogen Bond (carbon)	HsTrx2:THR63:CB (H-donor)	P11177:MET254:O (H-acceptor)	2,57795

HsTrx2 interacting	Electrostatic	Hydrogen	Salt	Hydrophobic	Other

residues	interaction	bonding	bridge	interaction	
**ASP60**	+	+	+	-	-
**THR63**	-	+	-	-	-
**SER72**	-	+	-	-	-

1.15. Creatine kinase S-type, mitochondrial (UniProt KB: P17540):

Interacting residues (P17540-HsTrx2)	Category (type)	From (chemistry)	To (chemistry)	Distance (Å)
HsTrx2:LYS35:NZ - P17540:ASP88:OD1	Electrostatic (attractive charge)	HsTrx2:LYS35:NZ (positive)	P17540:ASP88:OD1 (negative)	3,11345
P17540:ASP96:OD2 - HsTrx2:TRP30	Electrostatic (Pi-anion)	P17540:ASP96:OD2 (negative)	HsTrx2:TRP30 (Pi-orbitals)	4,39254
P17540:ASP96:OD2 - HsTrx2:TRP30	Electrostatic (Pi-anion)	P17540:ASP96:OD2 (negative)	HsTrx2:TRP30 (Pi-orbitals)	4,73695
P17540:LEU47:HN - HsTrx2:GLU42:OE1	Hydrogen Bond (conventional)	P17540:LEU47:HN (H-donor)	HsTrx2:GLU42:OE1 (H-acceptor)	2,42065
HsTrx2:LYS35:HZ1 - P17540:ARG77:O	Hydrogen Bond (conventional)	HsTrx2:LYS35:HZ1 (H-donor)	P17540:ARG77:O (H-acceptor)	2,6652
HsTrx2:TRP30:CD1 - P17540:THR237:O	Hydrogen Bond (carbon)	HsTrx2:TRP30:CD1 (H-donor)	P17540:THR237:O (H-acceptor)	2,79653
P17540:CYS238:SG - HsTrx2:TRP30	Other (Pi-sulfur)	P17540:CYS238:SG (sulfur)	HsTrx2:TRP30 (Pi-orbitals)	4,02897
P17540:CYS238:SG - HsTrx2:TRP30	Other (Pi-sulfur)	P17540:CYS238:SG (sulfur)	HsTrx2:TRP30 (Pi-orbitals)	4,57419
P17540:ARG46 - HsTrx2:PRO39	Hydrophobic (alkyl)	P17540:ARG46 (alkyl)	HsTrx2:PRO39 (alkyl)	3,52247
P17540:VAL80 - HsTrx2:ILE36	Hydrophobic (alkyl)	P17540:VAL80 (alkyl)	HsTrx2:ILE36 (alkyl)	4,60644
HsTrx2:ALA46 - P17540:LEU47	Hydrophobic (alkyl)	HsTrx2:ALA46 (alkyl)	P17540:LEU47 (alkyl)	4,90273

HsTrx2 interacting residues	Electrostatic interaction	Hydrogen bonding	Salt bridge	Hydrophobic interaction	Other
**TRP30**	+	+	-	-	+
**LYS35**	+	+	-	-	-
**ILE36**	-	-	-	+	-
**PRO39**	-	-	-	+	-
**GLU42**	-	+	-	-	-
**ALA46**	-	-	-	+	-

1.16. Cytochrome b-c1 complex subunit 2, mitochondrial (UniProt KB: P22695):

Interacting residues (P22695-HsTrx2)	Category (type)	From (chemistry)	To (chemistry)	Distance (Å)
HsTrx2:LYS35:NZ - P22695:TRP163	Electrostatic (Pi-cation)	HsTrx2:LYS35:NZ (positive)	P22695:TRP163 (Pi-orbitals)	4,22639
HsTrx2:LYS104:HZ2 -	Hydrogen Bond	HsTrx2:LYS104:HZ2	P22695:MET303:SD	2,63384

P22695:MET303:SD	(conventional)	(H-donor)	(H-acceptor)	
P22695:LEU292:HN - HsTrx2:ASP94:OD2	Hydrogen Bond (conventional)	P22695:LEU292:HN (H-donor)	HsTrx2:ASP94:OD2 (H-acceptor)	2,81432
HsTrx2:GLY32:CA - P22695:TRP163:O	Hydrogen Bond (carbon)	HsTrx2:GLY32:CA (H-donor)	P22695:TRP163:O (H-acceptor)	3,77281
HsTrx2:GLY32:CA - P22695:ILE164:O	Hydrogen Bond (carbon)	HsTrx2:GLY32:CA (H-donor)	P22695:ILE164:O (H-acceptor)	3,34934
P22695:PRO154:CD - HsTrx2:GLU95:OE1	Hydrogen Bond (carbon)	P22695:PRO154:CD (H-donor)	HsTrx2:GLU95:OE1 (H-acceptor)	2,96467
P22695:GLY289:CA - HsTrx2:ASP96:OD2	Hydrogen Bond (carbon)	P22695:GLY289:CA (H-donor)	HsTrx2:ASP96:OD2 (H-acceptor)	3,5895
HsTrx2:PRO33 - P22695:ILE164	Hydrophobic (alkyl)	HsTrx2:PRO33 (alkyl)	P22695:ILE164 (alkyl)	4,27509
HsTrx2:ILE36 - P22695:LEU150	Hydrophobic (alkyl)	HsTrx2:ILE36 (alkyl)	P22695:LEU150 (alkyl)	3,12197
HsTrx2:ILE36 - P22695:LEU160	Hydrophobic (alkyl)	HsTrx2:ILE36 (alkyl)	P22695:LEU160 (alkyl)	3,50128
HsTrx2:ILE36 - P22695:ILE164	Hydrophobic (alkyl)	HsTrx2:ILE36 (alkyl)	P22695:ILE164 (alkyl)	4,76288
HsTrx2:PRO39 - P22695:LEU160	Hydrophobic (alkyl)	HsTrx2:PRO39 (alkyl)	P22695:LEU160 (alkyl)	5,19805
HsTrx2:ARG40 - P22695:ILE153	Hydrophobic (alkyl)	HsTrx2:ARG40 (alkyl)	P22695:ILE153 (alkyl)	4,17101
HsTrx2:ARG40 - P22695:LEU160	Hydrophobic (alkyl)	HsTrx2:ARG40 (alkyl)	P22695:LEU160 (alkyl)	5,11556
HsTrx2:LYS93 - P22695:LEU295	Hydrophobic (alkyl)	HsTrx2:LYS93 (alkyl)	P22695:LEU295 (alkyl)	4,57826
P22695:TRP163 - HsTrx2:LYS35	Hydrophobic (Pi-aklyl)	P22695:TRP163 (Pi-orbitals)	HsTrx2:LYS35 (alkyl)	4,26079
P22695:TRP163 - HsTrx2:LYS35	Hydrophobic (Pi-aklyl)	P22695:TRP163 (Pi-orbitals)	HsTrx2:LYS35 (alkyl)	4,74924
P22695:PHE181 - HsTrx2:ALA73	Hydrophobic (Pi-aklyl)	P22695:PHE181 (Pi-orbitals)	HsTrx2:ALA73 (alkyl)	5,07681

HsTrx2 interacting residues	Electrostatic interaction	Hydrogen bonding	Salt bridge	Hydrophobic interaction	Other
**GLY32**	-	+	-	-	-
**PRO33**	-	-	-	+	-
**LYS35**	+	-	-	+	-
**ILE36**	-	-	-	+	-
**PRO39**	-	-	-	+	-
**ARG40**	-	-	-	+	-
**ALA73**	-	-	-	+	-
**LYS93**	-	-	-	+	-
**ASP94**	-	+	-	-	-
**GLU95**	-	+	-	-	-
**ASP96**	-	+	-	-	-

**LYS104**	-	+	-	-	-

1.17. ATP synthase subunit alpha, mitochondrial (UniProt KB: P25705):

Interacting residues (P25705-HsTrx2)	Category (type)	From (chemistry)	To (chemistry)	Distance (Å)
P25705:LYS123:HZ3 - HsTrx2:GLU70:OE2	Hydrogen Bond; Electrostatic (salt bridge)	P25705:LYS123:HZ3 (H-donor; positive)	HsTrx2:GLU70:OE2 (H-acceptor; negative)	3,10475
P25705:ARG214:NH2 - HsTrx2:ASP94:OD2	Electrostatic (attractive charge)	P25705:ARG214:NH2 (positive)	HsTrx2:ASP94:OD2 (negative)	5,4931
P25705:ASP79:HN - HsTrx2:THR63:O	Hydrogen Bond (conventional)	P25705:ASP79:HN (H-donor)	HsTrx2:THR63:O (H-acceptor)	1,78051
P25705:LYS123:HN - HsTrx2:ILE67:O	Hydrogen Bond (conventional)	P25705:LYS123:HN (H-donor)	HsTrx2:ILE67:O (H-acceptor)	2,21046
P25705:LYS123:HZ2 - HsTrx2:GLU68:O	Hydrogen Bond (conventional)	P25705:LYS123:HZ2 (H-donor)	HsTrx2:GLU68:O (H-acceptor)	2,81884
P25705:ALA283:HN - HsTrx2:SER72:O	Hydrogen Bond (conventional)	P25705:ALA283:HN (H-donor)	HsTrx2:SER72:O (H-acceptor)	2,50805
P25705:LYS252:CE - HsTrx2:VAL90:O	Hydrogen Bond (carbon)	P25705:LYS252:CE (H-donor)	HsTrx2:VAL90:O (H-acceptor)	3,77689
P25705:ILE77 - HsTrx2:ILE67	Hydrophobic (aklyl)	P25705:ILE77 (alkyl)	HsTrx2:ILE67 (alkyl)	4,52286
P25705:ALA279 - HsTrx2:VAL90	Hydrophobic (aklyl)	P25705:ALA279 (alkyl)	HsTrx2:VAL90 (alkyl)	3,7217
HsTrx2:CYS31 - P25705:VAL319	Hydrophobic (aklyl)	HsTrx2:CYS31 (alkyl)	P25705:VAL319 (alkyl)	5,30421
HsTrx2:TRP30 - P25705:ARG322	Hydrophobic (Pi-alkyl)	HsTrx2:TRP30 (Pi-orbitals)	P25705:ARG322 (alkyl)	5,02186
HsTrx2:TRP30 - P25705:LEU326	Hydrophobic (Pi-alkyl)	HsTrx2:TRP30 (Pi-orbitals)	P25705:LEU326 (alkyl)	4,44702
HsTrx2:TRP30 - P25705:LEU326	Hydrophobic (Pi-alkyl)	HsTrx2:TRP30 (Pi-orbitals)	P25705:LEU326 (alkyl)	4,34365

HsTrx2 interacting residues	Electrostatic interaction	Hydrogen bonding	Salt bridge	Hydrophobic interaction	Other
**TRP30**	-	-	-	+	-
**CYS31**	-	-	-	+	-
**THR63**	-	+	-	-	-
**ILE67**	-	+	-	+	-
**GLU68**	-	+	-	-	-
**GLU70**	+	+	+	-	-
**SER72**	-	+	-	-	-
**VAL90**	-	+	-	+	-
**ASP94**	+	-	-	-	-

1.18 Peroxiredoxin-6 (UniProt KB: P30041):

Interacting residues (P30041-HsTrx2)	Category (type)	From (chemistry)	To (chemistry)	Distance (Å)
HsTrx2:LYS104:HZ2 - P30041:GLU159:OE2	Hydrogen Bond; Electrostatic (salt bridge)	HsTrx2:LYS104:HZ2 (H-donor; positive)	P30041:GLU159:OE2 (H-acceptor; negative)	3,10538
P30041:LEU7:HN - HsTrx2:ILE92:O	Hydrogen Bond (conventional)	P30041:LEU7:HN (H-donor)	HsTrx2:ILE92:O (H-acceptor)	2,97018
P30041:ASP180:HN - HsTrx2:LYS103:O	Hydrogen Bond (conventional)	P30041:ASP180:HN (H-donor)	HsTrx2:LYS103:O (H-acceptor)	1,92499
HsTrx2:GLN97:HE22 - P30041:LYS144:O	Hydrogen Bond (conventional)	HsTrx2:GLN97:HE22 (H-donor)	P30041:LYS144:O (H-acceptor)	2,93269
P30041:ILE147:CA - HsTrx2:LYS88:O	Hydrogen Bond (carbon)	P30041:ILE147:CA (H-donor)	HsTrx2:LYS88:O (H-acceptor)	2,85627
HsTrx2:ASP87:CA - P30041:TYR149:OH	Hydrogen Bond (carbon)	HsTrx2:ASP87:CA (H-donor)	P30041:TYR149:OH (H-acceptor)	3,2734
P30041:VAL163 - HsTrx2:LYS104	Hydrophobic (alkyl)	P30041:VAL163 (alkyl)	HsTrx2:LYS104 (alkyl)	5,47215
P30041:VAL179 - HsTrx2:ILE106	Hydrophobic (alkyl)	P30041:VAL179 (alkyl)	HsTrx2:ILE106 (alkyl)	5,12888
HsTrx2:LYS88 - P30041:LEU148	Hydrophobic (alkyl)	HsTrx2:LYS88 (alkyl)	P30041:LEU148 (alkyl)	4,68147
HsTrx2:LYS93 - P30041:LEU7	Hydrophobic (alkyl)	HsTrx2:LYS93 (alkyl)	P30041:LEU7 (alkyl)	5,10823
P30041:TYR217 - HsTrx2:LYS47	Hydrophobic (Pi-alkyl)	P30041:TYR217 (Pi-orbitals)	HsTrx2:LYS47 (alkyl)	5,48537

HsTrx2 interacting residues	Electrostatic interaction	Hydrogen bonding	Salt bridge	Hydrophobic interaction	Other
**LYS47**	-	-	-	+	-
**ASP87**	-	+	-	-	-
**LYS88**	-	+	-	+	-
**ILE92**	-	+	-	-	-
**LYS93**	-	-	-	+	-
**GLN97**	-	+	-	-	-
**LYS103**	-	+	-	-	-
**LYS104**	+	+	+	+	-
**ILE106**	-	-	-	+	-

1.19. Peroxiredoxin-5 (UniProt KB: P30044):

Interacting residues (P30044-HsTrx2)	Category (type)	From (chemistry)	To (chemistry)	Distance (Å)
P30044:LYS63:HZ3 - HsTrx2:ASP64:OD2	Hydrogen Bond; Electrostatic (salt bridge)	P30044:LYS63:HZ3 (H-donor; positive)	HsTrx2:ASP64:OD2 (H-acceptor; negative)	2,51801
P30044:ARG86:NH1 - HsTrx2:GLU70:OE2	Electrostatic (attractive charge)	P30044:ARG86:NH1 (positive)	HsTrx2:GLU70:OE2 (negative)	4,08172
P30044:GLN68:HE22 -	Hydrogen Bond	P30044:GLN68:HE22	HsTrx2:ASP61:O	2,87127

HsTrx2:ASP61:O	(conventional)	(H-donor)	(H-acceptor)	
P30044:VAL23:CA - HsTrx2:SER72:O	Hydrogen Bond (carbon)	P30044:VAL23:CA (H-donor)	HsTrx2:SER72:O (H-acceptor)	3,0952
P30044:VAL69 - HsTrx2:ILE67	Hydrophobic (alkyl)	P30044:VAL69 (alkyl)	HsTrx2:ILE67 (alkyl)	5,19021
P30044:ARG95 - HsTrx2:ILE67	Hydrophobic (alkyl)	P30044:ARG95 (alkyl)	HsTrx2:ILE67 (alkyl)	3,71153
HsTrx2:TRP30 - P30044:LYS30	Hydrophobic (Pi-alkyl)	HsTrx2:TRP30 (Pi-orbitals)	P30044:LYS30 (alkyl)	3,48566
HsTrx2:TRP30 - P30044:LYS30	Hydrophobic (Pi-alkyl)	HsTrx2:TRP30 (Pi-orbitals)	P30044:LYS30 (alkyl)	4,86139

HsTrx2 interacting residues	Electrostatic interaction	Hydrogen bonding	Salt bridge	Hydrophobic interaction	Other
**TRP30**	-	-	-	+	-
**ASP61**	-	+	-	-	-
**ASP64**	+	+	+	-	-
**ILE67**	-	-	-	+	-
**GLU70**	+	-	-	-	-
**SER72**	-	+	-	-	-

1.20. Thioredoxin-dependent peroxide reductase, mitochondrial (Peroxiredoxin-3) (UniProt KB: P30048):

Interacting residues (P30048-HsTrx2)	Category (type)	From (chemistry)	To (chemistry)	Distance (Å)
P30048:ARG123:NH2 - HsTrx2:TRP30	Electrostatic (Pi-cation)	P30048:ARG123:NH2 (positive)	HsTrx2:TRP30 (Pi-orbitals)	4,30967
P30048:ARG123:NH2 - HsTrx2:TRP30	Electrostatic (Pi-cation)	P30048:ARG123:NH2 (positive)	HsTrx2:TRP30 (Pi-orbitals)	4,15051
HsTrx2:SER72:HG - P30048:ASN89:O	Hydrogen Bond (conventional)	HsTrx2:SER72:HG (H-donor)	P30048:ASN89:O (H-acceptor)	1,67115
P30048:TRP82:HE1 - HsTrx2:SER72:O	Hydrogen Bond (conventional)	P30048:TRP82:HE1 (H-donor)	HsTrx2:SER72:O (H-acceptor)	2,64148
HsTrx2:ALA73:CA - P30048:THR44:O	Hydrogen Bond (carbon)	HsTrx2:ALA73:CA (H-donor)	P30048:THR44:O (H-acceptor)	2,5035
P30048:PRO143:CA - HsTrx2:TRP30:O	Hydrogen Bond (carbon)	P30048:PRO143:CA (H-donor)	HsTrx2:TRP30:O (H-acceptor)	2,51657
HsTrx2:TRP30 - P30048:PRO40	Hydrophobic (Pi-alkyl)	HsTrx2:TRP30 (Pi-orbitals)	P30048:PRO40 (alkyl)	4,95643
HsTrx2:TRP30 - P30048:LEU142	Hydrophobic (Pi-alkyl)	HsTrx2:TRP30 (Pi-orbitals)	P30048:LEU142 (alkyl)	4,83636
P30048:PHE45 - HsTrx2:PRO33	Hydrophobic (Pi-alkyl)	P30048:PHE45 (Pi-orbitals)	HsTrx2:PRO33 (alkyl)	4,9569
HsTrx2:CYS31:SG - P30048:PHE45	Other (Pi-sulfur)	HsTrx2:CYS31:SG (sulfur)	P30048:PHE45 (Pi-orbitals)	3,8226

HsTrx2 interacting residues	Electrostatic interaction	Hydrogen bonding	Salt bridge	Hydrophobic interaction	Other
**TRP30**	+	+	-	+	-

**CYS31**	-	-	-	-	+
**PRO33**	-	-	-	+	-
**SER72**	-	+	-	-	-
**ALA73**	-	+	-	-	-

1.21. Cytochrome b-c1 complex subunit 1, mitochondrial (UniProt KB: P31930):

Interacting residues (P31930-HsTrx2)	Category (type)	From (chemistry)	To (chemistry)	Distance (Å)
HsTrx2:ARG40:HH12 - P31930:ASP349:OD2	Hydrogen Bond; Electrostatic (salt bridge)	HsTrx2:ARG40:HH12 (H-donor; positive)	P31930:ASP349:OD2 (H-acceptor; negative)	2,70561
HsTrx2:LYS35:NZ - P31930:GLU452:OE1	Electrostatic (attractive charge)	HsTrx2:LYS35:NZ (positive)	P31930:GLU452:OE1 (negative)	5,30399
HsTrx2:GLY32:HN - P31930:GLU452:O	Hydrogen Bond (conventional)	HsTrx2:GLY32:HN (H-donor)	P31930:GLU452:O (H-acceptor)	1,82879
HsTrx2:ARG40:HH22 - P31930:THR345:O	Hydrogen Bond (conventional)	HsTrx2:ARG40:HH22 (H-donor)	P31930:THR345:O (H-acceptor)	1,948
HsTrx2:LYS93:HZ2 - P31930:LYS315:O	Hydrogen Bond (conventional)	HsTrx2:LYS93:HZ2 (H-donor)	P31930:LYS315:O (H-acceptor)	2,62133
HsTrx2:LYS93:HZ3 - P31930:LYS315:O	Hydrogen Bond (conventional)	HsTrx2:LYS93:HZ3 (H-donor)	P31930:LYS315:O (H-acceptor)	2,99496
P31930:GLN318:HE21 - HsTrx2:ASP96:OD2	Hydrogen Bond (conventional)	P31930:GLN318:HE21 (H-donor)	HsTrx2:ASP96:OD2 (H-acceptor)	2,60586
P31930:GLN318:HE22 - HsTrx2:ASP94:OD2	Hydrogen Bond (conventional)	P31930:GLN318:HE22 (H-donor)	HsTrx2:ASP94:OD2 (H-acceptor)	2,90302
P31930:LEU453:HA - HsTrx2:TRP30:O	Hydrogen Bond (carbon)	P31930:LEU453:HA (H-donor)	HsTrx2:TRP30:O (H-acceptor)	3,08547
HsTrx2:PRO33 - P31930:LYS352	Hydrophobic (alkyl)	HsTrx2:PRO33 (alkyl)	P31930:LYS352 (alkyl)	3,42318
P31930:HIS446 - HsTrx2:ILE36	Hydrophobic (Pi-alkyl)	P31930:HIS446 (Pi-orbitals)	HsTrx2:ILE36 (alkyl)	4,67319

HsTrx2 interacting residues	Electrostatic interaction	Hydrogen bonding	Salt bridge	Hydrophobic interaction	Other
**TRP30**	-	+	-	-	-
**GLY32**	-	+	-	-	-
**PRO33**	-	-	-	+	-
**LYS35**	+	-	-	+	-
**ILE36**	-	-	-	+	-
**ARG40**	+	+	+	-	-
**LYS93**	-	+	-	-	-
**ASP94**	-	+	-	-	-
**ASP96**	-	+	-	-	-

1.22. Peroxiredoxin-2 (UniProt KB: P32119):

Interacting residues (P32119-HsTrx2)	Category (type)	From (chemistry)	To (chemistry)	Distance (Å)
HsTrx2:LYS104:NZ - P32119:GLU154:OE2	Electrostatic (attractive charge)	HsTrx2:LYS104:NZ (positive)	P32119:GLU154:OE2 (negative)	4,27304
P32119:SER3:HN - HsTrx2:VAL71:O	Hydrogen Bond (conventional)	P32119:SER3:HN (H-donor)	HsTrx2:VAL71:O (H-acceptor)	2,12161
P32119:ASN144:HD22 - HsTrx2:VAL85:O	Hydrogen Bond (conventional)	P32119:ASN144:HD22 (H-donor)	HsTrx2:VAL85:O (H-acceptor)	2,18193
P32119:GLY175:HN - HsTrx2:ALA100:O	Hydrogen Bond (conventional)	P32119:GLY175:HN (H-donor)	HsTrx2:ALA100:O (H-acceptor)	2,99758
HsTrx2:SER72:HG - P32119:TYR115:O	Hydrogen Bond (conventional)	HsTrx2:SER72:HG (H-donor)	P32119:TYR115:O (H-acceptor)	2,41153
HsTrx2:LYS93:HZ1 - P32119:ARG139:O	Hydrogen Bond (conventional)	HsTrx2:LYS93:HZ1 (H-donor)	P32119:ARG139:O (H-acceptor)	3,0633
P32119:ALA2 - HsTrx2:ILE59	Hydrophobic (alkyl)	P32119:ALA2 (alkyl)	HsTrx2:ILE59 (alkyl)	4,10008
P32119:ALA2 - HsTrx2:ALA66	Hydrophobic (alkyl)	P32119:ALA2 (alkyl)	HsTrx2:ALA66 (alkyl)	3,93138
P32119:ALA2 - HsTrx2:VAL71	Hydrophobic (alkyl)	P32119:ALA2 (alkyl)	HsTrx2:VAL71 (alkyl)	3,91066
P32119:VAL143 - HsTrx2:LYS88	Hydrophobic (alkyl)	P32119:VAL143 (alkyl)	HsTrx2:LYS88 (alkyl)	5,28751
P32119:ALA174 - HsTrx2:ALA100	Hydrophobic (alkyl)	P32119:ALA174 (alkyl)	HsTrx2:ALA100 (alkyl)	3,75016
P32119:ALA174 - HsTrx2:LYS103	Hydrophobic (alkyl)	P32119:ALA174 (alkyl)	HsTrx2:LYS103 (alkyl)	3,06922
HsTrx2:ALA73 - P32119:ARG7	Hydrophobic (alkyl)	HsTrx2:ALA73 (alkyl)	P32119:ARG7 (alkyl)	4,10281
HsTrx2:LYS88 - P32119:ILE141	Hydrophobic (alkyl)	HsTrx2:LYS88 (alkyl)	P32119:ILE141 (alkyl)	5,49295
HsTrx2:VAL90 - P32119:ILE8	Hydrophobic (alkyl)	HsTrx2:VAL90 (alkyl)	P32119:ILE8 (alkyl)	5,31201
HsTrx2:VAL90 - P32119:ILE141	Hydrophobic (alkyl)	HsTrx2:VAL90 (alkyl)	P32119:ILE141 (alkyl)	3,65444

HsTrx2 interacting residues	Electrostatic interaction	Hydrogen bonding	Salt bridge	Hydrophobic interaction	Other
**ILE59**	-	-	-	+	-
**ALA66**	-	-	-	+	-
**VAL71**	-	+	-	+	-
**SER72**	-	+	-	-	-
**ALA73**	-	-	-	+	-
**VAL85**	-	+	-	-	-
**LYS88**	-	-	-	+	-
**VAL90**	-	-	-	+	-
**LYS93**	-	+	-	-	-

**ALA100**	-	+	-	+	-
**LYS103**	-	-	-	+	-
**LYS104**	+	+	+	-	-

1.23. Stress-70 protein, mitochondrial (UniProt KB: P38646):

Interacting residues (P38646-HsTrx2)	Category (type)	From (chemistry)	To (chemistry)	Distance (Å)
P38646:ARG513:HH21 – HsTrx2:ASP96:OD1	Hydrogen Bond; Electrostatic (salt bridge)	P38646:ARG513:HH21 (H-donor; positive)	HsTrx2:ASP96:OD1 (H-acceptor; negative)	3,08509
HsTrx2:LYS51:NZ – P38646:GLU448:OE1	Electrostatic (attractive charge)	HsTrx2:LYS51:NZ (positive)	P38646:GLU448:OE1 (negative)	4,90827
HsTrx2:LYS103:NZ – P38646:ASP477:OD1	Electrostatic (attractive charge)	HsTrx2:LYS103:NZ (positive)	P38646:ASP477:OD1 (negative)	2,99585
P38646:ARG513:NH1 – HsTrx2:GLU99:OE2	Electrostatic (attractive charge)	P38646:ARG513:NH1 (positive)	HsTrx2:GLU99:OE2 (negative)	3,33385
P38646:ARG513:NH2 – HsTrx2:GLU99:OE1	Electrostatic (attractive charge)	P38646:ARG513:NH2 (positive)	HsTrx2:GLU99:OE1 (negative)	3,25126
P38646:ARG513:HH21 – HsTrx2:ASP96:O	Hydrogen Bond (conventional)	P38646:ARG513:HH21 (H-donor)	HsTrx2:ASP96:O (H-acceptor)	2,7713
P38646:ARG513:HH22 – HsTrx2:ASP96:O	Hydrogen Bond (conventional)	P38646:ARG513:HH22 (H-donor)	HsTrx2:ASP96:O (H-acceptor)	2,67452
P38646:ALA476 – HsTrx2:LYS103	Hydrophobic (alkyl)	P38646:ALA476 (alkyl)	HsTrx2:LYS103 (alkyl)	4,74825
HsTrx2:HIS49 – P38646:VAL453	Hydrophobic (Pi-alkyl)	HsTrx2:HIS49 (Pi-orbitals)	P38646:VAL453 (alkyl)	4,06725
HsTrx2:HIS49 – P38646:MET493	Hydrophobic (Pi-alkyl)	HsTrx2:HIS49 (Pi-orbitals)	P38646:MET493 (alkyl)	4,80945
P38646:MET493:SD – HsTrx2:HIS49:ND1	Other (Sulfur-X, X:O,N,S)	P38646:MET493:SD (sulfur)	HsTrx2:HIS49:ND1 (N)	3,06988

HsTrx2 interacting residues	Electrostatic interaction	Hydrogen bonding	Salt bridge	Hydrophobic interaction	Other
**HIS49**	-	-	-	+	+
**LYS51**	+	-	-	-	-
**ASP96**	+	+	+	-	-
**GLU99**	+	-	-	-	-
**LYS103**	+	-	-	+	-

1.24. Trifunctional enzyme subunit alpha, mitochondrial (UniProt KB: P40939):

Interacting residues (P40939-HsTrx2)	Category (type)	From (chemistry)	To (chemistry)	Distance (Å)
P40939:LYS440:HZ2 - HsTrx2:ASP96:OD2	Hydrogen Bond; Electrostatic (salt bridge)	P40939:LYS440:HZ2 (H-donor; positive)	HsTrx2:ASP96:OD2 (H-acceptor; negative)	3,18655
P40939:LYS440:HZ3 - HsTrx2:ASP94:OD1	Hydrogen Bond; Electrostatic (salt bridge)	P40939:LYS440:HZ3 (H-donor; positive)	HsTrx2:ASP94:OD1 (H-acceptor; negative)	2,42306

P40939:LYS386:NZ - HsTrx2:ASP94:OD2	Electrostatic (attractive charge)	P40939:LYS386:NZ (positive)	HsTrx2:ASP94:OD2 (negative)	5,45945
P40939:LYS386:HZ1 - HsTrx2:ILE92:O	Hydrogen Bond (conventional)	P40939:LYS386:HZ1 (H-donor)	HsTrx2:ILE92:O (H-acceptor)	2,63775
P40939:LYS386:HZ3 - HsTrx2:ILE92:O	Hydrogen Bond (conventional)	P40939:LYS386:HZ3 (H-donor)	HsTrx2:ILE92:O (H-acceptor)	2,91317
P40939:GLY431:HN - HsTrx2:LYS88:O	Hydrogen Bond (conventional)	P40939:GLY431:HN (H-donor)	HsTrx2:LYS88:O (H-acceptor)	2,44279
HsTrx2:LYS88:HN - P40939:GLY431:O	Hydrogen Bond (conventional)	HsTrx2:LYS88:HN (H-donor)	P40939:GLY431:O (H-acceptor)	2,53533
P40939:PHE426 - HsTrx2:ALA73	Hydrophobic (alkyl)	P40939:PHE426 (Pi-orbitals)	HsTrx2:ALA73 (alkyl)	4,88226
P40939:PHE426 - HsTrx2:VAL90	Hydrophobic (alkyl)	P40939:PHE426 (Pi-orbitals)	HsTrx2:VAL90 (alkyl)	4,87315

HsTrx2 interacting residues	Electrostatic interaction	Hydrogen bonding	Salt bridge	Hydrophobic interaction	Other
**ALA73**	-	-	-	+	-
**LYS88**	-	+	-	-	-
**VAL90**	-	-	-	+	-
**ILE92**	-	+	-	-	-
**ASP94**	+	+	+	-	-
**ASP96**	+	+	+	-	-

1.25. Isocitrate dehydrogenase [NADP], mitochondrial (UniProt KB: P48735):

Interacting residues (P48735-HsTrx2)	Category (type)	From (chemistry)	To (chemistry)	Distance (Å)
HsTrx2:ARG40:HH12 – P48735:ASP292:OD2	Hydrogen Bond; Electrostatic (salt bridge)	HsTrx2:ARG40:HH12 (H-donor; positive)	P48735:ASP292:OD2 (H-acceptor; negative)	3,24195
P48735:LYS256:HZ3 – HsTrx2:GLU95:OE1	Hydrogen Bond; Electrostatic (salt bridge)	P48735:LYS256:HZ3 (H-donor; positive)	HsTrx2:GLU95:OE1 (H-acceptor; negative)	2,98651
HsTrx2:LYS93:NZ – P48735:GLU286:OE1	Electrostatic (attractive charge)	HsTrx2:LYS93:NZ (positive)	P48735:GLU286:OE1 (negative)	4,77217
P48735:LYS263:NZ – HsTrx2:ASP94:OD2	Electrostatic (attractive charge)	P48735:LYS263:NZ (positive)	HsTrx2:ASP94:OD2 (negative)	3,81042
HsTrx2:GLN97:HE21 – P48735:GLN267:OE1	Hydrogen Bond (conventional)	HsTrx2:GLN97:HE21 (H-donor)	P48735:GLN267:OE1 (H-acceptor)	1,48952
P48735:ASN252:HN – HsTrx2:ASP94:OD2	Hydrogen Bond (conventional)	P48735:ASN252:HN (H-donor)	HsTrx2:ASP94:OD2 (H-acceptor)	2,45151
P48735:GLY260:CA – HsTrx2:ASP96:OD2	Hydrogen Bond (carbon)	P48735:GLY260:CA (H-donor)	HsTrx2:ASP96:OD2 (H-acceptor)	2,65488
P48735:LYS263:CE – HsTrx2:ASP94:OD2	Hydrogen Bond (carbon)	P48735:LYS263:CE (H-donor)	HsTrx2:ASP94:OD2 (H-acceptor)	3,04341
P48735:ARG288 – HsTrx2:ILE92	Hydrophobic (alkyl)	P48735:ARG288 (alkyl)	HsTrx2:ILE92 (alkyl)	4,88117

HsTrx2 interacting residues	Electrostatic interaction	Hydrogen bonding	Salt bridge	Hydrophobic interaction	Other
**ARG40**	+	+	+	-	-
**ILE92**	-	-	-	+	-
**LYS93**	+	-	-	-	-
**ASP94**	+	+	-	-	-
**GLU95**	+	+	+	-	-
**ASP96**	-	+	-	-	-
**GLN97**	-	+	-	-	-

1.26. Ribose 5-phosphate isomerase A (UniProt KB: P49247):

Interacting residues (P49247-HsTrx2)	Category (type)	From (chemistry)	To (chemistry)	Distance (Å)
HsTrx2:ARG40:NH2 - P49247:GLU154:OE1	Electrostatic (attractive charge)	HsTrx2:ARG40:NH2 (positive)	P49247:GLU154:OE1 (negative)	4,44027
HsTrx2:THR1:HT3 - P49247:GLN2:OE1	Hydrogen Bond (conventional)	HsTrx2:THR1:HT3 (H-donor)	P49247:GLN2:OE1 (H-acceptor)	1,84691
HsTrx2:THR2:HG1 - P49247:GLN2:O	Hydrogen Bond (conventional)	HsTrx2:THR2:HG1 (H-donor)	P49247:GLN2:O (H-acceptor)	2,73757
HsTrx2:LYS56:HZ1 - P49247:ARG3:O	Hydrogen Bond (conventional)	HsTrx2:LYS56:HZ1 (H-donor)	P49247:ARG3:O (H-acceptor)	2,31834
HsTrx2:LYS56:HZ3 - P49247:ARG3:O	Hydrogen Bond (conventional)	HsTrx2:LYS56:HZ3 (H-donor)	P49247:ARG3:O (H-acceptor)	2,74656
HsTrx2:SER72:HG - P49247:VAL281:O	Hydrogen Bond (conventional)	HsTrx2:SER72:HG (H-donor)	P49247:VAL281:O (H-acceptor)	1,71606
HsTrx2:LYS88:HZ3 - P49247:MET277:SD	Hydrogen Bond (conventional)	HsTrx2:LYS88:HZ3 (H-donor)	P49247:MET277:SD (H-acceptor)	2,56017
HsTrx2:PRO75:CD - P49247:TYR188:OH	Hydrogen Bond (carbon)	HsTrx2:PRO75:CD (H-donor)	P49247:TYR188:OH (H-acceptor)	2,90475
P49247:GLY5:CA - HsTrx2:ASN4:OD1	Hydrogen Bond (carbon)	P49247:GLY5:CA (H-donor)	HsTrx2:ASN4:OD1 (H-acceptor)	2,44527
HsTrx2:LYS88 - P49247:MET277	Hydrophobic (alkyl)	HsTrx2:LYS88 (alkyl)	P49247:MET277 (alkyl)	5,221
P49247:PHE7 - HsTrx2:LYS35	Hydrophobic (Pi-alkyl)	P49247:PHE7 (Pi-orbitals)	HsTrx2:LYS35 (alkyl)	5,10217
P49247:TYR188 - HsTrx2:PRO33	Hydrophobic (Pi-alkyl)	P49247:TYR188 (Pi-orbitals)	HsTrx2:PRO33 (alkyl)	3,9292
P49247:TYR188 - HsTrx2:ILE92	Hydrophobic (Pi-alkyl)	P49247:TYR188 (Pi-orbitals)	HsTrx2:ILE92 (alkyl)	3,9143
P49247:MET277:SD - HsTrx2:LYS88:NZ	Other (sulfur-X, X:O,N,S)	P49247:MET277:SD (sulfur)	HsTrx2:LYS88:NZ (N)	2,85794

HsTrx2 interacting residues	Electrostatic interaction	Hydrogen bonding	Salt bridge	Hydrophobic interaction	Other
**THR1**	-	+	-	-	-
**THR2**	-	+	-	-	-
**ASN4**	-	+	-	-	-

**PRO33**	-	-	-	+	-
**LYS35**	-	-	-	+	-
**ARG40**	+	+	+	-	-
**LYS56**	-	+	-	-	-
**SER72**	-	+	-	-	-
**PRO75**	-	+	-	-	-
**LYS88**	-	+	-	+	+
**ILE92**	-	-	-	+	-

1.27. Elongation factor Tu, mitochondrial, EF-Tu (UniProt KB: P49411):

Interacting residues (P49411-HsTrx2)	Category (type)	From (chemistry)	To (chemistry)	Distance (Å)
HsTrx2:VAL74:HN – P49411:THR439:O	Hydrogen Bond (conventional)	HsTrx2:VAL74:HN (H-donor)	P49411:THR439:O (H-acceptor)	2,31706
P49411:THR439:HG1 – HsTrx2:SER72:O	Hydrogen Bond (conventional)	P49411:THR439:HG1 (H-donor)	HsTrx2:SER72:O (H-acceptor)	2,08583
P49411:ALA441:HN – HsTrx2:CYS31:SG	Hydrogen Bond (conventional)	P49411:ALA441:HN (H-donor)	HsTrx2:CYS31:SG (H-acceptor)	2,43519
P49411:ALA441:HN – HsTrx2:VAL74:O	Hydrogen Bond (conventional)	P49411:ALA441:HN (H-donor)	HsTrx2:VAL74:O (H-acceptor)	2,29779
HsTrx2:CYS31 – P49411:LEU440	Hydrophobic (alkyl)	HsTrx2:CYS31 (alkyl)	P49411:LEU440 (alkyl)	4,03379
P49411:ALA441 – HsTrx2:CYS31	Hydrophobic (alkyl)	P49411:ALA441 (alkyl)	HsTrx2:CYS31 (alkyl)	5,23678
P49411:ALA441 – HsTrx2:PRO33	Hydrophobic (alkyl)	P49411:ALA441 (alkyl)	HsTrx2:PRO33 (alkyl)	3,11395
P49411:ALA441 – HsTrx2:PRO75	Hydrophobic (alkyl)	P49411:ALA441 (alkyl)	HsTrx2:PRO75 (alkyl)	3,53192

HsTrx2 interacting residues	Electrostatic interaction	Hydrogen bonding	Salt bridge	Hydrophobic interaction	Other
**CYS31**	-	+	-	+	-
**PRO33**	-	-	-	+	-
**SER72**	-	+	-	-	-
**VAL74**	-	+	-	-	-
**PRO75**	-	-	-	+	-

1.28. NADH dehydrogenase [ubiquinone] flavoprotein 1, mitochondrial (UniProt KB: P49821):

Interacting residues (P49821-HsTrx2)	Category (type)	From (chemistry)	To (chemistry)	Distance (Å)
P49821:ARG199:NH1 - HsTrx2:GLU70:OE1	Electrostatic (attractive charge)	P49821:ARG199:NH1 (positive)	HsTrx2:GLU70:OE1 (negative)	4,0584
P49821:VAL197:HN - HsTrx2:SER72:O	Hydrogen Bond (conventional)	P49821:VAL197:HN (H-donor)	HsTrx2:SER72:O (H-acceptor)	2,70311
P49821:ARG199:HH21 - HsTrx2:GLU70:O	Hydrogen Bond (conventional)	P49821:ARG199:HH21 (H-donor)	HsTrx2:GLU70:O (H-acceptor)	2,18875

HsTrx2:VAL90:HN - P49821:ILE216:O	Hydrogen Bond (conventional)	HsTrx2:VAL90:HN (H-donor)	P49821:ILE216:O (H-acceptor)	1,9164
P49821:ARG199:CD - HsTrx2:GLU70:O	Hydrogen Bond (carbon)	P49821:ARG199:CD (H-donor)	HsTrx2:GLU70:O (H-acceptor)	3,70532
P49821:GLY218:CA - HsTrx2:LYS88:O	Hydrogen Bond (carbon)	P49821:GLY218:CA (H-donor)	HsTrx2:LYS88:O (H-acceptor)	2,48179
HsTrx2:GLY91:CA - P49821:TYR155:OH	Hydrogen Bond (carbon)	HsTrx2:GLY91:CA (H-donor)	P49821:TYR155:OH (H-acceptor)	3,57862
P49821:ARG152 - HsTrx2:PRO33	Hydrophobic (alkyl)	P49821:ARG152 (alkyl)	HsTrx2:PRO33 (alkyl)	5,21781
P49821:VAL198 - HsTrx2:VAL90	Hydrophobic (alkyl)	P49821:VAL198 (alkyl)	HsTrx2:VAL90 (alkyl)	4,30659
HsTrx2:ALA73 - P49821:VAL197	Hydrophobic (alkyl)	HsTrx2:ALA73 (alkyl)	P49821:VAL197 (alkyl)	4,98336
HsTrx2:ALA73 - P49821:VAL198	Hydrophobic (alkyl)	HsTrx2:ALA73 (alkyl)	P49821:VAL198 (alkyl)	5,01602
P49821:PHE196 - HsTrx2:ALA73	Hydrophobic (Pi-alkyl)	P49821:PHE196 (Pi-orbitals)	HsTrx2:ALA73 (alkyl)	5,0561
P49821:PHE236 - HsTrx2:LYS93	Hydrophobic (Pi-alkyl)	P49821:PHE236 (Pi-orbitals)	HsTrx2:LYS93 (alkyl)	4,39057
HsTrx2:CYS31:SG - P49821:PHE196	Other (Pi-sulfur)	HsTrx2:CYS31:SG (sulfur)	P49821:PHE196 (Pi-orbitals)	3,78648
HsTrx2:ILE92:O - P49821:PHE236	Other (Pi-lone pair)	HsTrx2:ILE92:O (lone pair)	P49821:PHE236 (Pi-orbitals)	2,96185

HsTrx2 interacting residues	Electrostatic interaction	Hydrogen bonding	Salt bridge	Hydrophobic interaction	Other
**CYS31**	-	-	-	-	+
**PRO33**	-	-	-	+	-
**GLU70**	+	+	-	-	-
**SER72**	-	+	-	-	-
**ALA73**	-	-	-	+	-
**LYS88**	-	+	-	-	-
**VAL90**	-	+	-	+	-
**GLY91**	-	+	-	-	-
**ILE92**	-	-	-	-	+
**LYS93**	-	-	-	+	-

1.29. Isocitrate dehydrogenase [NAD] subunit alpha, mitochondrial (UniProt KB: P50213):

Interacting residues (P50213-HsTrx2)	Category (type)	From (chemistry)	To (chemistry)	Distance (Å)
HsTrx2:LYS35:NZ - P50213:GLU38:OE2	Electrostatic (attractive charge)	HsTrx2:LYS35:NZ (positive)	P50213:GLU38:OE2 (negative)	4,03068
P50213:TRP36:HN - HsTrx2:GLN29:OE1	Hydrogen Bond (conventional)	P50213:TRP36:HN (H-donor)	HsTrx2:GLN29:OE1 (H-acceptor)	2,56277
P50213:LYS25:HA - HsTrx2:TRP30:O	Hydrogen Bond (carbon)	P50213:LYS25:HA (H-donor)	HsTrx2:TRP30:O (H-acceptor)	2,59168

HsTrx2:TRP30 - P50213:TRP36	Hydrophobic (Pi-Pi stacked)	HsTrx2:TRP30 (Pi-orbitals)	P50213:TRP36 (Pi-orbitals)	3,22769
HsTrx2:TRP30 - P50213:TRP36	Hydrophobic (Pi-Pi stacked)	HsTrx2:TRP30 (Pi-orbitals)	P50213:TRP36 (Pi-orbitals)	3,20477
HsTrx2:TRP30 - P50213:TRP36	Hydrophobic (Pi-Pi stacked)	HsTrx2:TRP30 (Pi-orbitals)	P50213:TRP36 (Pi-orbitals)	4,70402
P50213:TRP36 - HsTrx2:TRP30	Hydrophobic (Pi-Pi stacked)	P50213:TRP36 (Pi-orbitals)	HsTrx2:TRP30 (Pi-orbitals)	4,60399
HsTrx2:TRP30 - P50213:PHE27	Hydrophobic (Pi-Pi T-shaped)	HsTrx2:TRP30 (Pi-orbitals)	P50213:PHE27 (Pi-orbitals)	5,60335
HsTrx2:TRP30 - P50213:ILE34	Hydrophobic (Pi-alkyl)	HsTrx2:TRP30 (Pi-orbitals)	P50213:ILE34 (alkyl)	4,89153
HsTrx2:TRP30 - P50213:ALA32	Hydrophobic (Pi-alkyl)	HsTrx2:TRP30 (Pi-orbitals)	P50213:ALA32 (alkyl)	4,41936
HsTrx2:TRP30 - P50213:ILE34	Hydrophobic (Pi-alkyl)	HsTrx2:TRP30 (Pi-orbitals)	P50213:ILE34 (alkyl)	4,05354

HsTrx2 interacting residues	Electrostatic interaction	Hydrogen bonding	Salt bridge	Hydrophobic interaction	Other
**GLN29**	-	+	-	-	-
**TRP30**	-	+	-	+	-
**LYS35**	+	-	-	-	-

1.30. Triosephosphate isomerase (UniProt KB: P60174):

Interacting residues (P60174-HsTrx2)	Category (type)	From (chemistry)	To (chemistry)	Distance (Å)
HsTrx2:LYS93:HZ3 - P60174:ASP49:OD2	Hydrogen Bond; Electrostatic (salt bridge)	HsTrx2:LYS93:HZ3 (H-donor; positive)	P60174:ASP49:OD2 (H-acceptor; negative)	2,02456
P60174:THR75:HN - HsTrx2:LYS104:O	Hydrogen Bond (conventional)	P60174:THR75:HN (H-donor)	HsTrx2:LYS104:O (H-acceptor)	2,25751
P60174:THR75:HG1 - HsTrx2:LEU105:O	Hydrogen Bond (conventional)	P60174:THR75:HG1 (H-donor)	HsTrx2:LEU105:O (H-acceptor)	2,89819
P60174:GLY76:HN - HsTrx2:ASP87:OD2	Hydrogen Bond (conventional)	P60174:GLY76:HN (H-donor)	HsTrx2:ASP87:OD2 (H-acceptor)	2,73021
HsTrx2:VAL90:HN - P60174:ALA46:O	Hydrogen Bond (conventional)	HsTrx2:VAL90:HN (H-donor)	P60174:ALA46:O (H-acceptor)	2,3907
HsTrx2:LYS93:HZ2 - P60174:CYS86:SG	Hydrogen Bond (conventional)	HsTrx2:LYS93:HZ2 (H-donor)	P60174:CYS86:SG (H-acceptor)	2,41942
HsTrx2:LYS93:HZ3 - P60174:CYS86:SG	Hydrogen Bond (conventional)	HsTrx2:LYS93:HZ3 (H-donor)	P60174:CYS86:SG (H-acceptor)	2,84018
HsTrx2:GLN97:HE22 - P60174:MET82:O	Hydrogen Bond (conventional)	HsTrx2:GLN97:HE22 (H-donor)	P60174:MET82:O (H-acceptor)	2,14091
P60174:ALA73:CA - HsTrx2:LYS103:O	Hydrogen Bond (carbon)	P60174:ALA73:CA (H-donor)	HsTrx2:LYS103:O (H-acceptor)	3,47018
P60174:GLY76:CA - HsTrx2:VAL86:O	Hydrogen Bond (carbon)	P60174:GLY76:CA (H-donor)	HsTrx2:VAL86:O (H-acceptor)	3,16741
HsTrx2:LEU105:CA - P60174:THR75:OG1	Hydrogen Bond (carbon)	HsTrx2:LEU105:CA (H-donor)	P60174:THR75:OG1 (H-acceptor)	2,91538

P60174:ALA46 - HsTrx2:VAL90	Hydrophobic (alkyl)	P60174:ALA46 (alkyl)	HsTrx2:VAL90 (alkyl)	4,49765
HsTrx2:ALA100 - P60174:MET82	Hydrophobic (alkyl)	HsTrx2:ALA100 (alkyl)	P60174:MET82 (alkyl)	3,74769
P60174:TYR47 - HsTrx2:VAL90	Hydrophobic (Pi-alkyl)	P60174:TYR47 (Pi-orbitals)	HsTrx2:VAL90 (alkyl)	4,605
P60174:PHE50 - HsTrx2:VAL90	Hydrophobic (Pi-alkyl)	P60174:PHE50 (Pi-orbitals)	HsTrx2:VAL90 (alkyl)	5,35846

HsTrx2 interacting residues	Electrostatic interaction	Hydrogen bonding	Salt bridge	Hydrophobic interaction	Other
**VAL86**	-	+	-	-	-
**ASP87**	-	+	-	-	-
**VAL90**	-	+	-	+	-
**LYS93**	+	+	+	-	-
**GLN97**	-	+	-	-	-
**ALA100**	-	-	-	+	-
**LYS103**	-	+	-	-	-
**LYS104**	-	+	-	-	-
**LEU105**	-	+	-	-	-

1.31. 40S ribosomal protein S18 (UniProt KB: P62269):

Interacting residues (P62269-HsTrx2)	Category (type)	From (chemistry)	To (chemistry)	Distanc e (Å)
P62269:LYS89:HZ1 - HsTrx2:GLU68:OE2	Hydrogen Bond; Electrostatic (salt bridge)	P62269:LYS89:HZ1 (H-donor; positive)	HsTrx2:GLU68:OE2 (H-acceptor; negative)	2,76599
P62269:ARG85:NH2 - HsTrx2:GLU68:OE2	Electrostatic (attractive charge)	P62269:ARG85:NH2 (positive)	HsTrx2:GLU68:OE2 (negative)	5,47011
P62269:ARG117:NH1 - HsTrx2:ASP87:OD1	Electrostatic (attractive charge)	P62269:ARG117:NH1 (positive)	HsTrx2:ASP87:OD1 (negative)	4,93351
P62269:ARG117:NH2 - HsTrx2:ASP87:OD2	Electrostatic (attractive charge)	P62269:ARG117:NH2 (positive)	HsTrx2:ASP87:OD2 (negative)	3,9893
P62269:LYS89:HZ2 - HsTrx2:GLU68:O	Hydrogen Bond (conventional)	P62269:LYS89:HZ2 (H-donor)	HsTrx2:GLU68:O (H-acceptor)	2,06532
P62269:LYS89:HZ3 - HsTrx2:GLU68:O	Hydrogen Bond (conventional)	P62269:LYS89:HZ3 (H-donor)	HsTrx2:GLU68:O (H-acceptor)	2,22265
P62269:ALA114:HN - HsTrx2:GLY107:OXT	Hydrogen Bond (conventional)	P62269:ALA114:HN (H-donor)	HsTrx2:GLY107:OX T (H-acceptor)	2,88525
P62269:ARG117:HH1 2 - HsTrx2:VAL86:O	Hydrogen Bond (conventional)	P62269:ARG117:HH1 2 (H-donor)	HsTrx2:VAL86:O (H-acceptor)	2,36886
HsTrx2:SER72:HG - P62269:ALA93:O	Hydrogen Bond (conventional)	HsTrx2:SER72:HG (H-donor)	P62269:ALA93:O (H-acceptor)	2,80398
HsTrx2:LYS88:HZ1 - P62269:ALA92:O	Hydrogen Bond (conventional)	HsTrx2:LYS88:HZ1 (H-donor)	P62269:ALA92:O (H-acceptor)	2,35874
HsTrx2:LYS88:HZ2 - P62269:ALA91:O	Hydrogen Bond (conventional)	HsTrx2:LYS88:HZ2 (H-donor)	P62269:ALA91:O (H-acceptor)	2,87691
HsTrx2:LYS88:HZ2 -	Hydrogen Bond	HsTrx2:LYS88:HZ2	P62269:ALA92:O	2,02827

P62269:ALA92:O	(conventional)	(H-donor)	(H-acceptor)	
HsTrx2:LYS104:HZ3 - P62269:PHE116:O	Hydrogen Bond (conventional)	HsTrx2:LYS104:HZ3 (H-donor)	P62269:PHE116:O (H-acceptor)	1,82383
P62269:PRO98 - HsTrx2:VAL85	Hydrophobic (alkyl)	P62269:PRO98 (alkyl)	HsTrx2:VAL85 (alkyl)	4,97126
P62269:ALA114 - HsTrx2:LYS103	Hydrophobic (alkyl)	P62269:ALA114 (alkyl)	HsTrx2:LYS103 (alkyl)	5,02665
HsTrx2:VAL85 - P62269:LEU88	Hydrophobic (alkyl)	HsTrx2:VAL85 (alkyl)	P62269:LEU88 (alkyl)	4,05532

HsTrx2 interacting residues	Electrostatic interaction	Hydrogen bonding	Salt bridge	Hydrophobic interaction	Other
**GLU68**	+	+	+	-	-
**SER72**	-	+	-	-	-
**VAL85**	-	-	-	+	-
**VAL86**	-	+	-	-	-
**ASP87**	+	-	-	-	-
**LYS88**	-	+	-	-	-
**LYS103**	-	-	-	+	-
**LYS104**	-	+	-	-	-
**GLY107**	-	+	-	-	-

1.32. O-acetyl-ADP-ribose deacetylase MACROD1 (UniProt KB: Q9BQ69):

Interacting residues (Q9BQ69-HsTrx2)	Category (type)	From (chemistry)	To (chemistry)	Distance (Å)
HsTrx2:LYS93:HZ2 - Q9BQ69:GLU289:OE1	Hydrogen Bond; Electrostatic (salt bridge)	HsTrx2:LYS93:HZ2 (H-donor; positive)	Q9BQ69:GLU289:OE1 (H-acceptor; negative)	2,76327
HsTrx2:LYS93:NZ - Q9BQ69:GLU292:OE1	Electrostatic (attractive charge)	HsTrx2:LYS93:NZ (positive)	Q9BQ69:GLU292:OE1 (negative)	5,30838
HsTrx2:LYS104:NZ - Q9BQ69:TYR321	Electrostatic (Pi-cation)	HsTrx2:LYS104:NZ (cation)	Q9BQ69:TYR321 (Pi-orbitals)	2,96481
HsTrx2:SER72:HG - Q9BQ69:SER249:OG	Hydrogen Bond (conventional)	HsTrx2:SER72:HG (H-donor)	Q9BQ69:SER249:OG (H-acceptor)	2,81614
HsTrx2:GLN97:HE21 - Q9BQ69:GLU292:O	Hydrogen Bond (conventional)	HsTrx2:GLN97:HE21 (H-donor)	Q9BQ69:GLU292:O (H-acceptor)	2,94524
HsTrx2:LYS104:HN - Q9BQ69:ARG143:N	Hydrogen Bond (conventional)	HsTrx2:LYS104:HN (H-donor)	Q9BQ69:ARG143:N (H-acceptor)	2,98345
Q9BQ69:ARG143:N - HsTrx2:ALA100:O	Hydrogen Bond (conventional)	Q9BQ69:ARG143:N (H-donor)	HsTrx2:ALA100:O (H-acceptor)	2,47744
Q9BQ69:GLN293:HE22 - HsTrx2:ILE92:O	Hydrogen Bond (conventional)	Q9BQ69:GLN293:HE22 (H-donor)	HsTrx2:ILE92:O (H-acceptor)	3,08153
Q9BQ69:GLN293:HE22 - HsTrx2:GLN97:OE1	Hydrogen Bond (conventional)	Q9BQ69:GLN293:HE22 (H-donor)	HsTrx2:GLN97:OE1 (H-acceptor)	2,48251
Q9BQ69:SER245:CB - HsTrx2:GLU70:O	Hydrogen Bond (carbon)	Q9BQ69:SER245:CB (H-donor)	HsTrx2:GLU70:O (H-acceptor)	2,71128
Q9BQ69:THR286:CA - HsTrx2:LYS88:O	Hydrogen Bond (carbon)	Q9BQ69:THR286:CA (H-donor)	HsTrx2:LYS88:O (H-acceptor)	3,35769

Q9BQ69:HIS294:CE1 - HsTrx2:VAL90:O	Hydrogen Bond (carbon)	Q9BQ69:HIS294:CE1 (H-donor)	HsTrx2:VAL90:O (H-acceptor)	3,60296
HsTrx2:LYS88 - Q9BQ69:LEU248	Hydrophobic (alkyl)	HsTrx2:LYS88 (alkyl)	Q9BQ69:LEU248 (alkyl)	5,39698
HsTrx2:VAL90 - Q9BQ69:LEU248	Hydrophobic (alkyl)	HsTrx2:VAL90 (alkyl)	Q9BQ69:LEU248 (alkyl)	4,46722
HsTrx2:ALA100 - Q9BQ69:ARG143	Hydrophobic (alkyl)	HsTrx2:ALA100 (alkyl)	Q9BQ69:ARG143 (alkyl)	3,94501
Q9BQ69:ARG143 - HsTrx2:LYS103	Hydrophobic (alkyl)	Q9BQ69:ARG143 (alkyl)	HsTrx2:LYS103 (alkyl)	4,77267
Q9BQ69:ALA237 - HsTrx2:VAL16	Hydrophobic (alkyl)	Q9BQ69:ALA237 (alkyl)	HsTrx2:VAL16 (alkyl)	4,47121
Q9BQ69:ALA237 - HsTrx2:MET80	Hydrophobic (alkyl)	Q9BQ69:ALA237 (alkyl)	HsTrx2:MET80 (alkyl)	4,48975
Q9BQ69:ALA240 - HsTrx2:VAL85	Hydrophobic (alkyl)	Q9BQ69:ALA240 (alkyl)	HsTrx2:VAL85 (alkyl)	3,36857
Q9BQ69:ALA241 - HsTrx2:LEU78	Hydrophobic (alkyl)	Q9BQ69:ALA241 (alkyl)	HsTrx2:LEU78 (alkyl)	5,05709
Q9BQ69:ALA241 - HsTrx2:VAL85	Hydrophobic (alkyl)	Q9BQ69:ALA241 (alkyl)	HsTrx2:VAL85 (alkyl)	4,2692
Q9BQ69:ARG244 - HsTrx2:LYS88	Hydrophobic (alkyl)	Q9BQ69:ARG244 (alkyl)	HsTrx2:LYS88 (alkyl)	3,74846
HsTrx2:TYR69 - Q9BQ69:ALA237	Hydrophobic (Pi-alkyl)	HsTrx2:TYR69 (Pi-orbitals)	Q9BQ69:ALA237 (alkyl)	5,1916
HsTrx2:TYR69 - Q9BQ69:ALA241	Hydrophobic (Pi-alkyl)	HsTrx2:TYR69 (Pi-orbitals)	Q9BQ69:ALA241 (alkyl)	4,30429
Q9BQ69:TYR144 - HsTrx2:LYS104	Hydrophobic (Pi-alkyl)	Q9BQ69:TYR144 (Pi-orbitals)	HsTrx2:LYS104 (alkyl)	4,24306

HsTrx2 interacting residues	Electrostatic interaction	Hydrogen bonding	Salt bridge	Hydrophobic interaction	Other
**VAL16**	-	-	-	+	-
**TYR69**	-	-	-	+	-
**GLU70**	-	+	-	-	-
**SER72**	-	+	-	-	-
**LEU78**	-	-	-	+	-
**MET80**	-	-	-	+	-
**VAL85**	-	-	-	+	-
**LYS88**	-	+	-	+	-
**VAL90**	-	+	-	+	-
**ILE92**	-	+	-	-	-
**LYS93**	+	+	+	-	-
**GLN97**	-	+	-	-	-
**ALA100**	-	+	-	+	-
**LYS103**	-	-	-	+	-
**LYS104**	+	+	-	+	-

1.33. Acetyl-coenzyme A synthetase 2-like, mitochondrial (UniProt KB: Q9NUB1):

Interacting residues (Q9NUB1-HsTrx2)	Category (type)	From (chemistry)	To (chemistry)	Distance (Å)
HsTrx2:ARG14:HH22 - Q9NUB1:GLU145:OE1	Hydrogen Bond; Electrostatic (salt bridge)	HsTrx2:ARG14:HH22 (H-donor; positive)	Q9NUB1:GLU145:OE1 (H-acceptor; negative)	2,49949
HsTrx2:ARG14:NH2 - Q9NUB1:GLU145:OE2	Electrostatic (attractive charge)	HsTrx2:ARG14:NH2 (positive)	Q9NUB1:GLU145:OE2 (negative)	4,49411
HsTrx2:LYS56:NZ - Q9NUB1:GLU124:OE2	Electrostatic (attractive charge)	HsTrx2:LYS56:NZ (positive)	Q9NUB1:GLU124:OE2 (negative)	5,39769
Q9NUB1:ARG140:NH1 - HsTrx2:ASP7:OD2	Electrostatic (attractive charge)	Q9NUB1:ARG140:NH1 (positive)	HsTrx2:ASP7:OD2 (negative)	4,31526
Q9NUB1:ARG140:NH1 - HsTrx2:ASP10:OD1	Electrostatic (attractive charge)	Q9NUB1:ARG140:NH1 (positive)	HsTrx2:ASP10:OD1 (negative)	2,83926
Q9NUB1:ARG140:NH2 - HsTrx2:ASP7:OD1	Electrostatic (attractive charge)	Q9NUB1:ARG140:NH2 (positive)	HsTrx2:ASP7:OD1 (negative)	3,30025
Q9NUB1:ARG140:NH2 - HsTrx2:ASP10:OD2	Electrostatic (attractive charge)	Q9NUB1:ARG140:NH2 (positive)	HsTrx2:ASP10:OD2 (negative)	5,26238
Q9NUB1:ARG140:NH2 - HsTrx2:ASP13:OD2	Electrostatic (attractive charge)	Q9NUB1:ARG140:NH2 (positive)	HsTrx2:ASP13:OD2 (negative)	4,59974
HsTrx2:ARG14:HH11 - Q9NUB1:ARG140:O	Hydrogen Bond (conventional)	HsTrx2:ARG14:HH11 (H-donor)	Q9NUB1:ARG140:O (H-acceptor)	2,17032
Q9NUB1:ARG140:HE - HsTrx2:ASP13:OD2	Hydrogen Bond (conventional)	Q9NUB1:ARG140:HE (H-donor)	HsTrx2:ASP13:OD2 (H-acceptor)	2,85059
HsTrx2:ARG14:CD - Q9NUB1:ARG140:O	Hydrogen Bond (carbon)	HsTrx2:ARG14:CD (H-donor)	Q9NUB1:ARG140:O (H-acceptor)	2,54939
HsTrx2:ARG14 - Q9NUB1:ARG140	Hydrophobic (alkyl)	HsTrx2:ARG14 (alkyl)	Q9NUB1:ARG140 (alkyl)	4,75561

HsTrx2 interacting residues	Electrostatic interaction	Hydrogen bonding	Salt bridge	Hydrophobic interaction	Other
**ASP7**	+	-	-	-	-
**ASP10**	+	-	-	-	-
**ASP13**	+	+	-	-	-
**ARG14**	+	+	+	+	-
**LYS56**	+	-	-	-	-

1.34. Probable D-lactate dehydrogenase, mitochondrial (UniProt KB: Q86WU2):

Interacting residues (Q86WU2-HsTrx2)	Category (type)	From (chemistry)	To (chemistry)	Distance (Å)
HsTrx2:LYS88:NZ - Q86WU2:GLU76:OE2	Electrostatic (attractive charge)	HsTrx2:LYS88:NZ (positive)	Q86WU2:GLU76:OE2 (negative)	4,21452
HsTrx2:LYS88:NZ - Q86WU2:GLU191:OE2	Electrostatic (attractive charge)	HsTrx2:LYS88:NZ (positive)	Q86WU2:GLU191:OE2 (negative)	4,70114
Q86WU2:ARG198:NH2 - HsTrx2:ASP94:OD1	Electrostatic (attractive charge)	Q86WU2:ARG198:NH2 (positive)	HsTrx2:ASP94:OD1 (negative)	5,08932
Q86WU2:ARG205:NH1 - HsTrx2:ASP87:OD1	Electrostatic (attractive charge)	Q86WU2:ARG205:NH1 (positive)	HsTrx2:ASP87:OD1 (negative)	5,26379
HsTrx2:ILE92:HN -	Hydrogen Bond	HsTrx2:ILE92:HN	Q86WU2:GLY197:O	2,47314

Q86WU2:GLY197:O	(conventional)	(H-donor)	(H-acceptor)	
Q86WU2:ARG198:HH11 - HsTrx2:ILE92:O	Hydrogen Bond (conventional)	Q86WU2:ARG198:HH11 (H-donor)	HsTrx2:ILE92:O (H-acceptor)	2,81543
Q86WU2:ARG198:CA - HsTrx2:ILE92:O	Hydrogen Bond (carbon)	Q86WU2:ARG198:CA (H-donor)	HsTrx2:ILE92:O (H-acceptor)	3,09204
Q86WU2:ARG198:CD - HsTrx2:ILE92:O	Hydrogen Bond (carbon)	Q86WU2:ARG198:CD (H-donor)	HsTrx2:ILE92:O (H-acceptor)	2,52107
Q86WU2:ARG205:CD - HsTrx2:ASP87:OD1	Hydrogen Bond (carbon)	Q86WU2:ARG205:CD (H-donor)	HsTrx2:ASP87:OD1 (H-acceptor)	3,53181
HsTrx2:VAL90 - Q86WU2:VAL75	Hydrophobic (alkyl)	HsTrx2:VAL90 (alkyl)	Q86WU2:VAL75 (alkyl)	2,79104
HsTrx2:VAL90 - Q86WU2:LEU199	Hydrophobic (alkyl)	HsTrx2:VAL90 (alkyl)	Q86WU2:LEU199 (alkyl)	4,3067
Q86WU2:ALA83 - HsTrx2:ILE92	Hydrophobic (alkyl)	Q86WU2:ALA83 (alkyl)	HsTrx2:ILE92 (alkyl)	3,67399

HsTrx2 interacting residues	Electrostatic interaction	Hydrogen bonding	Salt bridge	Hydrophobic interaction	Other
**ASP87**	+	+	-	-	-
**LYS88**	+	-	-	-	-
**VAL90**	-	-	-	+	-
**ILE92**	-	+	-	+	-
**ASP94**	+	-	-	-	-

1.35. Peroxiredoxin-4 (UniProt KB: Q13162):

Interacting residues (Q13162-HsTrx2)	Category (type)	From (chemistry)	To (chemistry)	Distance (Å)
Q13162:LYS80:HZ3 - HsTrx2:VAL90:O	Hydrogen Bond (conventional)	Q13162:LYS80:HZ3 (H-donor)	HsTrx2:VAL90:O (H-acceptor)	2,28326
Q13162:LEU199:HN - HsTrx2:GLU70:OE2	Hydrogen Bond (conventional)	Q13162:LEU199:HN (H-donor)	HsTrx2:GLU70:OE2 (H-acceptor)	2,65266
Q13162:GLN213:HE22 - HsTrx2:VAL86:O	Hydrogen Bond (conventional)	Q13162:GLN213:HE22 (H-donor)	HsTrx2:VAL86:O (H-acceptor)	2,2067
HsTrx2:ALA73 - Q13162:LEU76	Hydrophobic (alkyl)	HsTrx2:ALA73 (alkyl)	Q13162:LEU76 (alkyl)	4,75215
HsTrx2:LYS88 - Q13162:ILE214	Hydrophobic (alkyl)	HsTrx2:LYS88 (alkyl)	Q13162:ILE214 (alkyl)	3,48451
HsTrx2:VAL90 - Q13162:LYS80	Hydrophobic (alkyl)	HsTrx2:VAL90 (alkyl)	Q13162:LYS80 (alkyl)	4,57544

HsTrx2 interacting residues	Electrostatic interaction	Hydrogen bonding	Salt bridge	Hydrophobic interaction	Other
**GLU70**	-	+	-	-	-
**ALA73**	-	-	-	+	-
**VAL86**	-	+	-	-	-
**LYS88**	-	-	-	+	-
**VAL90**	-	+	-	+	-

1.36. Mitochondrial ribosomal protein L23 (UniProt KB: Q16540):

Interacting residues (Q16540-HsTrx2)	Category (type)	From (chemistry)	To (chemistry)	Distance (Å)
Q16540:LYS279:HZ2 - HsTrx2:ASP61:OD2	Hydrogen Bond; Electrostatic (salt bridge)	Q16540:LYS279:HZ2 (H-donor; positive)	HsTrx2:ASP61:OD2 (H-acceptor; negative)	1,69769
Q16540:LYS279:NZ - HsTrx2:ASP58:OD2	Electrostatic (attractive charge)	Q16540:LYS279:NZ (positive)	HsTrx2:ASP58:OD2 (negative)	5,4458
HsTrx2:THR1:N - Q16540:ASP322:OD1	Electrostatic (attractive charge)	HsTrx2:THR1:N (positive)	Q16540:ASP322:OD1 (negative)	4,68728
HsTrx2:THR1:N - Q16540:ASP324:OD1	Electrostatic (attractive charge)	HsTrx2:THR1:N (positive)	Q16540:ASP324:OD1 (negative)	4,69971
Q16540:ALA74:HN - HsTrx2:ASP13:OD1	Hydrogen Bond (conventional)	Q16540:ALA74:HN (H-donor)	HsTrx2:ASP13:OD1 (H-acceptor)	2,35037
HsTrx2:GLN6:HE21 - Q16540:HIS280:O	Hydrogen Bond (conventional)	HsTrx2:GLN6:HE21 (H-donor)	Q16540:HIS280:O (H-acceptor)	2,25332
HsTrx2:GLN12:HE22 - Q16540:ASP67:OD1	Hydrogen Bond (conventional)	HsTrx2:GLN12:HE22 (H-donor)	Q16540:ASP67:OD1 (H-acceptor)	2,75534
HsTrx2:ARG14:HH22 - Q16540:TYR194:OH	Hydrogen Bond (conventional)	HsTrx2:ARG14:HH22 (H-donor)	Q16540:TYR194:OH (H-acceptor)	2,83565
Q16540:ALA71:CA - HsTrx2:ASN17:O	Hydrogen Bond (carbon)	Q16540:ALA71:CA (H-donor)	HsTrx2:ASN17:O (H-acceptor)	3,39706
HsTrx2:ARG14 - Q16540:LEU77	Hydrophobic (alkyl)	HsTrx2:ARG14 (alkyl)	Q16540:LEU77 (alkyl)	4,36035

HsTrx2 interacting residues	Electrostatic interaction	Hydrogen bonding	Salt bridge	Hydrophobic interaction	Other
**THR1**	+	-	-	-	-
**GLN6**	-	+	-	-	-
**GLN12**	-	+	-	-	-
**ASP13**	-	+	-	-	-
**ARG14**	-	+	-	+	-
**ASN17**	-	+	-	-	-
**ASP58**	+	-	-	-	-
**ASP61**	+	+	+	-	-

1.37. Protein/nucleic acid deglycase DJ-1 (UniProt KB: Q99497):

Interacting residues (Q99497-HsTrx2)	Category (type)	From (chemistry)	To (chemistry)	Distance (Å)
Q99497:ARG27:HH21 - HsTrx2:GLU68:OE1	Hydrogen Bond; Electrostatic (salt bridge)	Q99497:ARG27:HH21 (H-donor; positive)	HsTrx2:GLU68:OE1 (H-acceptor; negative)	2,17033
Q99497:ARG27:NH2 - HsTrx2:ASP64:OD2	Electrostatic (attractive charge)	Q99497:ARG27:NH2 (positive)	HsTrx2:ASP64:OD2 (negative)	3,53192
Q99497:ARG27:HE - HsTrx2:GLU68:OE1	Hydrogen Bond (conventional)	Q99497:ARG27:HE (H-donor)	HsTrx2:GLU68:OE1 (H-acceptor)	2,88328
Q99497:VAL51:HN - HsTrx2:GLU19:OE1	Hydrogen Bond (conventional)	Q99497:VAL51:HN (H-donor)	HsTrx2:GLU19:OE1 (H-acceptor)	2,39352

Q99497:CYS53:HN - HsTrx2:SER18:O	Hydrogen Bond (conventional)	Q99497:CYS53:HN (H-donor)	HsTrx2:SER18:O (H-acceptor)	2,07613
HsTrx2:ASN17:CA - Q99497:CYS53:O	Hydrogen Bond (carbon)	HsTrx2:ASN17:CA (H-donor)	Q99497:CYS53:O (H-acceptor)	3,5677
Q99497:ARG27 - HsTrx2:PRO9	Hydrophobic (alkyl)	Q99497:ARG27 (alkyl)	HsTrx2:PRO9 (alkyl)	4,20213
Q99497:ARG28 - HsTrx2:PRO9	Hydrophobic (alkyl)	Q99497:ARG28 (alkyl)	HsTrx2:PRO9 (alkyl)	3,39663

HsTrx2 interacting residues	Electrostatic interaction	Hydrogen bonding	Salt bridge	Hydrophobic interaction	Other
**PRO9**	-	-	-	+	-
**ASN17**	-	+	-	-	-
**SER18**	-	+	-	-	-
**GLU19**	-	+	-	-	-
**ASP64**	+	-	-	-	-
**GLU68**	+	+	+	-	-

1.38. Aconitate hydratase, mitochondrial (UniProt KB: Q99798):

Interacting residues (Q99798-HsTrx2)	Category (type)	From (chemistry)	To (chemistry)	Distance (Å)
Q99798:LYS370:HZ1 - HsTrx2:ASP96:OD1	Hydrogen Bond; Electrostatic (salt bridge)	Q99798:LYS370:HZ1 (H-donor; positive)	HsTrx2:ASP96:OD1 (H-acceptor; negative)	2,40459
Q99798:LYS250:NZ - HsTrx2:GLU42:OE1	Electrostatic (attractive charge)	Q99798:LYS250:NZ (positive)	HsTrx2:GLU42:OE1 (negative)	4,71865
Q99798:LYS370:NZ - HsTrx2:GLU95:OE1	Electrostatic (attractive charge)	Q99798:LYS370:NZ (positive)	HsTrx2:GLU95:OE1 (negative)	4,95507
HsTrx2:LYS35:NZ - Q99798:GLU510:OE1	Electrostatic (attractive charge)	HsTrx2:LYS35:NZ (positive)	Q99798:GLU510:OE1 (negative)	4,81692
HsTrx2:ARG40:NH1 - Q99798:GLU363:OE2	Electrostatic (attractive charge)	HsTrx2:ARG40:NH1 (positive)	Q99798:GLU363:OE2 (negative)	4,37003
HsTrx2:ARG40:NH2 - Q99798:GLU363:OE1	Electrostatic (attractive charge)	HsTrx2:ARG40:NH2 (positive)	Q99798:GLU363:OE1 (negative)	2,42195
HsTrx2:LYS43:NZ - Q99798:GLU363:OE2	Electrostatic (attractive charge)	HsTrx2:LYS43:NZ (positive)	Q99798:GLU363:OE2 (negative)	4,22418
HsTrx2:LYS56:NZ - Q99798:GLU344:OE1	Electrostatic (attractive charge)	HsTrx2:LYS56:NZ (positive)	Q99798:GLU344:OE1 (negative)	5,31013
Q99798:GLU339:HN - HsTrx2:THR2:O	Hydrogen Bond (conventional)	Q99798:GLU339:HN (H-donor)	HsTrx2:THR2:O (H-acceptor)	2,23003
HsTrx2:ARG40:HE - Q99798:GLU363:OE2	Hydrogen Bond (conventional)	HsTrx2:ARG40:HE (H-donor)	Q99798:GLU363:OE2 (H-acceptor)	1,84665
HsTrx2:LYS43:HZ2 - Q99798:ALA358:O	Hydrogen Bond (conventional)	HsTrx2:LYS43:HZ2 (H-donor)	Q99798:ALA358:O (H-acceptor)	2,5617
HsTrx2:LYS43:HZ3 - Q99798:ALA358:O	Hydrogen Bond (conventional)	HsTrx2:LYS43:HZ3 (H-donor)	Q99798:ALA358:O (H-acceptor)	2,67267

Q99798:LYS346 - HsTrx2:LYS35	Hydrophobic (alkyl)	Q99798:LYS346 (alkyl)	HsTrx2:LYS35 (alkyl)	4,4831
Q99798:ALA358 - HsTrx2:LYS43	Hydrophobic (alkyl)	Q99798:ALA358 (alkyl)	HsTrx2:LYS43 (alkyl)	4,54009
Q99798:PRO360 - HsTrx2:PRO39	Hydrophobic (alkyl)	Q99798:PRO360 (alkyl)	HsTrx2:PRO39 (alkyl)	4,22595
Q99798:ALA362 - HsTrx2:ILE36	Hydrophobic (alkyl)	Q99798:ALA362 (alkyl)	HsTrx2:ILE36 (alkyl)	4,1907
HsTrx2:ALA46 - Q99798:ILE254	Hydrophobic (alkyl)	HsTrx2:ALA46 (alkyl)	Q99798:ILE254 (alkyl)	3,61939
Q99798:HIS348 - HsTrx2:PRO39	Hydrophobic (Pi-alkyl)	Q99798:HIS348 (Pi-orbitals)	HsTrx2:PRO39 (alkyl)	5,26698

HsTrx2 interacting residues	Electrostatic interaction	Hydrogen bonding	Salt bridge	Hydrophobic interaction	Other
**THR2**	-	+	-	-	-
**LYS35**	+	-	-	+	-
**ILE36**	-	-	-	+	-
**PRO39**	-	-	-	+	-
**ARG40**	+	+	-	-	-
**GLU42**	+	-	-	-	-
**LYS43**	+	+	-	+	-
**ALA46**	-	-	-	+	-
**LYS56**	+	-	-	-	-
**GLU95**	+	-	-	-	-
**ASP96**	+	+	+	-	-

Table 2. Solvent accessibility for all residues of HsTrx2. The percentages were generated by BIOVIA.

Percent solvent accessibility	Residue
55.616	THR1
37.595	THR2
9.749	PHE3
58.739	ASN4
3.982	ILE5
73.949	GLN6
49.18	ASP7
56.655	GLY8
76.83	PRO9
20.393	ASP10
2.642	PHE11
53.585	GLN12
69.048	ASP13
41.816	ARG14
2.094	VAL15
24.461	VAL16
67.877	ASN17
17.037	SER18
106.453	GLU19
29.728	THR20
12.262	PRO21
0.358	VAL22
0	VAL23
0.268	VAL24
4.303	ASP25
0.314	PHE26
21.863	HIS27
5.092	ALA28
82.096	GLN29
69.411	TRP30
9.622	CYS31
64.955	GLY32
46.931	PRO33
12.062	SER34
70.409	LYS35
57.563	ILE36
0.658	LEU37
12.798	GLY38
60.058	PRO39
30.707	ARG40
0.58	LEU41
30.008	GLU42
68.819	LYS43

17.66	MET44
0	VAL45
40.874	ALA46
67.371	LYS47
37.196	GLN48
93.963	HIS49
45.385	GLY50
66.075	LYS51
0	VAL52
15.297	VAL53
0.069	MET54
0	ALA55
16.733	LYS56
0	VAL57
11.649	ASP58
17.544	ILE59
52.261	ASP60
77.619	ASP61
13.101	HIS62
53.928	THR63
66.634	ASP64
6.966	LEU65
11.093	ALA66
78.612	ILE67
68.367	GLU68
19.56	TYR69
84.69	GLU70
18.017	VAL71
95.627	SER72
42.428	ALA73
19.692	VAL74
1.879	PRO75
0.806	THR76
0.179	VAL77
2.073	LEU78
1.005	ALA79
1.154	MET80
44.497	LYS81
80.353	ASN82
29.133	GLY83
66.23	ASP84
55.115	VAL85
62.388	VAL86
43.161	ASP87
50.53	LYS88
8.617	PHE89

55.826	VAL90
23.182	GLY91
52.779	ILE92
30.915	LYS93
53.617	ASP94
65.92	GLU95
80.944	ASP96
48.079	GLN97
0.083	LEU98
45.908	GLU99
43.16	ALA100
8.738	PHE101
5.971	LEU102
63.923	LYS103
83.451	LYS104
15.482	LEU105
36.843	ILE106
149.919	GLY107
