# Supplementary material for: Distinct or Overlapping Areas of Mitochondrial Thioredoxin 2 May Be Used for Its Covalent and Strong Non-Covalent Interactions with Protein Ligands
Source: Antioxidants (Basel). 2023 Dec 20;13(1):15. doi: 10.3390/antiox13010015 (PMC10812433; doi:10.3390/antiox13010015)
Supplement: Supplementary file 1 [file antioxidants-13-00015-s001.zip › Supplementary data S3 (HsTrx2 hot spots).pdf]

Supplementary Data S3: *Contact hot spots of HsTrx2*

Distinct or shared areas of mitochondrial thioredoxin 2 may be used for its covalent and strong non-covalent interactions with protein ligands

Charalampos Ntallis [1], Haralambos Tzoupis [1], Theodore Tselios [1], Christos T. Chasapis [2] and Alexios Vlamis-Gardikas [1,*]

[1] Department of Chemistry, University of Patras, Rion 26504, Greece; xntallis@gmail.com, c.ntallis@uu.nl (C.N.); haralambostz@gmail.com (H.T.); ttselios@upatras.gr (T.T.)
[2] Institute of Chemical Biology, National Hellenic Research Foundation, Vas. Constantinou 48 av, Athens, 11635, Greece; cchasapis@eie.gr. (C.T.C).
*Correspondence: avlamis@upatras.gr; Tel.: +30-2610-997634

Table 1: Number of interactions for each residue of thioredoxin-2 with its substrates (only covalent interactions included).

HsTrx2 residues (#)			Number of interactions (##)		
(#)	(##)	(#)	(##)	(#)	(##)
THR1	0	MET44	0	ASP87	2
THR2	1	VAL45	0	LYS88	4
PHE3	0	ALA46	2	PHE89	0
ASN4	0	LYS47	2	VAL90	3
ILE5	0	GLN48	0	GLY91	0
GLN6	0	HIS49	2	ILE92	3
ASP7	1	GLY50	0	LYS93	3
GLY8	0	LYS51	1	ASP94	2
PRO9	1	VAL52	0	GLU95	1
ASP10	1	VAL53	0	ASP96	4
PHE11	0	MET54	0	GLN97	2
GLN12	0	ALA55	0	LEU98	0
ASP13	1	LYS56	2	GLU99	2
ARG14	1	VAL57	0	ALA100	1
VAL15	0	ASP58	0	PHE101	0
VAL16	1	ILE59	0	LEU102	0
ASN17	1	ASP60	1	LYS103	4
SER18	1	ASP61	0	LYS104	3
GLU19	1	HIS62	0	LEU105	0
THR20	0	THR63	1	ILE106	1
PRO21	0	ASP64	1	GLY107	1
VAL22	0	LEU65	0		
VAL23	0	ALA66	0		
VAL24	0	ILE67	1		
ASP25	0	GLU68	1		
PHE26	0	TYR69	2		
HIS27	0	GLU70	3		
ALA28	0	VAL71	1		
GLN29	1	SER72	5		
TRP30	4	ALA73	2		
CYS31	2	VAL74	1		
GLY32	1	PRO75	1		
PRO33	3	THR76	0		
SER34	0	VAL77	0		
LYS35	4	LEU78	1		
ILE36	3	ALA79	0		
LEU37	0	MET80	2		
GLY38	0	LYS81	0		
PRO39	2	ASN82	0		
ARG40	2	GLY83	0		
LEU41	0	ASP84	1		
GLU42	2	VAL85	2		
LYS43	1	VAL86	2		

Table: 2 Number of interactions for each residue of thioredoxin-2 with its substrates (only strong non-covalent interactions included).

**HsTrx2 residues (#)**			**Number of interactions (##)**		
**(#)**	**(##)**	**(#)**	**(##)**	**(#)**	**(##)**
THR1	4	MET44	0	ASP87	1
THR2	2	VAL45	0	LYS88	3
PHE3	3	ALA46	2	PHE89	0
ASN4	1	LYS47	0	VAL90	2
ILE5	0	GLN48	0	GLY91	1
GLN6	1	HIS49	0	ILE92	3
ASP7	2	GLY50	1	LYS93	3
GLY8	1	LYS51	0	ASP94	4
PRO9	2	VAL52	0	GLU95	2
ASP10	3	VAL53	2	ASP96	3
PHE11	0	MET54	0	GLN97	1
GLN12	0	ALA55	0	LEU98	0
ASP13	1	LYS56	0	GLU99	0
ARG14	3	VAL57	0	ALA100	0
VAL15	0	ASP58	1	PHE101	0
VAL16	1	ILE59	0	LEU102	1
ASN17	1	ASP60	1	LYS103	2
SER18	0	ASP61	1	LYS104	3
GLU19	2	HIS62	1	LEU105	0
THR20	1	THR63	1	ILE106	0
PRO21	0	ASP64	0	GLY107	1
VAL22	0	LEU65	0		
VAL23	0	ALA66	0		
VAL24	0	ILE67	1		
ASP25	0	GLU68	1		
PHE26	0	TYR69	0		
HIS27	0	GLU70	1		
ALA28	0	VAL71	0		
GLN29	0	SER72	2		
TRP30	0	ALA73	3		
CYS31	1	VAL74	0		
GLY32	1	PRO75	0		
PRO33	2	THR76	0		
SER34	0	VAL77	0		
LYS35	1	LEU78	0		
ILE36	2	ALA79	0		
LEU37	0	MET80	0		
GLY38	0	LYS81	0		
PRO39	1	ASN82	0		
ARG40	2	GLY83	0		
LEU41	0	ASP84	0		
GLU42	0	VAL85	1		
LYS43	0	VAL86	1		

Table 3: Number of interactions for each residue of thioredoxin-2 with its substrates (all categories included, i.e., covalent, strong non-covalent and weak non-covalent interactions).

HsTrx2 residues (#)			Number of interactions (##)		
(#)	(##)	(#)	(##)	(#)	(##)
THR1	6	PRO39	3	VAL77	0
THR2	4	ARG40	5	LEU78	1
PHE3	3	LEU41	0	ALA79	0
ASN4	2	GLU42	2	MET80	2
ILE5	2	LYS43	1	LYS81	1
GLN6	2	MET44	0	ASN82	0
ASP7	3	VAL45	0	GLY83	0
GLY8	1	ALA46	4	ASP84	2
PRO9	3	LYS47	2	VAL85	6
ASP10	4	GLN48	0	VAL86	6
PHE11	0	HIS49	2	ASP87	4
GLN12	1	GLY50	1	LYS88	11
ASP13	3	LYS51	1	PHE89	0
ARG14	5	VAL52	0	VAL90	10
VAL15	0	VAL53	2	GLY91	1
VAL16	2	MET54	0	ILE92	7
ASN17	3	ALA55	0	LYS93	8
SER18	1	LYS56	3	ASP94	7
GLU19	3	VAL57	0	GLU95	3
THR20	1	ASP58	2	ASP96	7
PRO21	0	ILE59	2	GLN97	4
VAL22	0	ASP60	4	LEU98	0
VAL23	0	ASP61	3	GLU99	2
VAL24	0	HIS62	1	ALA100	4
ASP25	0	THR63	3	PHE101	0
PHE26	0	ASP64	2	LEU102	1
HIS27	0	LEU65	0	LYS103	8
ALA28	0	ALA66	2	LYS104	9
GLN29	2	ILE67	5	LEU105	1
TRP30	9	GLU68	3	ILE106	1
CYS31	5	TYR69	2	GLY107	2
GLY32	2	GLU70	8		
PRO33	8	VAL71	3		
SER34	0	SER72	14		
LYS35	6	ALA73	8		
ILE36	5	VAL74	3		
LEU37	0	PRO75	2		
GLY38	0	THR76	0		
