# Supplementary material for: Distinct or Overlapping Areas of Mitochondrial Thioredoxin 2 May Be Used for Its Covalent and Strong Non-Covalent Interactions with Protein Ligands
Source: Antioxidants (Basel). 2023 Dec 20;13(1):15. doi: 10.3390/antiox13010015 (PMC10812433; doi:10.3390/antiox13010015)
Supplement: Supplementary file 1 [file antioxidants-13-00015-s001.zip › Supplementary data S4 (SpotOn and PROT-ON).pdf]

Supplementary Data S4: *Prediction of possible thermodynamic hot spots by SpotOn and PROT-ON*

Distinct or shared areas of mitochondrial thioredoxin 2 may be used for its covalent and strong non-covalent interactions with protein ligands

Charalampos Ntallis [1], Haralambos Tzoupis [1], Theodore Tselios [1], Christos T. Chasapis [2] and Alexios Vlamis-Gardikas [1,*]

[1] Department of Chemistry, University of Patras, Rion 26504, Greece; xntallis@gmail.com, c.ntallis@uu.nl (C.N.); haralambostz@gmail.com (H.T.); ttselios@upatras.gr (T.T.)
[2] Institute of Chemical Biology, National Hellenic Research Foundation, Vas. Constantinou 48 av, Athens, 11635, Greece; cchasapis@eie.gr. (C.T.C).

*Correspondence: avlamis@upatras.gr; Tel.: +30-2610-997634

1. Contacts defined by BIOVIA

All contacts (77):
1-10, 12-14, 16-20, 29-33, 35, 36, 39, 40, 42, 43, 46, 47, 49-51, 53, 56, 58-64, 66-75, 78, 80, 81, 84-88, 90-97, 99, 100, 102-107.

Covalent contacts (58).
2, 7, 9, 10, 13, 14, 16-19, 29-33, 35, 36, 39, 40, 42, 42, 46, 47, 49, 51, 56, 60, 63, 64, 67-75, 78, 80, 84-88, 90, 92-97, 99, 100, 103, 104, 106, 107.

Strong non-covalent (sNC) contacts (51):
1-4, 6-10, 13, 14, 16, 17, 19, 20, 31-33, 35, 36, 39, 40, 46, 50, 53, 58, 60-63, 67, 68, 70, 72, 73, 85-88, 90-97, 102-104, 107.

Y2H (and other contacts not included as covalent or sNC):
1, 2, 4-6, 12-14, 17, 29-31, 33, 35, 40, 56, 58-61, 63, 64, 66-68, 70-75, 81, 84-88, 90, 92-94, 97, 100, 103-105.

2. Putative thermodynamic hot spot and null spot residues by Spot On concerning the complexes of Table 1.

Table 1: SPOTON results from monothiol HsTrx2 (PDB ID: 1w89) complexes.

**UniProt ID**	**Predicted Hot-spot residues**	**Predicted Null spot residues**
A8MXV4	70, 71, 80, 84	11, 12, 16, 17, 24, 26, 48, 65, 68, 69, 72, 76, 78, 79, 82, 83, 85, 86, 87, 88
O00483		1, 2, 3, 4, 5, 6, 10, 14, 18, 49, 50, 51, 53, 56
O75306	53, 102	1, 2, 3, 4, 5, 6, 10, 11, 13, 14, 17-21, 35, 36, 39, 40, 42, 43, 44, 46, 47, 48, 49, 50, 51, 52, 55, 92, 94, 95, 96, 97, 99, 100, 103, 104, 106, 107
O75489		1-11, 13, 14, 29, 42, 45, 46, 47, 49, 50, 60, 62, 63,
O75828	71, 87	30, 59, 63, 66, 67, 68, 70, 72, 73, 74, 84, 85, 86, 88, 89, 90, 101, 104
O75891	102, 103, 106	19, 44, 45, 47-52, 86, 87, 88, 93, 96, 97, 99, 100, 101, 104, 107
P00395	81, 88	34, 70, 72, 76, 82-87, 89, 90, 93, 96, 97, 100, 101, 103, 104, 105, 107

P00441		2, 28, 29, 30, 31, 32, 35, 58, 60, 63
P04406	84	33, 67-76, 78, 83, 85-91, 93, 100, 101, 104
P06576	73, 74	30-34, 36, 40, 70, 71, 72, 75, 76, 88, 90, 91, 92, 93, 97
P07195	50	1-7, 9-14, 16-20, 49, 51, 53, 56, 82
P07237	30, 59, 60, 71	6, 29, 31-35, 63, 66, 67, 70, 72, 73, 74
P08559	11, 64, 65	5-9, 12, 16, 29, 57-63, 66-69
P11177	71	31, 59, 60, 63, 64, 66, 67, 68, 70, 72, 73, 74, 76, 88
P17540	32, 36, 42	2, 29, 30, 33, 35, 38, 39, 46, 46, 56, 96
P22695		30-37, 39, 40, 70, 72, 73, 74, 88, 89, 90, 92-97, 100, 104
P25705		29-32, 35, 40, 59, 60, 63, 64, 66-74, 76, 88, 90, 92, 93, 94, 97
P30041	70, 107	43, 47, 48, 49, 51, 72, 73, 75, 85-94, 96, 97, 99, 99, 100, 102, 103, 104, 106
P30044	60, 63, 71	23, 30, 59, 61, 62, 64, 66, 67, 68, 69, 70, 72, 73, 74, 88, 93
P30048	30, 59, 60, 71, 73, 74	29, 31, 32, 33, 41, 63, 67, 70, 72, 75, 88, 90, 91
P31930	36	29-33, 35, 37, 40, 74, 92, 93, 94, 96
P32119	71, 85	26, 30, 59, 66, 67, 68, 69, 70, 72-76, 78, 83, 84, 86-94, 97, 99, 100-104
P38646	48	44, 47, 49, 51, 95-103, 107
P40939		30, 31, 32, 72-75, 85-97, 99, 100, 101, 103, 104
P48735	94	30, 31, 32, 33, 36, 37, 40, 47, 75, 89-93, 95, 96, 97, 100, 104
P49247	33	1, 2, 4, 28, 29, 30, 31, 32, 35, 36, 40, 42, 56, 58, 61, 69, 70, 72, 74, 75, 85-88, 90-94, 97, 104
P49411	74	30-34, 60, 72, 73, 75, 90, 91, 92, 94
P49821	73, 89, 91	30-36, 70, 71, 72, 74, 75, 88, 90, 92, 93, 94, 97, 100
P50213	30	28, 29, 30, 31, 32, 33, 35, 58, 59, 60, 61, 72, 74
P60174	90	46, 72, 73, 74, 75, 79, 81, 86, 87, 88, 89, 91, 92, 93, 94, 96, 97, 98, 100, 101, 103-107
P62269	87, 107	67-72, 78, 83, 84, 85, 86, 88, 93, 100, 103, 104, 106

Q9BQ69	69, 85, 88, 89	16, 68, 70, 71, 72, 76, 78, 80, 83, 84, 86, 87, 90-94, 97, 99, 100, 101, 103, 104
Q9NUB1	10, 13	1, 2, 3, 4, 6, 7, 8, 9, 11, 12, 14, 17, 56
Q86WU2	90, 91, 98	37, 71, 72, 73, 74, 76, 79, 81, 82, 85-89, 92, 93, 94, 101
Q13162	69, 70, 88, 89	12, 16, 67, 68, 71, 72, 73, 78, 79, 83-87, 90, 91, 92, 97, 104
Q16540	81	1-7, 9, 12, 13, 14, 17, 18, 19, 20, 42, 43, 45-51, 53, 58, 61, 68
Q99497		7, 8, 9, 10, 12, 13, 16, 17, 18, 19, 20, 23, 64, 65, 68, 82, 83, 84
Q99798		1, 2, 3, 4, 6, 14, 27, 28, 29, 35, 36, 37, 39, 40, 42, 43, 46, 47, 49, 56, 58, 61, 95, 96

All SpotON putative thermodynamic hot spots (36):
10, 11, 13, 30, 32, 33, 36, 42, 48, 50, 53, 59, 60, 63, 64, 65, 69, 70, 71, 73, 74, 80, 81, 84, 85, 87, 88, 89, 90, 91, 94, 98, 102, 103, 106, 107.

Boxed residues are excluded as not being recognized as contacts by BIOVIA. The resulting "approved" 31 residues are:
10, 13, 30, 32, 33, 36, 42, 50, 53, 59, 60, 63, 64, 69, 70, 71, 73, 74, 80, 81, 84, 85, 87, 88, 90, 91, 94, 102, 103, 106, 107.

Comparison of SpotOn putative hot spots and covalent contacts

7 residues included exclusively in "SpotOn":
50, 53, 59, 71, 81, 91, 102.

24 common residues in "SpotOn" and "Covalent contacts":
10, 13, 30, 32, 33, 36, 42, 60, 63, 64, 69, 70, 73, 74, 80, 84, 85, 87, 88, 90, 94, 103, 106, 107.

33 residues included exclusively in "Covalent contacts":
2, 7, 9, 14, 16, 17, 18, 19, 29, 31, 35, 39, 40, 43, 46, 47, 49, 51, 56, 67, 68, 72, 75, 78, 86, 92, 93, 95, 96, 97, 99, 100, 104.

Comparison of SpotOn putative hot spots and strong non-covalent (sNC) contacts

11 residues included exclusively in "SpotOn":
30, 42, 59, 64, 69, 71, 74, 80, 81, 84, 106
20 common residues in "SpotOn" and "sNC contacts":
10, 13, 32, 33, 36, 50, 53, 60, 63, 70, 73, 85, 87, 88, 90, 91, 94, 102, 103, 107.

30 residues included exclusively in "sNC contacts":

1, 2, 3, 4, 6, 7, 8, 9, 14, 16, 17, 19, 20, 31, 35, 39, 40, 46, 58, 61, 62, 68, 72, 86, 92, 93, 95, 96, 97, 104.

Summary*: All SpotON putative hot spots (31):
10, 13, **30**, 32, 33, 36, 42, 50, 53, 59, 60, 63, 64, 69, 70, 71, 73, 74, 80, 81, 84, 85, 87, **88**, 90, 91, 94, 102, **103**, 106, 107.

***Key**
Contact covalent hot spots were: **30**, 35, 72, **88**, 96, **103**. Common with SpotOn: are in bold **30**, **88**, **103.**
sNC contact hot spots were: 1, 3 ,10, 14, 73, 88, 92, 93, 94, 96, 104. Common with SpotOn are underlined: 10, 73, 88, 94**.**
All contacts hot spots: 30, 33, 70, 72, 73, 88, 90 93, 103, 104. The common with SpotOn are in yellow background: 30, 33, 70, 73, 88, 90, 103.
With red letters are all residues that are not included in interface areas as defined by the approach of contact hot spots and their network energy neighbors (Table 3).

3. SpotOn Null Spots

All null spots:
All 107 residues of HsTrx2 but 15, 22, 25, 54, 77.

Comparison of Null spots and All contacts
77 common residues in "Null Spots" and "All contacts":
1, 2, 3, 4, 5, 6, 7, 8, 9, 10, 12, 13, 14, 16, 17, 18, 19, 20, 29, 30, 31, 32, 33, 35, 36, 39, 40, 42, 43, 46, 47, 49, 50, 51, 53, 56, 58, 59, 60, 61, 62, 63, 64, 66, 67, 68, 69, 70, 71, 72, 73, 74, 75, 78, 80, 81, 84, 85, 86, 87, 88, 90, 91, 92, 93, 94, 95, 96, 97, 99, 100, 102, 103, 104, 105, 106, 107.

20 residues included exclusively in "Null Spots":
1, 3, 4, 5, 6, 8, 12, 20, 50, 53, 58, 59, 61, 62, 66, 71, 81, 91, 102, 105.

Comparison of Null Spots and Covalent contacts
57 common residues in "Null Spots" and "Covalent contacts":
2, 7, 9, 10, 13, 14, 16, 17, 18, 19, 29, 30, 31, 32, 33, 35, 36, 39, 40, 42, 43, 46, 47, 49, 51, 56, 60, 63, 64, 67, 68, 69, 70, 72, 73, 74, 75, 78, 80, 84, 85, 86, 87, 88, 90, 92, 93, 94, 95, 96, 97, 99, 100, 103, 104, 106,107.

0 residues included exclusively in "Covalent contacts".

Comparison of Null Spots and sNC contacts
27 residues included exclusively in "Null Spots":
5, 12, 18, 29, 30, 42, 43, 47, 49, 51, 56, 59, 64, 66, 67, 69, 71, 74, 75, 78, 80, 81, 84, 99, 100, 105, 106.

50 common residues in "Null Spots" and "sNC":
1, 2, 3, 4, 6, 7, 8, 9, 10, 13, 14, 16, 17, 19, 20, 31, 32, 33, 35, 36, 39, 40, 46, 50, 53, 58, 60, 61, 62, 63, 68, 70, 72, 73, 85, 86, 87, 88, 90, 91, 92, 93, 94, 95, 96, 97, 102, 103, 104, 107.

0 residues included exclusively in "sNC"

Comparison of the null spot residues that were common with covalent contacts ("C Null Spots) to those that were common with sNC contacts ("sNC Null Spots").

20 residues included exclusively in "C Null Spots":
18, 29, 30, 42, 43, 47, 49, 51, 56, 64, 67, 69, 74, 75, 78, 80, 84, 99, 100, 106.

37 common residues in "C Null Spots" and "sNC Null Spots":
2, 7, 9, 10, 13, 14, 16, 17, 19, 31, 32, 33, 35, 36, 39, 40, 46, 60, 63, 68, 70, 72, 73, 85, 86, 87, 88, 90, 92, 93, 94, 95, 96, 97, 103, 104, 107.

13 residues included exclusively in "sNC Null Spots":
1, 3, 4, 6, 8, 20, 50, 53, 58, 61, 62, 91,102.

4. PROT-ON

Presentation of depleting and enriching mutations in the 38 complexes of HsTrx2 with protein ligands.

The titles/numbers at the beginning of each paragraph correspond to the numbers of the Uniprot IDs of Table 1.

A8MXV4
The most depleting mutation is **Y69R** with **2.99** ΔΔG score.
The most enriching mutation is **G83F** with **-3.85** ΔΔG score.
The residue most frequently leading to depleting binding is **Y69.**
The residue most frequently leading to enriching binding is **D84.**

O00483
The most depleting mutation is **G50Y** with **6.89** ΔΔG score.
The most enriching mutation is **H49M** with **-4.74** ΔΔG score.
The residue most frequently leading to depleting binding is **G50.**
The residue most frequently leading to enriching binding is **H49.**

O75306
The most depleting mutation is **D10L** with **3.31** ΔΔG score.
The most enriching mutation is **E99W** with **-3.53** ΔΔG score.
The residue most frequently leading to depleting binding is **D10.**
The residue most frequently leading to enriching binding is **F3.**
O75489

The most depleting mutation is **D10W** with **3.79** ΔΔG score.
The most enriching mutation is **Q6M** with **-2.3** ΔΔG score.
The residue most frequently leading to depleting binding is **T63.**
The residue most frequently leading to enriching binding is **T1.**

O75828
The most depleting mutation is **S72W** with **4.45** ΔΔG score.
The most enriching mutation is **D84E** with **-2.21** ΔΔG score.
The residue most frequently leading to depleting binding is **I67.**
The residue most frequently leading to enriching binding is **E70.**

O75891
The most depleting mutation is **A100Y** with **3.19** ΔΔG score.
The most enriching mutation is **Q48F** with **-3.13** ΔΔG score.
The residue most frequently leading to depleting binding is **D96.**
The residue most frequently leading to enriching binding is **Q48.**

P00395
The most enriching mutation is **D96M** with **-3.86** ΔΔG score.
The residue most frequently leading to depleting binding is **F101.**
The residue most frequently leading to enriching binding is **D96.**

P00441
The most depleting mutation is **W30D** with **4.1** ΔΔG score.
The most enriching mutation is **Q29I** with **-3.99** ΔΔG score.
The residue most frequently leading to depleting binding is **W30.**
The residue most frequently leading to enriching binding is **Q29.**

P04406
The most depleting mutation is **V90R** with **2.5** ΔΔG score.
The most enriching mutation is **D87W** with **-2.53** ΔΔG score.
The residue most frequently leading to depleting binding is **E70.**
The residue most frequently leading to enriching binding is **D84.**

P06576
The most depleting mutation is **G91R** with **8.17** ΔΔG score.
The most enriching mutation is **S72Y** with **-2.36** ΔΔG score.
The residue most frequently leading to depleting binding is **A73.**
The residue most frequently leading to enriching binding is **W30.**

P07195
The most depleting mutation is **G50T** with **8.17** ΔΔG score.
The most enriching mutation is **D13Y** with **-7.16** ΔΔG score.
The residue most frequently leading to depleting binding is **G50.**
The residue most frequently leading to enriching binding is **D13.**

P07237
The most depleting mutation is **W30G** with **3.24** ΔΔG score.
The most enriching mutation is **D60R** with **-1.77** ΔΔG score.
The residue most frequently leading to depleting binding is **W30.**
The residue most frequently leading to enriching binding is **S72.**

P08559
The most depleting mutation is **G8W** with **25.16** ΔΔG score.
The most enriching mutation is **D64M** with **-5.19** ΔΔG score.
The residue most frequently leading to depleting binding is **G8.**
The residue most frequently leading to enriching binding is **D64.**

P11177
The most depleting mutation is **V71Y** with **5.0** ΔΔG score.
The most enriching mutation is **D64F** with **-2.13** ΔΔG score.
The residue most frequently leading to depleting binding is **V71.**
The residue most frequently leading to enriching binding is **D64.**

P17540
The most depleting mutation is **G32R** with **5.04** ΔΔG score.
The most enriching mutation is **K56T** with **-4.4** ΔΔG score.
The residue most frequently leading to depleting binding is **G32.**
The residue most frequently leading to enriching binding is **K56.**

P22695
The most depleting mutation is **G32R** with **2.74** ΔΔG score.
The most enriching mutation is **S72M** with **-4.23** ΔΔG score.
The residue most frequently leading to depleting binding is **I36.**
The residue most frequently leading to enriching binding is **S72.**

P25705
The most depleting mutation is **G32Y** with **4.37** ΔΔG score.
The most enriching mutation is **E70Q** with **-2.4** ΔΔG score.
The residue most frequently leading to depleting binding is **W30.**
The residue most frequently leading to enriching binding is **E70.**

P30041
The most depleting mutation is **G91D** with **6.49** ΔΔG score.
The most enriching mutation is **D87W** with **-3.58** ΔΔG score.
The residue most frequently leading to depleting binding is **G91.**
The residue most frequently leading to enriching binding is **K104.**

P30044
The most depleting mutation is **W30P** with **1.81** ΔΔG score.
The most enriching mutation is **T63L** with **-2.31** ΔΔG score.
The residue most frequently leading to depleting binding is **W30.**
The residue most frequently leading to enriching binding is **E70.**

P30048
The most depleting mutation is **S72W** with **7.62** ΔΔG score.
The most enriching mutation is **D60L** with **-2.18** ΔΔG score.
The residue most frequently leading to depleting binding is **W30.**
The residue most frequently leading to enriching binding is **D60.**

P31930
The most depleting mutation is **G32R** with **8.41** ΔΔG score.
The most enriching mutation is **K35M** with **-1.66** ΔΔG score.
The residue most frequently leading to depleting binding is **G32.**
The residue most frequently leading to enriching binding is **W30.**

P32119
The most depleting mutation is **S72R** with **3.6** ΔΔG score.
The most enriching mutation is **D96M** with **-3.52** ΔΔG score.
The residue most frequently leading to depleting binding is **S72.**
The residue most frequently leading to enriching binding is **D96.**

P38646
The most depleting mutation is **A100Y** with **2.2** ΔΔG score.
The most enriching mutation is **E99Q** with **-2.19** ΔΔG score.
The residue most frequently leading to depleting binding is **D96.**
The residue most frequently leading to enriching binding is **Q48.**

P40939
The most depleting mutation is **A73R** with **7.26** ΔΔG score.
The most enriching mutation is **S72W** with **-4.89** ΔΔG score.
The residue most frequently leading to depleting binding is **G91.**
The residue most frequently leading to enriching binding is **S72.**

P48735
The most depleting mutation is **G32K** with **7.03** ΔΔG score.
The most enriching mutation is **D94I** with **-3.57** ΔΔG score.
The residue most frequently leading to depleting binding is **E95.**
The residue most frequently leading to enriching binding is **D94.**

P49247
The most depleting mutation is **S72Y** with **8.04** ΔΔG score.
The most enriching mutation is **E70Y** with **-2.82** ΔΔG score.
The residue most frequently leading to depleting binding is **S72.**
The residue most frequently leading to enriching binding is **E70.**

P49411
The most depleting mutation is **C31Y** with **2.07** ΔΔG score.
The most enriching mutation is **S72F** with **-3.15** ΔΔG score.
The residue most frequently leading to depleting binding is **P75.**
The residue most frequently leading to enriching binding is **P33.**

P49821
The most depleting mutation is **G91Y** with **11.37** ΔΔG score.
The most enriching mutation is **G91M** with **-3.95** ΔΔG score.
The residue most frequently leading to depleting binding is **F89.**
The residue most frequently leading to enriching binding is **E70.**

P50213
The most depleting mutation is **A28F** with **3.92** ΔΔG score.
The most enriching mutation is **G32L** with **-2.6** ΔΔG score.
The residue most frequently leading to depleting binding is **W30.**
The residue most frequently leading to enriching binding is **G32.**

P60174
The most depleting mutation is **K104Q** with **1.31** ΔΔG score.
The most enriching mutation is **Q97M** with **-2.59** ΔΔG score.
The residue most frequently leading to depleting binding is **V90.**
The residue most frequently leading to enriching binding is **Q97.**

P62269
The most depleting mutation is **K88R** with **1.78** ΔΔG score.
The most enriching mutation is **E70F** with **-2.55** ΔΔG score.
The residue most frequently leading to depleting binding is **D87.**
The residue most frequently leading to enriching binding is **D84.**

Q9BQ69
The most depleting mutation is **K93Y** with **8.73** ΔΔG score.
The most enriching mutation is **K104Y** with **-2.8** ΔΔG score.
The residue most frequently leading to depleting binding is **S72.**
The residue most frequently leading to enriching binding is **K104.**

Q9NUB1
The most depleting mutation is **D13F** with **4.22** ΔΔG score.
The most enriching mutation is **D10W** with **-3.67** ΔΔG score.
The residue most frequently leading to depleting binding is **D7.**
The residue most frequently leading to enriching binding is **D10.**

Q86WU2
The most depleting mutation is **G91R** with **11.96** ΔΔG score.
The most enriching mutation is **Q97Y** with **-2.1** ΔΔG score.
The residue most frequently leading to depleting binding is **G91.**
The residue most frequently leading to enriching binding is **Q97.**

Q13162
The most depleting mutation is **E70Y** with **4.26** ΔΔG score.
The most enriching mutation is **D84F** with **-3.52** ΔΔG score.
The residue most frequently leading to depleting binding is **G91.**
The residue most frequently leading to enriching binding is **D84.**

Q16540
The most depleting mutation is **G50N** with **6.54** ΔΔG score.
The most enriching mutation is **N4W** with **-4.58** ΔΔG score.
The residue most frequently leading to depleting binding is **G50.**
The residue most frequently leading to enriching binding is **D13.**

Q99497
The most depleting mutation is **G83W** with **9.65** ΔΔG score.
The most enriching mutation is **Q12F** with **-3.49** ΔΔG score.
The residue most frequently leading to depleting binding is **G83.**
The residue most frequently leading to enriching binding is **Q12.**

Q99798
The most depleting mutation is **P39R** with **6.17** ΔΔG score.
The most enriching mutation is **K35W** with **-2.38** ΔΔG score.
The residue most frequently leading to depleting binding is **A46.**
The residue most frequently leading to enriching binding is **R40.**

PROT-ON Summary:
Enriching residues (22): **1**, **3**, **10, 12**, **13**, **29**, **30**, **32**, **33**, 40, 48, 49, **56**, 60, 64, **70**, **72**, 84, **94**, 96, 97, **104**.
Depleting (23): **7**, **8**, **10**, **30**, **32**, **46**, **50**, 63, 67, 69, **70-73**, **75**, 83, **87**, 89, **90, 91**, 95, 96, 101.

Boxed residues are excluded from further analyses as not being recognized as contacts by BIOVIA. With red letters are the residues that are not included in interface areas as defined by the approach of contact hot spots and their network energy neighbors (Table 3).

6 common residues between "Enriching residues" and "Depleting":
10, 30, 32, 70, 72, 96.

16 residues included exclusively in "Enriching residues":
1, 3, 12, 13, 29, 33, 40, 49, 56, 60, 64, 84, 94, 97, 104.

14 residues included exclusively in "Depleting":
7, 8, 46, 50, 63, 67, 69, 71, 73, 75, 87, 90, 91, 95.

16 common residues in "Depleting" and "Covalent contacts" (or "C+Depleting"):
7, 10, 30, 32, 46, 63, 67, 69, 70, 72, 73, 75, 87, 90, 95, 96.

15 common residues in "Depleting" and "sNC contacts" (or sNC+Depleting):
7, 8, 10, 32, 46, 50, 63, 70, 72, 73, 87, 90, 91, 95, 96.

12 common residues in "C+Depleting" and "sNC+Depleting":
7, 10, 32, 46, 63, 70, 72, 73, 87, 90, 95, 96.

18 common residues in "Covalent contacts" and "Enriching" (or "C+Enriching"):
10, 13, 29, 30, 32, 33, 40, 49, 56, 60, 64, 70, 72, 84, 94, 96, 97, 104.

14 common residues in "sNC contacts" and "Enriching" (or sNC+Enriching"):
1, 3, 10, 13, 32, 33, 40, 60, 70, 72, 94, 96, 97, 104.

12 common residues in "C+Enriching" and "sNC+Enriching":
10, 13, 32, 33, 40, 60, 70, 72, 94, 96, 97, 104.

5. Positioning of residues highlighted by SpotOn, PROT-ON in the contact hot spot-energy neighbor proposed contact areas

Residues highlighted by SpotOn and PROT-ON were depicted on the interface areas proposed by the contact hot spot-energy neighbor approach (SF1, 2). The positioning was shown as a general impression on the HsTrx2 molecule (SF1) and in the context of the cleft next to the Trp preceding the active site (SF2). Some of the putative hot spots by SpotOn and PROT-ON that were not selected by the network approach (40, 42, 49, 69, 80, 81, 84, 85) were adjacent to the network contact areas (SF1).

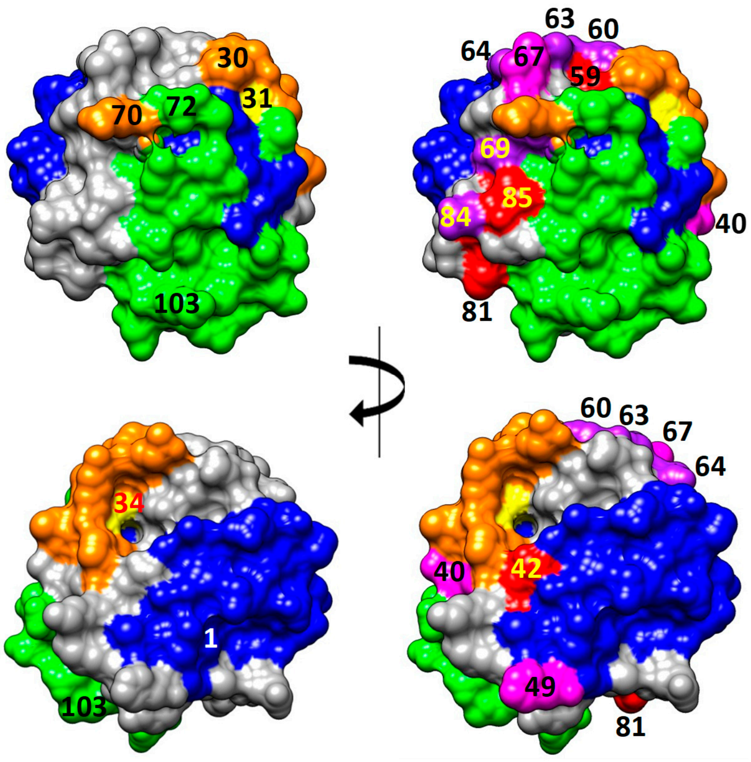

Supplementary Figure 1. Positioning of putative energy hot spots on HsTrx2 and comparison with the contact areas resulting from the network approach. The orange color corresponds to residues involved mostly in covalent interactions with substrates, the blue to strong non-covalent interactions and the green color corresponds to residues involved in both types of interactions. The left figures represent the two sides of HsTrx2 with their contact areas without the putative thermodynamic residues. The right part of the figure is as the right plus all residues identified as putative thermodynamic hot spots by SpotOn (red), PROT-ON (magenta), and both SpotOn and PROT-ON (purple). The structure of HsTrx2 is from PDB ID: 1w89, chain A with all water molecules removed [1].

The presence of another group of residues (59, 60, 63, 64, 67) was quite distinct from all contact hot spots defined areas. These residues formed almost half of the cleft located close to the active site (SF2).

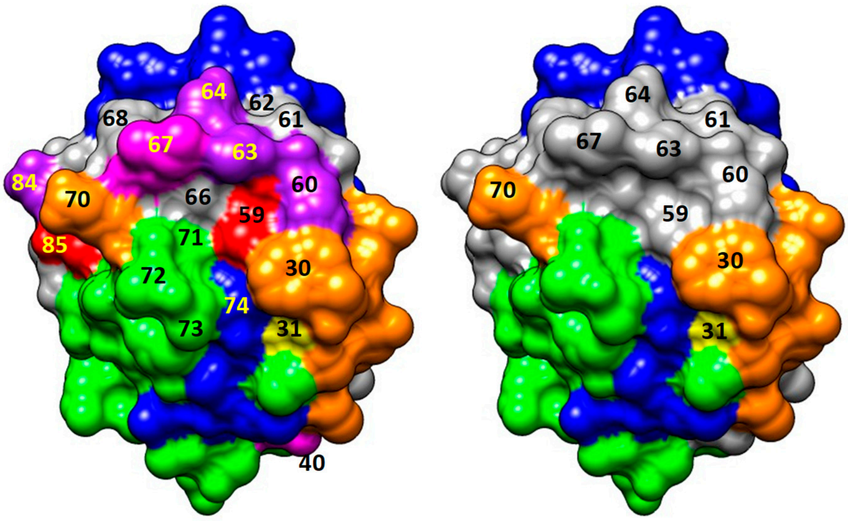

Supplementary Figure 2. The presence of putative thermodynamic hot spots by SpotOn and PROT-ON in the cleft area of HsTrx2 close to the Trp^{30} of the active site. The right figure shows the contact surfaces identified by the network approach that included all energy neighbors adjacent to contact hot spots. The orange color corresponds to residues involved mostly in covalent interactions with substrates, the blue to strong non-covalent interactions while the green color corresponds to residues involved in both types of interactions. The left figure is as the right plus all residues identified as putative thermodynamic hot spots by SpotOn (red), PROT-ON (magenta), and both SpotOn and PROT-ON (purple). The structure of HsTrx2 is from PDB ID: 1w89, chain A with all water molecules removed [1].

References

1. Smeets, A., et al., *Crystal structures of oxidized and reduced forms of human mitochondrial thioredoxin 2.* Protein Sci, 2005. **14**(10): p. 2610-21.
