# Supplementary material for: Distinct or Overlapping Areas of Mitochondrial Thioredoxin 2 May Be Used for Its Covalent and Strong Non-Covalent Interactions with Protein Ligands
Source: Antioxidants (Basel). 2023 Dec 20;13(1):15. doi: 10.3390/antiox13010015 (PMC10812433; doi:10.3390/antiox13010015)
Supplement: Supplementary file 1 [file antioxidants-13-00015-s001.zip › Supplementary data S5 (network parameters).pdf]

Supplementary Data S5: *Network parameters*

Distinct or shared areas of mitochondrial thioredoxin 2 may be used for its covalent and strong non-covalent interactions with protein ligands

Charalampos Ntallis [1], Haralambos Tzoupis [1], Theodore Tselios [1], Christos T. Chasapis [2] and Alexios Vlamis-Gardikas [1,*]

[1] Department of Chemistry, University of Patras, Rion 26504, Greece; xntallis@gmail.com, c.ntallis@uu.nl (C.N.); haralambostz@gmail.com (H.T.); ttselios@upatras.gr (T.T.)
[2] Institute of Chemical Biology, National Hellenic Research Foundation, Vas. Constantinou 48 av, Athens, 11635, Greece; cchasapis@eie.gr. (C.T.C).

*Correspondence: avlamis@upatras.gr; Tel.: +30-2610-997634

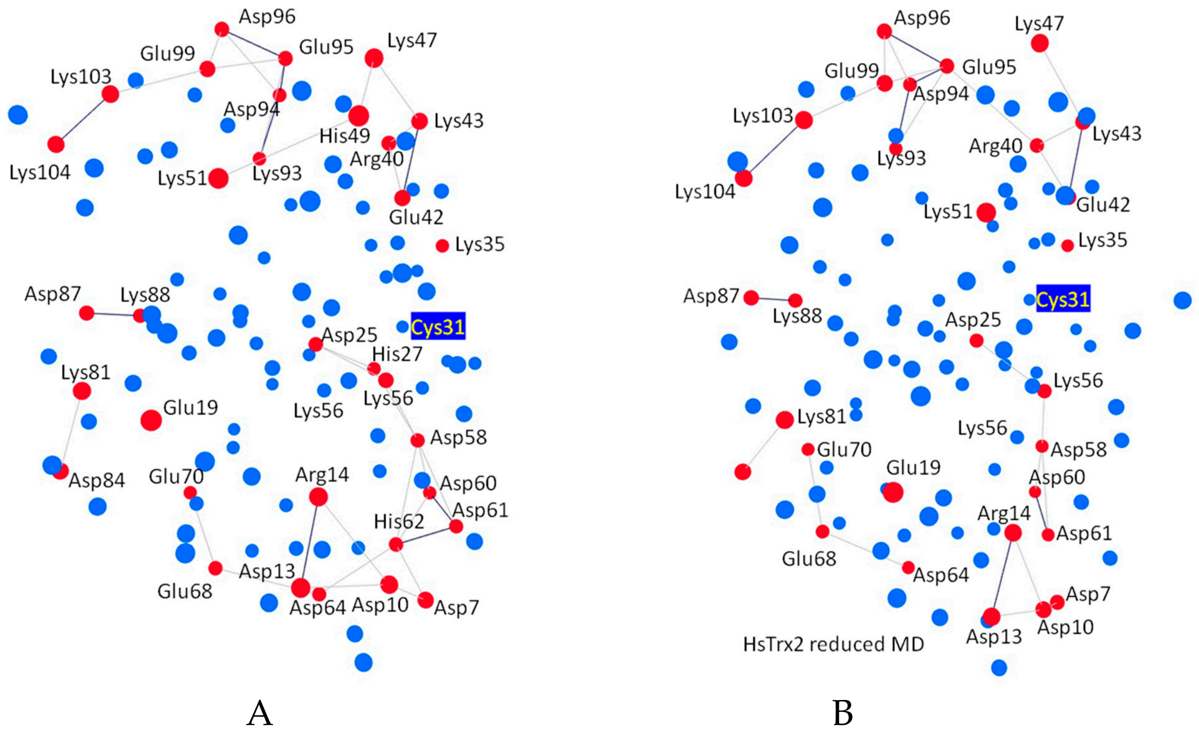

Supplementary Figure 1. Disruption of subnetworks of charged residues by protonation of His residues. All red dots represent charged residues (Glu, Lys, Asp, Arg, His). Connecting red lines represent subnetworks. Figure A corresponds to the crystal structure whereas Figure B is like A after energy minimization. Subnetworks are indicated by lines interconnecting charged residues. In Figure A, His are not protonated while in Figure B they are.

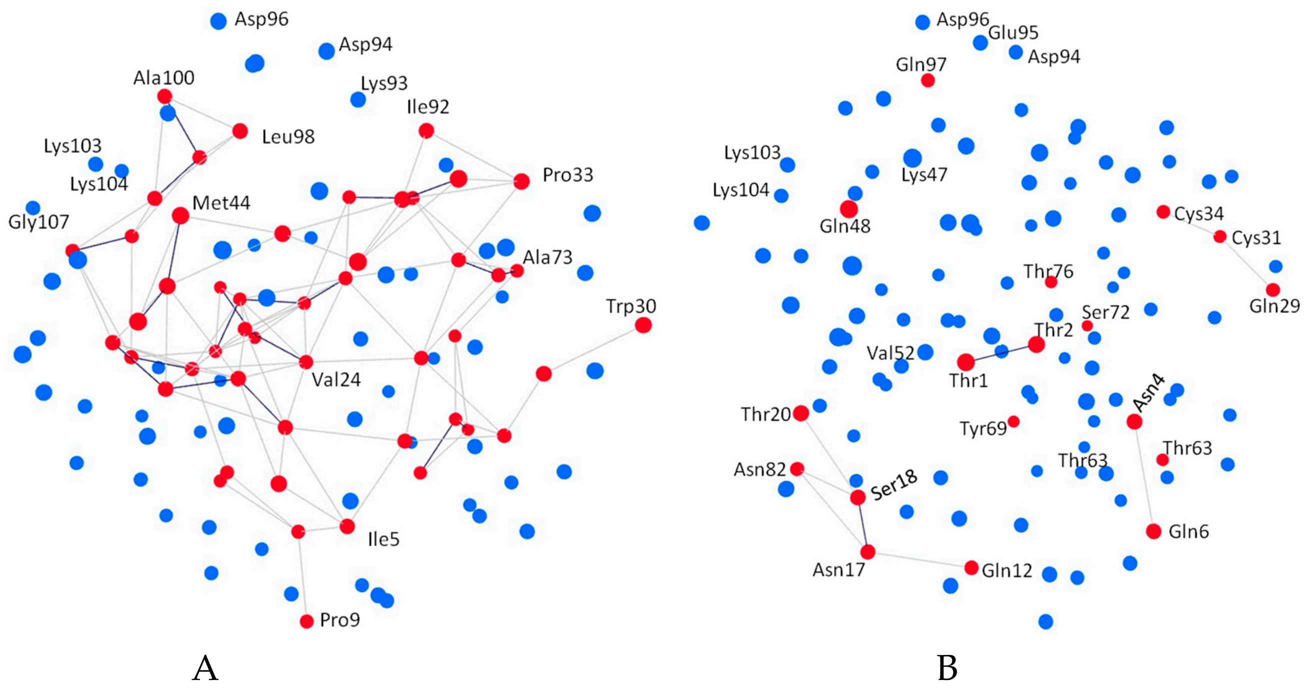

Supplementary Figure 2: Subnetworks (red dots) of the mutant C34S HsTrx2 (3D representation). **(A)** Hydrophobic residues (Ala, Ile, Leu, Val, Met, Phe, Pro, Trp). **(B)** Hydrophilic residues (Ser, Thr, Tyr, Cys, Gln, Asn).

Supplementary Table 1. The statistically significant contact residues (contact hot spots) of HsTrx2 and the corresponding residues for HsTrx1, EcoTrx1, and EcoTrx2. Residues were aligned according to Figure 8.

HsTrx2	HsTrx1		EcoTrx1		EcoTrx2	
Thr^{1}	1	M	2	D	2	E
Phe^{3}	3	K	5	I	4	I
Asp^{10}	10	A	11	S	10	T
Arg^{14}	14	A	15	D	14	L
Trp^{30}	31	W	31	W	29	W
Cys^{31}	32	C	32	C	30	C
Pro^{33}	34	P	34	P	32	P
Lys^{35}	36	K	36	K	34	R
Glu^{70}	70	E	71	G	69	G
Ser^{72}	72	K	73	R	71	R
Ala^{73}	73	C	74	G	72	S
Lys^{88}	88	E	89	T	87	M
Val^{90}	90	S	91	V	89	N
Ile^{92}	92	A	93	A	91	A

Lys^{93}	93	N	94	L	92	V
Asp^{94}	94	K	95	S	93	P
Asp^{96}	95	E	97	G	95	A
Lys^{103}	102	N	104	D	102	N
Lys^{104}	103	E	106	N	104	S

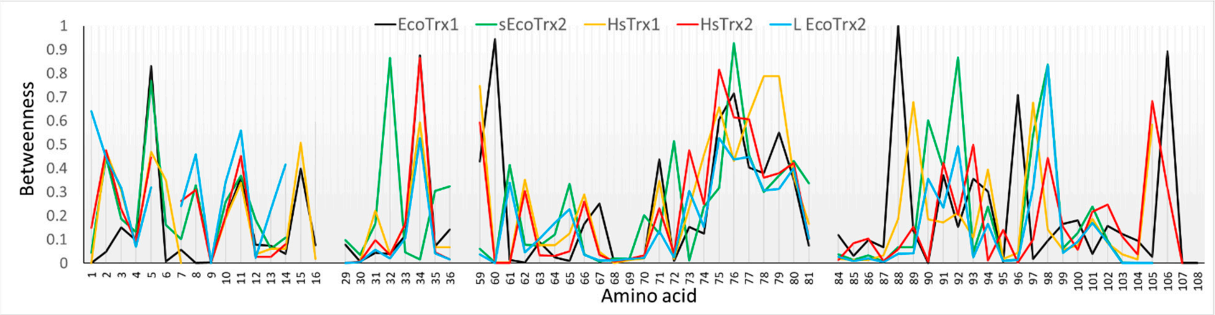

Supplementary Figure 3. Betweenness measures of four Trxs. sEcoTrx2 corresponds to the part EcoTrx2 without its first 34 amino acids, while LEcoTrx2 to the same sequence but with the first 34 amino acids being present but not shown in the figure. All Trxs are aligned according to HsTrx2 (Figure 8). The contact hot spot residues the interactions of HsTrx2 were 1, 3, 10, 14, 30, 31, 33, 35, 70, 72, 73, 88, 90 92, 93, 94, 96, 103, 104. Residues 74-80 are not accessible to solvent.

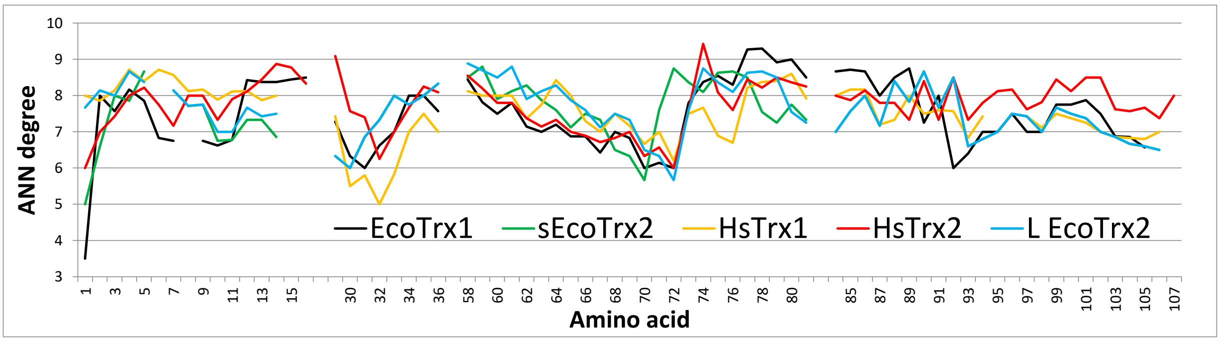

Supplementary Figure 4. Average Nearest neighbor degree (ANN degree) plot for the four Trxs. sEcoTrx2 corresponds to the part EcoTrx2 without its first 34 amino acids, while LEcoTrx2 to the same sequence but with the first 34 amino acids being present but not shown in the figure. All Trxs are aligned according to HsTrx2 (Figure 8). The contact hot spots of HsTrx2 were 1, 3, 10, 14, 30, 31, 33, 35, 70, 72, 73, 88, 90 92, 93, 94, 96, 103, 104. Residues 74-80 are not accessible to solvent. The numbering on the X axis corresponds to the numbering of the amino acids of HsTrx2.
