# Supplementary material for: Distinct or Overlapping Areas of Mitochondrial Thioredoxin 2 May Be Used for Its Covalent and Strong Non-Covalent Interactions with Protein Ligands
Source: Antioxidants (Basel). 2023 Dec 20;13(1):15. doi: 10.3390/antiox13010015 (PMC10812433; doi:10.3390/antiox13010015)
Supplement: Supplementary file 1 [file antioxidants-13-00015-s001.zip › Supplementary data S6 (contacts in crystals EcoTrx1, HsTrx1).pdf]

Supplementary Data S6: *Contact residues of EcoTrx1 and HsTrx1 in their crystal complexes with protein ligands*

Distinct or shared areas of mitochondrial thioredoxin 2 may be used for its covalent and strong non-covalent interactions with protein ligands

Charalampos Ntallis [1]. Haralambos Tzoupis [1]. Theodore Tselios [1]. Christos T. Chasapis [2] and Alexios Vlamis-Gardikas [1,*]

[1] Department of Chemistry. University of Patras. Rion 26504. Greece; xntallis@gmail.com. c.ntallis@uu.nl (C.N.); haralambostz@gmail.com (H.T.); ttselios@upatras.gr (T.T.)
[2] Institute of Chemical Biology. National Hellenic Research Foundation. Vas. Constantinou 48 av. Athens. 11635. Greece; cchasapis@eie.gr. (C.T.C).

*Correspondence: avlamis@upatras.gr; Tel.: +30-2610-997634

1. Contact residues of EcoTrx1 in its crystal complexes with protein ligands

1.1. T7 DNA polymerase (PDB ID: 1T7P): a complex of EcoTrx1 with G5p [1].

Interacting residues (G5p-EcoTrx1)	Category (type)	From (chemistry)	To (chemistry)	Distance (Å)
G5p:THR327:N - EcoTrx1:ILE75:O	Hydrogen Bond (conventional)	G5p:THR327:N (H-donor)	EcoTrx1:ILE75:O (H-acceptor)	2.90286
G5p:THR327:OG1 - EcoTrx1:CYS32:SG	Hydrogen Bond (conventional)	G5p:THR327:OG1 (H-donor)	EcoTrx1:CYS32:SG (H-acceptor)	2.994
G5p:VAL329:N - EcoTrx1:ARG73:O	Hydrogen Bond (conventional)	G5p:VAL329:N (H-donor)	EcoTrx1:ARG73:O (H-acceptor)	3.0883
EcoTrx1:ARG73:NH2 - G5p:TYR320:OH	Hydrogen Bond (conventional)	EcoTrx1:ARG73:NH2 (H-donor)	G5p:TYR320:OH (H-acceptor)	2.86865
EcoTrx1:ARG73:NH2 - G5p:TYR326:OH	Hydrogen Bond (conventional)	EcoTrx1:ARG73:NH2 (H-donor)	G5p:TYR326:OH (H-acceptor)	3.28787
EcoTrx1:ILE75:N - G5p:THR327:O	Hydrogen Bond (conventional)	EcoTrx1:ILE75:N (H-donor)	G5p:THR327:O (H-acceptor)	3.02215
EcoTrx1:THR89:OG1 - G5p:GLU319:OE1	Hydrogen Bond (conventional)	EcoTrx1:THR89:OG1 (H-donor)	G5p:GLU319:OE1 (H-acceptor)	3.10654
EcoTrx1:VAL91:N - G5p:GLU319:O	Hydrogen Bond (conventional)	EcoTrx1:VAL91:N (H-donor)	G5p:GLU319:O (H-acceptor)	2.92813
EcoTrx1:ALA93:N - G5p:PRO325:O	Hydrogen Bond (conventional)	EcoTrx1:ALA93:N (H-donor)	G5p:PRO325:O (H-acceptor)	2.78199
G5p:PRO287:CD - EcoTrx1:TRP31:O	Hydrogen Bond (carbon)	G5p:PRO287:CD (H-donor)	EcoTrx1:TRP31:O (H-acceptor)	3.08175
EcoTrx1:PRO34:CD - G5p:THR327:OG1	Hydrogen Bond (carbon)	EcoTrx1:PRO34:CD (H-donor)	G5p:THR327:OG1 (H-acceptor)	3.47822
G5p:PRO277 - EcoTrx1:MET37	Hydrophobic (alkyl)	G5p:PRO277 (alkyl)	EcoTrx1:MET37 (alkyl)	4.81137
G5p:VAL321 - EcoTrx1:LEU94	Hydrophobic (alkyl)	G5p:VAL321 (alkyl)	EcoTrx1:LEU94 (alkyl)	4.75678
G5p:ALA324 - EcoTrx1:LEU94	Hydrophobic (alkyl)	G5p:ALA324 (alkyl)	EcoTrx1:LEU94 (alkyl)	4.65972
G5p:PRO325 - EcoTrx1:PRO34	Hydrophobic (alkyl)	G5p:PRO325 (alkyl)	EcoTrx1:PRO34 (alkyl)	5.03292
G5p:VAL329 - EcoTrx1:ILE75	Hydrophobic (alkyl)	G5p:VAL329 (alkyl)	EcoTrx1:ILE75 (alkyl)	4.66883
EcoTrx1:PRO34 - G5p:ILE289	Hydrophobic (alkyl)	EcoTrx1:PRO34 (alkyl)	G5p:ILE289 (alkyl)	5.28245
EcoTrx1:ALA93 - G5p:PRO325	Hydrophobic (alkyl)	EcoTrx1:ALA93 (alkyl)	G5p:PRO325 (alkyl)	4.38959
G5p:TYR265 - EcoTrx1:ILE60	Hydrophobic (Pi-alkyl)	G5p:TYR265 (Pi-orbitals)	EcoTrx1:ILE60 (alkyl)	5.12054
G5p:TYR265 - EcoTrx1:ALA67	Hydrophobic (Pi-alkyl)	G5p:TYR265 (Pi-orbitals)	EcoTrx1:ALA67 (alkyl)	4.67645
G5p:TYR265 - EcoTrx1:PRO68	Hydrophobic (Pi-alkyl)	G5p:TYR265 (Pi-orbitals)	EcoTrx1:PRO68 (alkyl)	5.38793
G5p:PHE274 - EcoTrx1:PRO34	Hydrophobic (Pi-alkyl)	G5p:PHE274 (Pi-orbitals)	EcoTrx1:PRO34 (alkyl)	5.30569

G5p:TYR320 - EcoTrx1:VAL91	Hydrophobic (Pi-alkyl)	G5p:TYR320 (Pi-orbitals)	EcoTrx1:VAL91 (alkyl)	4.82123
G5p:TYR326 - EcoTrx1:VAL91	Hydrophobic (Pi-alkyl)	G5p:TYR326 (Pi-orbitals)	EcoTrx1:VAL91 (alkyl)	5.40115
G5p:HIS331 - EcoTrx1:PRO68	Hydrophobic (Pi-alkyl)	G5p:HIS331 (Pi-orbitals)	EcoTrx1:PRO68 (alkyl)	4.30139
EcoTrx1:TRP31 - G5p:PRO267	Hydrophobic (Pi-alkyl)	EcoTrx1:TRP31 (Pi-orbitals)	G5p:PRO267 (alkyl)	4.95059
EcoTrx1:MET37:SD - G5p:PHE274	Other (Pi-sulfur)	EcoTrx1:MET37:SD (sulfur)	G5p:PHE274 (Pi-orbitals)	5.19383

EcoTrx1 interacting residues	Electrostatic interaction	Hydrogen bonding	Salt bridge	Hydrophobic interaction	Other
TRP31	-	+	-	+	-
CYS32	-	+	-	-	-
PRO34	-	+	-	+	-
MET37	-	-	-	+	+
ILE60	-	-	-	+	-
ALA67	-	-	-	+	-
PRO68	-	-	-	+	-
ARG73	-	+	-	-	-
ILE75	-	+	-	+	-
THR89	-	+	-	-	-
VAL91	-	+	-	+	-
ALA93	-	+	-	+	-
LEU94	-	-	-	+	-

1.2. TrxR-EcoTrx1 complex (PDB: 1F6M) [2].

Interacting residues (TrxR-EcoTrx1)	Category (type)	From (chemistry)	To (chemistry)	Distance (Å)
TrxR:ARG130:NH2 – EcoTrx1:TYR70:O	Hydrogen Bond (conventional)	TrxR:ARG130:NH2 (H-donor)	EcoTrx1:TYR70:O (H-acceptor)	2.60149
EcoTrx1:TRP31:NE1 – TrxR:CYS138:O	Hydrogen Bond (conventional)	EcoTrx1:TRP31:NE1 (H-donor)	TrxR:CYS138:O (H-acceptor)	3.13459
EcoTrx1:ARG73:N – TrxR:GLY129:O	Hydrogen Bond (conventional)	EcoTrx1:ARG73:N (H-donor)	TrxR:GLY129:O (H-acceptor)	3.25012
EcoTrx1:ARG73:NE – TrxR:ARG130:O	Hydrogen Bond (conventional)	EcoTrx1:ARG73:NE (H-donor)	TrxR:ARG130:O (H-acceptor)	3.07444
EcoTrx1:ARG73:NH1 – TrxR:ARG130:O	Hydrogen Bond (conventional)	EcoTrx1:ARG73:NH1 (H-donor)	TrxR:ARG130:O (H-acceptor)	2.85782
EcoTrx1:ARG73:NH1 – TrxR:ALA237:O	Hydrogen Bond (conventional)	EcoTrx1:ARG73:NH1 (H-donor)	TrxR:ALA237:O (H-acceptor)	2.71026
EcoTrx1:ILE75:N – TrxR:ASP139:OD1	Hydrogen Bond (conventional)	EcoTrx1:ILE75:N (H-donor)	TrxR:ASP139:OD1 (H-acceptor)	2.78878
EcoTrx1:TRP31:CD1 – TrxR:THR137:O	Hydrogen Bond (carbon)	EcoTrx1:TRP31:CD1 (H-donor)	TrxR:THR137:O (H-acceptor)	3.49543

EcoTrx1:ARG73:C.O;GLY74:N – TrxR:PHE142	Hydrophobic (amide-Pi stacked)	EcoTrx1:ARG73:C.O;GLY74:N (amide)	TrxR:PHE142 (Pi-orbitals)	3.91293
TrxR:LYS39 – EcoTrx1:MET37	Hydrophobic (alkyl)	TrxR:LYS39 (alkyl)	EcoTrx1:MET37 (alkyl)	4.67061
TrxR:CYS138 – EcoTrx1:PRO34	Hydrophobic (alkyl)	TrxR:CYS138 (alkyl)	EcoTrx1:PRO34 (alkyl)	5.06124
TrxR:CYS138 – EcoTrx1:ILE75	Hydrophobic (alkyl)	TrxR:CYS138 (alkyl)	EcoTrx1:ILE75 (alkyl)	5.48344
EcoTrx1:ALA93 – TrxR:MET37	Hydrophobic (alkyl)	EcoTrx1:ALA93 (alkyl)	TrxR:MET37 (alkyl)	4.57981
TrxR:PHE81 – EcoTrx1:MET37	Hydrophobic (Pi-alkyl)	TrxR:PHE81 (Pi-orbitals)	EcoTrx1:MET37 (alkyl)	5.33256
TrxR:PHE81 – EcoTrx1:PRO40	Hydrophobic (Pi-alkyl)	TrxR:PHE81 (Pi-orbitals)	EcoTrx1:PRO40 (alkyl)	5.46234
TrxR:PHE142 – EcoTrx1:ARG73	Hydrophobic (Pi-alkyl)	TrxR:PHE142 (Pi-orbitals)	EcoTrx1:ARG73 (alkyl)	4.64594
TrxR:PHE142 – EcoTrx1:ILE75	Hydrophobic (Pi-alkyl)	TrxR:PHE142 (Pi-orbitals)	EcoTrx1:ILE75 (alkyl)	5.44979
TrxR:TYR143 – EcoTrx1:ARG73	Hydrophobic (Pi-alkyl)	TrxR:TYR143 (Pi-orbitals)	EcoTrx1:ARG73 (alkyl)	5.46537

EcoTrx1 interacting residues	Electrostatic interaction	Hydrogen bonding	Salt bridge	Hydrophobic interaction	Other
TRP31	-	+	-	-	-
PRO34	-	-	-	+	-
MET37	-	-	-	+	-
PRO40	-	-	-	+	-
TYR70	-	+	-	-	-
ARG73	-	+	-	+	-
ILE75	-	+	-	+	-
ALA93	-	-	-	+	-

1.3. MsrA-EcoTrx1 (PDB: 6YEV).

Interacting residues (MsrA-EcoTrx1)	Category (type)	From (chemistry)	To (chemistry)	Distance (Å)
EcoTrx1:LYS90:NZ – MsrA:ASP164:OD2	Electrostatic (Attractive Charge)	EcoTrx1:LYS90:NZ (Positive)	MsrA:ASP164:OD2 (Negative)	5.5975
EcoTrx1:ALA93:N – MsrA:GLY204:O	Hydrogen Bond (conventional)	EcoTrx1:ALA93:N (H-Donor)	MsrA:GLY204:O (H-Acceptor)	2.83316
MsrA:CYS206:N – EcoTrx1:ILE75:O	Hydrogen Bond (conventional)	MsrA:CYS206:N (H-Donor)	EcoTrx1:ILE75:O (H-Acceptor)	3.00363
EcoTrx1:PRO34:CD – MsrA:GLU116:OE2	Hydrogen Bond (carbon)	EcoTrx1:PRO34:CD (H-Donor)	MsrA:GLU116:OE2 (H-Acceptor)	3.73592
MsrA:PRO208:CD – EcoTrx1:ARG73:O	Hydrogen Bond (carbon)	MsrA:PRO208:CD (H-Donor)	EcoTrx1:ARG73:O (H-Acceptor)	3.29517
MsrA:ALA162 – EcoTrx1:VAL91	Hydrophobic (alkyl)	MsrA:ALA162 (Alkyl)	EcoTrx1:VAL91 (Alkyl)	4.2169

MsrA:ALA163 – EcoTrx1:VAL91	Hydrophobic (alkyl)	MsrA:ALA163 (Alkyl)	EcoTrx1:VAL91 (Alkyl)	4.08484
MsrA:CYS206 – EcoTrx1:PRO34	Hydrophobic (alkyl)	MsrA:CYS206 (Alkyl)	EcoTrx1:PRO34 (Alkyl)	4.83189
MsrA:PRO208 – EcoTrx1:ILE75	Hydrophobic (alkyl)	MsrA:PRO208 (Alkyl)	EcoTrx1:ILE75 (Alkyl)	5.41164
EcoTrx1:TRP31 – MsrA:CYS206	Hydrophobic (Pi-alkyl)	EcoTrx1:TRP31 (Pi-Orbitals)	MsrA:CYS206 (Alkyl)	5.10675

EcoTrx1 interacting residues	Electrostatic interaction	Hydrogen bonding	Salt bridge	Hydrophobic interaction	Other
TRP31				+	
PRO34		+		+	
ARG73		+			
ILE75		+		+	
LYS90	+				
VAL91				++	
ALA93		+			

1.4. PAPS reductase-EcoTrx1 (PDB: 2O8V) [3].

Interacting residues (MsrA-EcoTrx1)	Category (type)	From (chemistry)	To (chemistry)	Distance (Å)
EcoTrx1:LYS36:NZ – PAPS red:ASP206:OD1	Hydrogen Bond;Electrostatic (Salt Bridge)	EcoTrx1:LYS36:NZ (H-donor)	PAPS red:ASP206:OD1 (H-acceptor)	2.82895
EcoTrx1:ARG73:NH1 – PAPS red:GLU238:OE2	Hydrogen Bond;Electrostatic (Salt Bridge)	EcoTrx1:ARG73:NH1 (H-donor)	PAPS red:GLU238:OE2 (H-acceptor)	2.86245
EcoTrx1:ARG73:NH2 – PAPS red:GLU243:OE2	Hydrogen Bond;Electrostatic (Salt Bridge)	EcoTrx1:ARG73:NH2 (H-donor)	PAPS red:GLU243:OE2 (H-acceptor)	2.6723
EcoTrx1:ARG73:NH1 – PAPS red:GLU243:OE1	Electrostatic (Attractive Charge)	EcoTrx1:ARG73:NH1 (positive)	PAPS red:GLU243:OE1 (negative)	3.99202
EcoTrx1:ARG73:NH2 – PAPS red:GLU238:OE1	Electrostatic (Attractive Charge)	EcoTrx1:ARG73:NH2 (positive)	PAPS red:GLU238:OE1 (negative)	2.97327
PAPS red:TRP205:NE1 – EcoTrx1:GLU30:O	Hydrogen Bond (conventional)	PAPS red:TRP205:NE1 (H-donor)	EcoTrx1:GLU30:O (H-acceptor)	2.95719
PAPS red:CYS239:N – EcoTrx1:ILE75:O	Hydrogen Bond (conventional)	PAPS red:CYS239:N (H-donor)	EcoTrx1:ILE75:O (H-acceptor)	3.05945
PAPS red:LEU241:N – EcoTrx1:ARG73:O	Hydrogen Bond (conventional)	PAPS red:LEU241:N (H-donor)	EcoTrx1:ARG73:O (H-acceptor)	3.38506
EcoTrx1:LYS36:NZ – PAPS red:TRP205:O	Hydrogen Bond (conventional)	EcoTrx1:LYS36:NZ (H-donor)	PAPS red:TRP205:O (H-acceptor)	3.28943
EcoTrx1:ARG73:NE – PAPS red:GLU243:OE1	Hydrogen Bond (conventional)	EcoTrx1:ARG73:NE (H-donor)	PAPS red:GLU243:OE1	2.73015

			(H-acceptor)	
EcoTrx1:ALA93:N – PAPS red:ARG237:O	Hydrogen Bond (conventional)	EcoTrx1:ALA93:N (H-donor)	PAPS red:ARG237:O (H-acceptor)	2.85509
EcoTrx1:LYS36:NZ – PAPS red:TRP205	Electrostatic (Pi-Cation)	EcoTrx1:LYS36:NZ (positive)	PAPS red:TRP205 (Pi-orbitals)	4.59989
PAPS red:ASN187:C.O;ARG188:N – EcoTrx1:TRP31	Hydrophobic (Amide-Pi Stacked)	PAPS red:ASN187:C.O;ARG188:N (amide)	EcoTrx1:TRP31 (Pi-orbitals)	4.35499
PAPS red:ASN187:C.O;ARG188:N – EcoTrx1:TRP31	Hydrophobic (Amide-Pi Stacked)	PAPS red:ASN187:C.O;ARG188:N (amide)	EcoTrx1:TRP31 (Pi-orbitals)	4.67411
PAPS red:CYS239 – EcoTrx1:PRO34	Hydrophobic (alkyl)	PAPS red:CYS239 (alkyl)	EcoTrx1:PRO34 (alkyl)	4.8634
PAPS red:LEU241 – EcoTrx1:ILE60	Hydrophobic (alkyl)	PAPS red:LEU241 (alkyl)	EcoTrx1:ILE60 (alkyl)	5.37286
PAPS red:LEU241 – EcoTrx1:ILE75	Hydrophobic (alkyl)	PAPS red:LEU241 (alkyl)	EcoTrx1:ILE75 (alkyl)	5.02976
EcoTrx1:PRO34 – PAPS red:LEU235	Hydrophobic (alkyl)	EcoTrx1:PRO34 (alkyl)	PAPS red:LEU235 (alkyl)	4.33625
PAPS red:TRP205 – EcoTrx1:LYS36	Hydrophobic (Pi-alkyl)	PAPS red:TRP205 (Pi-orbitals)	EcoTrx1:LYS36 (alkyl)	5.14881
PAPS red:TRP205 – EcoTrx1:LYS36	Hydrophobic (Pi-alkyl)	PAPS red:TRP205 (Pi-orbitals)	EcoTrx1:LYS36 (alkyl)	5.16558
EcoTrx1:TRP31 – PAPS red:ARG188	Hydrophobic (Pi-alkyl)	EcoTrx1:TRP31 (Pi-orbitals)	PAPS red:ARG188 (alkyl)	4.47438
EcoTrx1:TRP31 – PAPS red:ARG188	Hydrophobic (Pi-alkyl)	EcoTrx1:TRP31 (Pi-orbitals)	PAPS red:ARG188 (alkyl)	4.03231
EcoTrx1:TRP31 – PAPS red:CYS239	Hydrophobic (Pi-alkyl)	EcoTrx1:TRP31 (Pi-orbitals)	PAPS red:CYS239 (alkyl)	5.07597

EcoTrx1 interacting residues	Electrostatic interaction	Hydrogen bonding	Salt bridge	Hydrophobic interaction	Other
GLU30		+			
TRP31				+++++	
PRO34				++	
LYS36	+	+	+	++	
ILE60				+	
ARG73	++	++	++		
ILE75		+		+	
ALA93		+			

2. Contact residues of HsTrx1 in its crystal complexes with protein ligands

2.1. HsTrx1-SlrP (PDB: 4PUF) [4].

Interacting residues (SlrP-HsTrx1)	Category (type)	From (chemistry)	To (chemistry)	Distance (Å)
SlrP:TYR424:OH - HsTrx1:GLU88:O	Hydrogen Bond (conventional)	SlrP:TYR424:OH (H-donor)	HsTrx1:GLU88:O (H-Acceptor)	2.46475
SlrP:SER457:N - HsTrx1:MET74:O	Hydrogen Bond (conventional)	SlrP:SER457:N (H-donor)	HsTrx1:MET74:O (H-Acceptor)	2.63933
SlrP:VAL459:N - HsTrx1:LYS72:O	Hydrogen Bond (conventional)	SlrP:VAL459:N (H-donor)	HsTrx1:LYS72:O (H-Acceptor)	3.35557
HsTrx1:ALA92:N - SlrP:ASP455:O	Hydrogen Bond (conventional)	HsTrx1:ALA92:N (H-donor)	SlrP:ASP455:O (H-Acceptor)	2.63262
HsTrx1:PRO75:CD - SlrP:ASP455:O	Hydrogen Bond (carbon)	HsTrx1:PRO75:CD (H-donor)	SlrP:ASP455:O (H-Acceptor)	3.69592
HsTrx1:CYS73:SG - SlrP:PHE456	Other Pi-Sulfur	HsTrx1:CYS73:SG (Sulfur)	SlrP:PHE456 (Pi-orbitals)	4.44873
SlrP:VAL459 - HsTrx1:VAL59	Hydrophobic (alkyl)	SlrP:VAL459 (alkyl)	HsTrx1:VAL59 (alkyl)	4.64785
SlrP:VAL459 - HsTrx1:MET74	Hydrophobic (alkyl)	SlrP:VAL459 (alkyl)	HsTrx1:MET74 (alkyl)	4.87158
HsTrx1:LYS96 - SlrP:MET453	Hydrophobic (alkyl)	HsTrx1:LYS96 (alkyl)	SlrP:MET453 (alkyl)	4.26638

HsTrx1 interacting residues	Electrostatic interaction	Hydrogen bonding	Salt bridge	Hydrophobic interaction	Other
VAL59				+	
LYS72		+			
CYS73					+
MET74		+		+	
PRO75		+			
GLU88		+			
ALA92		+			
LYS96				+	

2.2. HsTrx1-TrxR (PDB: 3QFA) [5].

Interacting residues (TrxR-HsTrx1)	Category (type)	From (chemistry)	To (chemistry)	Distance (Å)
TrxR:ARG121:NH1 - HsTrx1:GLU70:OE2	Hydrogen Bond;Electrostatic (Salt Bridge)	TrxR:ARG121:NH1 (H-donor)	HsTrx1:GLU70:OE2	3.74249
TrxR:ARG117:NH1 - HsTrx1:ASP60:OD1	Electrostatic (attractive charge)	TrxR:ARG117:NH1 (positive)	HsTrx1:ASP60:OD1	5.19949
TrxR:ARG121:NH2 - HsTrx1:GLU70:OE1	Electrostatic (attractive charge)	TrxR:ARG121:NH2 (positive)	HsTrx1:GLU70:OE1	5.53065
TrxR:LYS124:NZ - HsTrx1:GLU70:OE1	Electrostatic (attractive charge)	TrxR:LYS124:NZ (positive)	HsTrx1:GLU70:OE1	5.0851

HsTrx1:LYS36:NZ - TrxR:GLU103:OE2	Electrostatic (attractive charge)	HsTrx1:LYS36:NZ (positive)	TrxR:GLU103:OE2	5.00342
HsTrx1:LYS72:NZ - TrxR:GLU122:OE2	Electrostatic (attractive charge)	HsTrx1:LYS72:NZ (positive)	TrxR:GLU122:OE2	3.91194
TrxR:TRP114:NE1 - HsTrx1:LYS72:O	Hydrogen Bond (conventional)	TrxR:TRP114:NE1 (H-donor)	HsTrx1:LYS72:O (H-acceptor)	2.72736
TrxR:ARG117:NH2 - HsTrx1:VAL59:O	Hydrogen Bond (conventional)	TrxR:ARG117:NH2 (H-donor)	HsTrx1:VAL59:O (H-acceptor)	2.76195
TrxR:ARG121:NH2 - HsTrx1:VAL71:O	Hydrogen Bond (conventional)	TrxR:ARG121:NH2 (H-donor)	HsTrx1:VAL71:O (H-acceptor)	2.91987
HsTrx1:MET74:CE - TrxR:TRP114	Hydrophobic (Pi-Sigma)	HsTrx1:MET74:CE (C-H)	TrxR:TRP114 (Pi-orbitals)	3.22536
HsTrx1:MET74:CE - TrxR:TRP114	Hydrophobic (Pi-Sigma)	HsTrx1:MET74:CE (C-H)	TrxR:TRP114 (Pi-orbitals)	3.99918
TrxR:GLY110:C.O;SER111:N - HsTrx1:TRP31	Hydrophobic (amide-Pi stacked)	TrxR:GLY110:C.O;SER111:N (amide)	HsTrx1:TRP31 (Pi-orbitals)	3.79927
TrxR:GLY110:C.O;SER111:N - HsTrx1:TRP31	Hydrophobic (amide-Pi stacked)	TrxR:GLY110:C.O;SER111:N (amide)	HsTrx1:TRP31 (Pi-orbitals)	3.80979
TrxR:VAL118 - HsTrx1:LYS72	Hydrophobic (alkyl)	TrxR:VAL118 (alkyl)	HsTrx1:LYS72 (alkyl)	5.01552
TrxR:TRP114 - HsTrx1:VAL59	Hydrophobic (Pi-alkyl)	TrxR:TRP114 (Pi-orbitals)	HsTrx1:VAL59 (alkyl)	5.18936
TrxR:TRP114 - HsTrx1:VAL59	Hydrophobic (Pi-alkyl)	TrxR:TRP114 (Pi-orbitals)	HsTrx1:VAL59 (alkyl)	4.12479
TrxR:TRP114 - HsTrx1:ALA66	Hydrophobic (Pi-alkyl)	TrxR:TRP114 (Pi-orbitals)	HsTrx1:ALA66 (alkyl)	4.70456
TrxR:TRP114 - HsTrx1:VAL71	Hydrophobic (Pi-alkyl)	TrxR:TRP114 (Pi-orbitals)	HsTrx1:VAL71 (alkyl)	5.30522

HsTrx1 interacting residues	Electrostatic interaction	Hydrogen bonding	Salt bridge	Hydrophobic interaction	Other
TRP31				++	
LYS36	+				
VAL59		+		++	
ASP60	+				
ALA66				+	
GLU70	++		+		
VAL71		+		+	
LYS72	+	+		+	
MET74				+	

2.3. HsTrx1-Txnip (PDB: 4LL4) [6]

Interacting residues (Txnip-HsTrx1)	Category (type)	From (chemistry)	To (chemistry)	Distance (Å)
Txnip:ARG251:NH2 - HsTrx1:ASP60:OD2	Hydrogen Bond;Electrostatic (Salt Bridge)	Txnip:ARG251:NH2 (H-donor)	HsTrx1:ASP60:OD2 (H-acceptor)	3.00975
Txnip:CYS247:N -	Hydrogen Bond	Txnip:CYS247:N	HsTrx1:MET74:O	2.92279

HsTrx1:MET74:O	(conventional)	(H-donor)	(H-acceptor)	
HsTrx1:GLY33:N - Txnip:GLU202:OE2	Hydrogen Bond (conventional)	HsTrx1:GLY33:N (H-donor)	Txnip:GLU202:OE2 (H-acceptor)	3.14123
HsTrx1:MET74:N - Txnip:CYS247:O	Hydrogen Bond (conventional)	HsTrx1:MET74:N (H-donor)	Txnip:CYS247:O (H-acceptor)	2.85886
HsTrx1:ALA92:N - Txnip:GLY245:O	Hydrogen Bond (conventional)	HsTrx1:ALA92:N (H-donor)	Txnip:GLY245:O (H-acceptor)	3.17731
Txnip:ASP200:OD1 - HsTrx1:TRP31	Electrostatic (Pi-anion)	Txnip:ASP200:OD1 (negative)	HsTrx1:TRP31 (Pi-orbitals)	3.94097
Txnip:ASP200:OD2 - HsTrx1:TRP31	Electrostatic (Pi-anion)	Txnip:ASP200:OD2 (negative)	HsTrx1:TRP31 (Pi-orbitals)	3.87899
Txnip:CYS247 - HsTrx1:CYS32	Hydrophobic (alkyl)	Txnip:CYS247 (alkyl)	HsTrx1:CYS32 (alkyl)	3.30414
Txnip:CYS247 - HsTrx1:PRO34	Hydrophobic (alkyl)	Txnip:CYS247 (alkyl)	HsTrx1:PRO34 (alkyl)	4.98945
Txnip:CYS247 - HsTrx1:MET74	Hydrophobic (alkyl)	Txnip:CYS247 (alkyl)	HsTrx1:MET74 (alkyl)	5.44584
Txnip:ALA248 - HsTrx1:CYS73	Hydrophobic (alkyl)	Txnip:ALA248 (alkyl)	HsTrx1:CYS73 (alkyl)	5.00429
HsTrx1:TRP31 - Txnip:CYS247	Hydrophobic (Pi-alkyl)	HsTrx1:TRP31 (Pi-orbitals)	Txnip:CYS247 (alkyl)	4.7896

HsTrx1 interacting residues	Electrostatic interaction	Hydrogen bonding	Salt bridge	Hydrophobic interaction	Other
TRP31	++			+	
CYS32				+	
GLY33		+			
PRO34				+	
ASP60	+	+			
CYS73				+	
MET74		++		+	
AΛA92		+			

References

1. Doublie, S., et al., *Crystal structure of a bacteriophage T7 DNA replication complex at 2.2 A resolution.* Nature, 1998. **391**(6664): p. 251-8.
2. Lennon, B.W., C.H. Williams, Jr., and M.L. Ludwig, *Twists in catalysis: alternating conformations of Escherichia coli thioredoxin reductase.* Science, 2000. **289**(5482): p. 1190-4.
3. Chartron, J., et al., *3'-Phosphoadenosine-5'-phosphosulfate reductase in complex with thioredoxin: a structural snapshot in the catalytic cycle.* Biochemistry, 2007. **46**(13): p. 3942-51.
4. Zouhir, S., et al., *The structure of the Slrp-Trx1 complex sheds light on the autoinhibition mechanism of the type III secretion system effectors of the NEL family.* Biochem J, 2014. **464**(1): p. 135-44.
5. Fritz-Wolf, K., et al., *Crystal structure of the human thioredoxin reductase-thioredoxin complex.* Nat Commun, 2011. **2**: p. 383.
6. Hwang, J., et al., *The structural basis for the negative regulation of thioredoxin by thioredoxin-interacting protein.* Nat Commun, 2014. **5**: p. 2958.
